# Development and external validation of machine learning models for the early prediction of malnutrition in critically ill patients: a prospective observational study

**DOI:** 10.1186/s12911-025-03082-9

**Published:** 2025-07-03

**Authors:** Yi Liu, Yehua Xu, Lixia Guo, Zhongbin Chen, Xueqin Xia, Feng Chen, Li Tang, Hua Jiang, Caixia Xie

**Affiliations:** 1 Department of Nursing, Sichuan Provincial People’s Hospital, University of Electronic Science and Technology of China, Chengdu, China; 2 Department of Emergency Intensive Care Unit, Sichuan Provincial People’s Hospital, University of Electronic Science and Technology of China, Chengdu, China; 3 Department of Neurology, Sichuan Provincial People’s Hospital, University of Electronic Science and Technology of China, Chengdu, China; 4 Department of Surgical Intensive Care Unit, Sichuan Provincial People’s Hospital, University of Electronic Science and Technology of China, Chengdu, China; 5 Department of Gynecology and Obstetrics, Sichuan Provincial People’s Hospital, University of Electronic Science and Technology of China, Chengdu, China; 6 Department of Oncology, Sichuan Provincial People’s Hospital, University of Electronic Science and Technology of China, Chengdu, China; 7 Department of Operating Room Nursing, Sichuan Provincial People’s Hospital, University of Electronic Science and Technology of China, Chengdu, China; 8 Department of Emergency Medicine, Sichuan Provincial People’s Hospital, University of Electronic Science and Technology of China, Chengdu, China

**Keywords:** Critically ill patients, Malnutrition, Machine learning, Prediction model

## Abstract

**Background:**

Early detection of malnutrition in critically ill patients is crucial for timely intervention and improved clinical outcomes. However, identifying individuals at risk remains challenging due to the complexity and variability of patient conditions. This study aimed to develop and externally validate machine learning models for predicting malnutrition within 24 h of intensive care unit (ICU) admission, culminating in a web-based malnutrition prediction tool for clinical decision support.

**Methods:**

A total of 1006 critically ill adult patients (aged ≥ 18 years) were included in the model development group, and 300 adult patients comprised the external validation group. The development data were partitioned into training (80%) and testing (20%) sets. Hyperparameters were optimized via 5-fold cross-validation on the training set, eliminating the need for a separate validation set while ensuring internal validation. External validation was performed on an independent group to assess generalizability. Predictors were selected using random forest recursive feature elimination; seven machine learning models—Extreme Gradient Boosting (XGBoost), random forest, decision tree, support vector machine (SVM), Gaussian naive Bayes, k-nearest neighbor (k-NN), and logistic regression—were trained and evaluated for accuracy, precision, recall, F1 score, Area Under the Receiver Operating Characteristic Curve (AUC-ROC), Area Under the Precision-Recall Curve (AUC-PR). Model interpretability was analyzed using SHapley Additive exPlanations (SHAP) to quantify feature contributions.

**Results:**

In the development phase, among 1006 patients, 34.0% had moderate malnutrition and 17.9% severe malnutrition. The XGBoost model achieved superior predictive accuracy with an accuracy of 0.90 (95% CI = 0.86–0.94), precision of 0.92 (95% CI = 0.88–0.95), recall of 0.92 (95% CI = 0.89–0.95), F1 score of 0.92 (95% CI = 0.89–0.95), AUC-ROC of 0.98 (95% CI = 0.96–0.99), and AUC-PR of 0.97 (95% CI = 0.95–0.99) on the testing set. External validation confirmed robust performance with an accuracy of 0.75 (95% CI: 0.70–0.79), precision of 0.79 (95% CI: 0.75–0.83), recall of 0.75 (95% CI: 0.70–0.79), F1 score of 0.74 (95% CI: 0.69–0.78), AUC-ROC of 0.88 (95% CI: 0.86–0.91), and AUC-PR of 0.77 (95% CI: 0.73–0.80).

**Conclusions:**

Machine learning models, particularly XGBoost, demonstrated promising performance in early malnutrition prediction in ICU settings. The resultant web-based tool offers valuable resource for clinical decision support.

**Trial registration:**

Chinese Clinical Trial Registry ChiCTR2200058286 (https://www.chictr.org.cn/bin/project/edit? pid=248690). Registered 4th April 2022. Prospectively registered.

**Supplementary Information:**

The online version contains supplementary material available at 10.1186/s12911-025-03082-9.

## Background

Malnutrition, defined as an inadequate energy or nutrient status resulting from insufficient intake, increased metabolic demand, or impaired nutrient utilization, poses a significant threat to critically ill patients [1]. Critically ill patients with malnutrition often exhibit typical signs such as significant weight loss, cachexia, muscle wasting, reduced fat content, hypoalbuminemia, and edema [2, 3]. These manifestations not only reflect the severity of the underlying condition but also indicate the systemic impact of malnutrition on multiple organ systems. Malnutrition in critically ill patients can lead to a range of adverse outcomes, such as prolonged mechanical ventilation, impaired wound healing, increased complication rates, extended hospital stays, and elevated mortality rates [4–7]. According to a systematic review, the prevalence of malnutrition among critically ill patients ranges from 38 to 78% [8], highlighting the widespread nature of this issue in the intensive care setting. To mitigate the risks associated with malnutrition, international guidelines from the American Society for Parenteral and Enteral Nutrition (ASPEN) and the European Society for Clinical Nutrition and Metabolism (ESPEN) strongly advocate for early malnutrition assessment and diagnosis in critically ill populations [9, 10]. In alignment with these global standards, the Chinese Society for Parenteral and Enteral Nutrition (CSPEN) provides regionally adapted recommendations, emphasizing nutritional risk screening with biomarker thresholds tailored to Asian physiological profiles [11]. Early identification and intervention through these evidence-based protocols are critical for optimizing clinical outcomes and reducing the systemic burden of ICU malnutrition.

Accurately identifying the factors that lead to malnutrition in critically ill patients is the first step in predicting the occurrence of malnutrition. According to multiple literature reviews, the factors contributing to malnutrition primarily include disease severity, body mass index (BMI), weight loss, age, food intake, gastrointestinal injury, albumin and hemoglobin levels, muscle mass, and inflammatory markers such as C-reactive protein (CRP) [10–14]. While the majority of the studies have focused on predicting malnutrition among hospitalized cancer patients, research on critically ill patients in the intensive care unit (ICU) remains relatively limited [15–18]. In addition to the commonly associated factors, other specific conditions and treatments in the ICU may also further exacerbate the risk of malnutrition. These include mechanical ventilation, level of consciousness, the use of vasopressor drugs and sedatives, severe infection, and shock. The interplay between these factors and the underlying disease processes creates a complex and dynamic environment that significantly impacts nutritional status. Therefore, when predicting malnutrition in critically ill patients, it is crucial to consider not only nutritional indicators but also a wide range of factors related to patients’ circulatory, respiratory, and nervous system functions, as well as their treatment protocols. A comprehensive approach to screening for these relevant predictors is essential to ensure the accuracy and efficacy of malnutrition prediction in critically ill patients.

In recent years, machine learning (ML) prediction models have emerged as powerful tools for disease risk stratification and clinical decision support. These models have demonstrated success across diverse medical domains, including diabetes prognosis, cancer diagnosis, nutritional intervention planning, and drug discovery [19–22]. Compared to traditional statistical methods (e.g., logistic regression), ML algorithms excel at capturing intricate nonlinear relationships between multidimensional clinical variables and outcomes [23]. For instance, Wang et al. developed an optimized XGBoost model for early enteral nutrition assessment in ICU patients, achieving superior predictive performance (AUC-ROC: 0.904 vs. 0.884 for logistic regression) [24], which highlights ML’s potential to enhance precision in critical care nutrition management. While ML applications in malnutrition prediction have expanded to diverse populations—from neonatal cohorts (e.g., Yalçın et al. [25] predicting discharge weight in preterm infants) to pediatric cardiac surgery patients (e.g., Shi et al. [26] forecasting postoperative malnutrition)—these studies predominantly focus on non-adult populations. Critically, malnutrition drivers (e.g., growth velocity in neonates) and biomarker thresholds (e.g., age-adjusted z-scores) differ fundamentally from those in adult ICU patients, where inflammatory markers and chronic comorbidities play dominant roles.

Building on these advancements, this study aims to explore their applications in predicting malnutrition risk among critically ill adult patients. In addition to identifying key factors contributing to malnutrition, we developed predictive models and conducted both internal and external validation to identify the best-performing model. Furthermore, we created an interactive web tool using the best-performing model to assist clinicians in making informed malnutrition risk predictions for ICU patients.

## Methods

### Study design

This prospective observational study was undertaken at the Sichuan Provincial People’s Hospital in Chengdu, China. For the purposes of model development and internal validation, a sample comprising patients from the Emergency ICU (EICU), Surgical ICU (SICU), and Neurosurgical ICU (NICU) was selected for the period between March 2022 and December 2022. The external validation of the model utilized an additional sample from the Respiratory ICU (RICU) and Medical ICU (MICU) within the same hospital system, collected between January 2024 and November 2024. Participants were recruited based on the inclusion criteria of being aged 18 years or older. The exclusion criteria included pregnancy or breastfeeding, mental illness, a history of extracorporeal membrane oxygenation therapy or continuous renal replacement therapy, and death within 24 h of admission. Additionally, patients who withdrew from the study for various reasons were also excluded.

### Data collection

Patient information, which included sex, age, disease status (digestive, circulatory, respiratory, etc.), marital status, and surgical history, was obtained from the electronic medical records (EMR) system and compared against baseline characteristics. Additionally, scores such as the Acute Physiology and Chronic Health Evaluation II (APACHE II), Sequential Organ Failure Assessment (SOFA), Glasgow Coma Scale (GCS), and Nutrition Risk Screening 2002 (NRS 2002) were calculated for each patient.

Candidate predictors were screened through a comprehensive literature review of studies focusing on factors influencing malnutrition in critically ill patients. Seven databases (Web of Science, PubMed, Cochrane Library, China National Knowledge Infrastructure (CNKI), Chinese Medical Journal Full-text Database, SinoMed, and Wanfang Medical Network Chinese Journal Database) were searched from inception to March 10, 2022, using a combination of MeSH terms and free-text keywords. The PubMed search strategy exemplifies our approach:

(ICU [Title/Abstract]) OR (intensive care [Title/Abstract]) OR (critical care [Title/Abstract]) OR (critically ill patient* [Title/Abstract]) OR (critical illness [Title/Abstract]) OR (acute [Title/Abstract]) OR (emergency [Title/Abstract]) AND (malnutrition [Title/Abstract]) OR (malnourishment [Title/Abstract]) OR (undernutrition [Title/Abstract]) OR (nutritional deficiency* [Title/Abstract]) OR (nutrition [Title/Abstract]) AND (predict* model* [Title/Abstract]) OR (influence factor* [Title/Abstract]) OR (risk factor* [Title/Abstract]) OR (risk assessment [Title/Abstract]) NOT (pediatrics [Title/Abstract]) OR (child* [Title/Abstract]) OR (infant* [Title/Abstract]) AND (“1980/01/01” [Date - Publication]: “2022/03/10” [Date - Publication]).

In adherence to the TRIPOD guidelines for predictive model development, the inclusion criteria were strictly limited to cohort studies and cross-sectional designs focusing on adult critically ill patients (aged ≥ 18). The exclusion criteria encompassed conference papers, abstracts, case reports, inaccessible full-text articles, non-English/Chinese studies, and duplicates. It is considered that conference articles typically present preliminary findings, and case reports lack generalizability.

**Fig. 1:**
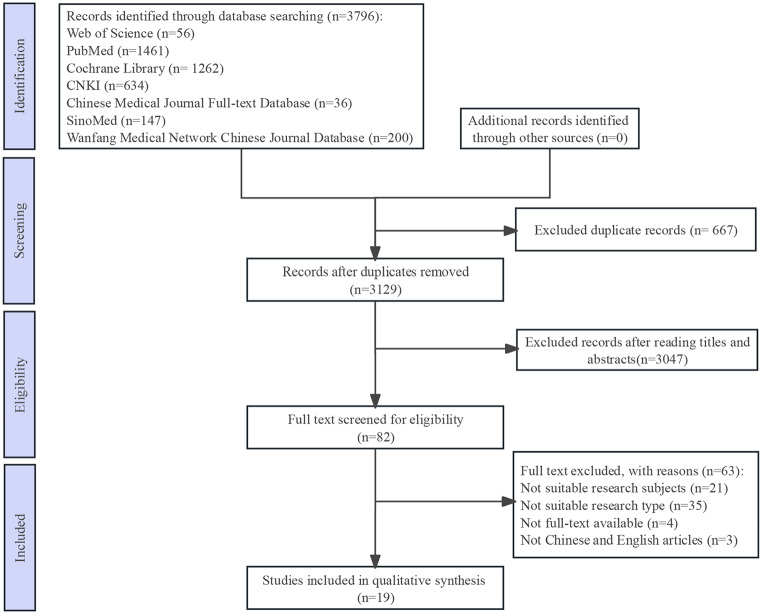
Flow chart of the study selection

Initially, 3,796 articles were retrieved and narrowed down to 19 articles after duplicate removal and initial and secondary screening [27–45]. The search process is illustrated in Fig. 1. The complete literature screening records have been provided as supplementary material (Additional File: Table S1), containing tracking details for all 82 candidate studies.

Furthermore, international and national nutrition guidelines—including those from the European Society for Clinical Nutrition and Metabolism (ESPEN), the American Society for Parenteral and Enteral Nutrition (ASPEN), and the Chinese Society for Parenteral and Enteral Nutrition (CSPEN)—published between 2006 and 2023 were systematically reviewed [9–11, 46, 47]. These consensus documents establish evidence-based frameworks for nutritional intervention strategies across diverse clinical populations, notably critical care, surgical, and medical patients. The synthesized evidence consistently identifies body mass index (BMI), serum albumin levels, and weight loss rate as pivotal biomarkers for malnutrition risk stratification in these guidelines.

Subsequently, we conducted an in-person expert consensus meeting involving ten specialists with substantial clinical and academic expertise in intensive care and nutritional medicine. The multidisciplinary panel consisted of six critical care physicians, two clinical nutritionists, and two researchers, collectively ensuring comprehensive clinical and methodological perspectives. All participants demonstrated at least ten years of frontline clinical experience in critical care or nutritional management, with selected experts (including professor Jiang and professor Xie) having authored five or more peer-reviewed publications in ICU nutrition or related domains during the preceding decade. Professor Jiang contribute to international nutritional guidelines (CSPEN) align with evidence-based standards (complete expert profiles provided in Additional File: Table S2). The session commenced with a systematic review of literature on nutritional assessment in critically ill populations, encompassing publications from database inception through 2022, with particular emphasis on prognostic factors strongly associated with clinical outcomes. Through rigorous evidence synthesis, we identified 50 candidate predictors spanning four domains: baseline patient characteristics (age, gender, marital status, APACHE II, SOFA scores), nutritional biomarkers (albumin, hemoglobin), inflammatory markers (leukocytes, neutrophils, C-reactive protein), and electrolyte/metabolic parameters.

The brainstorming session was divided into two orderly phases. In the first phase, experts engaged in heated discussions regarding the 43 initially screened predictive factors, comprehensively assessing their clinical relevance and practical operability. Moving into the second phase, experts actively proposed new predictive factors based on their extensive knowledge and complemented them with insights from existing literature and clinical experience. Ultimately, through rigorous voting and in-depth consensus, we successfully identified 39 candidate predictive factors (7 dichotomous, 32 continuous). Details of voting results are presented in Additional File: Table S3, and the details of these 39 predictors are presented in Table 1.

**Table 1:** Candidate predictors

Dichotomous variables	Continuous variables
Gender, mechanical ventilation, treatment with vasopressor drugs, treatment with sedatives, reduced energy intake, chronic gastrointestinal symptoms, acute gastrointestinal symptoms	Age, BMI, total protein, albumin, hemoglobin, red blood cell count, white blood cell count, neutrophil count, lymphocyte count, hematocrit, fasting blood glucose value, IL-6, procalcitonin, CD4^+^ T lymphocyte count, whole blood hs-CRP, PH value, PO2, oxygen saturation, sodium ions, potassium ions, magnesium ions, phosphorus ions, platelet count, serum creatinine, serum urea, serum uric acid, total bilirubin, body temperature, heart rate, respiratory rate, systolic blood pressure, diastolic blood pressure

Note: Abbreviations, BMI = Body Mass Index, IL-6 = interleukin-6, hs-CRP = high-sensitivity C-reactive protein, PO2 = arterial oxygen partial pressure. Reduced energy intake means intake ≤ 50% of requirement for > 1 week or < requirement for > 2 weeks

The study’s outcome variable was the severity of malnutrition among ICU patients, which was classified into three categories: no malnutrition, moderate malnutrition, and severe malnutrition. This classification was based on the Global Leadership Initiative on Malnutrition (GLIM) criteria [13]. The diagnostic process involved two main steps: (1) NRS 2002 screening ≥ 3 post -admission for nutritional risk [48]; (2) confirmation of malnutrition status through the presence of both phenotypic (weight loss thresholds, age-adjusted BMI criteria, and calf circumference measurements for muscle mass assessment) and etiologic (reduced food intake ≤ 50% of energy requirement > 1 week, or any reduction for > 2 weeks, C-reactive protein (CRP) level > 10 mg/L) indicators. Malnutrition severity was then categorized on the basis of the accumulation of phenotypic criteria.

All patient information, candidate malnutrition predictors, malnutrition diagnosis, and clinical scales (APACHE II, SOFA, GCS, and NRS 2002) were collected within 24 h of each patient’s admission by trained clinicians. Data on reduced energy intake, gastrointestinal symptoms, and BMI were obtained from the patients themselves, their legal surrogates (if applicable), or medical records. All data were verified for accuracy and completeness before inclusion in the analysis.

### Sample size calculation

To develop a stable model, we determined the minimum sample size using the methodology outlined by Riley et al. [49]. Based on a conservative C-statistic of 0.858 derived from published literature and an estimated malnutrition prevalence of 39.0% [18], the calculated minimum required sample size for model development was 837 cases. To accommodate a potential 25% loss due to invalid samples, we adjusted the final sample size to 1,047 cases for developing the model. The R code and detailed calculation procedures are provided in Additional File: Fig. S1.

For external validation of the best-performing model, the minimum required sample size was estimated empirically through statistical simulation [50]. This process aimed to ensure adequate power for detecting differences in model performance across various levels of malnutrition severity. We therefore included a total of 300 samples, stratified as follows: 100 with no malnutrition, 100 with moderate malnutrition, and 100 with severe malnutrition.

### Data preprocessing and feature selection

Spyder, a Python integrated development environment (IDE) designed for scientific computing and machine learning, was used to remove duplicates in the raw data based on patients’ unique hospital admission numbers, normalize features, discard noise and outliers, treat missing values. Variables with > 20% missingness were excluded, while those with ≤ 20% missingness were imputed using a random forest algorithm. The proportion of missing data for each candidate predictor is detailed in Additional File: Table S4. Additionally, binary variables were encoded with the LabelEncoder function, and continuous variables were standardized with the StandardScaler function. Details are provided in Additional File: Table S5.

To address the class imbalance in malnutrition severity categories (no/moderate/severe), we applied the Synthetic Minority Over-sampling Technique (SMOTE). This method generates synthetic samples for minority classes by interpolating between existing instances in the feature space, thereby balancing class distributions while preserving the original data structure.

Subsequent to class balancing, the random forest feature importance ranking and recursive feature elimination (RFE) methods were applied to perform feature selection among the 39 candidate predictors [51]. Feature selection was performed through backward elimination guided by random forest importance rankings: Starting with the full feature set, we sequentially removed the least important feature in each iteration while monitoring 5-fold cross-validation accuracy. The elimination process was terminated when removing an additional feature caused the validation AUC to drop by > 5% compared to the previous iteration, retaining the optimal feature subset prior to this performance degradation. This process determined the optimal feature subset for subsequent model construction.

### Model development and internal–external validation

We developed models using data from patients in the EICU, SICU, and NICU between March 2022 and December 2022. The best-performing model, identified during this development phase, was subsequently externally validated using data from patients in the RICU and MICU between January 2024 and November 2024.

During the model development phase, the development group was divided into an 80% training set and a 20% testing set. To address potential overfitting and optimize hyperparameters, we employed 5-fold cross-validation on the training set. This method iteratively partitioned the training data into subsets, using four folds for training and one fold for validation in each iteration, thereby eliminating the need for a separate validation set while ensuring rigorous parameter tuning. The testing set remained entirely unseen during this process to preserve its role in final performance evaluation. Seven classification algorithms—XGBoost, random forest, decision tree, support vector machine (SVM), Gaussian naive Bayes, K-nearest neighbor (KNN), and logistic regression—were trained. Hyperparameters were optimized via grid search with 5-fold cross-validation (scikit-learn’s GridSearchCV), with search ranges detailed in Table 2. Optimal hyperparameter combinations (Table 3) were subsequently used for model evaluation.

**Table 2:** Hyperparameter search ranges

Model	Hyperparameters	Search Range/Values
KNN	n_neighbors	[3, 5, 7]
SVM	C	[0.1, 1, 10]
	kernel	[‘linear’, ‘rbf’]
Decision Tree	max_depth	[None, 5, 10, 20]
Random Forest	n_estimators	[50, 100, 200]
Naive Bayes	-	-
Logistic Regression	C	[0.1, 1, 10]
XGBoost	n_estimators	[50, 100, 200]
	max_depth	[3, 5, 7, 9]
	learning_rate	[0.01, 0.1, 0.2]

**Table 3:** Optimal hyperparameter results

Model	Optimal hyperparameter combination
KNN	{‘n_neighbors’: 7}
SVM	{‘C’: 10, ‘kernel’: ‘linear’}
Decision Tree	{‘max_depth’: 5}
Random Forest	{‘n_estimators’: 100}
Naive Bayes	{}
Logistic Regression	{‘C’: 10}
XGBoost	{‘n_estimators’: 200, ‘max_depth’: 5, ‘learning_rate’: 0.1}
KNN	{‘n_neighbors’: 7}
SVM	{‘C’: 10, ‘kernel’: ‘linear’}
Decision Tree	{‘max_depth’: 5}

For both internal and external validation phases, we assessed model performance using accuracy, macro-precision, macro-recall, macro-F1 score, AUC-ROC, and Area Under the Precision-Recall Curve (AUC-PR). Macro-averaging was selected to ensure equal weighting of all malnutrition severity classes (no malnutrition, moderate malnutrition, severe malnutrition), regardless of class imbalance. This approach calculates metrics independently for each class and then averages them, thereby avoiding bias toward majority classes. This is particularly critical in clinical settings where accurate identification of all severity levels is equally important [52].

### Model explainability analysis

We employed SHapley Additive exPlanations (SHAP) technique to enhance clinical interpretability. We implemented the SHAP framework to quantify feature importance and visualize effect distributions. TreeExplainer was applied to our best model to calculate SHAP values for all samples.

### Statistical analysis

Statistical analyses were conducted using SPSS Statistics 29.0 (IBM, USA) for the general clinical data. For continuous variables, normality was initially assessed using the Kolmogorov–Smirnov test. Normally distributed variables were presented as means ± standard deviations, with Student’s t-test used for comparisons between two groups. Conversely, non-normally distributed variables were summarized using medians (interquartile ranges [IQR]), with the nonparametric Mann–Whitney *U* test selected for intergroup comparisons. Categorical variables were described in terms of case numbers and percentages (rates), with chi-square tests used for comparisons between two groups.

### Transparent reporting of a multivariable prediction model for individual prognosis or Diagnosis + Artificial intelligence (TRIPOD + AI) statement

This research strictly adhered to the TRIPOD + AI statement and comprehensively reported the title, abstract, background, methods, open science, patient involvement, results and discussion to ensure the transparency, accuracy, and reproducibility of the research [53].

## Results

### Participant characteristics

**Fig. 2:**
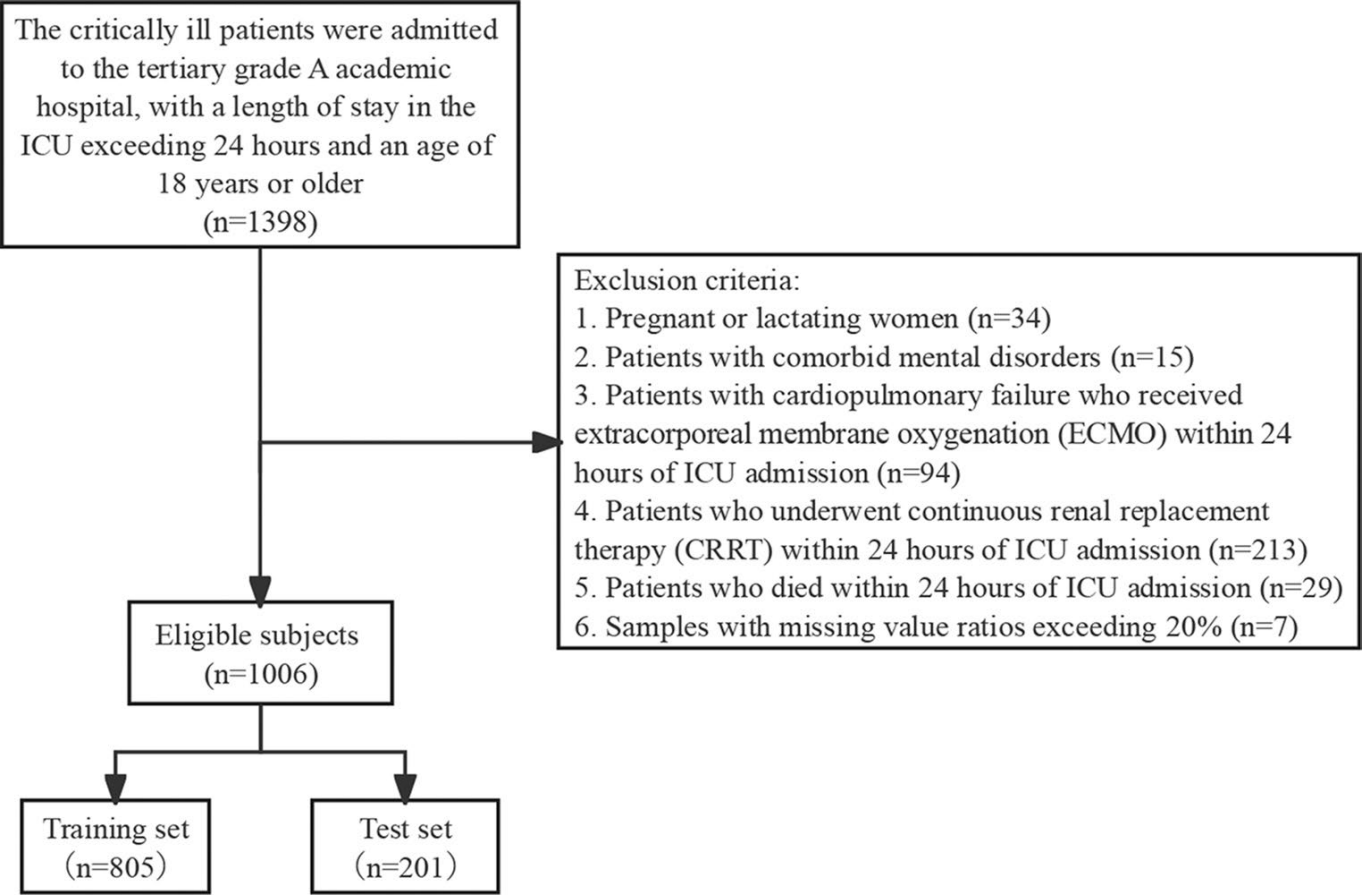
Patient screening flowchart

During the model development phase, 1,398 patients were initially included, of which 1,006 patients were ultimately enrolled in the study after applying the exclusion criteria. During the external validation phase, an additional 300 patients was included, stratified as follows: 100 with no malnutrition, 100 with moderate malnutrition, and 100 with severe malnutrition. Among the 1,006 patients in the development group, 94.1% were screened for nutritional risk using the NRS 2002 scale. A total of 51.9% were diagnosed with malnutrition based on the GLIM criteria, including 34.0% with moderate malnutrition and 17.9% with severe malnutrition. Statistically significant differences (*P* < 0.01) were observed between non-malnourished and malnourished patient groups regarding age, BMI, marital status, disease type, reduced energy intake, chronic gastrointestinal symptoms, percentage weight loss, APACHE II score, and NRS 2002 score. Detailed information on the patient screening process is provided in Fig. 2, and participant characteristics are summarized in Table 4.

**Table 4:** Demographic and clinical characteristics of the participants

Variables	All cases (*n* = 1006)	Non-malnourished (*n* = 484)	Malnourished (*n* = 522)	Z/χ²	*P*-value
Age (years), n(IQR)	64(52, 74)	57(48, 68)	68(56, 78)	-8.896	< 0.001
Sex, male, n (%)	616(61.2)	289(59.7)	327(62.6)	0.910	0.340
BMI, kg/m2	21.5(19.6, 23.7)	23.3(21.7, 25.7)	19.6(18.3, 21.2)	-19.787	< 0.001
Marital status, n (%) Married Unmarried Divorced Widowed	974(96.8) 27(2.7) 3(0.3) 2(0.2)	463(95.7) 21(4.3) 0(0) 0(0)	511(97.9) 6(1.1) 3(0.6) 2(0.4)	-3.705	< 0.001
Disease type, n (%) Digestive system Circulatory system Respiratory system Neurological system Others	287(28.5) 265(26.3) 232(23.1) 154(15.3) 68(6.8)	120(24.8) 177(36.6) 97(20.0) 52(10.7) 38(7.9)	167(32.0) 88(16.9) 135(25.9) 102(19.5) 30(5.7)	59.636	< 0.001
Mechanical ventilation, n (%)	506(50.3)	236(48.8)	270(51.7)	0.882	0.348
Treatment with vasopressor drugs, n (%)	425(42.2)	199(41.1)	226(43.3)	0.489	0.484
Treatment with sedatives, n (%)	472(46.9)	219(45.2)	253(48.5)	1.045	0.307
Reduced energy intake, n (%)	276(27.4)	52(10.7)	224(42.9)	130.537	< 0.001
Chronic gastrointestinal symptoms, n (%)	201(20.0)	41(8.5)	160(30.7)	77.278	< 0.001
Acute gastrointestinal symptoms, n (%)	309(30.7) b	144(29.8)	165(31.6)	0.407	0.523
Unintentional weight loss of ≥ 5% within 6 months, n (%)	234(23.3)	4(0.8)	230(44.1)	262.996	< 0.001
Unintentional weight loss of ≥ 10% within 6 months, n (%)	77(7.7)	1(0.2)	76(14.6)	73.195	< 0.001
Surgical history, n (%)	457(45.4)	207(42.8)	250(47.9)	2.660	0.103
GCS score ≤ 8 points, n (%)	402(40.0)	182(37.6)	220(42.1)	2.160	0.142
SOFA score ≥ 3 points, n (%)	869(86.4)	410(84.7)	459(87.9)	2.214	0.137
APACHE II score ≥ 10 points, n (%)	921(91.6)	429(88.6)	492(94.3)	10.242	0.001
NRS 2002 score ≥ 3 points, n (%)	947(94.1)	429(88.6)	522(100)	62.749	< 0.001

Note: Abbreviations, IQR = Interquartile Range, BMI = Body Mass Index, GCS = Glasgow Coma Scale, SOFA = Sequential Organ Failure Assessment, APACHE = Acute Physiology and Chronic Health Evaluation, NRS = Nutrition Risk Screening

Table 5 provides information on the laboratory test results. Compared with the non-malnourished group, the malnourished group presented lower levels of albumin, hemoglobin, red blood cell count, hematocrit, serum uric acid, fasting blood glucose, sodium ions, and magnesium ions (*P* < 0.01). Conversely, the malnourished group had a higher platelet count (*P* < 0.01). The results revealed statistically significant differences in the levels of these parameters between the malnourished and non-malnourished groups.

**Table 5:** Laboratory test results

Laboratory test	All cases (*n* = 1006)	Non-malnourished (*n* = 484)	Malnourished	Z	*P*
Total protein	56.55(50.20, 62.43)	56.50(51.20, 61.90)	56.75(48.50, 63.05)	-0.588	0.557
Albumin	31.30(27.00, 36.20)	33.45(28.13, 36.78)	30.45(26.18, 35.33)	-4.698	< 0.001
Hemoglobin	111.00(93.00, 127.00)	114.00(96.00, 130.00)	106.00(89.75, 123.25)	-3.806	< 0.001
Red blood cell count	3.75(3.19, 4.30)	3.84(3.32, 4.36)	3.65(3.09, 4.20)	-3.358	< 0.001
White blood cell count	11.19(7.83, 15.79)	11.32(7.93, 15.68)	10.93(7.74, 15.91)	-0.261	0.794
Neutrophil count	9.66(6.39, 13.57)	9.73(6.60, 13.43)	9.57(6.27, 13.79)	-0.229	0.819
Lymphocyte count	0.71(0.43, 1.18)	0.74(0.44, 1.22)	0.69(0.42, 1.14)	-0.879	0.379
Hematocrit	34.40(28.80, 39.40)	35.30(30.03, 39.98)	33.20(28.30, 38.53)	-3.378	< 0.001
Fasting blood glucose value	8.63(6.62, 11.68)	9.01(6.93, 12.84)	8.33(6.34, 11.02)	-3.774	< 0.001
Interleukin-6	121.14(62.93, 299.28)	178.68(35.59, 449.55)	204.15(32.70, 399.34)	-1.295	0.195
Procalcitonin	0.71(0.17, 3.86)	0.71(0.14, 3.98)	0.76(0.19, 3.82)	-0.497	0.619
CD4^+^ T lymphocyte count	192.00(122.00, 288.00)	245.00(107.00, 296.00)	258.00(129.00, 297.00)	-0.480	0.632
Whole blood hs - CRP	37.44(5.94, 114.42)	31.71(4.12, 123.15)	42.39(9.40, 109.52)	-1.671	0.095
Platelet count	158.00(107.75, 221.25)	150.00(99.00, 205.75)	165.00(113.75, 233.25)	-3.310	< 0.001
PO_2_	115.50(81.00, 158.30)	111.80(81.80, 156.80)	119.65(79.50, 161.48)	-0.769	0.442
Serum urea	7.07(4.95, 10.83)	6.99(5.03, 10.38)	7.21(4.91, 11.11)	-0.375	0.708
Serum creatinine	76.60(57.70, 116.90)	77.00(59.85, 120.38)	74.90(56.38, 112.23)	-1.903	0.057
Serum uric acid	304.00(216.00, 418.25)	319.50(237.00, 429.75)	292.50(200.00, 406.25)	-3.115	0.002
Total bilirubin	16.90(10.88, 26.83)	17.35(11.10, 27.08)	16.45(10.70, 26.35)	-0.923	0.356
PH value	7.38(7.32, 7.45)	7.38(7.32, 7.44)	7.38(7.32, 7.45)	-0.316	0.752
Sodium ions	139.20(135.60, 143.03)	140.00(136.13, 143.78)	138.80(135.10, 142.03)	-3.299	< 0.001
Potassium ions	4.03(3.65, 4.47)	4.04(3.64, 4.50)	4.00(3.66, 4.43)	-0.913	0.361
Magnesium ions	0.87(0.76, 1.04)	0.89(0.78, 1.11)	0.85(0.75, 0.99)	-4.078	< 0.001
Phosphorus ions	1.07(0.80, 1.37)	1.05(0.80, 1.37)	1.09(0.80, 1.37)	-0.391	0.696

Note: Abbreviations, hs - CRP = high - sensitivity C - Reactive Protein, PO_2_ = Arterial Oxygen Partial Pressure

### Important factors associated with malnutrition

**Fig. 3:**
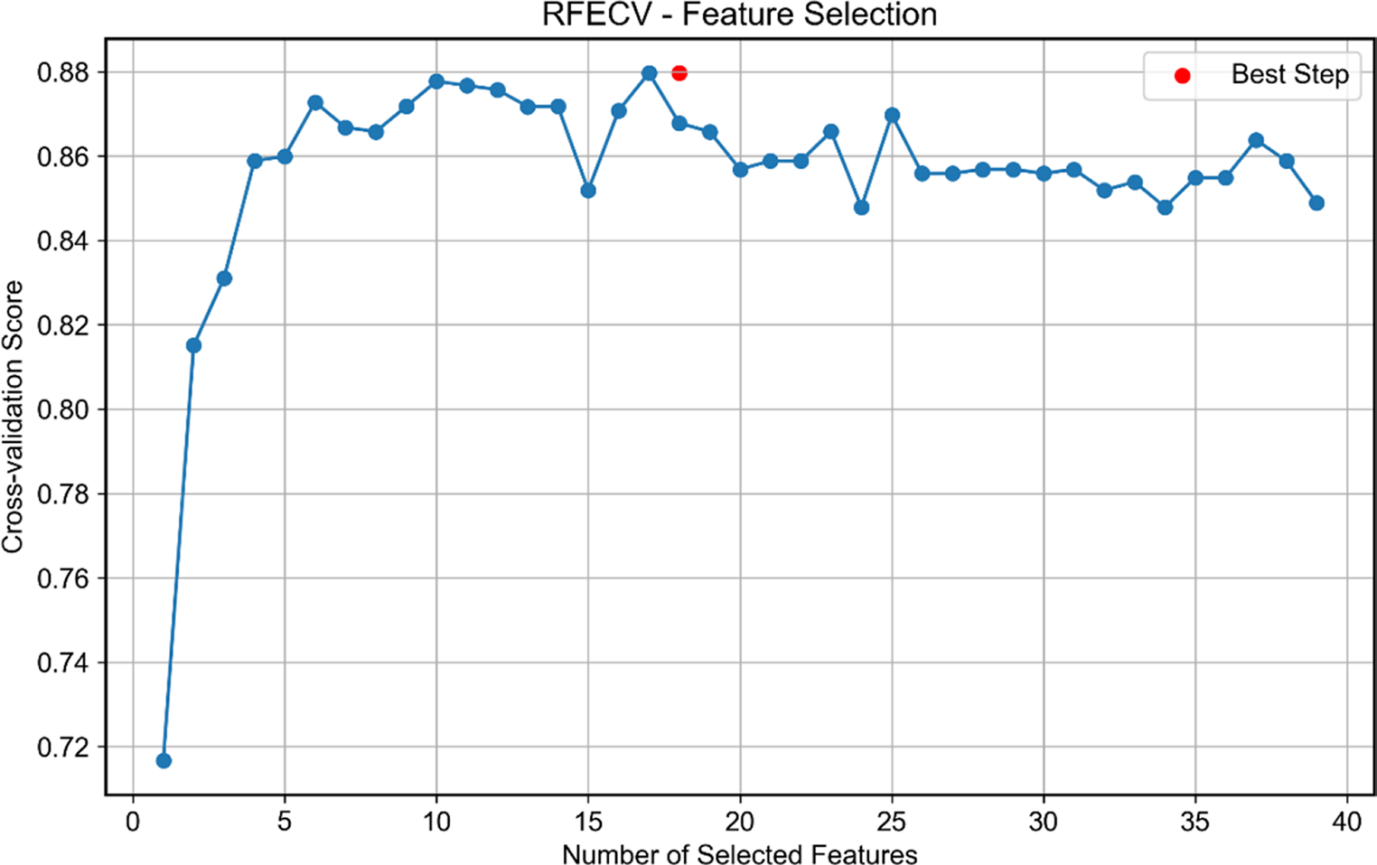
RFECV-Feature selection

**Fig. 4:**
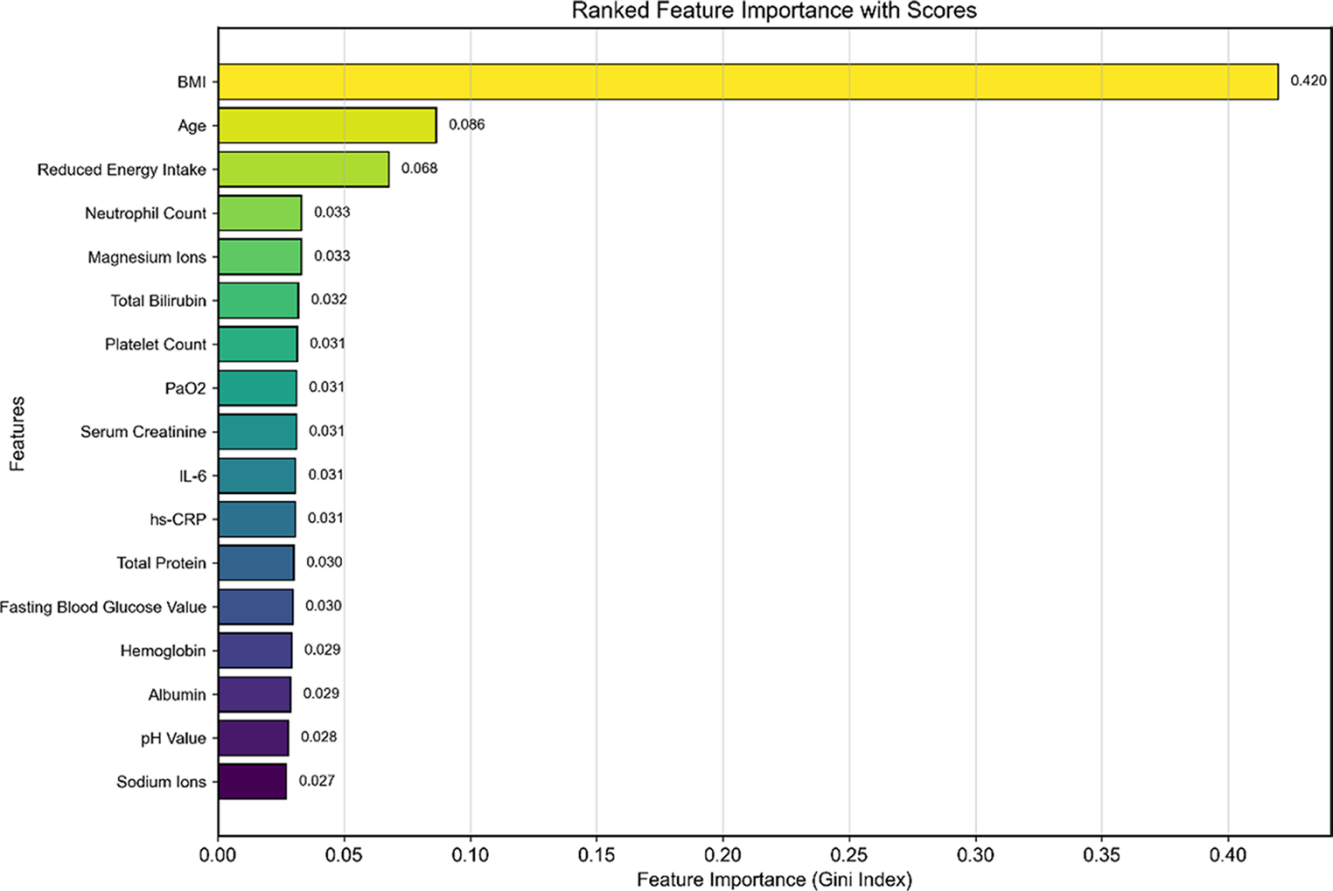
Ranked feature importance with scores

Using the random forest recursive feature elimination method, seventeen important factors associated with malnutrition were identified and ranked in descending order of importance, as detailed in Figs. 3 and 4.

### Model performance

**Fig. 5:**
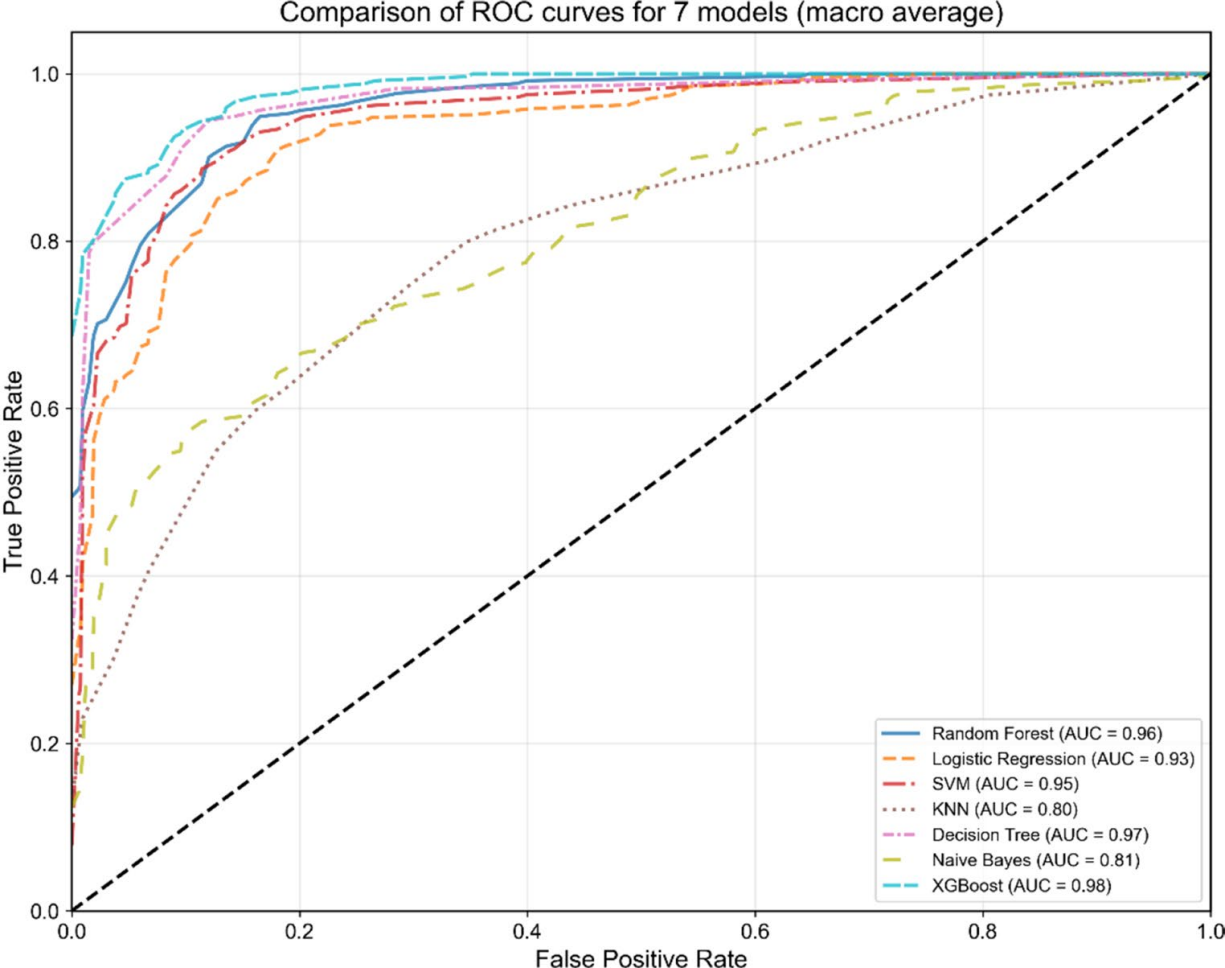
ROC curves

**Fig. 6:**
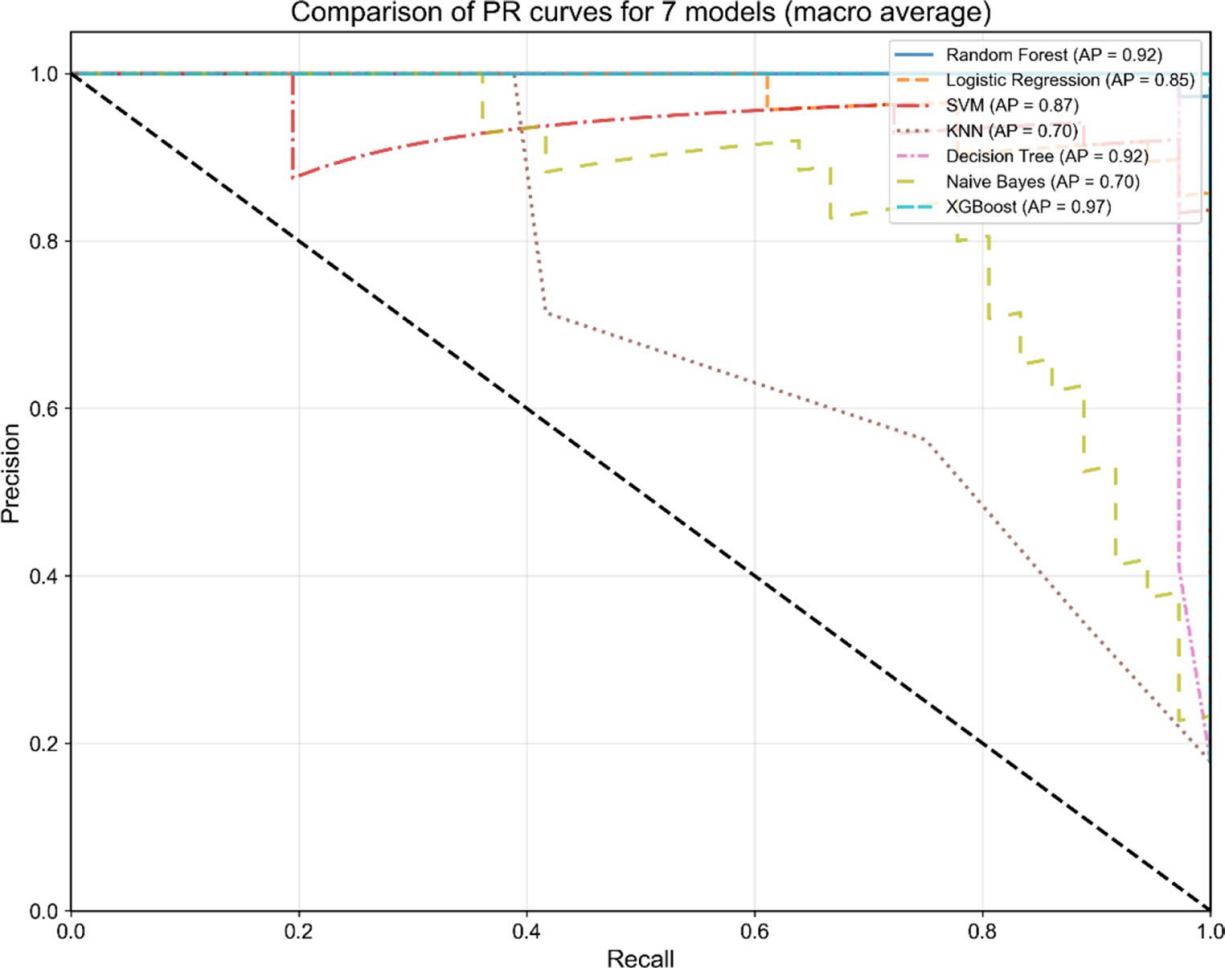
PR curves

**Fig. 7:**
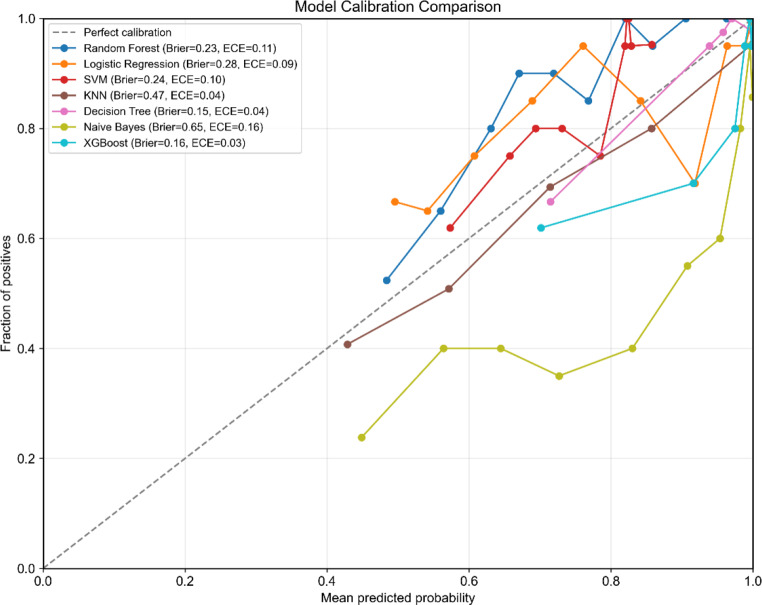
Model calibration comparison

In the model development phase, Table 6 compares the performance of seven models on the testing set using optimal hyperparameters. Notably, the XGBoost model outperformed the other models in terms of predictive accuracy, achieving an accuracy of 0.90 (95% CI = 0.86–0.94), precision of 0.92 (95% CI = 0.88–0.95), recall of 0.92 (95% CI = 0.89–0.95), F1 score of 0.92 (95% CI = 0.89–0.95), AUC-ROC of 0.98 (95% CI = 0.96–0.99), and AUC-PR of 0.97 (95% CI = 0.95–0.99). The decision tree algorithm demonstrated a close second place with an accuracy of 0.91 (95% CI = 0.87–0.95), precision of 0.93 (95% CI = 0.90–0.96), recall of 0.93 (95% CI = 0.89–0.96), F1 score of 0.92 (95% CI = 0.89–0.96), AUC-ROC of 0.97 (95% CI = 0.94–0.99), and AUC-PR of 0.92 (95% CI = 0.86–0.98), whereas the logistic regression algorithm was positioned fifth among the evaluated models. Figures 5 and 6 visually represent the distributions of AUC‒ROC and AUC‒PR for these models, highlighting the consistency of their performance across different thresholds. To assess the reliability of the predicted probabilities, calibration plots were constructed to examine the alignment between predicted probabilities and observed frequencies. Figure 7 presents the calibration curves for the seven predictive models, with corresponding Brier scores (BS) and expected calibration errors (ECE) quantifying the divergence between predicted probabilities and observed outcomes. Notably, the XGBoost model demonstrated superior calibration performance on the test set, attaining the lowest BS (0.16) and ECE (0.03) among all classifiers. These results indicate that the predicted probabilities generated by the XGBoost classifier exhibit well-calibrated reliability, suggesting strong potential for clinical risk stratification applications.

In the external validation phase, the XGBoost model demonstrated robust performance on the external validation set with an accuracy of 0.75 (95% CI: 0.70–0.79), precision of 0.79 (95% CI: 0.75–0.83), recall of 0.75 (95% CI: 0.70–0.79), F1 score of 0.74 (95% CI: 0.69–0.78), AUC-ROC of 0.88 (95% CI: 0.86–0.91), and AUC-PR of 0.77 (95% CI: 0.73–0.80). The model also exhibited lower Brier score of 0.43 and ECE values of 0.15, indicating well-calibrated predicted probabilities. Detailed results are shown in Additional File: Fig. S2-S4.

**Table 6:** Predictive performance of the 7 models on the testing set under the optimal hyperparameters

Metrics Models	Accuracy (95% CI)	Precision (95% CI)	Recall (95% CI)	F1 (95% CI)	AUC-ROC (95% CI)	AUC-PR (95% CI)	Brier Score	ECE
XGBboost	0.90 (0.86, 0.94)	0.92 (0.88, 0.95)	0.92 (0.89, 0.95)	0.92 (0.89, 0.95)	0.98 (0.96, 0.99)	0.97 (0.95, 0.99)	0.16	0.03
Random Forest	0.86 (0.80, 0.90)	0.87 (0.83, 0.92)	0.87 (0.83, 0.92)	0.87 (0.83, 0.92)	0.96 (0.93, 0.98)	0.92 (0.89, 0.96)	0.23	0.11
Decision Tree	0.91 (0.87, 0.95)	0.93 (0.90, 0.96)	0.93 (0.89, 0.96)	0.92 (0.89, 0.96)	0.97 (0.94, 0.99)	0.92 (0.86, 0.98)	0.15	0.04
SVM	0.86 (0.81, 0.90)	0.86 (0.82, 0.91)	0.87 (0.82, 0.91)	0.87 (0.82, 0.91)	0.94 (0.91, 0.97)	0.87 (0.81, 0.93)	9.24	0.10
Gaussian Naive Bayes	0.55 (0.50, 0.60)	0.54 (0.48, 0.60)	0.60 (0.55, 0.64)	0.50 (0.45, 0.55)	0.81 (0.77, 0.85)	0.70 (0.65, 0.76)	0.65	0.16
KNN	0.64 (0.58, 0.70)	0.71 (0.64, 0.77)	0.58 (0.52, 0.65)	0.61 (0.53, 0.68)	0.80 (0.75, 0.84)	0.70 (0.64, 0.77)	0.47	0.04
Logistic Regression	0.83 (0.78, 0.88)	0.84 (0.79, 0.89)	0.84 (0.79, 0.89)	0.84 (0.79, 0.89)	0.93 (0.90, 0.96)	0.86 (0.81, 0.91)	0.28	0.09

Note: Macro-averaged metrics (precision, recall, F1) were computed by averaging class-specific values with equal weight, ensuring balanced evaluation across all malnutrition severity classes (no/moderate/severe malnutrition). This approach is recommended for imbalanced multi-class clinical prediction tasks [49]

### Model explainability with SHAP

We employed SHAP analysis to interpret model decisions across global, class-specific, and individual levels. The summary plot (Additional File: Fig. S5) identifies BMI, age, and reduced energy intake as primary risk drivers (positive SHAP values), contrasting with albumin’s protective role (negative values), with color gradients indicating feature magnitude (red = high, blue = low). Class-stratified bar plots (Additional File: Fig. S6) confirm BMI’s dominance while revealing critical variations: serum creatinine/bilirubin drive Class 2 risk, whereas albumin/magnesium most impact Class 0. Extending these insights, partial dependence plots (Additional File: Fig. S7) demonstrate sodium’s U-shaped risk relationship in Class 2 (elevated risk at both extremes) versus Class 0’s inverse sodium-risk association. Finally, a patient-level waterfall plot (Additional File: Fig. S8) quantifies prediction decomposition: reduced energy intake (+ 2.59) and age (+ 1.73) elevate risk, partially offset by BMI’s protective effect (-0.24).

### Online prediction tool

**Fig. 8:**
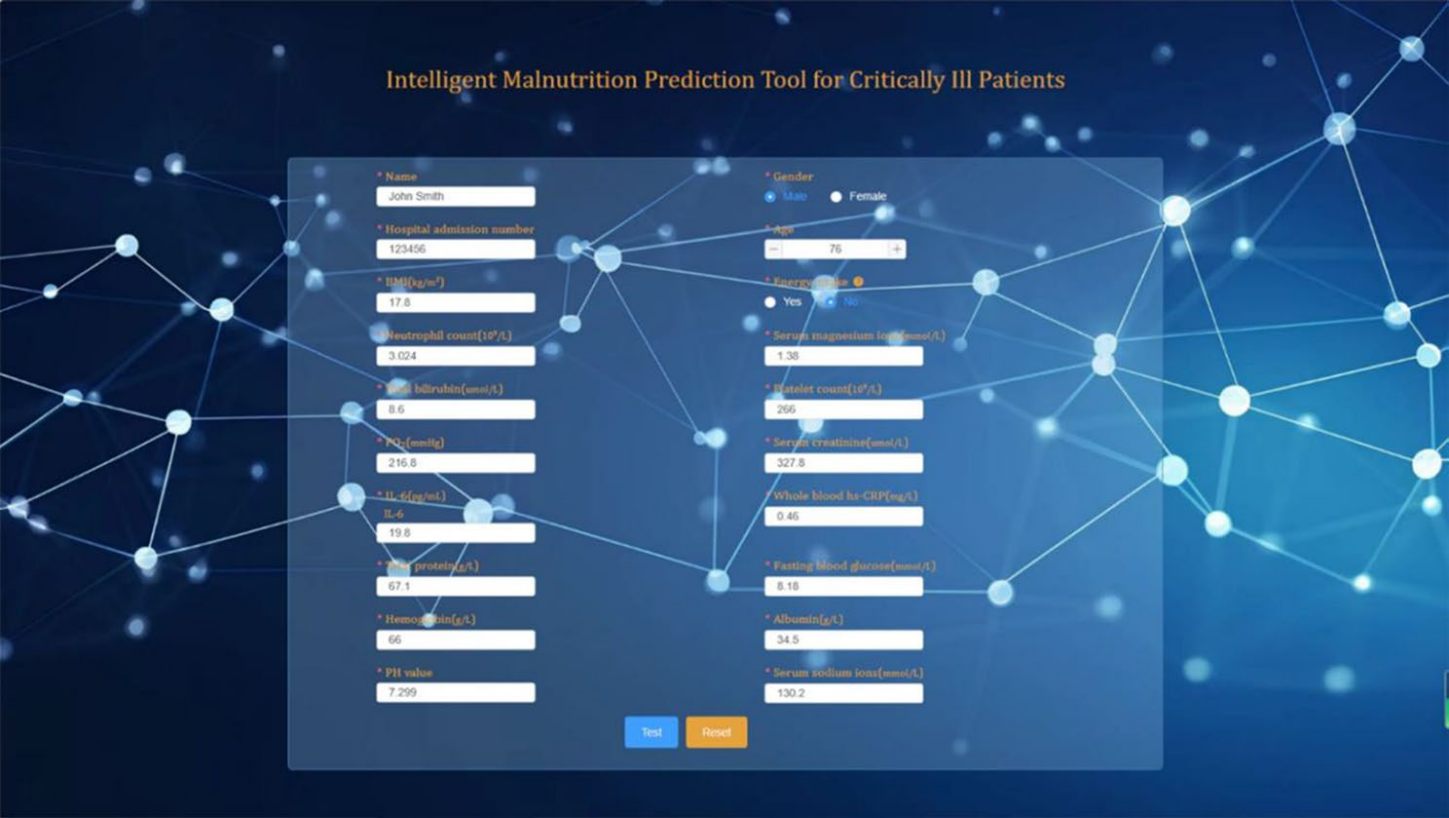
Intelligent malnutrition prediction tool for critically ill patients

**Fig. 9:**
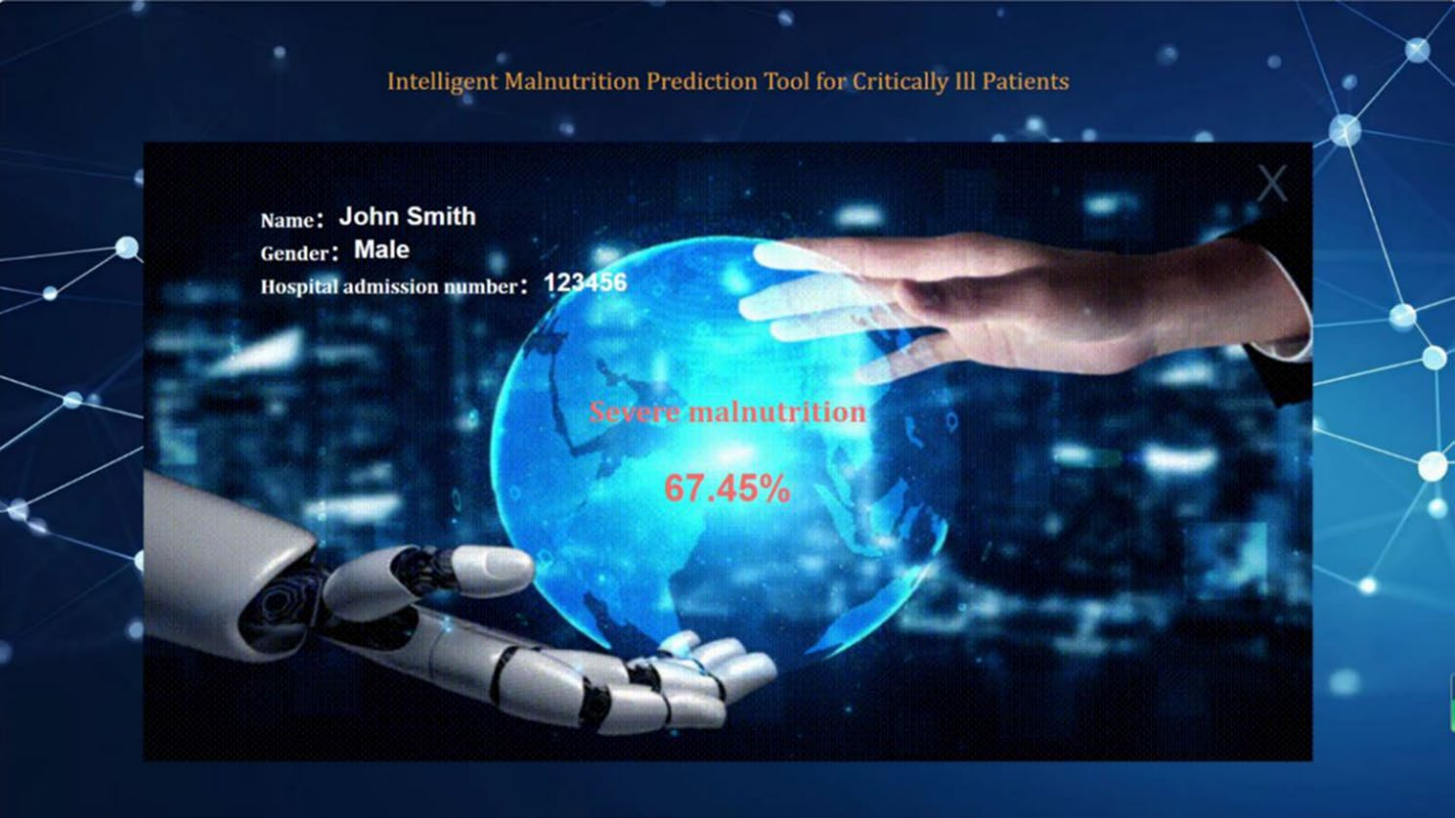
Presentation of prediction results

Ultimately, we successfully developed a web-based malnutrition prediction tool specifically designed for critically ill patients, utilizing the optimized XGBoost model. The tool operates under a privacy-by-design framework: all computations are performed locally within the user’s browser, and no patient data is stored or transmitted to external servers, ensuring compliance with GDPR and HIPAA standards. It is freely accessible online at http://www.malnutrition.top/ without requiring registration or login. the key features include: Missing mandatory fields (e.g., albumin, BMI) trigger real-time alerts via red highlighting, and predictions are disabled until all required data are provided (Figs. 8 and 9). The modular backend architecture allows seamless integration of updated prediction models (e.g., new biomarkers or algorithm versions), while the frontend code is openly hosted on GitHub (DOI:10.5281/zenodo.15575937) to support community-driven interface enhancements. Clinicians may deploy the tool locally using the archived open-source code, guaranteeing accessibility even if the web service is discontinued.

## Discussion

In the present study, we found that 51.9% of critically ill patients met the criteria for malnutrition as defined by the GLIM criteria, which is a substantial proportion and highlights the widespread nature of this issue in critical care settings. This prevalence aligns with the variability observed in previous research, where Theilla et al. [54] documented a lower incidence rate of 41.0%, while Milanez et al. [55] reported a higher prevalence of 65.5%. These discrepancies may be attributed to differences in patient populations, assessment methodologies, or the specific GLIM criteria applied. Furthermore, our results are consistent with the broad range of 15–68% identified by Díaz et al. [56] in their systematic review and meta-analysis, underscoring the complexity and heterogeneity of malnutrition incidence among critically ill patients.

Using machine learning algorithms to investigate primary malnutrition factors in critically ill patients, our study identified significant biomarkers, including BMI; age; energy intake; neutrophil and platelet counts; magnesium and sodium ions; total bilirubin; PO_2;_ and serum creatinine, IL-6, hs-CRP, total protein, fasting glucose, hemoglobin, and albumin levels. Notably, BMI, age, and reduced energy intake emerged as the top three factors, with statistically significant differences (*P* < 0.01) between malnourished and non-malnourished patients, emphasizing their critical importance in nutritional assessments. Consistent with Yin et al.‘s findings [17], the importance of BMI is reinforced, indicating its pivotal role in predicting malnutrition. We also found that advancing age, especially ≥ 65 years, increases malnutrition risk, exacerbated by physiological decline, chronic diseases, and the resulting malnutrition cycle. Additionally, inadequate nutrient intake is crucial, with 84% of patients at risk due to underestimated energy requirements, preexisting gastrointestinal conditions, acute illnesses, surgeries, and restricted food intake, contributing to increased mortality [57]. Consequently, clinicians should prioritize accurate BMI, age, and food intake assessments; optimize nutritional strategies to mitigate malnutrition risk; and improve outcomes in this vulnerable population.

While the identified biomarkers are strongly correlated with malnutrition, they may also reflect patients’ underlying inflammatory states or disease severity. For example, IL-6 and hs-CRP are well-known markers of systemic inflammation that can alter metabolic processes and nutrient utilization. Elevated bilirubin and creatinine levels, as well as changes in platelet count, PO_2_, and pH, might indicate organ dysfunction or severe illness. Electrolyte imbalances, such as those involving magnesium and sodium ions, can affect nutrient absorption and overall nutritional status. Therefore, while these biomarkers are important for predicting malnutrition, clinicians should interpret them within the context of patients’ overall health and potential pathological processes. Specifically regarding IL-6, its role as a predictor warrants nuanced interpretation. While IL-6 demonstrated predictive value in our model, its specificity to malnutrition versus systemic inflammation requires clarification. Elevated IL-6 levels may reflect acute inflammatory responses rather than direct nutritional depletion, particularly in critically ill patients with infections or trauma. This dual nature underscores the importance of contextual clinical correlation when utilizing IL-6 in malnutrition risk algorithms.

Given the inherent limitations of conventional assessment tools, which are prone to subjective patient perceptions and evaluator biases, the utilization of ML algorithms, as demonstrated in our study, represents a significant advancement. These algorithms circumvent the aforementioned shortcomings, presenting a more impartial and rigorous evaluation framework. The comprehensive suite of 17 factors identified, including BMI, age, reduced energy intake, and a diverse array of biomarkers, offers a nuanced and accurate portrayal of malnutrition severity in critically ill patients. This nuanced understanding, grounded in robust evidence, fortifies clinical decision-making processes. Consequently, we urge clinicians to incorporate these physiological and biochemical indicators into their malnutrition risk prediction and assessment practices, adopting a holistic and integrated approach that enhances the precision of malnutrition diagnosis in this vulnerable patient population.

In this study, we developed and validated seven ML models for early malnutrition prediction in critically ill patients. Notably, the XGBoost model achieved superior predictive performance (AUC: 0.98) for malnutrition risk stratification in ICU patients, outperforming traditional logistic regression (AUC: 0.93). Consistent with Yalçın et al. [58], who employed ML to predict oral feeding transitions in general adult inpatients (AUC: 0.770), our study achieved enhanced predictive accuracy by prioritizing ICU-specific malnutrition biomarkers, including body mass index (BMI), serum albumin levels, and weight loss rate—variables explicitly linked to nutritional deterioration in critical illness. This performance divergence reinforces the necessity of context-driven model development, as malnutrition drivers in ICU patients (e.g., metabolic stress, prolonged immobilization) fundamentally differ from those in general wards.

According to established standards in clinical prediction modeling, an AUC-ROC threshold of ≥ 0.70 is widely regarded as the minimum benchmark for clinical utility [59]. Our model significantly exceeded this criterion, with an AUC-ROC of 0.98 in the development cohort and 0.88 in the external validation cohort, indicating excellent discriminatory ability. Furthermore, the Brier scores (0.16 for development; 0.43 for validation) demonstrated strong calibration, with values approaching 0 reflecting high precision in probabilistic predictions. These results collectively suggest that the model not only meets but surpasses conventional acceptability thresholds, positioning it as a reliable decision-support tool for early malnutrition risk stratification in critical care settings. While external validation demonstrated robust performance (AUC-ROC 0.88), the validation cohort originated from ICUs within the same hospital system. While this confirms generalizability across specialized units under consistent protocols, broader validation across geographically diverse institutions with varying patient demographics and care standards remains essential to establish universal applicability.

Additionally, we developed a novel malnutrition prediction tool for critically ill patients via an interactive online interface with the XGBoost algorithm. This tool outperforms traditional models, facilitating seamless integration with clinical systems for automated data extraction and analysis. It may may help reduce potential misdiagnoses (e.g., false negatives in severe malnutrition cases) and could streamline clinical workflows by automating risk stratification. Furthermore, the tool’s high prediction accuracy effectively mitigates misdiagnoses and omissions due to memory bias or inexperience among clinicians, especially junior staff, ensuring timely and accurate evaluation of a patient’s nutritional status. In essence, this tool enhances clinical nutrition assessment efficiency, accuracy, and decision-making, exhibiting substantial clinical and research value.

The main strength of this study lies in the use of objective methods to accurately predict malnutrition severity in ICU patients. We utilized a range of anthropometric, nutritional, and biochemical indicators, including IL-6, which has significant clinical relevance and predictive ability within our model’s specific context. Leveraging ML, specifically XGBoost, significantly enhanced the prediction accuracy. However, several limitations should be acknowledged. First, potential sample bias arises from the single-center design at Sichuan Provincial People’s Hospital, limiting generalizability to regions with differing ICU practices. Second, our reliance on 24-hour static measurements, while clinically pragmatic, may not capture dynamic nutritional changes during prolonged ICU stays. Serial biomarker monitoring could enhance temporal sensitivity but requires validation in future studies. Third, while the use of IL-6 as a predictive factor contributes to the model’s predictive value, its specificity to malnutrition versus systemic inflammation requires careful interpretation in clinical contexts. Fourth, although external validation was performed in different ICUs within the same hospital system, the shared institutional protocols, homogeneous patient demographics, and consistent clinical practices may limit the generalizability of the model to external healthcare settings with distinct operational standards or patient populations. Fifth, the current external validation cohort (*n* = 300) precludes subgroup analyses (e.g., age, sex, or comorbidity-specific performance evaluation). As emphasized by TRIPOD + AI guidelines [53], a minimum of 100 cases per subgroup is required for statistically reliable fairness validation—a threshold not met in our study due to sample size constraints. Additionally, our decision to exclude conference articles and case reports from the literature review may have resulted in a more conservative estimate of certain features’ predictive power.

Beyond these methodological constraints, implementation challenges warrant attention. Self-reported parameters (e.g., BMI, energy intake) may introduce recall bias, necessitating objective verification via body composition analysis. While XGBoost demonstrated superior performance, its “black-box” nature may hinder clinical trust; future work should explore interpretable AI architectures. Practical barriers include electronic health record (EHR) integration and regulatory compliance, requiring systematic evaluation. Finally, our literature review’s exclusion criteria may undervalue predictive features explored in non-ICU cohorts. These limitations necessitate cautious interpretation and further validation in diverse populations. Future studies should: (1) Conduct multicenter collaborations with larger cohorts to enable subgroup validation and geographic generalizability; (2) Explore dynamic biomarker monitoring; (3) Investigate implementation frameworks for EHR deployment; (4) Reassess excluded literature for novel predictive insights.

## Conclusions

In this study, we observed a high incidence of malnutrition among critically ill patients, warranting close attention from clinicians. With the aim of developing a clinical decision-support tool for malnutrition, we developed and validated seven machine learning models for early prediction in these patients, with the XGBoost model demonstrating superior predictive performance. In addition, we developed an easy-to-use interactive tool based on the XGBoost model that visualizes predictions and offers clinicians a convenient and accurate means of clinical decision support.

## Electronic supplementary material

Below is the link to the electronic supplementary material.

Supplementary Material 1

Supplementary Material 2

Supplementary Material 3

Supplementary Material 4

Supplementary Material 5

Supplementary Material 6

Supplementary Material 7

Supplementary Material 8

Supplementary Material 9

Supplementary Material 10

Supplementary Material 11

Supplementary Material 12

Supplementary Material 13

Supplementary Material 14

Supplementary Material 15

Supplementary Material 16

Supplementary Material 17

Supplementary Material 18

Supplementary Material 19

Supplementary Material 20

Supplementary Material 21

Supplementary Material 22

Supplementary Material 23

Supplementary Material 24

Supplementary Material 25

## Data Availability

Data is provided within the supplementary information files.
