# Supplementary figures and images for: Development and external validation of machine learning models for the early prediction of malnutrition in critically ill patients: a prospective observational study
Source: BMC Med Inform Decis Mak. 2025 Jul 3;25:248. doi: 10.1186/s12911-025-03082-9 (PMC12225150; doi:10.1186/s12911-025-03082-9)

XGBoost Multiclass ROC Curve

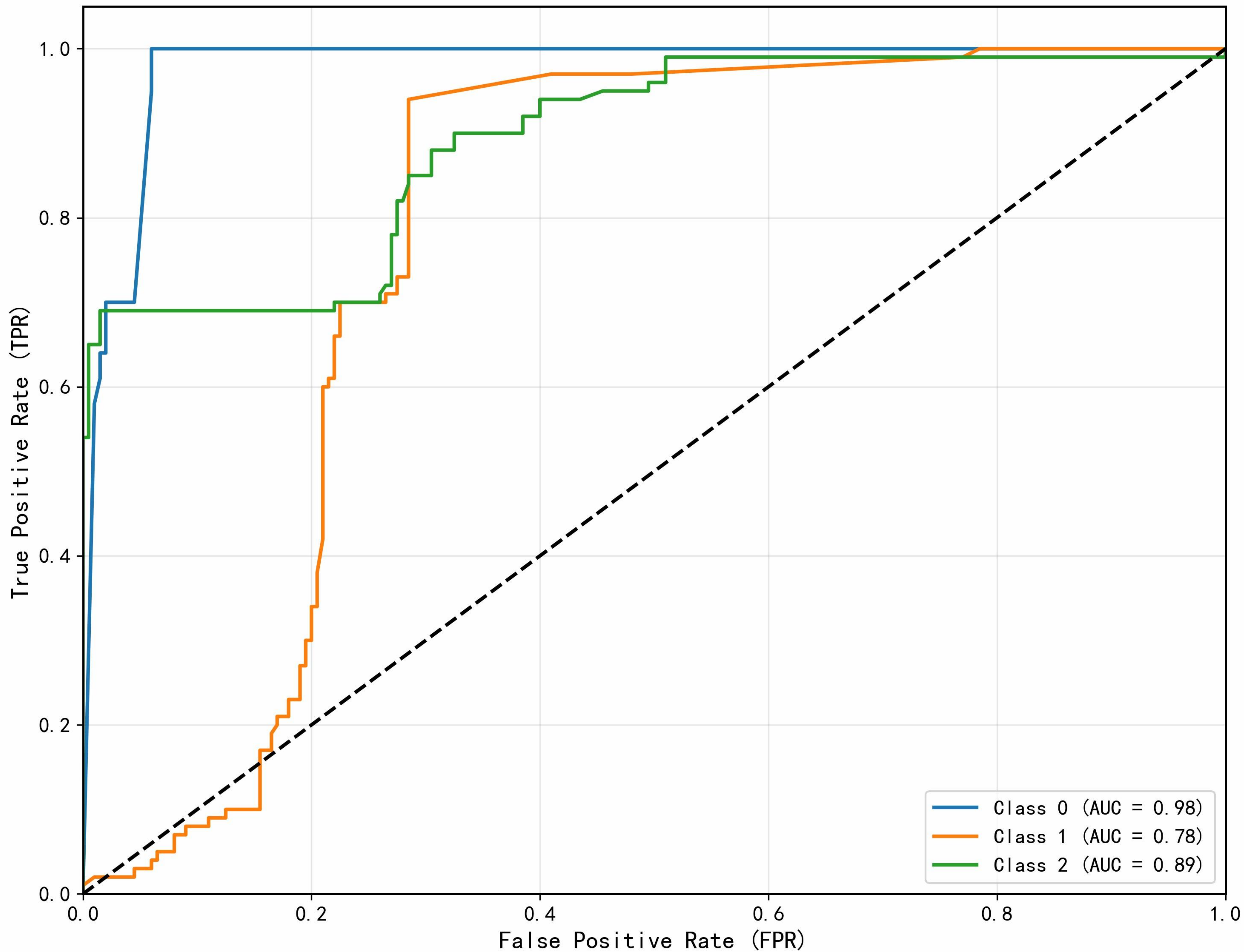

Supplement: Supplementary file 1 — Supplementary Material 1 [file 12911_2025_3082_MOESM1_ESM.pdf]

XGBoost Multiclass PR Curve

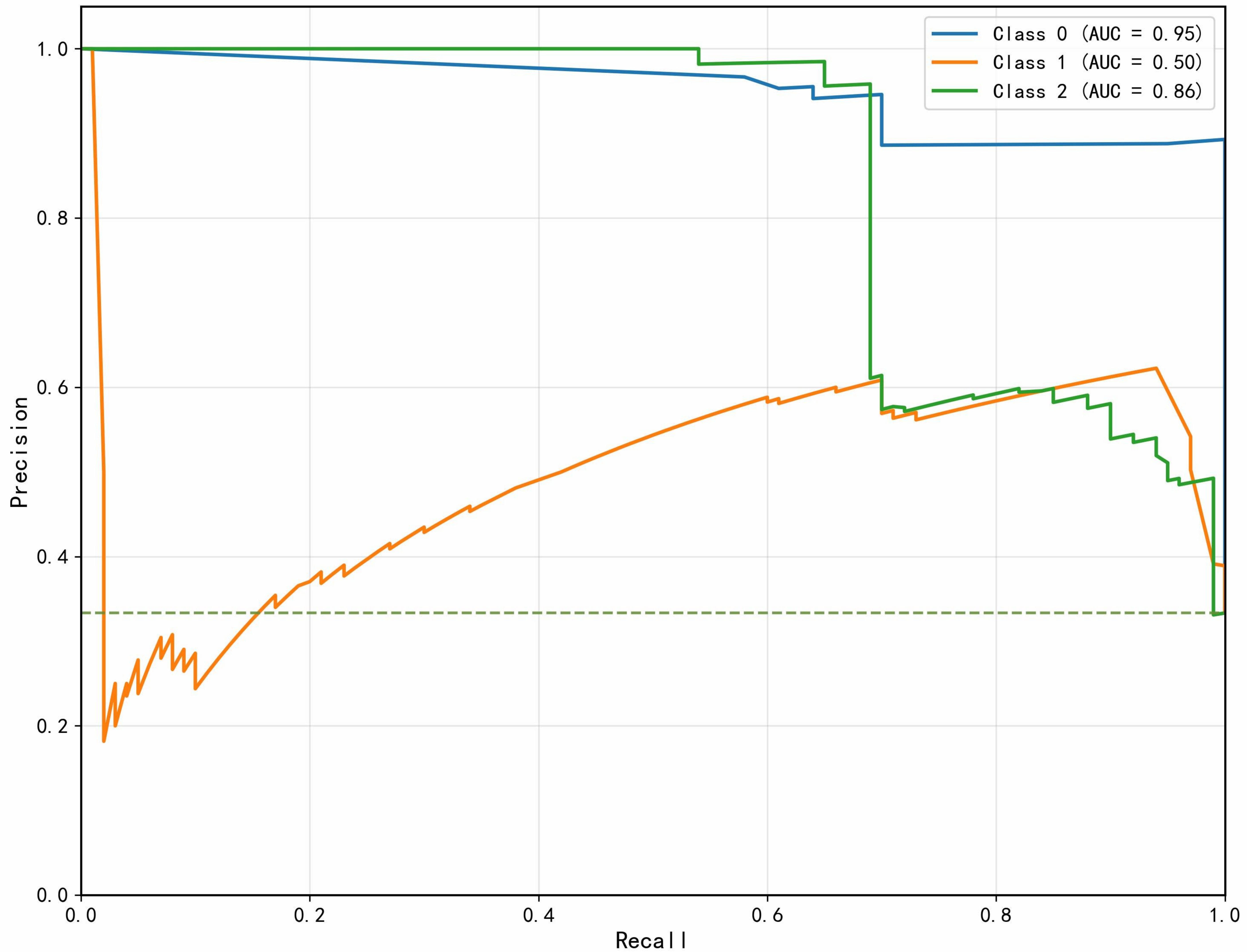

Supplement: Supplementary file 2 — Supplementary Material 2 [file 12911_2025_3082_MOESM2_ESM.pdf]

# XGBoost Calibration Curve

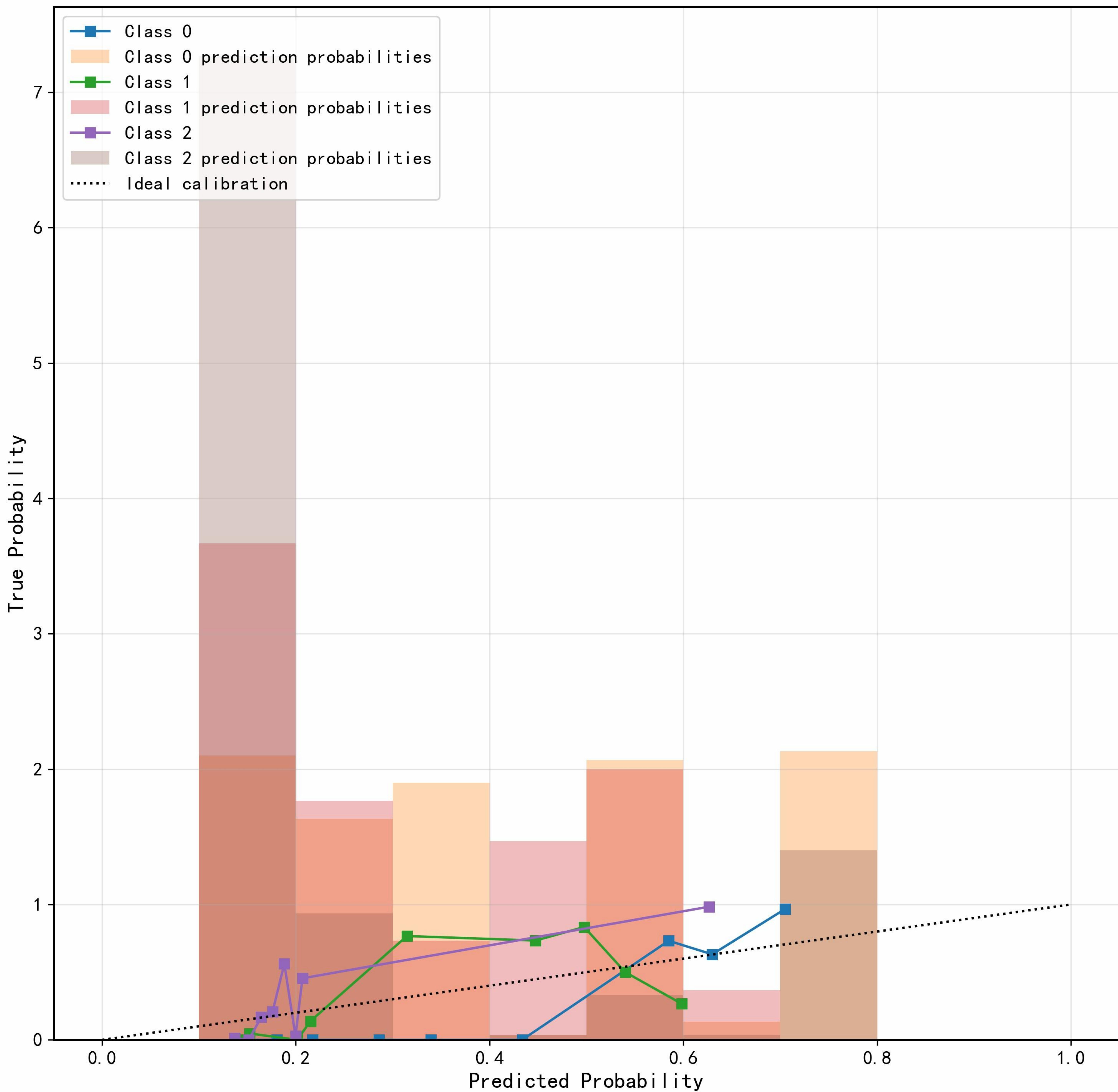

Supplement: Supplementary file 3 — Supplementary Material 3 [file 12911_2025_3082_MOESM3_ESM.pdf]

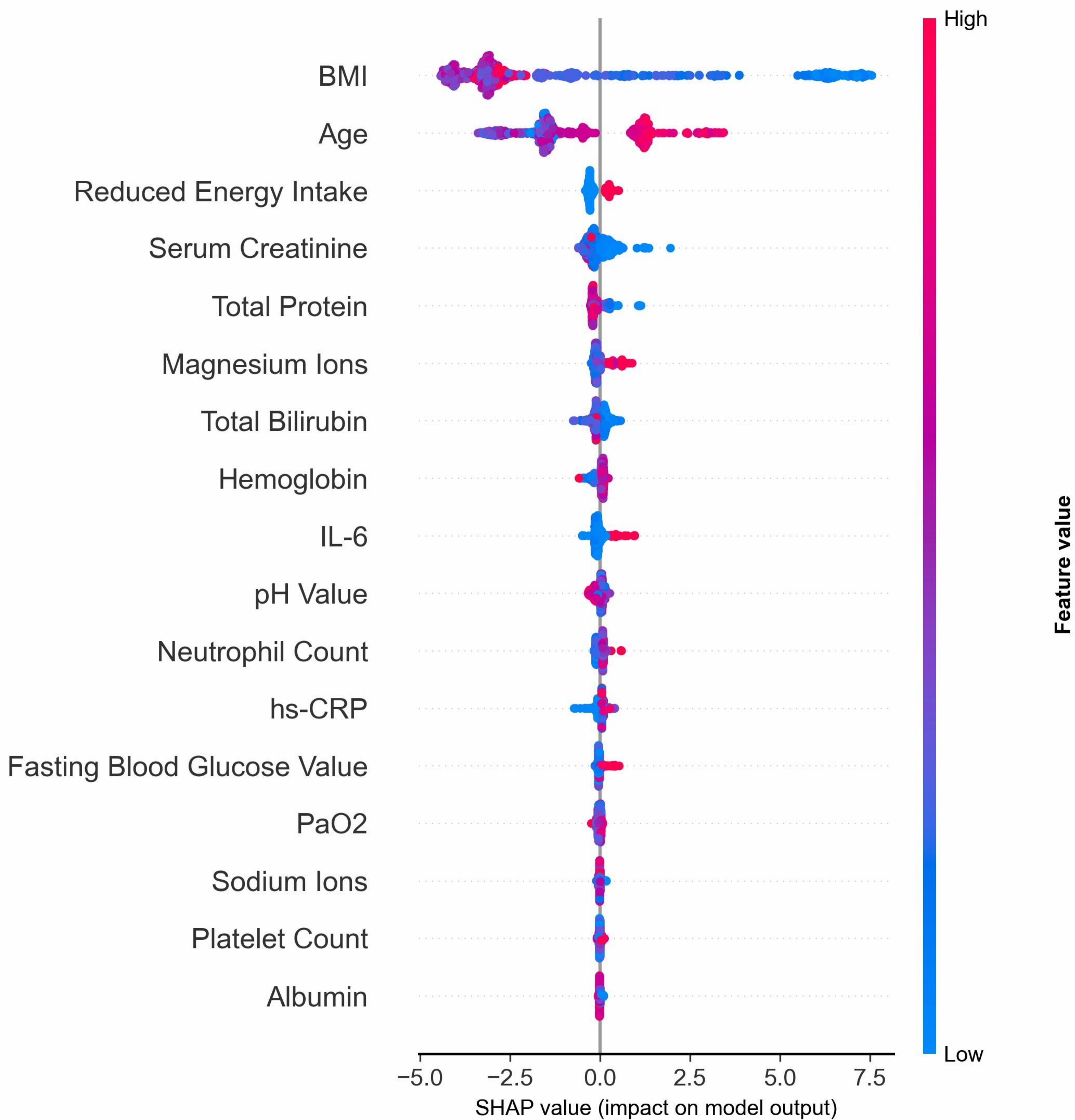

Supplement: Supplementary file 4 — Supplementary Material 4 [file 12911_2025_3082_MOESM4_ESM.pdf]

SHAP Feature Importance (Bar Plot)

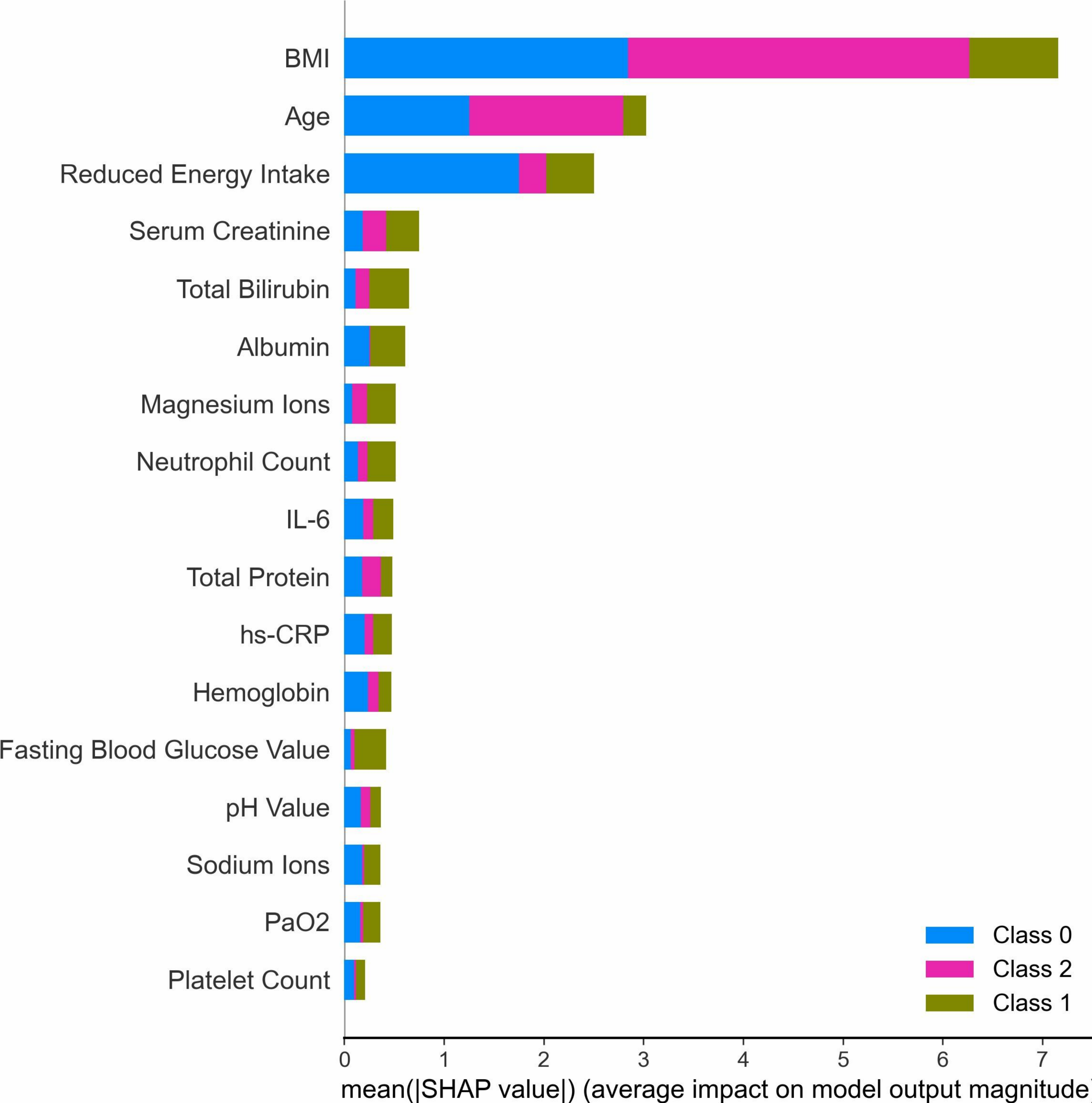

Supplement: Supplementary file 5 — Supplementary Material 5 [file 12911_2025_3082_MOESM5_ESM.pdf]

## Partial Dependence Plots for Top Features by Class

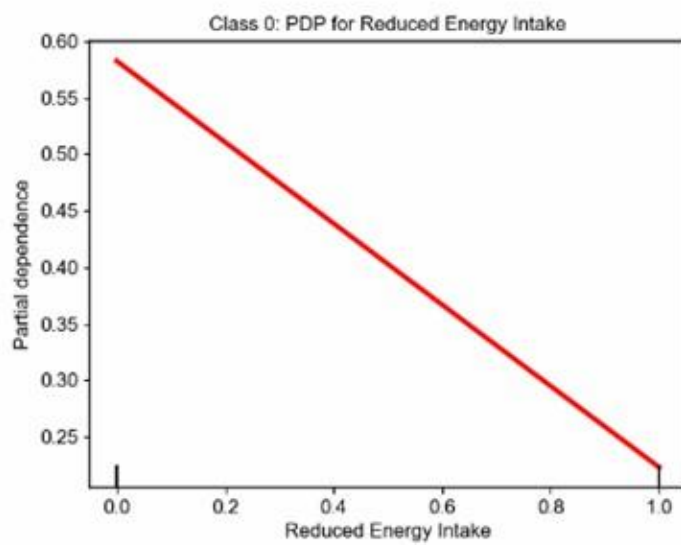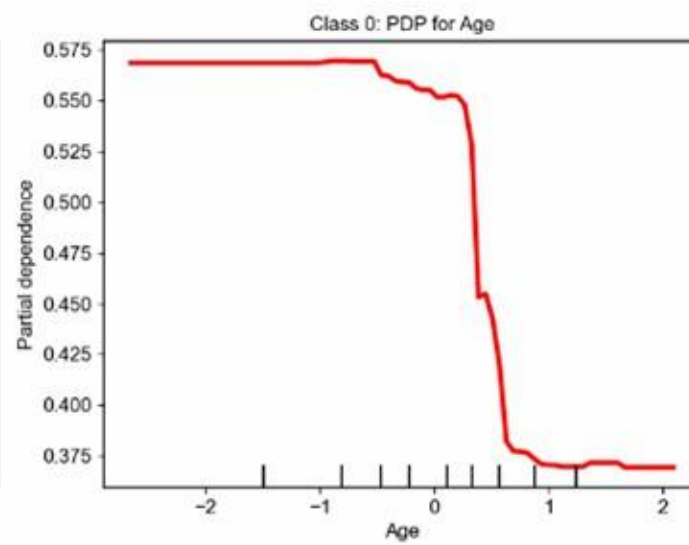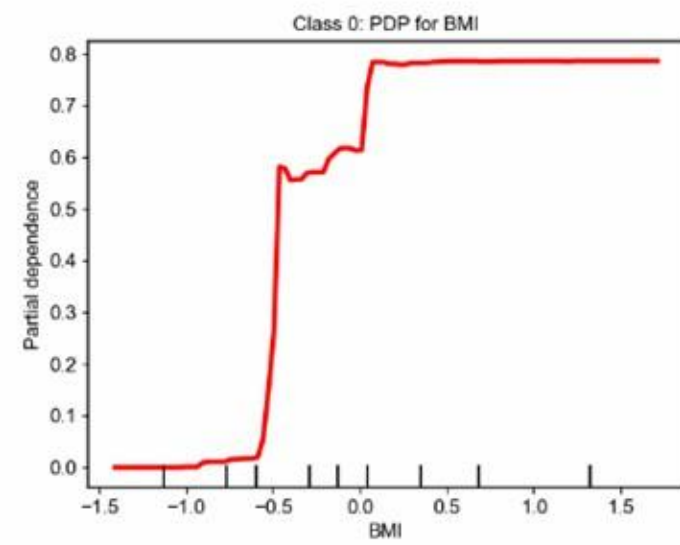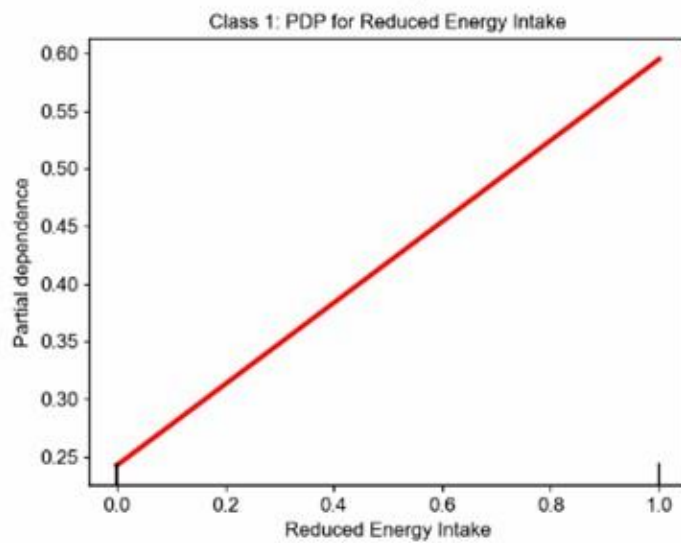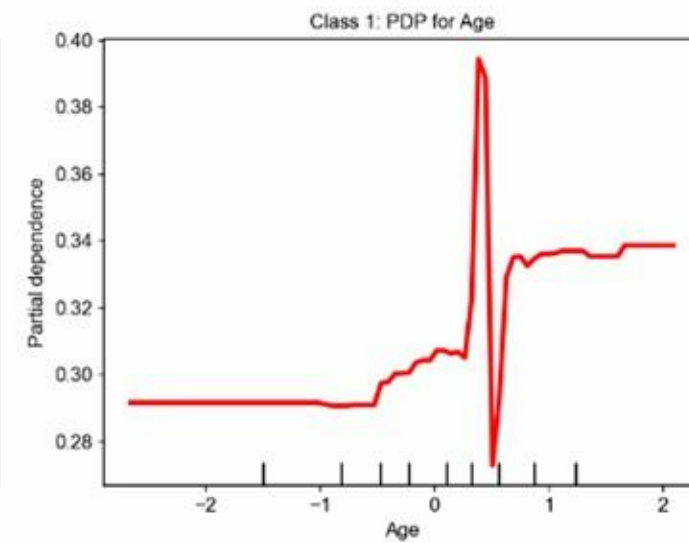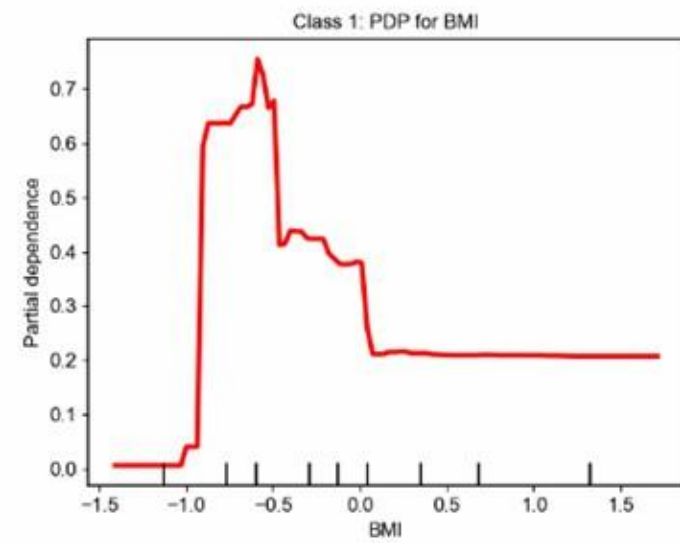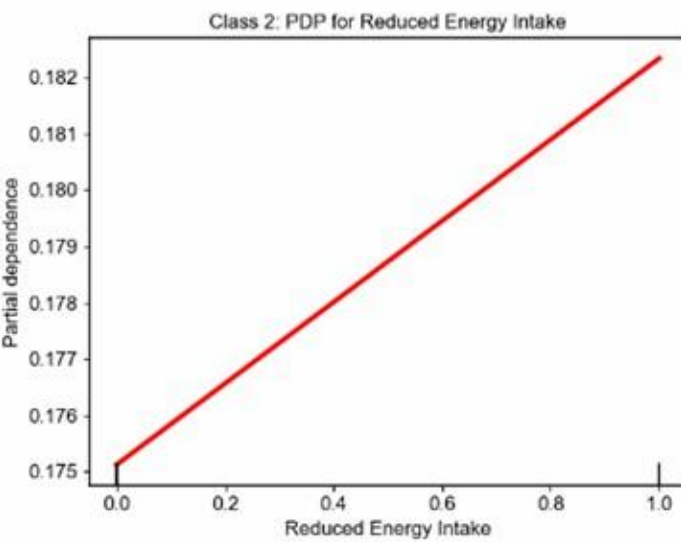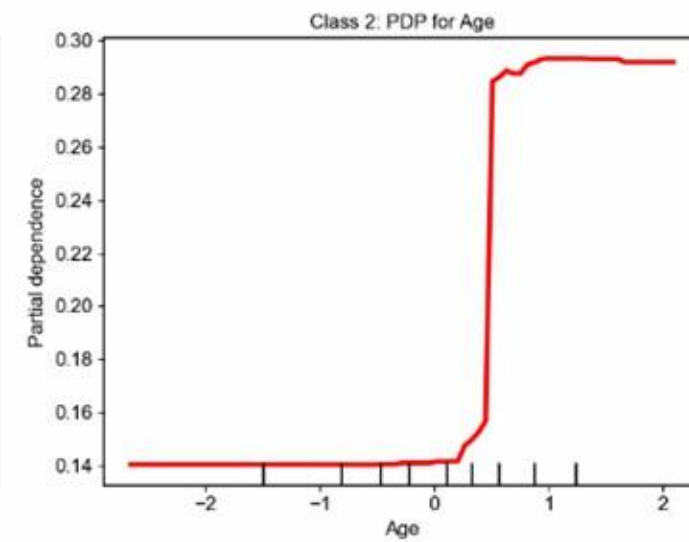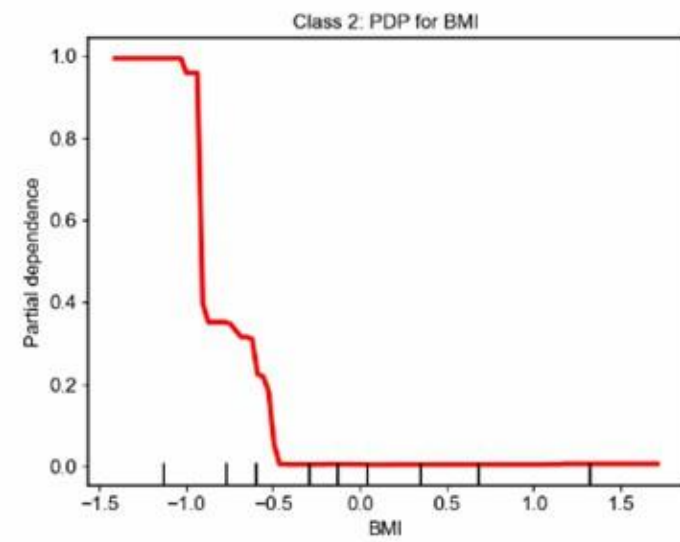

Supplement: Supplementary file 6 — Supplementary Material 6 [file 12911_2025_3082_MOESM6_ESM.pdf]

SHAP Waterfall Plot for Sample 0 (Class 1)

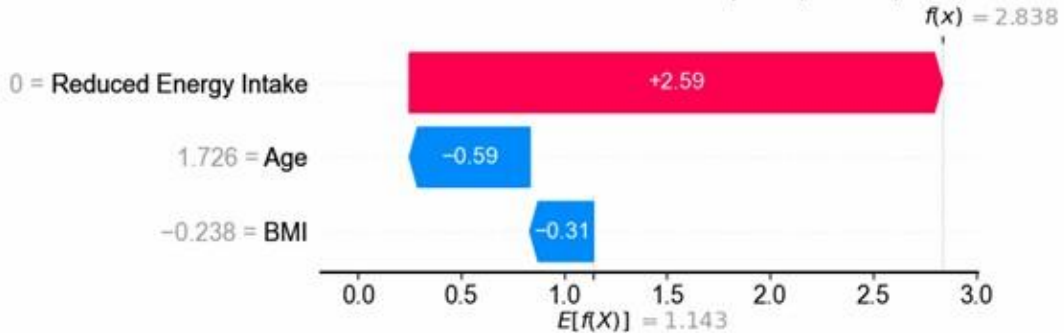

Supplement: Supplementary file 7 — Supplementary Material 7 [file 12911_2025_3082_MOESM7_ESM.pdf]
