# Supplementary material for: Development and external validation of machine learning models for the early prediction of malnutrition in critically ill patients: a prospective observational study
Source: BMC Med Inform Decis Mak. 2025 Jul 3;25:248. doi: 10.1186/s12911-025-03082-9 (PMC12225150; doi:10.1186/s12911-025-03082-9)
Supplement: Supplementary file 8 — Supplementary Material 8 [file 12911_2025_3082_MOESM8_ESM.pdf]

老年脑卒中患者急性期营养不良的影响因素分析及其风险预测模型的构建▲

张倩[1,2]　黄宏汰[2]　方金菊[2]　阮贞[3]　庞舒娴[1]　汪莉[1*]

1 广西医科大学第三附属医院/南宁市第二人民医院护理部，南宁市　530000；2 广西中医药大学护理学院，南宁市　530000；3 广西医科大学第三附属医院/南宁市第二人民医院神经内科，南宁市　530000

【摘要】 目的　探讨老年脑卒中患者急性期营养不良的影响因素及其风险预测模型的价值。方法　选取192 例老年急性期脑卒中患者，使用微型营养评估法评估患者的营养状态，并据此将患者分为营养不良组和非营养不良组。对老年脑卒中患者急性期营养不良的危险因素进行单因素分析和多因素 logistic 回归分析，构建老年脑卒中患者急性期营养不良风险预测模型并绘制列线图和受试者操作特征（ROC）曲线。结果　192 例老年急性期脑卒中患者中有 59 例（30.73%）出现营养不良。多因素 logistic 回归分析结果显示，年龄增加、低体质量指数（BMI）、焦虑、认知障碍、低血红蛋白水平、低白蛋白水平均是老年脑卒中患者急性期发生营养不良的独立危险因素（均 $P<0.05$）。基于上述 6 项指标建立的老年脑卒中患者急性期营养不良风险预测模型，其 Hosmer-Lemeshow 检验结果显示，$\chi^2=2.852$，$P=0.943$；模型 ROC 曲线下面积为 0.897，灵敏度为 0.831，特异度为 0.820。结论　老年脑卒中患者急性期营养不良的发生率较高，年龄增加、低 BMI、焦虑、认知障碍、低血红蛋白水平、低白蛋白水平均是老年脑卒中患者急性期发生营养不良的独立危险因素；基于上述独立危险因素建立的老年脑卒中患者急性期营养不良的风险预测模型对老年脑卒中患者急性期发生营养不良有较好的预测能力。

【关键词】 脑卒中；急性期；老年人；营养不良；危险因素；logistic 回归分析；风险预测模型；列线图；受试者操作特征曲线

【中图分类号】 R 743　【文献标识码】 A　【文章编号】 1673-7768（2023）01-0008-06

DOI：10.16121/j.cnki.cn45-1347/r.2023.01.02

Analysis of influencing factors of malnutrition in elderly acute stroke patients and construction of its risk prediction model

ZHANG Qian[1,2], *HUANG Hongtai*[2], *FANG Jinju*[2], *RUAN Zhen*[3], *PANG Shuxian*[1], *WANG Li*[1]

1 *Department of Nursing*, *the Third Affiliated Hospital of Guangxi Medical University/the Second Nanning People's Hospital*, *Nanning* 530000, *Guangxi*, *China*; 2 *School of Nursing*, *Guangxi University of Chinese Medicine*, *Nanning* 530000, *Guangxi*, *China*; 3 *Department of Neurology*, *the Third Affiliated Hospital of Guangxi Medical University/the Second Nanning People's Hospital*, *Nanning* 530000, *Guangxi*, *China*

【Abstract】 **Objective** To explore the influencing factors of malnutrition in elderly acute stroke patients and the value of its risk prediction model. **Methods** A total of 192 elderly acute stroke patients were selected to assess their nutritional status by Mini-Nutritional Assessment, according to which the patients were divided into a malnutrition group or a non-malnutrition group. Univariate analysis and multivariate logistic regression analysis were carried out to find the risk factors of malnutrition in elderly acute stroke patients, and a risk prediction model of malnutrition in elderly acute stroke patients was constructed, and the nomogram and receiver operating characteristic (ROC) curve were drawn. **Results** Of the 192 elderly acute stroke patients, 59 cases (30.73%) developed malnutrition. The results of multivariate logistic regression analysis showed that aging, low body mass index (BMI), anxiety, cognitive impairment, low hemoglobin level, and low albumin level were independent risk factors of malnutrition in elderly acute stroke patients (all $P<0.05$). A risk prediction model of malnutrition in elderly acute stroke patients was constructed based on the above 6 indicators, the Hosmer-Lemeshow test results showed that $\chi^2=2.852$, $P=0.943$; the area under the ROC curve of the model was 0.897, the sensitivity was

▲基金项目：广西科技计划项目（桂科 AB18221006，桂科 AA22096032）

* 通信作者：汪莉

(C)1994-2023 China Academic Journal Electronic Publishing House. All rights reserved. http://www.cnki.net

0.831, and the specificity was 0.820. **Conclusion** The incidence of malnutrition in elderly acute stroke patients is quite high, and aging, low BMI, anxiety, cognitive impairment, low hemoglobin level, and low albumin level are independent risk factors of malnutrition in elderly acute stroke patients; the risk prediction model of malnutrition in elderly acute stroke patients constructed based on the above independent risk factors has good capability to predict malnutrition in elderly acute stroke patients.

【Key words】 Stroke; Acute phase; Elder; Malnutrition; Risk factor; Logistic regression analysis; Risk prediction model; Nomogram; Receiver operating characteristic curve

脑卒中具有发病率高、复发率高、致残率高、病死率高的特点[1]。最新全球疾病负担研究表明,我国脑卒中疾病负担高于全球平均水平,并呈现增长趋势[2]。营养不良是脑卒中患者出现卒中后不良结局的重要原因,改善脑卒中患者营养不良情况有助于提高康复效果[3-5]。目前,关于脑卒中患者急性期营养不良的研究较少,临床上也缺乏针对老年脑卒中患者的特异性的营养不良筛查工具。因此,本研究对老年急性期脑卒中患者进行营养状况调查,分析其营养不良的危险因素并构建老年脑卒中患者急性期营养不良的风险预测模型,旨在帮助临床医护人员早期识别存在营养不良风险的高危患者,尽早制定并采取相应的营养措施。现报告如下。

1 对象与方法

1.1 研究对象 选取 2021 年 1 月至 2022 年 3 月在南宁市某三级甲等医院住院的 192 例老年脑卒中患者作为研究对象。纳入标准:(1)符合脑卒中诊断标准[6],并经颅脑 CT 或 MRI 影像学证实;(2)急性期患者(发病后 2 周内)[7];(3)年龄≥60 岁;(4)能够配合完成研究者;(5)均签署研究知情同意书。排除标准:(1)存在严重代谢性疾病、内分泌疾病和血液系统疾病者;(2)存在恶性肿瘤、严重肝肾功能衰竭和严重营养不良的患者。

1.2 方法

1.2.1 收集资料 于患者入院 48 h 内通过医院病历系统、与患者和家属进行面对面的问卷调查以收集相关资料。(1)一般人口学资料:性别、年龄、民族、文化程度、体质量指数(body mass index, BMI)、体育锻炼习惯、饮食习惯等。(2)病史和临床资料:脑卒中类型(缺血性脑卒中、脑出血性脑卒中)、疾病史(脑卒中、高血压、冠心病、糖尿病等)、吸烟史、饮酒史、美国国立卫生研究院卒中量表(National Institutes of Health Stroke Scale, NIHSS)评分[8](分数越高表示患者神经功能越差)、Barthel 指数[9](总分 0~100 分,100 分为无须依赖,61~99 分为轻度依赖,41~60 分为中度依赖,≤40 分为重度依赖)、洼田饮水试验评分[10](1~2 级为无吞咽障碍,3~5 级为有吞咽障碍)。(3)心理认知状况评估:焦虑自评量表评分[11](<50 分为无焦虑;≥50 为焦虑)、抑郁严重程度指数[11](指数<0.5 为正常,指数≥0.5 为抑郁)、蒙特利尔认知评估量表评分[12](满分 30 分,得分<26 分为认知障碍)。(4)实验室检查指标:红细胞计数、淋巴细胞计数,以及血红蛋白、血清白蛋白、总蛋白、血清前蛋白水平等。

1.2.2 营养不良的评价标准 采用微型营养评估法(Mini Nutritional Assessment, MNA)[13]评估患者的营养情况。MNA 是欧洲肠外肠内营养学会建议的一种简便、快捷、为老年人群筛查与评估营养状态的量表。MNA 包含测量人体的 4 项指标:BMI、体重下降、上臂围度、腓肠肌围;整体评价的 6 项指标:医疗的情况、用药的情况、生活的类型、神经或精神是否有异常、活动能力、是否长褥疮或皮肤是否有溃烂;膳食情况的 6 项指标:食欲、蛋白质的摄取情况、蔬菜水果的摄取情况、每日的餐次、摄水量、摄食行为模式;主观评价的 2 项指标:自评、他评。满分 30 分,各个项目分数相加即患者营养评估的得分。总评定结果得分<17 分提示患者营养不良,17~23 分提示有营养不良的风险,≥24 分提示营养状况良好。

患者的营养评估由主管医生、营养师、主管护士共同评估,患者入院后 48 h 开始第一次评估,往后每 3 d(或出现病情变化时)评估一次,持续评估至患者入院第 14 d(住院不满 14 d 的患者,则评估至其出院当天)。患者住院期间出现 MNA<17 分即判断其为营养不良,根据评估结果将患者分成营养不良组和非营养不良组。

1.3 统计学处理 应用 SPSS 26.0 软件对数据进行统计分析。正态分布的计量资料以 $\bar{x} \pm s$ 表示,两组间均数的比较采用独立样本 t 检验;非正态分布的计量资料以 $M(P_{25}, P_{75})$ 表示,比较采用秩和检验。计数资料以例数和百分数表示,两组间的比较采用 χ^2 检验。应用多因素 logistic 回归分析探讨老年脑卒中患者急性期营养不良的影响因素。使用 R 4.1.1

(C)1994-2023 China Academic Journal Electronic Publishing House. All rights reserved. http://www.cnki.net

和 rms 程序包构建老年脑卒中患者急性期营养不良的列线图风险预测模型；应用 Hosmer-Lemeshow 检验和受试者操作特征（receiver operating characteristic，ROC）曲线下面积评价模型的拟合优度与预测能力。以$P<0.05$为差异有统计学意义。

2 结 果

2.1 老年脑卒中患者急性期营养不良发生情况 本研究共纳入 192 例老年急性期脑卒中患者，营养不良发生率为 30.73%（59/192）。

2.2 老年脑卒中患者急性期营养不良的单因素分析 将 192 例老年急性期脑卒中患者分为营养不良组（59 例）和非营养不良组（133 例）。两组患者民族、脑卒中类型、脑卒中史、高血压史、冠心病史、糖尿病史、吸烟史、饮酒史、NIHSS 评分、洼田饮水试验评级、淋巴细胞计数的差异均无统计学意义（均$P>0.05$）；两组患者年龄、性别、文化程度、BMI、体育锻炼频率、饮食习惯、Barthel 指数、焦虑、抑郁、认知障碍、红细胞计数，以及血红蛋白、总蛋白、白蛋白、前蛋白水平的差异均有统计学意义（均$P<0.05$）。见表 1。

表 1 老年脑卒中患者急性期营养不良的单因素分析

变量	营养不良组（$n=59$）	非营养不良组（$n=133$）	$t/\chi^2/z$	P
年龄（$\bar{x}\pm s$，岁）	73.39±7.11	69.20±6.60	3.963	<0.001
性别（%）			4.427	0.035
男性	32(54.24)	93(69.92)		
女性	27(45.76)	40(30.08)		
文化程度（%）			13.689	0.001
小学及以下	49(83.05)	74(55.64)		
初中	8(13.56)	40(30.08)		
高中（中专）及以上	2(3.39)	19(14.29)		
民族（%）			0.040	0.842
汉族	45(76.27)	98(73.68)		
壮族	14(23.73)	35(26.32)		
BMI（$\bar{x}\pm s$，kg/m^2）	21.18±2.68	23.88±3.02	−5.901	<0.001
体育锻炼频率（%，h/周）			12.544	<0.001
≥8	47(79.66)	70(52.63)		
<8	12(20.34)	63(47.37)		
饮食习惯（%）			13.175	<0.001
饮食清淡	35(59.32)	52(39.10)		
饮食适中	19(32.20)	38(28.57)		
饮食油腻	5(8.47)	43(32.33)		
脑卒中类型（%）			2.138	0.144
缺血性脑卒中	51(86.44)	125(93.99)		
出血性脑卒中	8(13.56)	8(6.01)		
脑卒中史（%）	18(30.51)	58(43.61)	2.933	0.087
高血压史（%）	16(27.12)	40(30.08)	0.173	0.678
冠心病史（%）	13(22.03)	24(18.05)	0.418	0.518
糖尿病史（%）	13(22.03)	40(30.08)	1.322	0.250
吸烟史（%）	15(25.42)	33(24.81)	0.008	0.928
饮酒史（%）	16(27.12)	32(24.06)	0.204	0.652
NIHSS 评分（%，分）			0.331	0.847
0~1	26(44.07)	58(43.61)		
2~4	13(22.03)	34(25.56)		
≥5	20(33.90)	41(30.83)		
Barthel 指数（%，分）			13.593	0.004
100	3(5.08)	36(27.07)		
61~99	30(50.85)	52(39.10)		
41~60	12(20.34)	27(20.30)		
≤40	14(23.73)	18(13.53)		

(C)1994-2023 China Academic Journal Electronic Publishing House. All rights reserved. http://www.cnki.net

续表1

变量	营养不良组(n=59)	非营养不良组(n=133)	$t/\chi^2/z$	P
洼田饮水试验(%,级)			2.654	0.103
1~2	46(77.97)	116(87.22)		
3~5	13(22.03)	17(12.78)		
焦虑(%)	43(72.88)	52(39.10)	18.660	<0.001
抑郁(%)	36(61.02)	39(29.32)	17.247	<0.001
认知障碍(%)	38(64.41)	41(30.83)	19.031	<0.001
淋巴细胞计数[$M(P_{25},P_{75})$, $\times10^9$/L]	1.44(1.00,1.93)	1.56(1.07,2.28)	-1.486	0.137
红细胞计数($\bar{x}\pm s$, $\times10^{12}$/L)	4.15±0.78	4.65±0.72	-4.326	<0.001
血红蛋白($\bar{x}\pm s$, g/L)	119.53±16.70	134.23±18.00	-5.336	<0.001
总蛋白($\bar{x}\pm s$, g/L)	62.98±6.76	65.60±5.08	-2.967	0.003
白蛋白($\bar{x}\pm s$, g/L)	36.15±3.59	39.09±2.92	-5.986	<0.001
前蛋白($\bar{x}\pm s$, mg/L)	194.14±57.67	230.11±49.64	-4.403	<0.001

2.3 老年脑卒中患者急性期营养不良的多因素logistic回归分析 将单因素分析中差异有统计学意义的因素作为自变量,以是否发生营养不良作为因变量进行多因素logistic回归分析,结果显示:年龄增加、低BMI、焦虑、认知障碍、低血红蛋白水平、低白蛋白水平均是老年脑卒中患者急性期发生营养不良的独立危险因素(均$P<0.05$)。见表2、表3。

2.4 老年脑卒中患者急性期营养不良风险预测模型的建立 基于年龄增加、低BMI、焦虑、认知障碍、低血红蛋白水平、低白蛋白水平等老年脑卒中患者急性期营养不良的危险因素,建立老年脑卒中患者急性期营养不良风险预测模型并绘制列线图(见图1)。Hosmer-Lemeshow检验结果显示,$\chi^2=2.852$,$P=0.943$。该模型ROC曲线下面积为0.897($P<0.001$),约登指数最大值为0.651,最佳临界值为0.288,其灵敏度为0.831,特异度为0.820,见图2。

表2 变量赋值表

变量	赋值
因变量	
营养不良	否=0;是=1
自变量	
年龄	实际观测值
性别	男=0;女=1
文化程度	小学及以下=1;初中=2;高中(中专)及以上=3
BMI	实际观测值
体育锻炼频率	<8 h/周=0;≥8 h/周=1
饮食习惯	饮食清淡=1;饮食适中=2;饮食油腻=3
Barthel指数	100分=1;61~99分=2;41~60分=3;≤40分=4
焦虑	无=0;有=1
抑郁	无=0;有=1
认知障碍	无=0;有=1
红细胞计数	实际观测值
血红蛋白	实际观测值
总蛋白	实际观测值
白蛋白	实际观测值
前蛋白	实际观测值

表3 老年脑卒中患者急性期营养不良的多因素logistic回归分析

因素	B	$S.E.$	Wald χ^2	P	OR(95% CI)
常量	12.024	4.181	8.272	0.004	
年龄	0.082	0.032	6.363	0.012	1.085(1.018,1.157)
BMI	-0.303	0.079	14.730	0.000	0.739(0.633,0.862)
焦虑	1.218	0.452	7.253	0.007	3.380(1.393,8.203)
认知障碍	1.153	436	6.991	0.008	3.168(1.348,7.452)
血红蛋白	-0.039	013	9.082	0.003	0.962(0.937,0.986)
白蛋白	-0.215	069	9.671	0.002	0.807(0.704,0.923)

(C)1994-2023 China Academic Journal Electronic Publishing House. All rights reserved. http://www.cnki.net

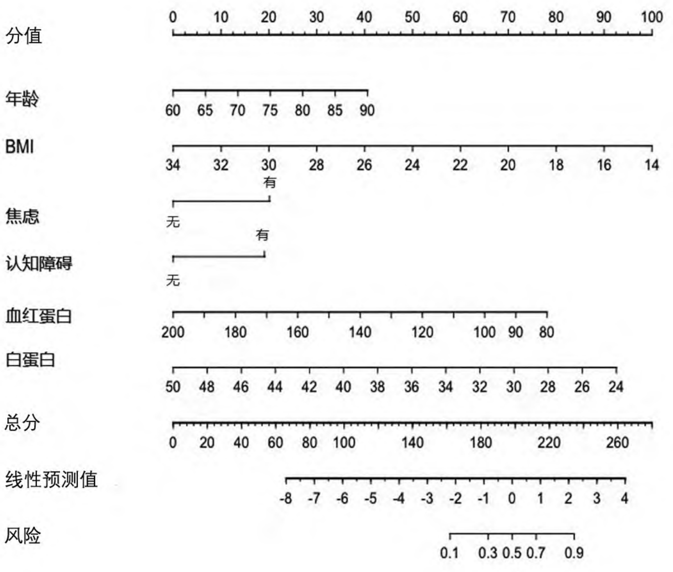

图1 老年脑卒中患者急性期营养不良风险预测模型列线图

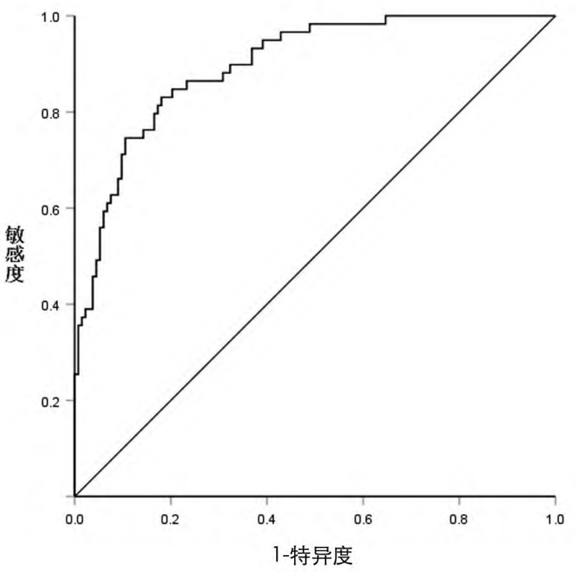

图2 老年脑卒中患者急性期营养不良风险预测模型的 ROC 曲线

3 讨 论

3.1 老年脑卒中患者急性期营养不良的发生情况

近年来有研究证实营养不良是影响脑卒中预后的独立危险因素，有增加病死率和感染的风险[4]。脑卒中患者营养不良发生率在 6.1% ~62.0% 之间[3]。本研究结果显示: 老年脑卒中患者急性期营养不良发生率为 30.73%。老年人机体器官功能衰退，多存在营养吸收功能不良，若合并慢性病则更能引起代谢功能障碍，更易导致营养摄入不足，致使老年脑卒中患者在发病之前或已经存在营养不良发生风险，且脑卒中急性期患者机体处于高应激状态，蛋白质分解加快，发生代谢紊乱，加剧营养成分消耗; 加之脑卒中患者多伴有吞咽困难、意识障碍、肢体活动功能障碍，营养摄入困难，导致营养不良的风险更高[14]。因此，护理人员应该尽早对入院老年脑卒中患者进行营养筛查和评估，随时追踪患者的营养状况，尤其是在急性期，在药物治疗和康复训练的同时要持续监测膳食摄入和营养状况，并根据患者的个体需求制定合理的营养方案，避免营养不良的发生。

3.2 老年脑卒中患者急性期营养不良的影响因素

本研究结果显示: 年龄增加是老年脑卒中患者急性期营养不良的独立危险因素，这与唐容等[15]的研究结果相似。随着年龄的增长，患者身体功能逐渐下降，日常生活能力不足，进而使患者的营养状态受到影响。因此，护理人员应关注高龄脑卒中患者，尽早发现其存在的营养不良风险并及时进行干预。有研究[16]证实，BMI 与脑卒中患者急性期发生营养不良的风险密切相关。本研究发现低 BMI 的老年脑卒中患者急性期发生营养不良的风险增加，这与 Wong 等[17]的研究结果一致。但脑卒中患者常合并肢体功能障碍和意识障碍，难以准确测量其体重和身高，这给应用 BMI 评估患者的营养不良风险带来挑战。本研究结果显示，体育锻炼 <8 h/周是老年脑卒中患者急性期发生营养不良的独立危险因素。既往研究[18]表明，活动能力较差的脑卒中住院患者发生营养不良的风险较高。分析其原因可能为，老年脑卒中患者入院前若缺乏体育锻炼，活动量减少，机体需求热量降低，摄入热量减少，或可导致其出现营养不良，且如果患者住院后存在肢体活动障碍，其康复锻炼效果欠佳，更能严重影响营养吸收，增加营养不良风险。

焦虑和抑郁是脑卒中患者普遍存在的并发症[19]。本研究结果显示: 焦虑是老年脑卒中患者急性期发生营养不良的独立危险因素。部分老年脑卒中患者可能因生活难以自理、生存质量下降而出现焦虑情绪，极大地影响其食欲从而导致营养不良的发生。因此应关注老年急性期脑卒中患者焦虑情绪，给予更多的人文关怀和针对性的心理疏导，并对患者家属做好宣教，提高患者的信心，缓解焦虑情绪。此外本研究还发现认知障碍患者发生营养不良的风险是认知功能正常患者的 3.168 倍。患者发生脑卒中后，可能出现认知障碍，导致患者进食行为发生改变，进食量减少甚至不能进食，从而影响患者的营养状况[20]。因此医护需要关注患者的认知功能，及时实施认知功能的评定，对认知障碍的患者应尽早给予干预，以更好地促进其机体功能恢复，降低营养不良发生的风险。本研究结果显示: 低血红蛋白水平是老年脑卒中患者急性期发生营养不良的独立危险因素。

(C)1994-2023 China Academic Journal Electronic Publishing House. All rights reserved. http://www.cnki.net

老年人常常因为营养物质的缺乏、慢性疾病、慢性炎症等，导致血红蛋白合成减少[21]。本研究结果显示：低白蛋白水平是老年脑卒中患者急性期发生营养不良的独立危险因素。白蛋白主要由肝脏合成，血清白蛋白是蛋白质-能量营养不良的一个临床标志[22]。脑卒中急性期患者容易出现肝脏功能异常状态，造成白蛋白水平降低，影响其营养状况。

3.3 老年脑卒中患者急性期营养不良风险模型的建立 基于上述独立危险因素，建立老年脑卒中患者急性期营养不良风险预测模型并绘制列线图，Hosmer-Lemeshow 检验结果表明模型的预测结果与实际发生情况的吻合度较高。该模型的 ROC 曲线下面积为 0.897，灵敏度为 0.831，特异度为 0.820，提示该模型用于预测老年脑卒中患者急性期发生营养不良的效果较好。有研究表明，脑卒中急性期进行营养补充可改善脑卒中患者的营养状况，降低营养不良的发生率，改善患者预后[23]。因此，本模型可应用于早期识别营养不良的老年脑卒中患者。

综上所述，老年脑卒中患者急性期营养不良发生率较高，年龄增加、低 BMI、焦虑、认知障碍和低血红蛋白、白蛋白水平均是老年脑卒中患者急性期发生营养不良的独立危险因素，基于上述独立危险因素建立的老年脑卒中患者急性期营养不良的风险预测模型对老年脑卒中患者急性期发生营养不良有较好的预测能力。本研究的局限性在于：为单中心研究，可能存在样本选择偏倚；未评估患者入院前营养状况，无法确定患者是在卒中前还是卒中后发生的营养不良；未纳入与临床医护人员相关的影响因素。

参 考 文 献

[1] 《中国脑卒中防治报告》编写组.《中国脑卒中防治报告 2019》概要[J]. 中国脑血管病杂志，2020，17(5)：272-281.

[2] 于洗河，高尚，贾欢欢，等. 1999 年、2009 年、2019 年我国与全球脑卒中疾病负担研究[J]. 中国卫生经济，2021，40(6)：58-61.

[3] 任姗姗，杨子艳，李冠臻，等. 老年脑卒中患者应用全球营养领导人发起的营养不良标准评价营养状态的研究[J]. 中华老年医学杂志，2022，41(3)：271-275.

[4] Nip WR，Perry L，McLaren S，et al. Dietary intake，nutritional status and rehabilitation outcomes of stroke patients in hospital[J]. J Hum Nutr Diet，2011，24(5)：460-469.

[5] Cai ZM，Wu YZ，Chen HM，et al. Being at risk of malnutrition predicts poor outcomes at 3 months in acute ischemic stroke patients[J]. Eur J Clin Nutr，2020，74(5)：796-805.

[6] 中华医学会神经病学分会，中华医学会神经病学分会脑血管病学组. 中国各类主要脑血管病诊断要点 2019[J]. 中华神经科杂志，2019，52(9)：710-715.

[7] 中华医学会神经病学分会，中华医学会神经病学分会脑血管病学组. 中国急性缺血性脑卒中诊治指南 2018[J]. 中华神经科杂志，2018，51(9)：666-682.

[8] 崔娟，崔晨曦，刘长春，等. 血栓通对急性缺血性脑卒中患者 NIHSS 评分、改良 Barthel 指数的影响[J]. 现代中西医结合杂志，2020，29(35)：3959-3962，3977.

[9] 徐玉琴，王雁南. Barthel 指数评分与营养水平对老年心内科患者压力性损伤发生的影响[J]. 中国老年学杂志，2022，42(24)：6005-6008.

[10] 孙宁. 洼田饮水试验在急性脑卒中后吞咽障碍患者中的应用价值[J]. 中国医药指南，2020，18(1)：88-89.

[11] 段泉泉，胜利. 焦虑及抑郁自评量表的临床效度[J]. 中国心理卫生杂志，2012，26(9)：676-679.

[12] 杨谦，第五永长，王威，等. 蒙特利尔认知评估量表用于社区轻度认知障碍筛查的初步研究[J]. 中风与神经疾病杂志，2022，39(2)：139-142.

[13] Cereda E. Mini nutritional assessment[J]. Curr Opin Clin Nutr Metab Care，2012，15(1)：29-41.

[14] 周峰，李新毅. 重症脑卒中患者早期动态营养评估及治疗策略的研究[J]. 中国医药指南，2015，13(13)：79.

[15] 唐容，段佳林，李倩茜，等. 脑卒中患者营养不良相关因素的系统评价[J]. 现代临床护理，2022，21(4)：66-76.

[16] Hsieh DY，Hung JW，Chang KC，et al. Malnutrition in acute stroke patients stratified by stroke severity- A hospital based study[J]. Acta Neurol Taiwan，2017，26(3)：120-127.

[17] Wong HJ，Harith S，Lua PL，et al. Prevalence and predictors of malnutrition risk among post-stroke patients in outpatient setting：a cross-sectional study[J]. Malays J Med Sci，2020，27(4)：72-84.

[18] 吴友红，章学媛，王益丽. 脑卒中住院患者营养风险的相关危险因素分析及预测模型构建[J]. 现代实用医学，2022，34(8)：999-1002.

[19] Kim Y，Kim MC，Park HS，et al. Association of the anxiety/depression with nutrition intake in stroke patients[J]. Clin Nutr Res，2018，7(1)：11-20.

[20] 陈胜云，王拥军，赵性泉. 住院卒中患者合并营养不良相关因素分析[J]. 中国卒中杂志，2010，5(8)：619-625.

[21] 李娟，黄沈珺，潘月龙，等. 晚期肿瘤患者营养状况检测指标的研究[J]. 重庆医学，2015，44(35)：4953-4954，4957.

[22] Zhang Q，Lei YX，Wang Q，et al. Serum albumin level is associated with the recurrence of acute ischemic stroke[J]. Am J Emerg Med，2016，34(9)：1812-1816.

[23] 马兰. 脑卒中患者急性期肠内营养支持方法的探讨[J]. 临床护理杂志，2011，10(2)：42-43.

（收稿日期：2022-11-06 修回日期：2023-01-17）

(C)1994-2023 China Academic Journal Electronic Publishing House. All rights reserved. http://www.cnki.net
