# Supplementary material for: Development and external validation of machine learning models for the early prediction of malnutrition in critically ill patients: a prospective observational study
Source: BMC Med Inform Decis Mak. 2025 Jul 3;25:248. doi: 10.1186/s12911-025-03082-9 (PMC12225150; doi:10.1186/s12911-025-03082-9)
Supplement: Supplementary file 9 — Supplementary Material 9 [file 12911_2025_3082_MOESM9_ESM.pdf]

DOI:10.3969/j.issn.1672-9463.2021.08.003

胃癌术后营养不良风险预测模型的构建及验证

杨眉 余家密 林雅 焦永娟 青菁

【摘要】 **目的** 分析胃癌术后 3 个月出现营养不良的危险因素并建立预测模型。**方法** 选取 2017 年 7 月~2019 年 3 月我院手术治疗的 263 例胃癌患者,采用患者主观整体评估量表(Patient-generated subjective globe assessment, PG-SGA)评估患者的营养状态。建立营养不良的风险预测模型,并选取 58 例患者验证预测模型的效果。**结果** 胃癌术后 3 个月有 60 例(22.8%)患者出现营养不良。单因素分析显示年龄、术前营养不良、全胃切除、术后并发症以及术后辅助化疗是发生术后营养不良的危险因素(P<0.05)。多因素分析显示年龄、术前营养不良、全胃切除、术后并发症以及术后辅助化疗是术后营养不良的独立危险因素(P<0.05)。本模型 ROC 曲线下面积为 0.849,灵敏度为 81.3%,特异度为 80.0%。模型验证结果:灵敏度为 80.0%,特异度为 89.6%,提示其预测效果较好。**结论** 胃癌术后营养不良的发生率较高,医护人员可根据预测模型判断患者营养不良的发生风险,及时对高危患者采取强化营养支持。

【关键词】 胃癌 营养不良 风险预测模型 高危因素

Establishment and verification of the risk prediction model for malnutrition after gastrectomy *Yang Mei, Yu Jiami, Lin Ya.*

Department of Medical Oncology, Fujian Medical University Cancer Hospital,Fujian Cancer Hospital, Fuzhou 350014

【Abstract】 **Objective** To analyze the risk factors of malnutrition 3 months after operation of gastric cancer and establish a risk prediction model for malnutrition. **Methods** A total of 263 patients with gastric cancer who were treated in our hospital from Jul 2017 to Mar 2019 were selected. The patient-generated subjective globe assessment (PG-SGA) was used to assess the nutritional status of the patients. A risk prediction model for malnutrition was established, and 58 patients were selected to verify the effect of the prediction model. **Results** A total of 60 (22.8%) patients with gastric cancer occured malnutrition 3 months after gastrectomy. Univariate analysis showed that age, preoperative malnutrition, total gastrectomy, postoperative complications and postoperative adjuvant chemotherapy were risk factors for postoperative malnutrition (P<0.05). Multivariate analysis showed that age, preoperative malnutrition, total gastrectomy, postoperative complications and postoperative adjuvant chemotherapy were independent risk factors for postoperative malnutrition (P<0.05). The area under ROC curve of this model was 0.849, the sensitivity was 81.3% and the specificity was 80.0%. Model verification results showed that the sensitivity was 80.0%, the specificity was 89.6%, it suggested that the prediction effect was better. **Conclusion** The incidence of malnutrition after gastrectomy is high. Medical staff can judge the risk of malnutrition in patients according to the prediction model, and promptly adopt intensive nutritional support for high-risk patients.

【Key words】 Gastric cancer Malnutrition Risk prediction model High risk factor

胃癌是世界第五大常见恶性肿瘤,第三大癌症相关性死亡原因[1]。根治性切除手术是治疗胃癌的主要方式。胃癌术后患者平均体重较术前下降10%~20%,营养不良的状况甚至需要长达 1 年才恢复[2]。术中消化道重建引起机体储存食物功能下降,进而影响消化吸收功能,导致进食量减少、体重减轻和营养状况恶化[3]。有研究表明,营养不良引起胃癌患者抵抗力减弱、组织愈合能力下降,并且增加肿瘤复发、降低患者 3 年无病生存率和总生存率[4,5]。本研究探讨胃癌术后 3 个月出现营养不良的危险因素并建立预测模型,以辅助医护人员尽早识别高危患者,并制定相应的强化营养措施。

作者单位:福建医科大学附属肿瘤医院 福建省肿瘤医院肿瘤内科,福建 福州 350014

1　材料与方法

1.1 一般资料　选取 2017 年 7 月~2019 年 3 月在我院手术治疗的胃癌患者 263 例。纳入标准:①病理明确为胃腺癌;②施行根治性手术;③配合良好、沟通正常、可完成调查问卷表;④临床病理资料完整,定期复查,随访可靠。排除标准:①因梗阻、出血等原因急诊手术者;②合并其他部位恶性肿瘤者;③术后 3 个月内死亡者。

1.2 评价标准　营养不良的诊断标准尚未统一,患者主观整体评估(Patient-generated subjective globe assessment, PG-SGA)是美国营养师协会和中国抗癌协会肿瘤营养专业委员会推荐用于评估肿瘤患者营养状况的量表,该量表分成三个等级:SGA-A 级(营养良好)评分为 0~3 分;SGA-B 级(中度营养不良)评分为 4~8 分;SGA-C 级(严重营养不良)评分 >8 分。得分≥4 分为营养不良, <4 分为非营养不良 [6]。由于胃癌术后 3 个月是患者发生营养不良的高峰期,且患者常在此期间进行常规术后肿瘤复查 [4],因此,本研究选择该时间段评估患者的营养状况。

1.3 统计学方法　采用 SPSS 22.0 统计学软件进行数据分析,计数资料以例数表示,单因素分析采用卡方检验,并将有统计学意义的参数采用二元 Logistic 回归进行多因素分析,建立风险预测模型,采用 ROC 曲线下面积检验模型预测效果。以 $P<0.05$ 表示差异具有统计学意义。

2　结果

2.1 胃癌术后患者营养不良发生情况　胃癌术后 3 个月共有 60 例(22.8%)患者发生营养不良,作为营养不良组;203 例(77.2%)营养状况良好,作为非营养不良组。其中 SGA-A 级 203 例,SGA-B 级 37 例,SGA-C 级 23 例。

2.2 胃癌术后患者营养不良的单因素和多因素分析　单因素分析表明性别、文化程度、婚姻状况、手术方式、吸烟、糖尿病、高血压、肿瘤大小、病理分期与术后 3 个月营养不良无关($P>0.05$)。年龄、术前营养不良、手术切除范围、术后并发症以及术后辅助化疗与术后 3 个月营养不良的发生相关($P<0.05$),见表 1。多因素分析表明年龄、术前营养不良、全胃切除、术后并发症以及术后辅助化疗是术后营养不良的独立危险因素($P<0.05$),见表 2。模型公式为 Z=0.728×年龄+1.071×术前营养不良+1.308×全胃切除+1.240×术后并发症+1.403×术后辅助化疗-3.887。

表 1　胃癌患者术后发生营养不良的单因素分析(n)

项目	营养不良组(n=60)	非营养不良组(n=203)	χ^2	P
性别			0.672	0.412
男	38	140		
女	22	63		
年龄(岁)			8.182	0.004
≥60	33	70		
<60	27	133		
文化程度			1.520	0.218
初中及以下	49	150		
高中及以上	11	53		
婚姻状况			1.383	0.240
已婚	45	136		
未婚/离异	15	67		
手术方式			1.071	0.301
腹腔镜手术	25	100		
开放手术	35	103		
吸烟			2.332	0.127
无	48	142		
有	12	61		
糖尿病			2.589	0.108
无	35	141		
有	25	62		
高血压			0.418	0.518
无	42	133		
有	18	70		
肿瘤大小(cm)			2.547	0.110
≤5	24	105		
>5	36	98		
病理分期			4.050	0.132
Ⅰ期	9	51		
Ⅱ期	15	58		
Ⅲ期	36	94		
术前营养不良			8.429	0.004
是	18	28		
否	42	175		
手术切除范围			17.399	0.000
全胃切除	34	56		
远端胃大部切除	26	147		

续表 1

项目	营养不良组（n=60）	非营养不良组（n=203）	χ^2	P
术后并发症			11.665	0.001
有	22	33		
无	38	170		
术后辅助化疗			13.236	0.000
是	49	113		
否	11	90		

2.3 ROC 曲线对胃癌术后营养不良风险预测模型的验证　绘制 ROC 曲线验证胃癌术后营养不良风险预测模型的效果，以约登指数最大值为最佳临界值。ROC 曲线下面积为 0.849[95%*CI*（0.790，0.907）]，约登指数为 0.881，灵敏度为 81.3%，特异度为 80.0%，见图 1。

2.4 胃癌术后营养不良风险预测模型的应用效果　选取 2019 年 5 月~2020 年 1 月我院治疗的 58 例胃癌患者作为研究对象，验证营养不良风险预测模型。当 *Z* 值≥0.881 时表明可能出现营养不良。本模型预测 13 例患者出现营养不良，45 例未出现营养不良。实际结果为 10 例发生营养不良，48 例未发生营养不良。预测结果与实际结果比较，本预测模型灵敏度为 80.0%（8/10），特异度为 89.6%（43/48）。

表 2　胃癌患者术后发生营养不良的多因素分析

危险因素	β	*SE*	*Wald*	*P*	*OR*	95%*CI*
年龄（≥ 60 岁）	0.728	0.332	4.801	0.028	2.071	1.080~3.972
术前营养不良（是）	1.071	0.398	7.261	0.007	2.919	1.339~6.361
手术切除范围（全胃切除）	1.308	0.338	14.996	0.000	3.698	1.908~7.170
术后并发症（有）	1.240	0.375	10.933	0.001	3.457	1.657~7.210
术后辅助化疗（是）	1.403	0.400	12.287	0.000	4.068	1.856~8.916
常量	−3.887	1.042	19.907	0.000	0.000	

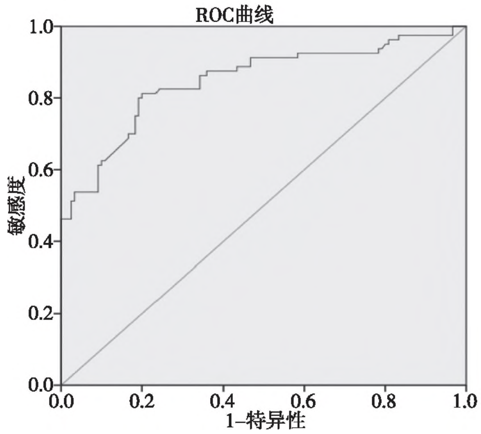

图 1　预测胃癌术后发生营养不良的 ROC 曲线

3　讨论

本研究中术后 3 个月营养不良的发生率高达 22.8%。Fujiya 等 [7] 对 760 例胃癌根治性手术患者进行回顾性研究，发现术后营养不良对总生存率有显著影响。Eo 等 [8] 将 314 例根治性手术切除胃癌患者纳入研究，发现与营养不良反应风险低的患者相比，营养不良反应风险高的患者 5 年无病生存率和 5 年生存率均较低。原因考虑为：非自主性体重下降导致 T 细胞活性降低和肠道内环境恶化，导致全身免疫功能减弱。同时，营养不良增加术后辅助治疗的副作用，导致化疗剂量减少甚至中止化疗，进而影响患者的生存时间 [9]。因此，加强术后营养管理，保持良好的营养状态，对促进患者尽早恢复非常重要。

预测模型对预测疾病发展、筛查未确诊患者、指导临床决策以及判断治疗效果至关重要。当 ROC 曲线下面积 <0.7 时表示预测效能低；0.7≤ROC 曲线下面积≤0.9 时表示预测效能中等；ROC 曲线下面积 >0.9 时表示预测效能高。本预测模型 ROC 曲线下面积为 0.849，表示预测效果较为理想。对预测模型进行验证，结果显示灵敏度为 80.0%，特异度为 89.6%，表明预测效果较好。有研究表明，口服营养补充可改善进展期胃癌患者全胃切除术后辅助化疗期间的营养状况，降低营养不良的发生率 [10]。因此，利用该模型有利于医护人员早期识别营养不良的患者，提前采取全面的营养支持。干预措施包括：强化营养教育和膳食指导、口服营养补充；存在进食障碍的患者可考虑鼻饲喂养；经口进食 + 肠内营养仍无法满足机体需要者建议肠外营养。

胃癌患者由于消化吸收功能受损、上腹部疼痛不适，幽门梗阻导致食物摄入量减少，肿瘤分解代谢增多引起机体慢性消耗、失血，大约 20.0% 的患

者在术前出现营养不良[11]。本研究中,术前营养不良是术后营养不良的重要原因。一方面,术前营养不良造成术后恢复延迟、并发症增多和住院时间延长。另一方面,术前营养不良表明肿瘤消耗更多且临床分期较晚,需要清扫淋巴结的范围更大,对患者 的创伤更严重。我国《胃癌胃切除手术加速康复外科专家共识(2016 版)》指出,具有术前营养不良的患者需要进行≥ 1 周的术前营养支持治疗[12]。

术后辅助化疗可以消灭微小肿瘤病灶、抑制肿瘤细胞生长、延长患者生存时间。然而,术后辅助化疗是发生术后营养不良的独立危险因素。化疗常伴随恶心、呕吐、腹泻、口腔粘膜炎、味觉改变,导致患者食欲下降、摄入量减少,从而导致体重下降、营养不良。因此,需要积极处理化疗的不良反应,并加强饮食指导。

胃癌血供丰富、淋巴结清扫难度大,术后并发症总发生率为 20%[13]。并发症延长患者的住院时间、增加心理负担以及加重全身炎症反应,使机体处于负氮平衡。本研究中,术后并发症是营养不良的独立危险因素。对出现术后并发症的患者需要强化营养支持,促进术后康复。

患者年龄越大,术后越容易出现营养不良。老年人由于生理机能减退、新陈代谢减慢,从而导致术后恢复延迟。同时,老年患者常合并糖尿病等基础疾病,术后容易出现切口感染、肺炎等并发症。

相对于远端胃大部切除患者,全胃切除更容易出现营养不良。全胃切除的手术范围更大、手术及麻醉时间更长、术中出血量更多,从而延长术后恢复时间。胃具有储存食物、分泌消化液、消化和吸收的功能。全胃切除引起消化液分泌减少、肠道动力和体液分泌失调,导致患者营养状况不佳。因此,专家建议在保证手术治疗效果的前提下,尽可能保留残胃功能,减少对患者术后生活质量的影响[14]。

总之,术后营养不良对患者的预后产生不利影响,本研究构建胃癌术后营养不良的风险预测模型,可有效预测营养不良的发生风险,有助于临床上早期识别营养不良高危人群并及时进行干预,以改善患者的预后。

参 考 文 献

1 Bray F, Ferlay J, Soerjomataram I, et al.Global cancer statistics 2018:GLOBOCAN estimates of incidence and mortality worldwide for 36 cancers in 185 countries[J].CA Cancer J Clin,2018,68:394-424

2 Lee HH,Park JM,Song KY,et al.Survival impact of postoperative body mass index in gastric cancer patients undergoing gastrectomy[J].Eur J Cancer,2016,52:129-137

3 Guo ZQ, Yu JM, Li W, et al.Survey and analysis of the nutritional status in hospitalized patients with malignant gastric tumors and its influence on the quality of life[J].Support Care Cancer,2020,28(1):373-380

4 Hirahara N, Tajima Y, Fujii Y, et al.Preoperative prognostic nutritional index predicts long-term outcome in gastric cancer:a propensity score-matched analysis[J].Anticancer Res,2018,38(8):4735-4746

5 朱希山,赵业,马飞艳,等.营养状态评价在胃癌患者全胃切除术预后中的价值[J].中华医学杂志,2021,101(6):421-428

6 Balstad TR, Bye A, Jenssen CR, et al.Patient interpretation of the Patient-generated subjective global assessment (PG-SGA) short form[J].Patient Prefer Adherence,2019,13:1391-1400

7 Fujiya K, Kawamura T, Omae K, et al.Impact of malnutrition after gastrectomy for gastric cancer on long-term survival[J].Ann Surg Oncol,2018,25(4):974-983

8 Eo WK, Chang HJ, Suh J, et al.The prognostic nutritional index predicts survival and identifies aggressiveness of gastric cancer[J].Nutr Cancer,2015,25(4):940-983

9 Zhou J, Hiki N, Mine S, et al.Role of prealbumin as a powerful and simple index for predicting postoperative complications after gastric cancer surgery[J].Ann Surg Oncol,2017,24:510-517

10 朱文强,吴震峰,吴丽红,等.口服营养补充降低进展期胃癌患者全胃切除术后辅助化疗期间营养不良风险的研究[J].中国肿瘤外科杂志,2021,13(1):33-36

11 Zheng HL, Lu J, Li P, et al.Effects of preoperative malnutrition on short- and long-term outcomes of patients with gastric cancer:Can we do better[J].Ann Surg Oncol,2017,24(11):3376-3385

12 中国研究型医院学会机器人与腹腔镜外科专业委员会.胃癌胃切除手术加速康复外科专家共识(2016 版)[J].中华消化外科杂志,2017,16(1):14-17

13 胡祥,张驰.日本和韩国胃癌术后并发症诊断及风险评价[J].中华胃肠外科杂志,2017,20(2):129-134

14 卫江鹏,余鹏飞,杜昆利,等.功能保留性胃切除术后的功能评价[J].中华胃肠外科杂志,2021,24(5):397-402

(收稿:2021-06-25)
