# Supplementary material for: Development and external validation of machine learning models for the early prediction of malnutrition in critically ill patients: a prospective observational study
Source: BMC Med Inform Decis Mak. 2025 Jul 3;25:248. doi: 10.1186/s12911-025-03082-9 (PMC12225150; doi:10.1186/s12911-025-03082-9)
Supplement: Supplementary file 10 — Supplementary Material 10 [file 12911_2025_3082_MOESM10_ESM.pdf]

# 老年重症脑卒中患者营养不良风险列线图预测模型的构建及验证

李君卓　　杨　雯　　刘光维▲　　张瑞昕　　阳佳家
重庆医科大学附属第一医院神经内科，重庆　400016

[摘要] 目的 通过分析老年重症脑卒中患者营养不良的危险因素，构建相应的列线图模型。方法 选取重庆医科大学附属第一医院神经内科重症监护室的老年重症脑卒中患者 782 例作为研究对象，将其分为建模组 547 例和验证组 235 例。根据血清白蛋白水平，将患者分为营养不良组、无营养不良组。通过 logistic 回归分析确认营养不良发生的影响因素，并构建营养不良风险列线图预测模型。应用 Bootstrap 法对模型的预测效果进行内部验证，利用验证组数据对模型进行外部验证。结果 两组年龄、慢性阻塞性肺疾病史、贫血、查尔森合并症指数（CCI）、格拉斯哥昏迷评分（GCS）、巴氏指数、营养支持方式、机械通气、中性粒细胞计数、红细胞压积、纤维蛋白原、白蛋白、总蛋白比例比较，差异有统计学意义（$P<0.05$）。logistic 回归分析显示，年龄、CCI、GCS 评分、贫血、中性粒细胞计数、总蛋白水平、营养支持方式为老年重症脑卒中患者营养不良的独立影响因素（$OR>1$，$P<0.05$）。构建模型的 ROC 曲线下面积为 0.858，内部验证结果显示，C-index 为 0.851；验证组 ROC 曲线下面积为 0.848。结论 本研究构建的列线图模型预测效能较好，可为临床早期预测老年重症脑卒中患者营养不良发生风险提供参考。

[关键词] 老年患者；脑卒中；营养不良；列线图
[中图分类号] R473.74　　[文献标识码] A　　[文章编号] 1673-7210(2023)02(b)-0028-05
DOI：10.20047/j.issn1673-7210.2023.05.06

## Construction and verification of a prediction model for malnutrition risk in senile patients with severe cerebral apoplexy

*LI Junzhuo　YANG Wen　LIU Guangwei▲　ZHANG Ruixin　YANG Jiajia*
Department of Neurology, the First Affiliated Hospital of Chongqing Medical University, Chongqing　400016, China

[Abstract] Objective To analyze the risk factors of malnutrition in senile patients with severe stroke, and to constructed the corresponding column graph model. Methods A total of 782 elderly patients with severe stroke in the intensive care Unit of Department of Neurology of the First Affiliated Hospital of Chongqing Medical University were selected as the research objects and divided into modeling group (547 cases) and validation group (235 cases). According to serum albumin level, patients were divided into malnutrition group and non-malnutrition group. The influencing factors of malnutrition were identified by logistic regression analysis, and the prediction model of malnutrition risk line graph was constructed. The prediction effect of the model was verified internally by Bootstrap method, and the model was verified externally by verification group data. Results There were statistically significant differences in age, history of chronic obstructive pulmonary disease, anemia, Charlson comorbidity index (CCI), Glasgow coma score (GCS), Barthel index, nutritional support, mechanical ventilation, neutrophil count, hematopoietic volume, proportion of fibrinogen, albumin, and total protein between the two groups ($P<0.05$). Logistic regression analysis showed that age, CCI, GCS score, anemia, neutrophil count, total protein level, and nutritional support were independent influencing factors for malnutrition in senile patients with severe stroke ($OR>1$, $P<0.05$). The area under ROC curve of the constructed model was 0.858, and the internal verification results showed that the C-index was 0.851. The area under ROC curve of the verification group was 0.848. Conclusion The prediction effect of the column graph model constructed in this study is good, and it can provide reference for early clinical prediction of malnutrition risk in elderly patients with severe stroke.

[Key words] Elderly patients; Stroke; Malnutrition; Nomogram

营养不良是老年脑卒中患者常见并发症之一，研究显示，其发生率为 26.2%～60.5%[1-3]。老年脑卒中患者由于常合并基础疾病，全身各器官功能都有衰退，由此引发摄食能力和吸收能力下降，进而出现营养不良。当此类患者病情进展至重症时，容易出现意识障碍、

[基金项目] 重庆市科卫联合医学科研项目（2021MSXM045）。
[作者简介] 李君卓（1997.7-），女，重庆医科大学第一临床学院 2020 级护理学专业在读硕士研究生；研究方向：神经重症护理。
▲通讯作者



(C)1994-2023 China Academic Journal Electronic Publishing House. All rights reserved.　http://www.cnki.net

肠道屏障功能障碍等症状，导致患者更易发生营养不良[4]，从而使患者不良结局的风险增加[5-6]。目前国内外关于老年重症脑卒中患者营养不良风险评估和危险因素研究的报道较少，且缺乏可视化、图形化、简便预测老年重症脑卒中患者营养不良的工具，基于 logistic 回归筛选变量构建预测营养不良列线图风险模型鲜有相关研究。因此，本研究旨在通过分析老年重症脑卒中患者营养不良的影响因素，构建并验证列线图预测模型，为早期预测老年重症脑卒中患者营养不良发生风险提供依据。

## 1 对象与方法

### 1.1 研究对象

选取 2018 年 1 月至 2020 年 12 月入住重庆医科大学附属第一医院神经内科重症监护室的患者作为研究对象。纳入标准：①年龄≥60 岁；②符合诊断标准[7]且经头颅 CT 或 MRI 诊断为脑卒中；③格拉斯哥昏迷评分（Glasgow coma scale，GCS）≤8 分或美国国立卫生研究院卒中量表（National Institutes of Health stroke scale，NIHSS）≥16 分[8]；④住院时间≥48 h。排除标准：①入院时已确诊营养不良；②入院血流动力学不稳定、多器官衰竭、严重肾脏疾病或肝脏疾病；③病历资料不完整。共纳入 782 例患者，按照时间顺序将 2018 年 1 月至 2019 年 12 月入住的 547 例作为建模组，2020 年 1 月至 12 月入住的 235 例作为验证组。本研究已获医院伦理委员会批准同意[2021 年科研伦理（2021-335）]。

### 1.2 研究方法

1.2.1 资料收集 采用回顾性研究法，通过查阅患者电子病历收集资料。①一般资料：年龄、性别、脑卒中类型、高血压史、糖尿病史、冠心病史、慢性阻塞性肺疾病史。②疾病相关资料：GCS 评分、NIHSS 评分、查尔森合并症指数（Charlson comorbidity index，CCI）、巴氏指数（Barthel index，BI）、营养支持方式（经口进食、鼻饲饮食、肠外营养）、是否使用机械通气。GCS 评分用于评估患者意识障碍程度，包括睁眼反应、肢体反应和语言反应 3 个部分，得分越高意识障碍越重[9]。NIHSS 评分评定患者神经功能缺损程度，总分 42 分，分数越高，提示神经功能缺损越严重[10]。CCI 包括疾病评估、严重程度评估和评分 3 个部分，用于评估患者合并症危险程度，评分越高提示患者预后越差[11]。BI 用于评定患者日常生活自理能力，总分 0～100 分，得分越低，提示自理能力越差[9]。上述评分均由医生在患者入院时评估，并录入电子病历系统。③实验室相关资料：白细胞计数、血细胞比容、中性粒细胞计数、淋巴细胞计数、纤维蛋白原、白蛋白、总蛋白、血红蛋白水平（贫血定义：女性血红蛋白浓度＜120 g/L，男性血红蛋白浓度＜130 g/L[12]）。实验室相关资料均为入院 24 h 内首次测量值。

1.2.2 分组方法 营养不良定义为血清白蛋白＜35 g/L[13]。连续监测患者在神经内科重症监护室期间的血清白蛋白水平，依据在此期间白蛋白最低值，将其分为无营养不良组（血清白蛋白≥35 g/L）和营养不良组（血清白蛋白＜35 g/L）。

### 1.3 统计学方法

采用 SPSS 25.0 软件对所得数据进行统计分析。符合正态分布的计量资料以均数±标准差（$\bar{x}\pm s$）表示。计数资料以例数或百分比表示，采用 $\chi^2$ 检验。logistic 回归分析确定营养不良发生的独立影响因素。使用 R4.1.1 软件构建列线图模型，受试者操作特征曲线（receiver operating characteristic curve，ROC）评价模型的预测能力，Bootstrap 法（自抽样次数为 2000）进行内部验证，并计算一致性指数（C-index），C-index 指数越接近于 1 提示列线图的预测能力越准确。基于构建的列线图模型，将验证组数据进行 ROC 分析，对模型实施外部验证。以 $P<0.05$ 为差异有统计学意义。

## 2 结果

### 2.1 老年重症脑卒中患者一般情况

782 例患者中，男 457 例，女 325 例；年龄 60～98 岁，平均（73.31±8.38）岁；305 例（39.0%）发生营养不良。建模组 547 例，男 324 例，女 223 例；年龄 60～98 岁，平均（73.47±8.37）岁；221 例（40.4%）发生营养不良。验证组 235 例，男 133 例，女 102 例；年龄 60～94 岁，平均（72.93±8.40）岁；84 例（35.7%）发生营养不良。

### 2.2 建模组老年重症脑卒中患者营养不良的单因素分析

结果显示，两组年龄、COPD 史、贫血、CCI、GCS、BI、营养支持方式、机械通气、中性粒细胞计数、红细胞压积、纤维蛋白原、白蛋白、总蛋白比例比较，差异有统计学意义（$P<0.05$）。见表 1。

### 2.3 建模组老年重症脑卒中患者营养不良的多因素 logistic 回归分析

将单因素分析中差异有统计学意义（$P<0.05$）的变量作为自变量，进行二元 logistic 回归分析。结果显示，年龄、CCI、GCS 评分、贫血、中性粒细胞计数、总蛋白水平、营养支持方式为老年重症脑卒中患者营养不良的独立影响因素（$OR>1$，$P<0.05$）。见表 2。

### 2.4 老年重症脑卒中患者营养不良风险列线图预测模型的建立

使用多因素分析筛选的营养不良的独立影响因素构建老年重症脑卒中患者发生营养不良的风险列线图模型，根据预测因子可在列线图中找到对应的分数，每项评分总和对应的值即为营养不良发生的概率。



(C)1994-2023 China Academic Journal Electronic Publishing House. All rights reserved. http://www.cnki.net

表 1　老年重症脑卒中患者营养不良的单因素分析（例）

| 特征 | 例数 | 无营养不良组（326 例） | 营养不良组（221 例） | $\chi^2$ 值 | $P$ 值 |
|---|---|---|---|---|---|
| 年龄（岁） | | | | 42.199 | <0.001 |
| <70 | 203 | 157 | 46 | | |
| ≥70 | 344 | 169 | 175 | | |
| 性别 | | | | 1.096 | 0.295 |
| 男 | 324 | 199 | 125 | | |
| 女 | 223 | 127 | 96 | | |
| 脑卒中类型 | | | | 1.198 | 0.274 |
| 脑梗死 | 397 | 231 | 166 | | |
| 脑出血 | 150 | 95 | 55 | | |
| 高血压病史 | | | | 2.736 | 0.098 |
| 有 | 409 | 252 | 157 | | |
| 无 | 138 | 74 | 64 | | |
| 糖尿病史 | | | | 0.186 | 0.667 |
| 有 | 179 | 109 | 70 | | |
| 无 | 368 | 217 | 151 | | |
| 冠心病史 | | | | 2.113 | 0.146 |
| 有 | 138 | 75 | 63 | | |
| 无 | 409 | 251 | 158 | | |
| COPD 史 | | | | 5.080 | 0.024 |
| 有 | 55 | 25 | 30 | | |
| 无 | 492 | 301 | 191 | | |
| 贫血 | | | | 19.472 | <0.001 |
| 有 | 132 | 57 | 75 | | |
| 无 | 415 | 269 | 146 | | |
| CCI（分） | | | | 90.373 | <0.001 |
| <4 | 281 | 222 | 59 | | |
| ≥4 | 266 | 104 | 162 | | |
| GCS 评分（分） | | | | 42.139 | <0.001 |
| ≤8 | 161 | 62 | 99 | | |
| >8 | 386 | 264 | 122 | | |
| NIHSS 评分（分） | | | | 0.726 | 0.394 |
| <16 | 64 | 35 | 29 | | |
| ≥16 | 483 | 291 | 192 | | |
| BI（分） | | | | 15.547 | <0.001 |
| <40 | 441 | 246 | 195 | | |
| 40～60 | 81 | 64 | 17 | | |
| >60 | 25 | 16 | 9 | | |
| 营养支持方式 | | | | 76.918 | <0.001 |
| 经口进食 | 132 | 119 | 13 | | |
| 鼻饲饮食 | 385 | 200 | 185 | | |
| 肠外营养 | 30 | 7 | 23 | | |
| 机械通气 | | | | 28.961 | <0.001 |
| 是 | 50 | 12 | 38 | | |
| 否 | 497 | 314 | 183 | | |
| 中性粒细胞计数 | | | | 50.391 | <0.001 |
| ≤6.3×$10^9$/L | 236 | 181 | 55 | | |
| >6.3×$10^9$/L | 311 | 145 | 166 | | |
| 红细胞压积 | | | | 5.2234 | 0.022 |
| <40% | 240 | 130 | 110 | | |
| ≥40% | 307 | 196 | 111 | | |
| 纤维蛋白原（g/L） | | | | 14.749 | <0.001 |
| ≤3 | 368 | 240 | 128 | | |
| >3 | 179 | 86 | 93 | | |
| 白蛋白（g/L） | | | | 9.290 | 0.002 |
| <40 | 334 | 182 | 152 | | |
| ≥40 | 213 | 144 | 69 | | |
| 总蛋白（g/L） | | | | 18.076 | <0.001 |
| <65 | 110 | 46 | 64 | | |
| ≥65 | 437 | 280 | 157 | | |

注：COPD：慢性阻塞性肺疾病；CCI：查尔森合并症指数；GCS：格拉斯哥昏迷评分；NIHSS：美国国立卫生研究院卒中量表；BI：巴氏指数

表 2　建模组老年重症脑卒中患者营养不良的多因素 logistic 回归分析

| 项目 | β | S.E. | Wald $\chi^2$ | $P$ 值 | $OR$ 值 | 95%$CI$ |
|---|---|---|---|---|---|---|
| 常数 | -4.730 | 0.442 | 114.601 | <0.001 | 0.009 | |
| 年龄 | 1.241 | 0.244 | 25.813 | <0.001 | 3.458 | 2.143～5.580 |
| CCI | 1.216 | 0.228 | 28.328 | <0.001 | 3.374 | 2.156～5.280 |
| GCS 评分 | 0.942 | 0.249 | 14.273 | <0.001 | 2.564 | 1.573～4.180 |
| 贫血 | 0.535 | 0.261 | 4.210 | 0.040 | 1.707 | 1.024～2.844 |
| 中性粒细胞计数 | 1.202 | 0.244 | 24.337 | <0.001 | 3.326 | 2.063～5.360 |
| 总蛋白 | 1.228 | 0.295 | 17.278 | <0.001 | 3.414 | 1.913～6.091 |
| 营养支持方式 | | | | | | |
| 鼻饲饮食 | 1.761 | 0.344 | 26.166 | <0.001 | 5.816 | 2.963～11.419 |
| 肠外营养 | 2.027 | 0.575 | 12.413 | <0.001 | 7.588 | 2.458～23.430 |

注：CCI：查尔森合并症指数；GCS：格拉斯哥昏迷评分

见图 1。

2.5 列线图预测模型的检验效能评价

建模组 ROC 曲线下面积为 0.858（95%$CI$：0.826～0.886）。使用 Bootstrap 进行内部验证，C-index 为 0.851，列线图预测精度良好。验证组 ROC 曲线下面积为 0.848（95%$CI$：0.795～0.891），见图 2。Hosmer-Lemeshow 拟合优度检验结果显示，$\chi^2$=5.196，$P$=0.636，提示该模型拟合较好。校准曲线在建模组和验证组都靠近标准曲线，表示预测值和实际观测值之间一致性较好。见图 3。

## 3 讨论

营养不良是脑卒中患者最常见的并发症之一，相关研究显示，营养不良是导致患者不良预后的重要因素[14]。因此，早期识别营养不良高风险人群，确定营养问题并实施相应的干预措施具有重要的临床意义。目前常用的营养不良风险筛查工具较多，包括营养风险筛查 2002、营养不良通用筛检工具等[15]，但以上工具更适用于意识清楚、沟通良好的患者，且资料收集内容较复杂，包括体重变化、饮食改变等方面，易受主观因素的影响，在老年重症脑卒中患者中应用较为局限。本研究通过分析老年重症脑卒中患者营养不良的影响因素，并在此基础上构建预测营养不良的风险列线图模型，希望为老年重症脑卒中患者营养不良高危人群制订营养支持方案提供参考。

本研究发现，年龄、CCI、GCS 评分、贫血、中性粒细胞计数、血清总蛋白水平、营养支持方式是老年重症脑卒中患者营养不良的独立影响因素。高龄患者因消化系统功能下降，咀嚼吞咽功能障碍等原因，是发生营养不良的高危人群[16]。高 CCI 患者因合并多种疾病，疾病消耗大，营养素的消耗越高，增加了营养不良的风险[17]。GCS 评分≤8 分表示严重的脑部损伤[18]，老年重症脑卒中患者常伴随意识障碍，即使最轻微的嗜睡也会影响患者进食进而影响其营养状况[19]。本研究结果显示，贫血是营养不良的危险因素，这与既往研



(C)1994-2023 China Academic Journal Electronic Publishing House. All rights reserved.　http://www.cnki.net

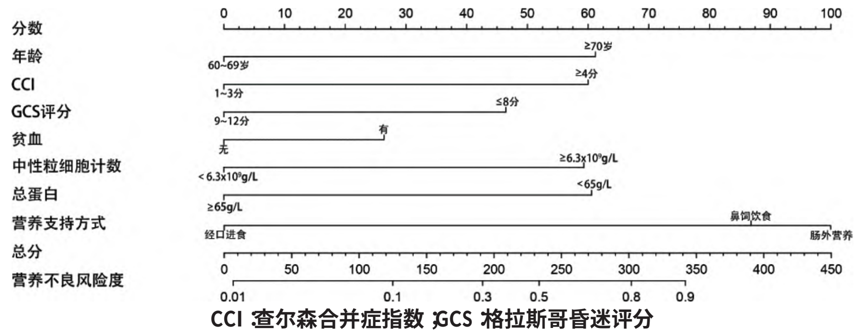


CCI：查尔森合并症指数；GCS：格拉斯哥昏迷评分

图 1　老年重症脑卒中患者营养不良风险列线图预测模型

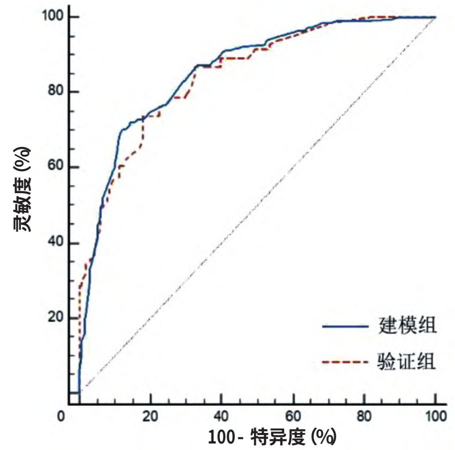


图 2　老年重症脑卒中患者营养不良风险列线图预测模型 ROC 曲线

究结果相似[20-21]。血红蛋白是反映老年营养不良风险的指标，可以反映体内铁代谢和蛋白质状况，其水平降低提示患者营养不足[22]。本研究结果发现，中性粒细胞计数增高，营养不良发生风险越高，这可能与炎症有关。炎症会增加循环细胞因子，激活蛋白质分解代谢以及导致体重下降[23]。低血清总蛋白水平的患者易发生营养不良可能与其蛋白质储备较低，加上摄入量减少，疾病消耗增加，导致营养物质的消耗增多有关。本研究表明，与经口进食的患者比较，鼻饲饮食和肠外营养的患者更容易发生营养不良。Chen 等[24]研究显示，管饲营养是脑卒中患者营养不良的危险因素，

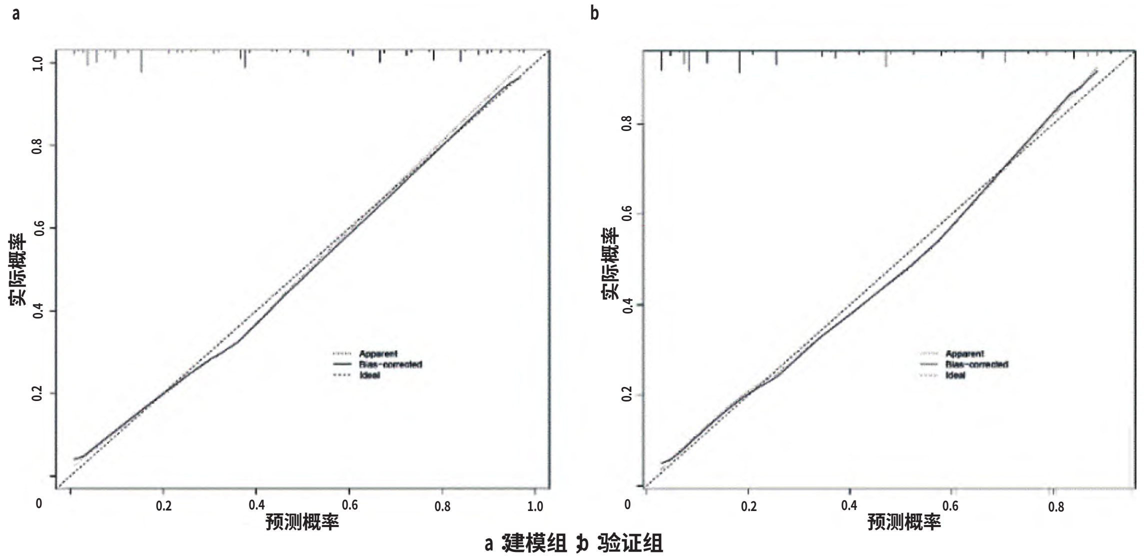


a：建模组；b：验证组

图 3　老年重症脑卒中患者营养不良风险列线图预测模型的校准曲线

这与本研究结果类似。这可能与鼻饲饮食会导致患者丧失对食物的兴趣，增加反流、误吸和肺部感染的风险有关。肠外营养可能会导致肠黏膜损害及胃肠功能障碍，增加了营养不良的风险[25]。

列线图能够将预测结果简洁化、可视化，直观地预测个体的临床结局，已广泛应用于多种疾病预后预测[26-29]。本研究构建的列线图模型具有较好的准确性、一致性及校准度，模型涉及指标基于患者常见的临床和实验室特征，易于获取，在患者入住神经内科重症监护室初期便可以使用，可为临床医护人员快速区分患者是否有发生营养不良的风险提供可靠的参考，并以此实施更有针对性的预防措施，增强对高龄、高合并症、意识障碍患者发生营养不良的预防意识，密切关注患者血红蛋白、总蛋白水平及中性粒细胞计数，积极治疗患者疾病，改善患者炎症状态，纠正贫血，补充优质蛋白，及时评估患者营养状况为其选择合适的营养支持方式，制订针对性、可预见性的营养支持方案。

综上所述，年龄、CCI、GCS 评分、贫血、血清总蛋



(C)1994-2023 China Academic Journal Electronic Publishing House. All rights reserved. http://www.cnki.net

白水平、中性粒细胞计数、营养支持方式是老年重症脑卒中患者营养不良的独立影响因素。本研究构建的列线图风险预测模型可有效预测老年重症脑卒中患者营养不良的发生，为临床医护人员早期筛查营养不良高危人群提供了高效、便捷的工具。但本研究为单中心、回顾性研究，未来可扩大样本量、开展多中心、前瞻性研究，进一步改进模型的预测效能和外推性，以期实现老年重症脑卒中患者营养不良的有效预防。

## [参考文献]


[1] 赵芸芸，曾维.老年脑卒中患者营养现状及营养不良的影响因素[J].中国老年学杂志，2016，36(10)：2372-2373.

[2] 朱群妹，朱珠，葛文静，等.区域医疗联合体中社区老年脑卒中病人吞咽障碍及营养状况调查[J].护理研究，2019，33(18)：3242-3245.

[3] Yuan K，Zhu S，Wang H，*et al*. Association between malnutrition and long-term mortality in older adults with ischemic stroke [J]. Clin Nutr，2021，40(5)：2535-2542.

[4] Yuan F，Yang F，Zhang W，*et al*. Optimizing early enteral nutrition in severe stroke (OPENS)：protocol for a multicentre randomized controlled trial [J]. BMC Neurol，2019，19(1)：24.

[5] 刘家硕，朴哲，杨瑞丽，等.三种营养风险筛查工具对老年脑卒中住院患者的筛查与比较[J].中国老年学杂志，2020，40(20)：4286-4288.

[6] 张新胜，马新安.老年脑卒中营养不良危险因素分析[J]蚌埠医学院学报，2017，42(7)：950-951.

[7] 中华医学会神经病学分会，中华医学会神经病学分会脑血管病学组，中国各类主要脑血管病诊断要点 2019[J].华神经科杂志，2019(9)：710-715.

[8] 陈洪波，张艳，宿英英.急性重症脑卒中患者脑心交互现象的临床特点与预后分析[J].中华危重病急救医学，2019(8)：953-957.

[9] Kasner SE. Clinical interpretation and use of stroke scales [J]. Lancet Neurol，2006，5(7)：603-612.

[10] Kwah LK，Diong J. National Institutes of Health Stroke Scale (NIHSS) [J]. J Physiother，2014，60(1)：61.

[11] 张霖，皮春梅，聂秀红.查尔森合并症指数对老年慢性阻塞性肺疾病患者预后的评估价值[J].中国呼吸与危重监护杂志，2016，15(4)：333-336.

[12] 赏石丽，孙紫娟，毕莉娜，等.糖尿病肾病患者发生贫血的影响因素研究[J].中国全科医学，2022，25(12)：1464-1469.

[13] 惠宁，张文杰.慢性心力衰竭患者营养不良风险预测模型的构建及验证[J].中华护理杂志，2021，56(3)：325-329.

[14] 任姗姗，杨子艳，李冠臻，等.老年脑卒中患者应用全球营养领导人发起的营养不良标准评价营养状态的研究[J].中华老年医学杂志，2022，41(3)：271-275.

[15] 王宇娇，黄迎春，高岚.重症营养风险评分表的应用进展[J].中华护理杂志，2017，52(5)：568-570.

[16] 翁敏，代正燕，甘志明，等.常见恶性肿瘤住院患者营养状况及影响因素分析[J].肿瘤代谢与营养电子杂志，2022，9(2)：195-199.

[17] Chen CT，Tung HH，Chen YC，*et al*. Depressive symptoms and nutritional status in the frail older adults [J]. Arch Gerontol Geriatr，2019，83：96-100.

[18] 陈华辉，张刚利，张豪，等.急性颅脑损伤患者早期预后相关因素分析[J].山西医科大学学报，2019，50(1)：112-116.

[19] 封靖，杜明艳，张瑞华，等.急性缺血性脑卒中合并低白蛋白血症临床分析[J].河北医药，2010，32(22)：3136-3137.

[20] 龙艳红，孙敏，杨晓琳，等.老年住院患者营养不良辅助诊断指标[J].中国老年学杂志，2020，40(19)：4139-4142.

[21] 林首武.急性缺血性脑卒中患者发生危重症性多发性神经病的危险因素及对预后的影响[J].中外医学研究，2022，20(11)：140-143.

[22] Frangos E，Trombetti A，Graf CE，*et al*. Malnutrition in Very Old Hospitalized Patients：A New Etiologic Factor of Anemia? [J]. J Nutr Health Aging，2016，20(7)：705-713.

[23] Deer RR，Volpi E. Protein Requirements in Critically Ill Older Adults [J]. Nutrients，2018，10(3)：378.

[24] Chen N，Li Y，Fang J，*et al*. Risk factors for malnutrition in stroke patients：A meta-analysis [J]. Clin Nutr，2019，38(1)：127-135.

[25] Lappas BM，Patel D，Kumpf V，*et al*. Parenteral Nutrition：Indications，Access，and Complications [J]. Gastroenterol Clin North Am，2018，47(1)：39-59.

[26] 唐倩芸，邢柏.预测 PICC 导管相关血流感染风险的列线图模型的建立与验证[J].中国医药导报，2020，17(36)：45-48.

[27] 张涵，段霞，庞启英，等.初产妇会阴裂伤风险列线图预测模型的建立与验证[J].中国医药导报，2022，19(27)：25-29.

[28] 李树雄，王秋梨.针刺良性阵发性位置性眩晕复位后残余症状疗效的危险因素分析及其相关列线图模型建立[J].中国医学创新，2021，18(29)：112-116.

[29] 李序薇，陈艳茹，陈燕.非小细胞肺癌化疗引起中性粒细胞减少性发热的列线图预测模型的构建[J].中国医药科学，2022，12(22)：8-11，53.


(收稿日期：2022-04-02)



(C)1994-2023 China Academic Journal Electronic Publishing House. All rights reserved. http://www.cnki.net
