# Supplementary material for: Development and external validation of machine learning models for the early prediction of malnutrition in critically ill patients: a prospective observational study
Source: BMC Med Inform Decis Mak. 2025 Jul 3;25:248. doi: 10.1186/s12911-025-03082-9 (PMC12225150; doi:10.1186/s12911-025-03082-9)
Supplement: Supplementary file 11 — Supplementary Material 11 [file 12911_2025_3082_MOESM11_ESM.pdf]

ICU危重患者营养不良的现状及影响因素

亢凤兵①

【摘要】 **目的**：探究ICU老年危重患者营养不良的现状及影响因素。**方法**：选取2016年9月-2019年3月在本院ICU进行治疗的178例危重患者，根据患者营养情况分为营养不良组（n=53）及对照组（n=125），比较两组一般资料、相应症状发生情况及急性生理学及慢性健康状况评分（APACHEⅡ）评分，对差异有统计学意义的因素进行Logistic回归分析，探究ICU危重患者营养不良的危险因素。**结果**：178例危重患者中，有53例患者发生营养不良，发生率为29.78%。Logistic回归分析结果显示，营养支持方式、消化道出血、去甲肾上腺素及镇静、检查或手术、腹腔高压、呕吐及腹泻症状、APACHEⅡ评分为ICU危重患者营养不良的危险因素（P<0.05）。**结论**：目前，ICU危重患者营养不良发生率仍较高，且影响因素主要包括营养支持方式、消化道出血、去甲肾上腺素、镇静、营养支持期间检查或手术、腹腔高压、伴有呕吐腹泻症状及APACHEⅡ评分。医护人员应对存在以上因素的患者采取强化干预措施，以降低营养不良的发生率。

【关键词】 ICU危重患者　营养不良　影响因素　APACHEⅡ评分

doi：10.14033/j.cnki.cfmr.2020.36.018　　文献标识码　B　　文章编号　1674-6805（2020）36-0047-03

Current Status and Influencing Factors of Malnutrition in ICU Critically Ill Patients/KANG Fengbing. //Chinese and Foreign Medical Research, 2020, 18(36): 47-49

[Abstract] **Objective:** To explore the current status and influencing factors of malnutrition in ICU critically ill patients. **Method:** From September 2016 to March 2019, 178 critically ill patients who were treated in the ICU of our hospital were selected. According to their nutritional status, they were divided into the malnutrition group (n=53) and the control group (n=125). The general data, symptom occurrence and acute physiology and chronic health evaluation Ⅱ (APACHE Ⅱ) score were compared between the two groups. Logistic regression analysis was performed for the factors with statistically significant differences to explore the risk factors of malnutrition in ICU critically ill patients. **Result:** Among 178 critically ill patients, malnutrition occurred in 53 patients, with an incidence of 29.78%. Logistic regression analysis results showed that nutritional support mode, gastrointestinal bleeding, norepinephrine and sedation, examination or surgery, intra-abdominal hypertension, vomiting and diarrhea, APACHE Ⅱ score were risk factors for malnutrition in ICU critically ill patients (P<0.05). **Conclusion:** At present, the incidence of malnutrition in critically ill ICU patients is still high, and the main factors include nutrition support mode, gastrointestinal bleeding, norepinephrine and sedation, examination or surgery during nutrition support, intra-abdominal hypertension, vomiting and diarrhea, APACHE Ⅱ score. Medical staff should take intensive intervention measures for patients with the above factors to reduce the incidence of malnutrition.

[Key words] ICU critical patients　Malnutrition　Influencing factors　APACHE Ⅱ score

First-author's address: Daxing District Hospital of Integrated Traditional Chinese and Western Medicine in Beijing, Beijing 100067, China

①北京市大兴区中西医结合医院　北京　100067

2013，33（4）：230-233.

[5] 牛凤环，王要军．食管内125I粒子带膜支架与普通支架治疗中晚期食管癌临床治疗效果对比[J].中华保健医学杂志，2010，12（3）：175-177.

[6] 王瑞刚，王贵齐．内镜黏膜下剥离术治疗早期食管癌的应用及进展[J].中国肿瘤，2018，27（1）：46-53.

[7] 任鹏，于振涛．晚期食管癌的姑息治疗[J].中国肿瘤临床，2013（10）：604-607.

[8] 汪亮．食管癌的免疫治疗[J].国际肿瘤学杂志，2017，44（5）：386-389.

[9] 李辉．现代食管外科学[M].北京：人民军医出版社，2004：331-332.

[10] Cox M，Eslick G，Nagaraja V.Safety and efficacy of esophageal stents preceding or during neoadjuvant chemotherapy for esophageal cancer：a systematic review and meta-analysis[J].J Gastrointest Oncol，2014，5（2）：119-126.

[11] 文卫锋，张建华，孔小锋，等．胃镜联合数字减影血管造影引导支架植入术治疗重度狭窄食管癌36例分析[J].实用医学影像杂志，2015，16（5）：7-9.

[12] 江振强，李咏松，邬艺忠，等．胃镜下食管支架置入术与透视下食管支架置入术的疗效对比[J].临床医学工程，2019，26（4）：27-28.

[13] Baerlocher M O，Asch M R，Vellahottam A，et al.Safety and efficacy of gastrointestinal stents in cancer patients at a community hospital[J].Canadian Journal of Surgery，2008，51（2）：130-134.

[14] Shinke T，Itoh T，Ishida M，et al.Early and mid-term vascular responses to optical coherence tomography guided everolimus-eluting stent implantation in stable coronary artery disease[J].Canadian Journal of Cardiology，2019，35（11）：1513-1522.

[15] 黄丽静．晚期食管癌食管支架置入术患者55例并发症分析[J].临床合理用药杂志，2018，11（5）：158-159.

[16] 文卫锋，张建华，孔小锋，等．胃镜联合数字减影血管造影引导支架植入术治疗重度狭窄食管癌36例分析[J].实用医学影像杂志，2015，16（5）：369-371.

[17] 唐富英，杨新魁，关权煊，等．内镜下植入食管支架治疗食管癌性狭窄36例报道[J].中国现代医生，2010，48（1）：24-25.

[18] 吴木军，张凡．食道支架置入在食管恶性梗阻的应用价值[J].现代消化及介入诊疗，2016，21（6）：828-830.

（收稿日期：2020-06-23）（本文编辑：马竹君）

(C)1994-2022 China Academic Journal Electronic Publishing House. All rights reserved. http://www.cnki.net

ICU 危重患者因伴有多器官功能障碍或衰竭等症状，导致机体处于高分解、高代谢状态，于晓帆等[1]指出，ICU 危重患者体重丢失速度可达 0.5~1.0 kg/d，进而引发营养不良，临床常规对该类患者实施营养支持，营养支持作为 ICU 患者常用营养干预手段，主要包括肠内营养及肠外营养两种方式[2]。前者通过管饲或口服营养剂，后者通过静脉输入脂肪乳剂、葡萄糖或氨基酸等物质，以达到满足患者营养需求的目的。但临床经验表明，由于 ICU 危重患者病情较为复杂，常导致患者发生营养不良等情况，影响了患者症状的康复[3]。为探究 ICU 危重患者营养不良现状，并进而分析其影响因素，本次研究选取 2016 年 9 月 -2019 年 3 月在本院 ICU 进行治疗的 178 例危重患者作为研究对象，现报告如下。

1 资料与方法

1.1 一般资料

选取 2016 年 9 月 -2019 年 3 月在本院 ICU 进行治疗的 178 例危重患者，其中男 85 例、女 93 例；年龄 50~81 岁，平均（65.25±4.57）岁；原发疾病：脑血管疾病 76 例，呼吸衰竭 34 例，胃肠道功能损伤 29 例，循环功能障碍 13 例，其他 26 例；营养支持方式：肠内营养素 54 例，肠外营养素 124 例。纳入标准：（1）在本院 ICU 接受治疗；（2）神志清楚，能够接受营养支持；（3）营养支持时间≥3 d。患者对本次研究知情，并自愿参与。

1.2 方法

患者进入 ICU 后，在对患者进行对症治疗的同时，根据患者具体情况实施营养支持，并在营养支持 3 d 后，记录患者身高、体重、血清白蛋白（ALB），并计算体重指数（BMI），根据 BMI 及 ALB 水平，判定患者营养情况。营养不良标准：BMI<18.5 kg/m^2 或 ALB<35 g/L，并根据营养情况分为营养不良组（n=53）及对照组（n=125）。

1.3 观察指标

采用本院自制的《ICU 重症患者营养支持记录表》记录性别、年龄，同时对营养支持方式、消化道出血、去甲肾上腺素及镇静、检查或手术情况、腹腔高压情况、伴有呕吐腹泻症状的情况进行记录，并进行急性生理学及慢性健康状况评分（APACHEⅡ）。

1.4 统计学处理

本研究数据采用 SPSS 18.0 统计学软件进行分析和处理，计量资料以（$\bar{x}\pm s$）表示，采用 t 检验，计数资料以率（%）表示，采用 χ^2 检验，采用 logistic 回归分析 ICU 危重患者营养不良的影响因素，P<0.05 为差异有统计学意义。

2 结果

2.1 ICU 危重患者营养不良发生情况

178 例危重患者中，有 53 例患者发生营养不良，发生率为 29.78%。

2.2 ICU 危重患者营养不良的单因素分析

两组营养支持方式、消化道出血、去甲肾上腺素、镇静、检查或手术情况、腹腔高压情况、伴有呕吐腹泻症状及 APACHEⅡ评分方面比较差异有统计学意义（P<0.05），见表 1。

表1 ICU危重患者营养不良的单因素分析 例（%）

组别	年龄		性别		营养支持方式	
	<65 岁	≥65 岁	男	女	肠内	肠外
营养不良组（n=53）	25（47.17）	28（52.83）	24（45.28）	29（54.72）	8（15.09）	45（84.91）
对照组（n=125）	56（44.80）	69（55.20）	61（48.80）	64（51.20）	46（36.80）	79（63.20）
χ^2 值	0.298		2.004		8.297	
P 值	0.192		0.059		0.003	

表1（续）

组别	消化道出血		去甲肾上腺素、镇静		检查或手术情况	
	是	否	是	否	是	否
营养不良组（n=53）	35（66.04）	18（33.96）	31（58.49）	22（41.51）	36（67.92）	17（32.08）
对照组（n=125）	31（24.80）	94（75.20）	28（22.40）	97（77.60）	42（33.60）	83（66.40）
χ^2 值	27.129		21.877		8.004	
P 值	0.000		0.000		0.003	

表1（续）

组别	腹腔高压		呕吐、腹泻症状		APACHEⅡ评分	
	是	否	是	否	<20 分	≥20 分
营养不良组（n=53）	40（75.47）	13（24.53）	37（69.81）	16（30.19）	19（35.85）	34（64.15）
对照组（n=125）	38（30.40）	87（69.60）	32（25.60）	93（74.40）	98（78.40）	27（21.60）
χ^2 值	5.394		6.330		9.004	
P 值	0.018		0.005		0.002	

2.3 ICU 危重患者营养不良的 Logistic 回归分析

Logistic 回归分析结果显示，营养支持方式、消化道出血、去甲肾上腺素及镇静、检查或手术、腹腔高压、呕吐及腹泻症状、APACHEⅡ评分均为 ICU 危重患者营养不良的危险因素（P<0.05），见表 2。

3 讨论

营养支持作为 ICU 危重患者常见干预措施，能够通过降低患者体内蛋白质等物质的消耗，避免患者因营养不良而出现风

(C)1994-2022 China Academic Journal Electronic Publishing House. All rights reserved. http://www.cnki.net

表2　ICU危重患者营养不良的Logistic回归分析

因素	β	SE	Wald	*P* 值	*OR* 值	95%*CI*
营养支持方式	1.162	0.425	3.473	0.002	3.125	1.153，7.225
消化道出血	0.917	0.416	4.256	0.004	2.819	1.214，6.984
去甲肾上腺素、镇静	0.792	0.316	6.318	0.007	2.482	1.016，6.192
检查或手术	0.612	0.297	4.752	0.003	1.497	1.022，2.927
腹腔高压	0.782	0.458	5.163	0.018	1.863	1.326，4.121
呕吐、腹泻症状	1.157	0.372	3.462	0.005	1.526	1.128，5.563
APACHEⅡ评分	0.124	0.418	4.281	0.002	3.253	1.452，7.314

险事件，同时能够有效维持患者机体脏器功能，达到降低死亡率的效果[4]。但由于危重患者病情较为容易复发，因此部分患者在进行营养支持后，营养供给程度仍难以满足机体需求，造成营养不良等不良情况的发生[5]。本次研究结果显示，178 例危重患者中，有 53 例患者发生营养不良，发生率为 29.78%。该结果表明，目前 ICU 危重患者发生营养不良概率仍较高，同时也说明了采取相应护理干预措施的重要性。

本次研究中，以患者营养不良为因变量，以相关因素为自变量进行 Logistic 回归分析，结果显示，营养支持方式、消化道出血、去甲肾上腺素及镇静、检查或手术、腹腔高压、呕吐及腹泻症状、APACHEⅡ评分均为 ICU 危重患者营养不良的危险因素（$P<0.05$），其原因包括：（1）肠外营养素，实施肠外营养素的患者通常病情更为严重，体重丢失速度更高，且部分患者因机体发生多器官功能衰竭等情况，无法完全吸收营养物质[6-7]。同时，肠外营养支持患者更易发生导管脱出或移位等情况，也影响了营养支持效果。（2）消化道出血，临床经验表明，ICU 危重患者常伴有一定程度的凝血功能异常，加之部分患者本身即伴有消化道溃疡等症状，因此极易导致消化道出血等情况的发生，而消化道出血则可严重影响患者胃肠道原有功能，影响营养物质的吸收，进而导致营养不良的发生[8]。（3）使用去甲肾上腺素作为 ICU 常用急救药物，能够改善感染性休克等危重症，但该药物的使用可导致心、肾等脏器因毛细血管吸收不良而出现功能障碍，同时患者胃肠道对营养物质的吸收能力也将受到严重影响。（4）营养支持期间进行检查或手术，临床经验表明，多数 ICU 危重患者需外出接受造影或 CT 等检查，部分患者还需进行手术治疗，此时，患者需中断营养支持，进而影响营养素的供给[9]。而当患者回到 ICU 后，医护人员通常按照原有剂量继续进行营养支持，因此难以满足患者营养需求，尤其对于进行手术的患者，因术前患者需进行胃肠准备，因此对营养支持患者影响较大。（5）腹腔高压及呕吐、腹泻症状，吴栋等[10]指出腹腔高压是导致患者营养不良的主要因素，其原因主要为，肠腔内积聚的气体及液体可导致腹内压升高，而肠道因对腹内压升高较为敏感，因此可导致肠管及肠壁血管受压，促使肠壁出现缺血症状，导致肠道蠕动减弱，另外，腹腔高压还可导致胃肠道绒毛发生萎缩或断裂，进而影响肠道功能，而当患者出现呕吐及腹泻等症状时，可导致机体营养水平进一步降低，患儿因营养需求加大而出现营养不良[11]。（6）APACHEⅡ评分，孙丽娟等[12]指出，APACHEⅡ评分是导致喂养不足的危险因素，且随着评分的提高，患者营养耐受性逐渐降低，主要原因包括 APACHEⅡ评分较高的患者病情更为严重，且常伴有糖尿病、高血压等症状，影响了胃肠功能，同时，该类患者因常使用广谱抗生素，导致机体内有益菌生存环境受到破坏，导致机体内菌群失调，并使肠道天然生物屏障受到破坏，影响了营养素的吸收效果。

综上所述，目前，ICU 危重患者发生营养不良概率仍较高，且影响因素主要包括营养支持方式、消化道出血、去甲肾上腺素、镇静、检查或手术情况、腹腔高压情况、伴有呕吐腹泻症状情况、APACHEⅡ评分，医护人员应对存在以上因素的患者采取强化干预措施，以降低营养不良的发生率。

参考文献

[1] 于晓帆，万晓红，万林骏，等 . 脓毒症患者 ICU 获得性肌无力的高危因素分析 [J]. 中华危重病急救医学，2018，30（4）：355-359.

[2] 中华医学会肠外肠内营养学分会 . 成人围手术期营养支持指南 [J]. 中华外科杂志，2016，54（9）：641-657.

[3] 朱革珍 . 持续肠内营养法在 ICU 危重症患者中的应用 [J]. 实用临床医药杂志，2017，21（14）：171-172.

[4] 王颖，刘世炎 . 早期营养支持降低 ICU 肿瘤重症患者呼吸机相关性肺炎的临床效果 [J]. 现代肿瘤医学，2016，24（2）：120-122.

[5] 王燕娟 .COPD 老年患者急性加重期营养不良的危险因素分析 [J]. 中国急救复苏与灾害医学杂志，2019，14（5）：495-497.

[6] 孙英群，宋云川，陈效曦，等 .ICU 老年患者术后精神障碍的发病率及相关因素分析 [J]. 国际精神病学杂志，2018，45（2）：146-148，164.

[7] 麦燕婷，郭泽霞，王佳月 .1054 例 ICU 患者器械性压疮的调查及对策分析 [J]. 西南国防医药，2018，28（5）：500-502.

[8] 安永莲 . 碳酸氢钠溶液联合 0.9% 氯化钠注射液预防鼻空肠营养管堵塞的效果观察 [J]. 蚌埠医学院学报，2017，42（2）：275-276.

[9] 卓剑，李洁 . 不同营养支持途径对 ICU 急性出血性胰腺炎患者营养状况及免疫功能的影响 [J]. 临床和实验医学杂志，2016，15（2）：79-81.

[10] 吴栋，展翰翔，王磊，等 . 微创与加速康复外科理念下胰腺癌围手术期的营养管理 [J]. 腹腔镜外科杂志，2019，24（9）：717-720.

[11] 闫晓红，孙萱，张杰 . 营养支持对白血病化疗患者营养状况及不良反应的影响 [J]. 中国食物与营养，2018，24（9）：72-74.

[12] 孙丽娟，薛森海，闫凤，等 . 预消化的肠内营养对 ICU 危重症患者的营养状况及肠内营养耐受性的影响 [J]. 现代生物医学进展，2019，19（10）：1883-1888.

（收稿日期：2020-06-23）（本文编辑：薛琦琪）

(C)1994-2022 China Academic Journal Electronic Publishing House. All rights reserved.　http://www.cnki.net
