# Supplementary material for: Development and external validation of machine learning models for the early prediction of malnutrition in critically ill patients: a prospective observational study
Source: BMC Med Inform Decis Mak. 2025 Jul 3;25:248. doi: 10.1186/s12911-025-03082-9 (PMC12225150; doi:10.1186/s12911-025-03082-9)
Supplement: Supplementary file 12 — Supplementary Material 12 [file 12911_2025_3082_MOESM12_ESM.pdf]

临床护理·肿瘤科护理

ICU 恶性肿瘤患者早期肠内营养达标情况及影响因素调查

李佳芮 覃惠英 马盈盈 孙仲文 邹秀梅 杜慧

【摘要】 **目的** 探讨ICU恶性肿瘤患者早期肠内营养达标情况及影响因素。**方法** 采用回顾性调查方法,运用自行设计的调查表,共纳入161例患者。**结果** ICU恶性肿瘤患者早期肠内营养达标率为57.14%,不达标的独立危险因素包括合并症($P<0.05$, OR=2.312, OR 95%CI 1.078~4.958)、急性生理与慢性健康评分(APACHEⅡ)($P<0.05$, OR=1.111, OR 95%CI 1.017~1.214)、低白蛋白血症($P<0.05$, OR=2.252, OR 95%CI 1.036~4.892),既往肠内营养经历为保护性因素($P<0.01$, OR=0.123, OR 95%CI 0.021~0.494)。达标对患者近期结局无显著影响,但达标组白蛋白水平增高及APACHEⅡ评分改善更为显著,同时ICU住院时间更短。**结论** ICU恶性肿瘤患者早期肠内营养达标率较低,影响因素众多,同时对患者的疾病恢复有利,需进行综合干预以提高早期肠内营养有效性。

【关键词】 护理; 重症监护病房; 早期肠内营养; 耐受性

Adequate feeding of early enteral nutrition in critical patients with cancer *Li Jiarui, Qin Huiying*, Ma Yingying, Sun Zhongwen, Zou Xiumei, Du Hui. *Department of Nursing, Sun Yat-sen University Cancer Hospital, Guangzhou 510060, China*

Corresponding author: Qin Huiying, *Email*: qinhy@sysucc.org.cn

[Abstract] **Objective** To describe the adequate feeding of early enteral nutrition in critical patients with cancer, and explore its influencing factors. **Methods** A retrospective analysis study was performed and 161 patients were involved. **Results** Nearly 57.14% patients got adequate feeding. The independent risk factors included complications ($P<0.05$, OR=2.312, OR 95%CI 1.078-4.958), Acute Physiology and Chronic Health Evaluation Ⅱ (APACHE Ⅱ) scores ($P<0.05$, OR=1.111, OR 95% CI 1.017-1.214) and hypoalbuminemia ($P<0.05$, OR=2.252, OR 95%CI 1.036-4.892), and the protective factor was previous enteral nutrition experience ($P<0.01$, OR=0.123, OR 95%CI 0.021-0.494). Patients who got adequate feeding showed more increase in albumin levels, more decrease in APACHE Ⅱ scores and shorter ICU stays. **Conclusions** Part of patients got inadequate feeding, due to comprehensive effects of many factors. And adequate feeding may be beneficial to the increase of albumin levels, also helps patients rehabilitate early and shorten the ICU stays.

[Key words] Nursing care; Intensive care units; Early enteral nutrition; Tolerance

ICU恶性肿瘤患者是营养不良的高发人群[1],对营养支持的需求迫切。早期肠内营养(early enteral nutrition, EEN)作为一种更加贴合人体正常生理情况的营养支持措施,具有改善患者营养状态、减少并发症、促进疾病恢复等作用[2-3],受到广泛的认可。但ICU患者肠道耐受性较差[4],EEN喂养量不足的发生率较高[5],导致肠内营养的有效性降低。本研究通过调查EEN达标现状,探讨其影响因素,为进一步提高EEN有效性提供参考和依据,现报道如下。

对象与方法

1. 研究对象。本研究回顾性调查了2012年1月至2014年12月在中山大学肿瘤防治中心ICU住院并进行早期肠内营养的恶性肿瘤患者。纳入标准:年龄18岁及以上,明确诊断为恶性肿瘤,且在进入ICU后48 h内开始进行肠内营养者。排除标准:(1)在肠内营养开始后3 d内由于转科、出院、再手术、死亡而不能继续接受肠内营养者;(2)肠内营养开始前存在胃高残余量、呕吐、腹泻、腹胀、消化道出血、消化道瘘;(3)病历资料不全者。本研究行多因素回

DOI:10.3760/cma.j.issn.1672-7088.2017.03.010

作者单位:510060 广州,中山大学附属肿瘤医院重症医学科(李佳芮、孙仲文、邹秀梅、杜慧),护理部(覃惠英);中山大学附属第三医院外科重症监护室(马盈盈)

通信作者:覃惠英,Email:qinhy@sysucc.org.cn

Q2xvdWRWZXJzaW9uLQo?

归分析，探索各因素对 ICU 恶性肿瘤患者早期肠内营养达标情况的影响。根据一般规则，样本量数量是自变量的 5～10 倍，本研究中共有 11 项变量作为自变量进行 Logistic 回归分析，所需最小样本量 n=5×11=55 例，本研究共纳入 161 例患者，样本量符合要求。

2. 肠内营养方法。患者进入 ICU 后由重症医学科及临床营养科医生共同会诊后，根据患者情况决定肠内营养开始时间、方式。每日肠内营养摄入量及配方由临床营养科医生制订，并由临床营养科护士统一配制。对所有患者统一采取肠内营养泵连续泵入，并根据患者耐受情况调节速度。

3. 判定标准。(1)早期肠内营养：指患者进入 ICU 48 h 内开始进行的肠内营养。(2)目标量：采用 Harris-Benedict 公式计算[6]，男性目标量(kcal)= 66.5 + 13.75×W + 5.003×H − 6.775×A，女性目标量(kcal)= 655.1 + 9.563×W +1.85×H − 4.676×A，其中：W 为体质量(kg)，H 为身高(cm)，A 为年龄(岁)。(3)肠内营养达标：患者肠内营养第 3 天肠内营养摄入能量达到目标量的 60%[3,7]。

4. 测量工具。本研究采用自行设计的 ICU 恶性肿瘤患者营养支持情况调查表进行资料收集，包括基本疾病资料、肠内营养资料及近期预后资料。

5. 数据处理。运用 SPSS 19.0 软件对资料进行统计分析。使用平均数及标准差、频次加构成比对数据进行描述。单因素分析采取 t 检验、秩和检验或 χ^2 检验。采用多因素 Logistic 回归分析影响达标的独立因素，检验水准 α = 0.05。

结　果

1. 患者一般资料及肠内营养资料。本研究中患者以男性为主，年龄分布在 28～90 岁，年龄(60.77 ± 11.30)岁，中老年居多，体质量指数(body mass index，BMI)采用中国标准[7]，消瘦患者(BMI < 18.5 kg/m²)共 21 例，正常患者(BMI 18.5～23.9 kg/m²)共 93 例，超重患者(BMI≥24.0 kg/m²)共 47 例。77.02%(124/161)的患者均为术后患者，入 ICU 后 24 h 内急性生理学与慢性健康状况评估Ⅱ(Acute Physiology and Chronic Health Evaluation Ⅱ，APACHE Ⅱ)为(13.15 ± 4.80)分。患者入 ICU 后开始肠内营养时间为(18.55 ± 17.27) h，54.04%的患者均采用了幽门后喂养。患者一般资料见表 1。

2. 患者 EEN 达标情况。161 例患者中共有 92 例患者早期肠内营养达标，未达标 69 例，达标率为 57.14%。共有 23 例患者占 14.29%在肠内营养第 3 天肠内营养摄入能量达 100%目标值。共有 10 例患者占 6.21%发生了肠内营养中断。

3. EEN 达标的影响因素。(1)单因素分析。将 161 例患者分为达标组及未达标组，经单因素分析，2 组患者在低蛋白血症、APACHE Ⅱ、合并症、化疗史、肠内营养经历、营养制剂、纤维配方、抗生素联合使用、机械通气治疗、镇静镇痛及血管活性药物使用方面的差异有统计学意义(P < 0.05)，见表 2。(2)多因素分析。以单因素分析中有统计学意义的 11 项因素为自变量，以是否达标为应变量($y_{达标}$=0，$y_{不达标}$=1)，进行 Logistic 回归分析。得到 4 个独立因素，合并症、低蛋白血症及 APACHE Ⅱ 得分的增高为不达标的危险因素，入 ICU 前肠内营养经历为达标的保护性因素，见表 3。

4. EEN 达标对预后的影响。是否达标的患者近期临床结局差异无统计学意义。EEN 达标组患者在 ICU 住院期间白蛋白水平的增长更为显著，APACHE Ⅱ 改善较为明显，同时 ICU 住院时间更短，见表 4。

表 1　161 例早期肠内营养患者一般资料

项目	例数(%)	项目	例数(%)
性别		诊断	
男	126(78.26)	食管癌	89(55.28)
女	35(21.74)	肺癌	21(13.04)
年龄(岁)		淋巴瘤	12(7.45)
28～44	11(6.83)	甲状腺癌	12(7.45)
45～59	51(31.68)	鼻咽癌	6(3.70)
60～90	99(61.49)	肠癌	4(2.49)
体质量指数(kg/m²)		其他	17(10.56)
消瘦(<18.5)	21(13.04)	化疗史	
正常(18.5～23.9)	93(57.76)	无	115(71.43)
超重(≥24.0)	47(29.19)	有	46(28.57)
是否手术		放疗史	
否	37(22.98)	无	140(86.96)
是	124(77.02)	有	21(13.04)

讨　论

1. ICU 恶性肿瘤患者 EEN 达标现状。本研究中共有 92 例患者早期肠内营养达标，占 57.14%。与既往 ICU 患者研究中的达标率较为一致[8-9]。仍然提示存在较多患者肠内营养的喂养量不足，ICU 恶性肿瘤患者的肠内营养质量有待提高。护士一方面应当注重对肠内营养喂养量的提升，另一方面需要加强对肠内营养不良反应的预防，避免因不良反应造成的喂养减少或中断。

2. ICU 恶性肿瘤患者 EEN 达标的影响因素。(1)患者的疾病及治疗因素。患者入 ICU 时的疾病资料中，APACHE Ⅱ 得分越高反映病情越重，得分每增加 1 分不达标的风险增加 1.111 倍，存在合并症的患者不达标风险也相应增高。提示肠道功能和耐受性受到整体病情的影响，与徐文秀等[6]的结果一致。由于危重患者机体处于应激状态，血流动力学不稳定，为保持心肺脑等重要器官的血流供应，往往导致肠血流急剧减少，肠功能受到损害[10-11]。并且危重患者由于乏力、昏迷、镇静等因素，长期限制卧床，活动减少，肠蠕动减慢，均可能增加腹胀、胃潴留、消化不良等症状的发生，导致肠内营养的减缓甚至暂停，喂养量的增加因此受到干扰。因此促进基础病情的改善是肠内营养顺利进行的重要保障。同时，低白蛋白血症也是不达标的危险因素。ICU 患者机体处于应激状态，分解代谢增强，是低白蛋白血症的高发人群。而低白蛋白血症患者容易发生肠黏膜水肿，破坏肠黏膜功能，对 EEN 耐受性较差。既往研究已显示存在低白蛋白血症的患者肠内营养不良反应较多[12-13]，不利于喂养量的

Q2xvdWRWZXJzaW9uLQo?

表 2　早期肠内营养患者一般资料及肠内营养资料单因素分析

组别	例数	性别[例(%)] 男	女	年龄[例(%)] 26～45岁	45～60岁	60～90岁	体质量指数[例(%)] 消瘦(< 18.5 kg/m²)	正常(18.5～23.9 kg/m²)	超重(≥24.0 kg/m²)	是否手术[例(%)] 否	是
未达标组	69	56(44.4)	13(37.1)	7(63.6)	19(37.2)	43(43.4)	10(47.6)	36(38.7)	23(44.9)	21(56.8)	48(38.7)
达标组	92	70(55.6)	22(62.9)	4(36.4)	32(62.8)	56(56.6)	11(52.4)	57(61.3)	24(51.1)	16(43.2)	76(61.3)
Z/χ^2 值		0.596		−1.112			1.557			3.790	
P 值		0.563		0.911			0.460			0.060	

组别	APACHE Ⅱ (分, $\bar{x} \pm s$)	低白蛋白血症[例(%)] 无	有	贫血[例(%)] 无	有	化疗史[例(%)] 无	有	放疗史[例(%)] 无	有
未达标组	11.78 ± 3.80	34(32.7)	33(62.3)	17(37.0)	52(45.6)	41(35.7)	28(60.9)	59(42.1)	10(47.6)
达标组	14.96 ± 5.40	70(67.3)	20(37.7)	29(63.0)	62(54.4)	74(64.3)	18(39.1)	81(57.9)	11(52.4)
Z/χ^2 值	3.794	12.550		1.002		8.532		0.224	
P 值	< 0.001	0.001		0.379		0.005		0.645	

组别	合并症[例(%)] 无	有	幽门后喂养[例(%)] 否	是	肠内营养经历[例(%)] 无	有	营养制剂[例(%)] 整蛋白型	非整蛋白型	纤维配方[例(%)] 否	是
不达标组	22(30.6)	47(52.8)	37(50.0)	55(63.2)	65(47.5)	4(16.7)	62(40.8)	7(77.8)	8(80.0)	61(40.4)
达标组	50(69.4)	42(47.2)	37(50.0)	32(36.8)	72(52.5)	20(83.3)	90(59.2)	2(22.2)	2(20.0)	90(59.6)
χ^2 值	8.048		2.853		7.900		4.747		6.006	
P 值	0.006		0.111		0.006		0.039		0.020	

组别	机械通气[例(%)] 否	是	镇静镇痛[例(%)] 无	有	血管活性药[例(%)] 无	有	抗生素联合应用[例(%)] 无	有	消化道用药[例(%)] 无	有
不达标组	14(27.5)	55(50.0)	3(10.0)	66(50.4)	3(7.1)	66(55.5)	29(33.0)	40(54.8)	5(71.4)	64(41.6)
达标组	37(72.5)	55(50.0)	27(90.0)	65(49.6)	39(92.9)	53(44.5)	59(67.0)	33(45.2)	2(28.6)	90(58.4)
χ^2 值	7.234		16.254		29.596		7.771		2.439	
P 值	0.010		0.000		0.000		0.007		0.139	

注：APACHE Ⅱ：急性生理学与慢性健康状况评估

增加。故对于存在低白蛋白血症的患者，应及早予以纠正。另外，在单因素分析中，既往具有化疗史的患者达标率较低，可能与肠道曾受化疗药损害有关。(2)肠内营养因素。入 ICU 前有肠内营养经历，有助于 EEN 达标。一方面是由于这类患者没有经历禁食给肠道功能带来的损伤[13-14]；另一方面也体现了肠内营养对肠道的维护作用，能较快实现喂养量的增加。研究显示，肠内营养可明显改善肠道的局部血供[15]，促进肠上皮细胞的生长、修复，有助于维持肠道功能[16]。故在临床工作中应尽量避免不必要的禁食，早期开展肠内营养。

肠内营养的实施过程中，营养制剂的选择可能会对患者存在影响，应当由临床营养师、重症科医生及护士等专业人员结合患者病情进行选择。使用纤维制剂的患者达标率较高，可能与纤维制剂对肠道功能的维护有关。

肠内营养期间的治疗情况也会对达标产生影响，抗生素联合应用的患者达标率也较低。机械通气患者机体可能存在缺氧情况，影响肠道供氧，且常伴有镇静镇痛使用，镇静镇痛药物可能对肠道功能造成干扰作用，应加强对此类患者的观察和护理，实行合理镇静镇痛治疗，实行每日唤醒等措施以缩短总镇静时间，可减少对肠内营养的影响。同时，血管活性药物的使用反映出患者存在血流动力学不稳定的状态，对肠道供血会产生影响[11]，同样不利于肠内营养的进行及喂养量的增加，应对这类患者进行严格的液体管理，尤其是血压的控制，避免肠道低灌注的发生。

表 3　161 例患者早期肠内营养达标 Logistic 回归分析（逐步前进法）

项目	*B* 值	*P* 值	*OR* 值	95% *CI*
合并症	0.838	0.031	2.312	1.078～4.958
低白蛋白血症	0.812	0.040	2.252	1.036～4.892
APACHE Ⅱ 评分	0.106	0.020	1.111	1.017～1.214
肠内营养经历	−2.098	0.003	0.123	0.021～0.494

注：APACHE Ⅱ：急性生理学与慢性健康状况评估

3. EEN 达标对患者预后的影响。在本研究中，早期肠内营养达标对患者的近期结局(包括转出、死亡、出院等)没有明显影响(χ^2=1.388，*P*=0.500)，与既往研究中早期肠内营养达标有助于减低患者死亡率[17]的结果不一致。分析其原因，可能为本研究中患者 ICU 住院时间较短，营养改善对预后的影响尚未体现。而且就肿瘤患者而言，由于受疾病的长期消耗，并受到抗肿瘤治疗的影响，尤其是在肿瘤晚期患者中，恶液质及多器官衰竭多见，机体的防御能力和恢复能力严重受损，营养改善对预后的影响作用可能较为有限，故

中华医学会杂志社
Q2xvdWRWZXJzaW9uLQo7

表 4　肠内营养达标对近期结局的影响

组别	例数	近期结局[例(%)]			白蛋白水平	APACHEⅡ评分	ICU 住院时间	总住院时间	机械通气时间
		转科	死亡	出院	(g/L, $\bar{x}\pm s$)	(分, $\bar{x}\pm s$)	(d, $\bar{x}\pm s$)	(d, $\bar{x}\pm s$)	(h, $\bar{x}\pm s$)
达标组	92	72(78.3)	14(15.2)	6(6.5)	4.55 ± 5.25	−4.01 ± 3.40	8.62 ± 5.74	33.46 ± 18.03	177.46 ± 168.84
不达标组	69	50(72.5)	12(17.4)	7(10.1)	2.20 ± 4.53	−2.81 ± 2.89	11.59 ± 8.75	34.21 ± 18.28	235.74 ± 219.99
$\chi^2/Z/t$ 值			2.110		2.973	−3.109	−2.020	−0.181	−1.331
P 值			0.348		0.004	0.002	0.043	0.857	0.183

注:APACHEⅡ:急性生理学与慢性健康状况评估

EEN 达标对患者结局的影响仍有待进一步研究。

早期肠内营养是帮助患者病情改善的有效措施[18],而本研究中发现,在进行早期肠内营养的患者,喂养量达标患者的疾病恢复可能存在更大的优势。本研究中达标患者白蛋白水平增高更为明显,提示 EEN 达标更有助于营养改善。同时,达标患者 APACHEⅡ得分的改善较不达标患者更为显著,ICU 住院时间相对更短,提示有质量的肠内营养更有利于患者病情的改善的速度和程度,肠内营养的有效性是其发挥作用的重要保障。目前早期肠内营养的应用已广受认可[19-20],在护理工作中更应当注重喂养量的增加,不断提高早期肠内营养的质量。

参 考 文 献

[1] 沈珏,赵擎宇,顾葆春,等. 肿瘤重症患者的营养状况评估及营养支持现状分析[J]. 热带医学杂志,2014(1):52-55.

[2] Chourdakis M,Kraus MM,Tzellos T,et al. Effect of early compared with delayed enteral nutrition on endocrine function in patients with traumatic brain injury: an open-labeled randomized trial[J]. J Parenter Enteral Nutr,2012,36(1):108-116. DOI:10.1177/0148607110397878.

[3] Heidegger CP,Darmon P,Pichard C. Enteral vs. parenteral nutrition for the critically ill patient:a combined support should be preferred[J]. Curr Opin Crit Care,2008,14(4):408-414.DOI:10.1097/MCC.0b013e3283052cdd.

[4] Blaser AR,Starkopf J,Kirsimagi U,et al. Definition, prevalence, and outcome of feeding intolerance in intensive care:a systematic review and meta-analysis[J]. Acta Anaesthesiol Scand, 2014,58(8):914-922. DOI:10.1111/aas.12302.

[5] Kim H,Shin JA,Shin JY,et al. Adequacy of nutritional support and reasons for underfeeding in neurosurgical intensive care unit patients[J]. Asian Nurs Res (Korean Soc Nurs Sci),2010,4(2):102-110. DOI:10.1016/S1976-1317(10)60010-2.

[6] 焦广宇,蒋卓勤. 临床营养学[M]. 3 版. 北京:人民卫生出版社,2010:90.

[7] 徐文秀,方理超,刘励军. 影响危重症患者早期肠内营养达标的因素[J]. 中国急救医学, 2010,30(6):502-504. DOI:10.3969/j.issn.1002-1949.2010.06.007.

[8] 方理超,徐文秀,刘励军. APACHE Ⅱ评分在危重症患者早期肠内营养中的应用[J]. 中国血液流变学杂志,2010,20(1):108-110;123. DOI:10.3969/j.issn.1009-881X.2010.01.038.

[9] 高宝祥. 不同应激程度危重症患者早期肠内营养达标率差异的研究[D]. 苏州:苏州大学,2013.

[10] 夏斌,靳风烁,胡森,等. 肠缺血-再灌流对早期肠内营养肠耐受性的影响[J]. 第三军医大学学报,2005,27(10):1016-1019. DOI:10.3321/j.issn.1000-5404.2005.10.026.

[11] Berger MM,Revelly JP,Cayeux MC,et al. Enteral nutrition in critically ill patients with severe hemodynamic failure after cardiopulmonary bypass[J]. Clin Nutr,2005,24(1):124- 132. DOI:10.1016/j.clnu.2004.08.005.

[12] 李为明,徐鹏远,岑云云,等. 手术后经空肠造口管早期肠内营养病人的耐受性分析[J]. 肠外与肠内营养,2009,16(2):90-92. DOI:10.3969/j.issn.1007-810X.2009.02.008.

[13] 蒋洋洋,许勤,宋燕波,等. ICU 患者肠内营养耐受性分析及护理对策[J]. 中国实用护理杂志,2011,27(2):17-19.DOI: 10.3760/cma.j.issn.1672-7088.2011.02.042.

[14] Kim H,Stotts NA,Froelicher ES,et al. Why patients in critical care do not receive adequate enteral nutrition? A review of the literature[J]. J Crit Care,2012,27(6):702-713. DOI:10.1016/j.jcrc.2012.07.019.

[15] Gatt M,Macfie J,Anderson AD,et al. Changes in superior mesenteric artery blood flow after oral, enteral, and parenteral feeding in humans[J]. Crit Care Med,2009,37(1):171- 176. DOI:10.1097/CCM.0b013e318192fb44.

[16] 海波,王昆华. 肠屏障功能的破坏与防护[J]. 中国医药指南,2012,10(4):65-67. DOI:10.3969/j.issn.1671-8194.2012.04.038.

[17] Chiang YH,Chao DP,Chu SF,et al. Early enteral nutrition and clinical outcomes of severe traumatic brain injury patients in acute stage: a multi-center cohort study[J]. J Neurotrauma,2012,29(1):75-80. DOI:10.1089/neu.2011.1801.

[18] 曾丽萍,何清,叶华,等. 术后脓毒症患者早期肠内营养的应用[J]. 岭南现代临床外科, 2012,12(4):338-340.DOI:10.3969/j.issn.1009-976X.2012.04.007.

[19] 中华医学会重症医学分会. 危重病人营养支持指导意见(2006)[J]. 中国实用外科杂志,2006,26(10):721- 732.DOI:10.3321/j.issn.1005-2208.2006.10.001.

[20] Mcclave SA,Martindale RG,Vanek VW,et al. Guidelines for the Provision and Assessment of Nutrition Support Therapy in the Adult Critically Ill Patient: Society of Critical Care Medicine (SCCM) and American Society for Parenteral and Enteral Nutrition (A.S.P.E.N.) [J]. JPEN J Parenter Enteral Nutr,2009,33(3):277-316. DOI:10.1177/0148607109335234.

(收稿日期:2016-02-29)

(本文编辑:梁秀凤)

Q2xvdWRWZXJzaW9uLQo?
