# Supplementary material for: Development and external validation of machine learning models for the early prediction of malnutrition in critically ill patients: a prospective observational study
Source: BMC Med Inform Decis Mak. 2025 Jul 3;25:248. doi: 10.1186/s12911-025-03082-9 (PMC12225150; doi:10.1186/s12911-025-03082-9)
Supplement: Supplementary file 13 — Supplementary Material 13 [file 12911_2025_3082_MOESM13_ESM.pdf]

论　著

影响危重症患者早期肠内营养达标的因素

徐文秀，　方理超，　刘励军

作者单位：215004　江苏，苏州大学附属第二人民医院综合重症监护科
作者简介：徐文秀（1981 -），男，在职研究生，住院医师，现在苏州市中医医院综合重症监护科工作。
通讯作者：刘励军（1963 -），男，主任医师，教授，硕士研究生导师，急诊科及综合重症监护科主任。

［**摘要**］　**目的**　试图阐明影响危重症患者早期肠内营养达标的主要因素。**方法**　根据患者是否给予早期肠内营养等入选标准，回顾性分析近 18 个月收住重症监护病房（ICU）治疗的患者 192 例。依据第 3 天肠内营养供给能量是否达到目标营养供给量［25 kcal/（kg · d）］的 60%，分为达标组和未达标组。分析比较两组患者的临床特征、实验室检查和治疗药物。通过 Logistic 回归分析，阐明主要影响早期肠内营养达标的因素。**结果**　两组胃肠道手术、儿茶酚胺类药物、镇静镇痛药物、肾脏衰竭、肠内营养后腹胀、重症胰腺炎及急性生理和慢性健康状况评分Ⅱ（APACHEⅡ）等指标比较差异有统计学意义（P 均 < 0.05）。除重症胰腺炎外，将上述因素导入 Logistic 回归分析，显示胃肠道手术和儿茶酚胺类药物的使用对肠内营养达标具有明显的影响（P 均 < 0.05），其 OR 值分别是 7.094（95% CI 1.841 ~ 27.332）和 3.076（95% CI 1.295 ~ 7.302）。**结论**　除重症胰腺炎患者外，胃肠道完整性的破坏和血流动力学的障碍是影响早期肠内营养达标的因素。

［**关键词**］　危重症；　早期肠内营养；　Logistic 回归分析
doi：10.3969/j.issn.1002 - 1949.2010.06.007

Logistic regression analysis of the factors affecting the early enteral nutrition up to goal in critically ill patients　*XU Wen - xiu, FANG Li - chao, LIU Li - jun. ICU, the Second People's Hospital, Suzhou University, Suzhou* 215004, *China*

［**Abstract**］　**Objective**　To determine the major factors affecting the early enteral nutrition (EEN) up to goal in critically ill patients. **Methods**　During the past 18 months in our ICU, 192 critically ill patients who met the criteria of enrollment, were included in this study. Depending on whether enteral nutrition supply was reach to 60% of nutrition goal ［25 kcal/(kg · d)］ at the third day, the patients were divided into two groups: up to goal and not up to goal. The clinical characteristics, laboratory discoveries and all kinds of interventions were compared in the two groups, and then were analysed by means of Logistic regression analysis in order to clarify the main influencing factors of early enteral nutrition up to goal. **Results**　Gastrointestinal tract surgery, catecholamine, sedation and opioid, renal dysfunction, feed intolerance, severe acute pancreatitis (SAP) and APACHE Ⅱ score were significantly different between the two groups ($P < 0.05$). All of them were included in the Logistic regression analysis excepting for SAP. The results of Logistic regression analysis showed the gastrointestinal tract surgery and catecholamine had affected early enteral nutrition up to goal ($P < 0.05$). The OR values were 7.094 (95% CI 1.841 ~ 27.332) and 3.076 (95% CI 1.295 ~ 7.302) respectively. **Conclusion**　Except SAP, the main factors affecting the early enteral nutrition up to goal were the damage of gastrointestinal tract and the hemodynamic instability.

［**Key words**］　Critical illness;　Early enteral nutrition (EEN);　Logistic regression analysis

营养支持是危重症患者多器官支持的重要措施之一。临床研究证实，早期实施肠内营养能减少危重症患者感染并发症的发生，并缩短住院时间和降低治疗费用等[1]。早期肠内营养支持已得到广泛

的重视,被认为是首要考虑的营养支持方式。然而,实际应用中仅30% ~50%的ICU患者能在早期通过肠内营养支持达到目标量[2,3]。营养支持不足可导致患者感染几率增加,伤口愈合减慢等不良预后[4]。故此,本研究试图采用Logistic回归分析方法,探讨影响早期肠内营养达标的主要因素,为提高肠内营养达标率提供临床资料。

1 资料与方法

1.1 研究对象和分组 本研究回顾性分析2008-01-01~2009-06-30入住我院ICU的患者。纳入标准:①年龄≥18岁;②住院时间≥72 h;③入住ICU 48 h内予以肠内营养。入选患者共192例,其中男性126例,女性66例。原发疾病包括多发伤82例,慢性肺部疾病急性发作24例,外科择期手术后严重并发症18例,糖尿病伴严重并发症4例,急性中毒12例,重症胰腺炎12例,恶性心律失常6例,多器官功能障碍综合征22例,其他原因12例。依据肠内营养第3天供给能量是否达到目标营养供给量[25 kcal/(kg·d)]的60%,将患者分为达标组和未达标组[5]。192例危重症患者中,62例(32.3%)达到早期肠内营养标准,其余130例未达标。

1.2 肠内营养方法 选择肠内营养的时机和方案:①选择进入ICU后24~48 h,血流动力学相对稳定,无明显肠道缺血及梗阻症状,4 h胃液潴留量<200 mL的患者。②肠内营养序贯原则:先游离氨基酸或短肽型配方,后整蛋白型配方;先增量,后增浓度,由肠内营养泵控制。③以间断连续性(连续输注16~20 h,间断4~8 h)鼻胃管注入为主。④部分胃动力障碍及需抑制胰腺分泌的患者改用鼻空肠管注入;部分气管切开有自主吞咽功能的患者加用经口进食。⑤肠内营养剂主要为游离氨基酸、短肽和整蛋白类。

1.3 统计学处理 以SPSS13.0统计软件进行数据处理,计量资料的数据呈正态分布时,数值以均数±标准差($\bar{x} \pm s$)表示。组间计量资料、计数资料的比较分别采用t检验和χ^2检验,经过组间比较有统计学意义的变量分别予以统计学变量赋值,再纳入多因素Logistic回归分析。$P<0.05$为差异有统计学意义。

2 结果

2.1 基本情况 192例危重症患者中62例(32.3%)达标,死亡病例38例(其中达标组2例)。两组年龄、性别、颈髓损伤和严重低钾血症等比较差异无统计学意义(P均>0.05)。两组APACHEⅡ评分、胃肠道手术后、重症胰腺炎、肾脏衰竭、儿茶酚胺类药物、镇静镇痛药物、肠内营养后腹胀等比较差异有统计学意义(P均<0.05)。见表1。

表1 两组患者基本情况的比较

项目	达标组 ($n=62$)	未达标组 ($n=130$)	P值
性别(男/女)	40/22	86/44	0.823
年龄(岁)	54±21	56±19	0.676
APACHEⅡ评分(分)	17.6±8.3	25.6±8.1	0.000*
胃肠道手术(n)	3	36	0.000*
儿茶酚胺类药物(n)	16	89	0.000*
镇静镇痛药物(n)	15	94	0.000*
肠内营养后腹胀(n)	12	72	0.000*
肾脏衰竭(n)	4	28	0.009*
重症胰腺炎(n)	0	12	0.013*
严重低钾血症(n)	14	18	0.129
颈髓损伤(n)	2	6	0.652

与达标组比较:* $P<0.05$

2.2 Logistic回归分析结果 由于所有重症胰腺炎患者肠内营养均未达标,而不能引入Logistic回归分析,其他经组间比较有意义的指标均纳入Logistic回归分析,显示胃肠道手术和儿茶酚胺类药物的使用对肠内营养达标具有明显的影响($P<0.05$),其OR值分别是7.094(95% CI 1.841~27.332)和3.076(95% CI 1.295~7.302)。见表2。

表2 Logistic回归分析结果

项目	B	S.E.	Wald	Sig.	OR	95% CI	
						Lower	Upper
胃肠道手术	1.959	0.688	8.106	0.004*	7.094	1.841	27.332
儿茶酚胺类药物	1.123	0.441	6.486	0.011*	3.076	1.295	7.302
胃肠营养后腹胀	0.728	0.445	2.677	0.102	2.072	0.866	4.958
肾脏衰竭	0.716	0.649	1.216	0.270	2.045	0.573	7.297
镇静镇痛药物	0.494	0.412	1.436	0.231	1.639	0.731	3.677
APACHEⅡ评分	0.048	0.27	3.252	0.071	1.050	0.996	1.106

* $P<0.05$

3 讨论

危重症患者由于存在严重感染、创伤或应激等因素,使机体处于高分解代谢状态,机体通过消耗瘦体组织来满足早期应激状态的代谢需求;同时,由于营养摄入不足,易导致营养不良。营养不良可引起感染和多器官衰竭发生率增高,伤口愈合减慢,机械通气和住院时间延长,住院费用增加,最终使总体死亡率增加[4]。肠内营养是目前营养支持的首选,但是,早期肠内营养达到目标营养量常较困难。同时,由于肠外营养可导致患者感染并发症的发生率增高,故美国危重病协会和肠内肠外营养协会的指南推荐[6],在使用各种方法使肠内营养达到最大化后才开始使用肠外营养作为补充。如何给予最优化的营养支持仍然是危重症治疗的难点和热点之一。2009 年我国 25 家大型医院 ICU 的现状调查分析发现,仅有 47.7% 的患者能通过肠内营养达到目标量[2]。Heidegger 等[5]建议,在入院前 3 d 内肠内营养给予目标营养量的 60%,以发挥肠内营养的治疗优势。

本研究结果显示,肠内营养达标率仅为 32.3%。进一步的 Logistic 分析表明,胃肠道手术是影响肠内营养达标的最主要因素。其原因主要是胃肠道解剖结构的中断。另外,等待患者排气排便的恢复,也是失去早期肠内营养时机的重要原因。Dissanaike 等[7]在对腹部创伤患者的研究中发现,早期肠内营养支持对腹部伤口愈合速度及死亡率均无影响,因此,给予早期肠内营养是安全的,并能减少腹部感染的发生率。目前多主张能使用一段肠管就使用一段的理念。例如,对胃十二指肠手术患者应早期积极使用空肠营养;低位结肠手术则可通过无渣营养液早期实施肠内营养。

本研究发现,儿茶酚胺类药物的使用也是影响肠内营养实施的另一重要因素。其原因是由于使用儿茶酚胺类药物的患者均存在不同程度的血流动力学障碍。胃肠道又是血流动力学不稳定最易受累的器官,即此时胃肠道处于缺血缺氧状态,较多的肠内营养可能会加重肠道负荷,不仅使胃肠道受损加重,而且使肠内营养并发症增加。Berger 等[8]的研究也表明,血流动力学不稳定可导致肠内营养不耐受。

另外,本研究也发现,对于重症胰腺炎患者,尽管使用空肠营养,也难以在早期达到目标营养量。故此,重症胰腺炎患者应在患者耐受范围内给予适当的肠内营养,通过联合肠外营养达到目标营养量。O′Meara 等[9]研究发现,肠内营养的达标不仅与患者的疾病特征和治疗药物有关,也与胃肠管路、胃潴留、俯卧位等相关。此外,制定合理的营养支持流程不仅可使患者更早开始肠内营养,而且也更容易使肠内营养达标[10]。

应该指出,由于本研究是一回顾性分析研究,纳入的疾病种类较为复杂,而各种疾病影响肠道功能的病理生理机制也不相同,故此,结论具有一定的局限性。

综上所述,除重症胰腺炎患者外,胃肠道完整性的破坏和血流动力学的障碍均是影响早期肠内营养达标的因素。

参考文献

1 Artinian V, Krayem H, DiGiovine B. Effects of early enteral feeding on the outcome of critically ill mechanically ventilated medical patients[J]. Chest, 2006, 129(4): 960 - 967.

2 周华, 杜斌, 柴文昭, 等. 我国危重症患者营养支持现状调查分析[J]. 肠外和肠内营养, 2009, 16(5): 259 - 263.

3 Genton L, Dupertuis YM, Romand JA, *et al.* Higher calorie prescription improves nutrient delivery during the first 5 days of enteral nutrition[J]. Clin Nutr, 2004, 23(3): 307 - 315.

4 Villet S, Chiolero RL, Bollmann MD, *et al.* Negative impact of hypocaloric feeding and energy balance on clinical outcome in ICU patients[J]. Clin Nutr, 2005, 24(4): 502 - 509.

5 Heidegger CP, Darmon P, Pichard C, *et al.* Enteral vs parenteral nutrition for the critically ill patient: a combined support should be preferred[J]. Curr Opin Crit Care, 2008, 14(4): 408 - 414.

6 Robert G, Stephen A, Vincent W, *et al.* Guidelines for the provision and assessment of nutrition support therapy in the adult critically ill patient: Society of Critical Care Medicine and American Society for Parenteral and Enteral Nutrition[J]. Crit Care Med, 2009, 37(5): 1 - 30.

7 Dissanaike S, Pham T, Shalhub S, *et al.* Effect of immediate enteral feeding on trauma patients with an open abdomen: protection from nosocomial infections[J]. J Am Coll Surg, 2008, 207(5): 690 - 697.

8 Berger MM, Revelly JP, Cayeux MC, *et al.* Enteral nutrition in critically ill patients with severe hemodynamic failure after cardiopulmonary bypass[J]. Clin Nutr, 2005, 24(1): 124 - 132.

9 O′Meara D, Mireles - Cabodevila E, Frame F, *et al.* Evaluation of delivery of enteral nutrition in critically ill patients receiving mechanical ventilation[J]. Am J Crit Care, 2008, 17(1): 53 - 61.

10 Diog GS, Simpson F, Finfer S, *et al.* Effect of evidence - based feeding guidelines on mortality of critically ill adults[J]. JAMA, 2008, 300(23): 2731 - 2741.

[收稿日期:2010 - 01 - 05][本文编辑:裴俏]
