# Supplementary material for: Development and external validation of machine learning models for the early prediction of malnutrition in critically ill patients: a prospective observational study
Source: BMC Med Inform Decis Mak. 2025 Jul 3;25:248. doi: 10.1186/s12911-025-03082-9 (PMC12225150; doi:10.1186/s12911-025-03082-9)
Supplement: Supplementary file 14 — Supplementary Material 14 [file 12911_2025_3082_MOESM14_ESM.pdf]

分类号：R4 单位代码：10752

密　级：公开 学　　号：201900733

宁夏医科大学

硕士专业学位论文

(专业学位)

脓毒症患者营养状况及影响因素分析

Analysis of nutritional status and influencing factors of patients with sepsis

学 位 申 请 人：杨宇璐

指 导 教 师：马晓薇教授

合 作 指 导 教 师：姚再先、沈佳副主任医师

申请学位门类级别：医学

专 业 名 称：急诊医学

研 究 方 向：脓毒症

所 在 学 院：临床医学院

论 文 完 成 日 期：二零二二年三月

宁夏医科大学研究生院

(C)1994-2023 China Academic Journal Electronic Publishing House. All rights reserved. http://www.cnki.net

Ningxia Medical University
Thesis for Application of Master’s Degree

Analysis of nutritional status and influencing factors of patients with sepsis

Student’s Name: YangYulu

Supervisor: Prof: Ma Xiaowei

Assistant supervisor: Shen Jia and Yao Zaixian

Subject Category: Medical science

Major: Emergency Medicine

Specialty: Spesis

College: Clinical medical school

Completion Date: March 2022

(C)1994-2023 China Academic Journal Electronic Publishing House. All rights reserved. http://www.cnki.net

宁夏医科大学学位论文独创性声明

本人郑重声明：所呈交的学位论文，是个人在导师的指导下，独立进行研究工作所取得的成果，无抄袭及编造行为。除文中已经特别加以注明引用的内容外，本论文不含任何其他个人或集体已经发表或撰写过的作品成果。对本文的研究做出重要贡献的个人和集体，均已在文中以明确方式标明并致谢。本人完全意识到本声明的法律结果由本人承担。

论文作者签名[illegible]　　　　论文导师签名[illegible]

2022年 5 月 16 日　　　　2022年 5 月 16 日

宁夏医科大学关于学位论文使用授权的声明

宁夏医科大学有权保留使用本人学位论文，同意学校按规定向国家有关部门机构送交论文的复印件和电子版，允许被查阅和借阅。本人授权宁夏医科大学可以将本学位论文的全部或部分内容编入有关数据库进行检索，可以采用影印、缩印或其他复印手段保存和汇编本学位论文。可以公布（包括刊登）论文的全部或部分内容。

（保密论文在解密后应遵守此规定）

论文作者签名[illegible]　　　　论文导师签名[illegible]

2022年 5 月 16 日　　　　2022年 5 月 16 日

(C)1994-2023 China Academic Journal Electronic Publishing House. All rights reserved. http://www.cnki.net

脓毒症患者营养状况及影响因素分析

摘 要

目的 ：通过观察脓毒症患者在住院期间营养状况，分析及探讨脓毒症患者在发病急性期早期、急性后期、恢复期的营养状况变化及影响因素，为合理营养干预提供依据。

方法：本研究为前瞻性研究。收集 2020 年 10 月 1 日-2021 年 12 月 30 日入住宁夏医科大学总医院重症医学科的符合纳入及排除标准 102 例脓毒症患者为研究对象。依据 2019 年欧洲临床营养与代谢学会(European Society for Parenteral and Enteral Nutrition, ESPEN)将危重患者按照其代谢特点将脓毒症的发病过程分为：急性期早期(ICU D1-2)、急性后期(ICU D3-D7)、恢复期(ICU D7 以后)，分别以入 ICU24 小时内(D1)、第 3 天(D3)、第 7 天(D7)代表上述各时期。

1.一般资料收集：年龄、性别、BMI、慢性病史(高血压、糖尿病、冠心病等)、饮酒史、吸烟史、运动情况。

2.临床资料收集：收集 24 小时内(D1)、第 3 天(D3)、第 7 天(D7)的 APACHEII评分、SOFA 评分、mNUTRIC 评分、营养学指标 (白蛋白、前白蛋白、转铁蛋白、总蛋白、血红蛋白、淋巴细胞绝对值、胆固醇、尿素/肌酐)，是否合并感染性休克、是否行机械通气、是否合并肾上腺皮质功能不全、营养支持方式、是否手术等情况。

3.静息能量消耗(REE)和呼吸商(RQ)测定：使用代谢车对 30 名患者进行静息能量消耗(REE)和呼吸商(RQ)测定。

4.采用重复测量方差分析比较 D1、D3、D7 营养学指标、静息能量消耗(REE)和呼吸商(RQ)变化。

5.采用 Spearman 相关分析 D1、D3、D7 营养学指标与 mNUTRIC 评分的关系。

6.首先通过单因素分析确定 D1、D3、D7 影响脓毒症患者营养状况的可疑因素，然后带入多重线性回归进一步确认脓毒症患者营养状况的影响因素。

结果：1.总共纳入 102 例脓毒患者为研究对象，男性患者 60 例(58.82%)，女性患者 42 例

(C)1994-2023 China Academic Journal Electronic Publishing House. All rights reserved. http://www.cnki.net

(41.18%)年龄在 31 岁～91 岁之间，平均年龄(66.3±11.32)岁。

2.(1)纳入 102 患者中，其中脓毒症 53 例(51.96%)，感染性休克 49 例(48.04%)；主要感染部位为肺部感染 49 例(46.08%)，其次为腹腔感染 23 例(22.5%)，泌尿系感染 9 例(8.82%)、皮肤软组织感染 9 例(8.82%)、血行感染 4 例(3.92%)、颅内感染 3 例(2.94%)、肝脓肿 2 例(1.96%)以及其他部位感染 5 例(4.90%)；入 ICU 前 1 周行手术的有 34 例(33.33%)，未行手术为 68 例(66.67%)；(2)研究对象行机械通气人数在 D1、D3、D7 分别为 64 例(62.75%)、56 例(54.90%)、38 例(37.25%)；(3)研究对象合并肾上腺皮质功能不全人数在 D1、D3、D7 分别为 42 例(41.18%)、34 例(33.33%)、30 例(29.41%)。

3.对患者营养学指标分析：与 D1 相比，在 D3、D7 时前白蛋白升高，在 D7 转铁蛋和尿素/肌酐升高，在 D3、D7 血红蛋白和胆固醇均下降，差异有统计学意义($P<0.05$)；与 D3 相比，在 D7 时转铁蛋白升高,差异有统计学意义($P<0.001$)，在 D7 时转铁蛋白、血红蛋白和尿素/肌酐无明显变化，差异无统计意义($P>0.05$)；在 D1、D3、D7 时白蛋白、总蛋白和淋巴细胞绝对值无明显变化，差异无统计学意义($P>0.05$)。

4.利用代谢车对 30 名患者进行静息能量消耗(REE)和呼吸商(RQ)测定：D1、D3 和 D7 静息能量消耗(REE)分别为：1884.64±347.49(kcal/d)、1792.18±259.10(kcal/d) 1627.36±306.52(kcal/d), 静息能量消耗逐渐降低，但变化不明显，进行两两比较时差异无统计学意义(P>0.05)；D1、D3、D7 呼吸商(RQ)分别为：0.79±0.048、0.86±0.052、0.93±0.043，呼吸商逐渐增加，进行两两比较时，差异均有统计学意义($P<0.001$)。

5.采用 mNUTRIC 评分对 D1、D3、D7 患者进行营养风险评估，mNUTRIC≥5 分表示患者存在营养风险。D1 存在营养风险为 43 例(42.15%)、D3 为 39 例(38.24%)、D7 为 29 例(28.43%)。

6.采用 Spearman 相关分析脓毒症患者 D1、D3、D7 营养学指标与 mNUTRIC 得分相关的关系，结果显示：D1 脓毒症患者营养学指标与 mNUTRIC 得分无相关关系($P>0.05$)；D3 脓毒症患者淋巴细胞绝对值与 mNUTRIC 得分呈负相关，淋巴细胞绝对值越低，mNUTRIC 得分越高，营养状况越差；与尿素/肌酐呈正相关，尿素/肌酐越高，mNUTRIC 得分越高，营养状况越差；D7 脓毒症患者白蛋白、胆固醇、总蛋白、血红蛋白、转铁蛋

(C)1994-2023 China Academic Journal Electronic Publishing House. All rights reserved. http://www.cnki.net

白与 mNUTRIC 得分呈负相关，上述指标越低，mNUTRIC 得分越高，提示营养状况越差。

7.对 D1、D3、D7 脓毒症患者营养状况进行单因素分析显示：(1)在 D1 饮酒、合并感染性休克、APACHEII评分≥15 分、合并皮质功能不全、行机械通气对营养状况影响有统计学意义($P<0.05$)；(2)在 D3 合并感染休克、APACHEII评分≥15 分、合并皮质功能不全、行机械通气、营养支持方式对营养状况影响有统计学意义($P<0.05$)；(3)在 D7 合并感染休克、APACHEII评分≥15 分、合并皮质功能不全、行机械通气、营养支持方式对营养状况影响有统计学意义($P<0.05$)。

8.将患者 D1、D3、D7 营养状况影响因素进行多重线性回归分析结果显示：D1、D3 营养状况的影响因素为合并感染性休克、APACHEII评分≥15 分、合并肾上腺皮质功能不全；D7 营养状况的影响因素为合并感染性休克、APACHEII评分≥15；

结论：1.脓毒症患者在急性期早期、急性后期、恢复期均存在高营养风险；

2.脓毒症患者营养状况的影响因素为合并感染性休克、合并肾上腺皮质功能不全、APACHEⅡ评分≥15。

【关键词】脓毒症，营养状况，静息能量消耗，代谢车，影响因素

(C)1994-2023 China Academic Journal Electronic Publishing House. All rights reserved. http://www.cnki.net

Analysis of nutritional status and influencing factors of patients with sepsis

ABSTRACT

Objective By observing the nutritional status of sepsis patients during hospitalization, we analyzed and discussed the changes of nutritional status and the influencing factors of sepsis patients in the early acute phase, late acute phase and recovery phase of the disease, so as to provide a basis for reasonable nutritional intervention.

Methods This study was a prospective study. One hundred and two patients with sepsis who met the inclusion and exclusion criteria and were admitted to the Department of Critical Care Medicine, General Hospital of Ningxia Medical University from October 1, 2020 to December 30, 2021 were collected for the study. According to the 2019 European Society for Parenteral and Enteral Nutrition (ESPEN), the pathogenesis of sepsis in critically ill patients was classified according to their metabolic characteristics: early acute phase (ICU D1-2), late acute phase (ICU D3-D7), recovery phase (after ICU D7), and the above periods are represented by 24 hours of ICU admission (D1), day 3 (D3), and day 7 (D7), respectively.

1. General data collection: age, gender, BMI, history of chronic diseases (hypertension, diabetes, coronary heart disease, etc.), history of alcohol consumption, history of smoking, and exercise.

2. Clinical data collection: APACHE II score, SOFA score, mNUTRIC score, nutritional parameters (albumin, prealbumin, transferrin, total protein, hemoglobin, absolute lymphocyte value, cholesterol, urea/creatinine) within 24 hours (D1), day 3 (D3), day 7 (D7), whether combined with infectious shock, whether mechanical ventilation was performed, whether combined with adrenocortical insufficiency, whether mechanical ventilation was performed. Whether combined with adrenal cortical insufficiency, nutritional support, whether surgery, etc.

3.Resting energy expenditure (REE) and respiratory quotient (RQ) were measured: resting energy expenditure (REE) and respiratory quotient (RQ) were measured in 30 patients using a

(C)1994-2023 China Academic Journal Electronic Publishing House. All rights reserved. http://www.cnki.net

metabolic cart.

4. Comparison of changes in D1, D3, D7 nutritional parameters, resting energy expenditure (REE) and respiratory quotient (RQ) using repeated measures ANOVA.

5. Spearman correlation was used to analyze the relationship between D1, D3, and D7 nutritional indices and mNUTRIC scores.

6. Firstly, the suspected factors of D1, D3 and D7 influencing the nutritional status of sepsis patients were identified by univariate analysis, and then brought into multiple linear regression to further confirm the influencing factors of nutritional status of sepsis patients.

Results 1. A total of 102 patients with sepsis were included in the study, 60 male patients (58.82%) and 42 female patients (41.18%) aged between 31 and 91 years, with a mean age of (66.3±11.32) years.

2.(1) Among the 102 patients included, there were 53 cases (51.96%) of sepsis and 49 cases (48.04%) of infectious shock; the main sites of infection were lung infection in 49 cases (46.08%), followed by abdominal infection in 23 cases (22.5%), urinary tract infection in 9 cases (8.82%), skin soft tissue infection in 9 cases (8.82%), bloodstream infection in 4 cases (3.92%), 3 cases of intracranial infection (2.94%), 2 cases of liver abscess (1.96%), and 5 cases of other site infections (4.90%); 34 cases (33.33%) had surgery one week before admission to the ICU, and 68 cases (66.67%) did not have surgery; (2) the number of study subjects on mechanical ventilation was 64 cases (62.75%) in D1, D3, and D7, respectively. 56 (54.90%) and 38 (37.25%) cases respectively; (3) the number of study subjects with combined adrenocortical insufficiency was 42 (41.18%), 34 (33.33%) and 30 (29.41%) cases in D1, D3 and D7, respectively.

3. Analysis of patients' nutritional indexes: compared with D1, prealbumin was elevated at D3 and D7, transferrin egg and urea/creatinine were elevated at D7, hemoglobin and cholesterol were decreased at D3 and D7, and the difference was statistically significant ($P<0.05$); compared with D3, transferrin was elevated at D7, and the difference was statistically

(C)1994-2023 China Academic Journal Electronic Publishing House. All rights reserved. http://www.cnki.net

significant (P<0.001), and at D7 transferrin, There was no significant change in hemoglobin and urea/creatinine at D7, and the difference was not statistically significant (P>0.05); there was no significant change in the absolute values of albumin, total protein and lymphocytes at D1, D3 and D7, and the difference was not statistically significant (P>0.05).

4. Resting energy expenditure (REE) and respiratory quotient (RQ) were measured in 30 patients using a metabolic cart: resting energy expenditure (REE) at D1, D3 and D7 were 1884.64±347.49(kcal/d), 1792.18±259.10(kcal/d) 1627.36±306.52(kcal/d), respectively. Energy consumption gradually decreased, but the change was not significant, and the difference was not statistically significant when two comparisons were made (P>0.05); respiratory quotient (RQ) of D1, D3 and D7 were 0.79±0.048, 0.86±0.052 and 0.93±0.043, respectively, and the respiratory quotient gradually increased, and the difference was statistically significant when two comparisons were made (P<0.001).

5. MNUTRIC score was used to assess the nutritional risk of D1, D3, and D7 patients. mNUTRIC ≥ 5 indicated that the patients were at nutritional risk. 43 (42.15%) patients were at nutritional risk for D1, 39 (38.24%) for D3, and 29 (28.43%) for D7.

6. Spearman's correlation was used to analyze the relationship between the nutritional indicators of D1, D3 and D7 sepsis patients and mNUTRIC score correlation, and the results showed that there was no correlation between the nutritional indicators of D1 sepsis patients and mNUTRIC score (P>0.05); the absolute value of lymphocytes in D3 sepsis patients was negatively correlated with mNUTRIC score, and the lower the absolute value of lymphocytes The lower the absolute value of lymphocytes, the higher the mNUTRIC score, the worse the nutritional status; there was a positive correlation with urea/creatinine, the higher the urea/creatinine, the higher the mNUTRIC score, the worse the nutritional status; in D7 sepsis patients, albumin, cholesterol, total protein, hemoglobin and transferrin were negatively correlated with the mNUTRIC score, the lower the above indexes, the higher the mNUTRIC score, suggesting the worse the nutritional status.

(C)1994-2023 China Academic Journal Electronic Publishing House. All rights reserved. http://www.cnki.net

7. Univariate analysis of the nutritional status of patients with sepsis in D1, D3, and D7 showed that (1) there was a statistically significant effect of alcohol consumption, combined infectious shock, APACHE II score ≥15, combined cortical insufficiency, and mechanical ventilation on nutritional status in D1 ($P<0.05$); (2) there was a statistically significant effect of combined infectious shock, APACHE II score ≥15, combined cortical insufficiency, and mechanical ventilation in D3 (2) in D3 co-infection shock, APACHE II score ≥ 15, combined cortical insufficiency, mechanical ventilation, nutritional support mode on nutritional status had a statistically significant effect ($P<0.05$); (3) in D7 co-infection shock, APACHE II score ≥ 15, combined cortical insufficiency, mechanical ventilation, nutritional support mode on nutritional status had a statistically significant effect ($P<0.05$).

8. Multiple linear regression analysis of the factors influencing the nutritional status of patients D1, D3 and D7 showed that the factors influencing the nutritional status of D1 and D3 were co-infected shock, APACHE II score ≥ 15, and co-infected adrenocortical insufficiency; the factors influencing the nutritional status of D7 were co-infected shock and APACHE II score ≥ 15.

Conclusion 1. Patients with sepsis are at high nutritional risk in the early acute, late acute, and recovery phases.

2. Factors influencing the nutritional status of patients with sepsis are combined infectious shock, combined adrenocortical insufficiency, and APACHE II score ≥ 15.

KEYWORDS Sepsis, Nutritional status, Resting energy expenditure, Metabolic vehicle, Influencing factors

(C)1994-2023 China Academic Journal Electronic Publishing House. All rights reserved. http://www.cnki.net

符号说明

缩略词	英文全称	中文全称
ICU	Intensive Care Unite	加强监护病房
ESPEN	European Society for Parenteral and Enteral Nutrition	欧洲临床营养与代谢学会
ASPEN	American Society for Parenteral and Enteral Nutrition	美国肠外与肠内营养学会
SOFA	Sequential Organ Failure assessment	序贯器官衰竭评分
APACHE II	Acute Physiology and Chronic Health EvaluationII	急性生理和慢性健康评分
NUTRIC	Malnutrition universal screening tool	营养风险评分
NRS2002	Nutrition risk screening	住院患者营养风险筛查评估表
SGA	Subjective global assessment	主观综合营养评估
RQ	Respiratory quotient	呼吸商
REE	Resting energy expenditure	静息能量消耗
EN	Enteral nutrition	肠内营养
PN	parenteral nutrition	肠外营养
CIRCI	Critical illness related corticosteroid insufficiency	危重症相关性肾上腺皮质功能不全
AGI	Acute Gastrointestinal Injury	急性胃肠道损伤
FI	Feeding Intolerance	喂养不耐受

(C)1994-2023 China Academic Journal Electronic Publishing House. All rights reserved. http://www.cnki.net

目录

前 言 1
材料与方法 4
结果 8
讨论 22
结论 27
参考文献 28
文献综述 33
综述参考文献 40
附录 45
致谢 47
攻读学位期间发表的学术论文目录 48
个人简介 49
开题、中期及学位论文答辩委员组成 50

(C)1994-2023 China Academic Journal Electronic Publishing House. All rights reserved. http://www.cnki.net

前 言

脓毒症(Sepsis)是机体对感染反应失调导致危及生命的器官功能障碍综合征[1]。据统计，2017 年，全球脓毒症发病约 4890 万例，与脓毒症相关的死亡约 1100 万例，占全球死亡人数的 19.7％[2]。50-70%的脓毒症幸存者出院死亡率继续增加，其中许多人面临重症肌病、神经病和重症监护后综合征，这导致了巨大的社会经济负担[3]。在 ICU 接受治疗的脓毒症患者由于代谢紊乱和全身炎症反应综合征(SIRS)或代偿性抗炎反应综合征(CARS)持续增加，伴随的感染和多器官衰竭导致吸收和消化障碍进而发生营养不良[4, 5]。营养不良的后果是治疗时间延长(30-50%)、脓毒症并发症发生率增加(15-30%)、呼吸和循环衰竭、心脏骤停以及死亡率增加(30-60%)[3]。脓毒症是重症医学面临的重要临床问题。

人体在健康时，分解代谢和合成代谢基本保持一致。脓毒症患者打破了这种代谢平衡，通常是分解代谢大于合成代谢。脓毒症在疾病发展各阶段代谢特点有所差异，依据欧洲临床营养与代谢学会(ESPEN)对危重患者进行代谢分期，脓毒症按照代谢可分为三个时期，急性期早期(ICU D1-2)：代谢不稳定且分解代谢急剧增加，血流动力学不稳定，心输出量减少、耗氧量减少、低体温，胰高血糖素、儿茶酚胺和游离脂肪酸的水平升高；急性后期(ICU D3-D7)：此期特点为症状改善、恢复或进入持续的炎症/分解代谢状态，住院时间延长。机体总耗氧量、代谢率、心输出量和能源物质(碳水化合物、氨基酸和脂肪)氧化增加；恢复期(ICU D7 以后)：明显的肌肉消耗和代谢趋于稳定[6, 7]。如果病情得到控制，内环境稳定以及全身炎症反应得到改善，葡萄糖利用率升高、蛋白质与脂肪合成增加，合成代谢逐渐占主导地位。相反，如果患者在恢复期不能完全恢复，则可能发展为持续炎症-免疫抑制-分解代谢综合征，表现为长期的免疫与代谢紊乱，静息能量消耗增加和严重分解代谢，导致营养不良、反复感染、瘦体组织减少、伤口不愈合等[8]。基于此，脓毒症患者营养状况变化有高度动态性特点。美国肠外与肠内营养学会(American Society for Parenteral and Enteral Nutrition，ASPEN)指出营养风险筛查是一个

(C)1994-2023 China Academic Journal Electronic Publishing House. All rights reserved. http://www.cnki.net

识别可能有营养不良或有营养不良风险的患者的过程，以确定是否需要进行详细的营养评估和适当的干预[9]。有营养风险的患者如果不接受营养支持将会导致并发症增加、住院时间增加。合理的营养支持对存在高营养风险和营养不足的患降低住院时间、改善预后有重要意义。

实施营养干预时，过多的营养物质摄入可造成肝功能及中性粒细胞功能受损、血糖、血脂及电解质紊乱、加重肝肾负担以及二氧化碳消耗增加；而营养补充不足则会导致抵抗力下降、肌肉萎缩肌力下降、营养不良等的发生、增加院内感染[10]。营养过多和营养不足时均会产生不良影响，对患者实行营养摄入的评估有重要意义。

由于机械通气、感染严重程度、合并症、合并基础疾病以及疾病的严重可影响脓毒症代谢进程，脓毒症各时期代谢可不同。美国重症医学会(SCCM)与 ASPEN 的《2016 年成人危重症病人营养支持治疗实施与评价指南》[11]及欧洲临床营养和代谢学会(ESPEN)指南均推荐[7]，对于危重患营养支持的能量评估，可采用间接测热法测定静息能量消耗(Resting energy expenditure，REE)。静息能量消耗(REE)是指机体禁食 2 小时以上，在合适温度下平卧休息 30 分钟后的能量消耗，维持人体最基本生命活动所需的能量，约占每日能量消耗的 65-70% [12]，因此经常用 REE 来代替危重症患者的总能量消耗[13]。呼吸商(RQ)被认为是底物氧化的极好指标，可以反映三大营养物质的供能情况。它是产生的二氧化碳量与消耗的氧气量的比率[14, 15]。监测呼吸商在不同时期的变化，依据呼吸商可为患者制订个体化、精准的热卡供给方案。

传统评估营养状况的如身体质量指数(BMI)根据病人的体重(kg)和身高(米)的平方计算。因为水肿或使用利尿剂等引起体液平衡的改变，或患者处于危重状态不易搬动测量，体重指数和体重测量在评估危重病人的营养状况时存在误差。因此 BMI 对脓毒症患者的营养状况监测价值不大[16]。

目前营养状况评估方法众多，分为单一营养指标以及复合营养指标。常见的有住院患者营养风险筛查评估表(Nutrition risk screening，NRS2002)、营养不良通用筛查工具(malnutrition universal screening tool，MUST)和主观全球评估(Subjective global assessment，SGA)。这些工具通评分通常包括身高、近期体重变化、膳食摄入变化等指标。在入住 ICU

(C)1994-2023 China Academic Journal Electronic Publishing House. All rights reserved. http://www.cnki.net

的脓毒症患者这一特殊群体中，大多数患者处于昏迷状态或需要机械通气、镇静，上述指标无法准确获得。

实验指标如血红蛋白(Hemoglobin，HGB)、白蛋白(albumin, ALB)、总蛋白(Total protein,TP)、转铁蛋白(Transferrin,TF)、前白蛋白(Prealbumin,PA)、淋巴细胞绝对值(absolute lymphocyte,LYM)、胆固醇(total cholesterin,TC)、尿素/肌酐(Urea/creatinine，UCR)等，脓毒症患者营养状况的改变，可出现血清蛋白以及部分营养学指标的变化，因此监测营养学指标变化是有必要的。

Heyland 等学者[17]开发并验证了一种营养风险筛查的工具营养风险评分表(Malnutrition universal screening tool，NUTRIC)，专门应用于危重病人。根据患者的营养风险区分哪些患者将从更积极的营养治疗中受益。该评分其中两项指标为序贯器官衰竭评估(Sequential Organ Failure assessment,SOFA)和急性生理学和慢性健康评估(Acute Physiology and Chronic Health EvaluationII,APACHE II)。除了白细胞介素-6(IL-6)，NUTRIC 评分的其他变量很容易获得，(IL-6)不是重症监护室常规测量指标。当考虑到 IL-6 时，此工具的最终得分为 0-10，评分≥6 为高危患者。随后 Rahman[18]等人进行了研究，验证了未使用 IL-6 的危重病人的(改良)营养风险评分(mNUTRIC)。在没有 IL-6 的情况下，评分范围为 0-9 分别，评分≥5 的患者是营养不良风险患者，分值越高者营养风险越大。 ASPEN 指南建议使用 NUTRIC 评分评估危重患者的营养风险[11]。目前，ICU 患者营养不良没有公认的客观标准，但明显的分解代谢临床表现总是需要从营养的角度进行处理[9]。

单一指标并不能全面反应脓毒症患者的营养状况，脓毒症患者需要在结合营养学指标及营养风险筛查工具评估其营养状况。本研究分析脓毒症患者于 24 小时内(D1)、第 3 天(D3)、第 7 天(D7)营养学指标、部分患者静息能量消耗(REE)和呼吸商(RQ)、营养风险情况探讨影响脓毒症患营养状况的因素，为脓毒症患者营养支持个体化、精准化提供理论依据。

(C)1994-2023 China Academic Journal Electronic Publishing House. All rights reserved. http://www.cnki.net

材料与方法

1. 一般资料

1.1 研究对象

本研究共纳入在 2020 年 10 月 1 号-2021 年 12 月 30 号期间入住宁夏医科大学总院心脑血管医院重症医学科的 102 例患者为研究对象。

1.2 纳入标准：

(1)年龄≥18 岁；

(2)符合脓毒症 Sepsis3.0 诊断标准[19]：感染+SOFA≥2 分；

1.3 排除标准：

(1)年龄<18 岁；

(2)恶性肿瘤晚期患者；

(3)严重肝病、肾病的患者；

(4)合并风湿免疫疾病患者；

(5)入住 ICU 时间小于 7 天(包括转科、出院、死亡)。

2.方法

收集纳入研究的 102 例患者一般资料、营养学指标、是否合并感染性休克、感染部位、营养干预情况等临床资料。本研究属于前瞻性观察性研究，整个过程不干预临床决策。

2.1.1 一般资料

一般资料包括姓名、年龄、BMI、既往病史(高血压、糖尿病、冠心病、其他慢性病史)、饮酒史、吸烟史、入 ICU 前运动情况(≥8 小时/周、<8 小时/周)。

2.1.2 临床资料

收集入 ICU 患者 D1、D3、D7 患者的 APACHEII评分(见附表 1)、SOFA 评分(见附表 2)，是否合并感染性休克、行机械通气、合并危重病相关肾上腺皮质功能不全、主要感染部位、是否手术等。

2.1.3 营养学指标

(C)1994-2023 China Academic Journal Electronic Publishing House. All rights reserved. http://www.cnki.net

收集 D1、D3、D7 患者的血红蛋白(HGB)、白蛋白(ALB)、总蛋白(TP)、转铁蛋白(TF)、前白蛋白(PA)、淋巴细胞绝对值(LYM)、胆固醇(TC)、尿素/肌酐(UCR)

2.1.4 代谢车测定静息能量消耗(REE)和呼吸商(RQ)

30 名脓毒患者于 D1、D3、D7 利用代谢车测定静息能量消耗(REE)和呼吸商(RQ)。具体操作步骤如下：

(1)患者准备：测试前禁食或者停止肠内营养 2h 以上和前一小时无刺激(翻身、静脉采血、吸痰)，平静仰卧 30min 后测试；

(2)设备：采用美国麦加菲 CCM EXPRESS 代谢车营养代谢测试系统。机械通气患者通过器官插管与代谢车连接(图 1)、自主呼吸的患者使用面罩与代谢车连接(图 2)[20]。

(3)代谢车工作原理：通过密封的机械通气管路或者面罩，测量一定时间内氧气消耗量(VO_2)和二氧化碳产生量(VCO_2)，计算出呼吸商(RQ)。通过再根据 Weir 公式计算出这一时间内的能量消耗，推算出 24 小时内静息能量消耗(REE)。

(4)操作方法与流程：由经过专业培训的代谢车操作师进行测定，测量前对机器进行预热、校准、定标。使用呼吸机时，氧浓度低于 65%。测量时保持患者处于“稳定状态”，“稳定状态”的定义为连续 5 分钟内患者 VO2 和 VCO2 变化小于 10%，采集稳态阶段数据，测定时间约为 15-30 分钟。测量期间按照质控标准严格进行，避免吸痰、翻身、更衣、采血等护理操作。整个过程由同一人完成。

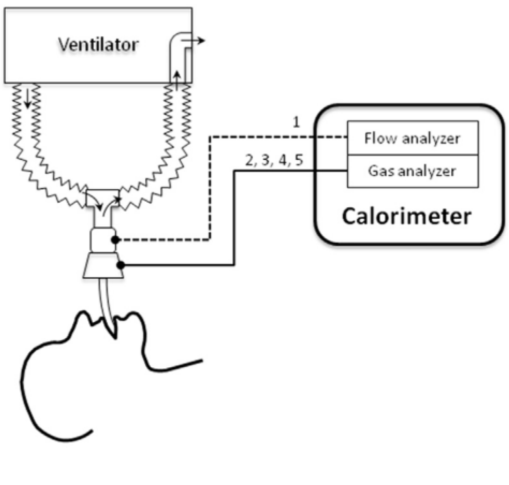

图 1 机械通气患者与代谢车连接示意图

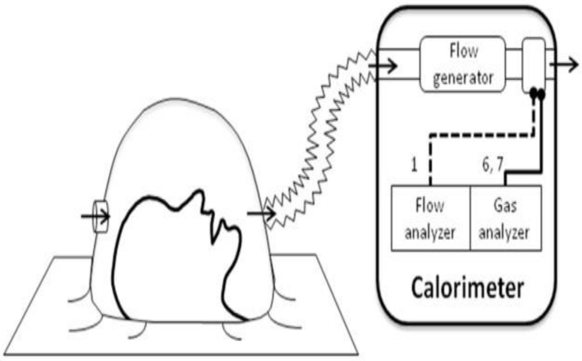

图 2 自主呼吸患者与代谢车连接示意图

(C)1994-2023 China Academic Journal Electronic Publishing House. All rights reserved. http://www.cnki.net

2.1.5 营养风险评估

于 D1、D3、D7 采用 mNUTRIC(见附表 3)评分方法筛查患者营养风险。mNUTRIC 评分≥5 分表示存在营养风险。

2.1.6 营养干预情况

包括普通饮食、肠内营养(EN)、全肠外营养(PN)、肠内联合肠外营养(EN+PN)四种方式。

2.2 相关定义以及诊断方法

(1)感染性休克：尽管给予充分的液体复苏，仍然表现为急性循环功能障碍，需要升压药物治疗的低血压伴氧利用不足(高乳酸血症)。

(2)危重症相关肾上腺皮质功能不全(critical illness-related corticosteroid insufficiency, CIRCI)：美国危重病医学会/欧洲危重病医学会在 2017 年关于 CIRCI 的指南中指出[21]静脉推注 250ugACTH 试验(HD-ACTH)后 60min,测定血浆总皮质醇含量低于 248.4nmol/L(9ug/dL)或随机总皮质醇水平低于 276.0nmol/L(10ug/dL),则可能存在 CIRCI。本研究诊断的肾上腺皮质功能不全为随机总皮质醇水平低于 276.0nmol/L(10ug/dL)。

(3)普通饮食：患者可经口自主进食，而且没有专门配置营养配方。

(4)肠内营养(EN)：指通过胃肠道提供人体消化及代谢所需的各种营养物质的营养支持模式，主要包括鼻胃管、鼻空肠饲管和空肠造口管等[9]。或者口服营养剂达到营养需求。

(5)全肠外营养(PN)：是指患者的全部营养需求(所有的宏观和微观营养)均由肠外营养提供，且除了静脉内营养外，不通过任何途径给予营养的情况[9]。

(6)肠内联合肠外营养：患者通过管饲或口服营养剂不能满足营养需求，还需要通过静脉输入营养物质，从而达到患者营养需求。

(7)手术：规定为入 ICU 前 1 周内行急诊或者择期手术治疗。

2.3 伦理学

本研究符合医学伦理学标准，并获得医院伦理委员会批准(审批号：KYLL-2021-26)。

2.4 统计学方法

(1)统计学分析采用 SPSS21.0 软件进行数据统计分析，以 α=0.05 作为比较的检验水准。

(C)1994-2023 China Academic Journal Electronic Publishing House. All rights reserved. http://www.cnki.net

(2)符合正态分布的计量资料采用均数±标准差进行描述；不符合正态分布则采用中位数和四分位间距描述；计数资料采用频数、百分比等进行描述。

(3)D1、D3、D7 营养学指标、静息能量消耗(REE)和呼吸商(RQ)变化情况采用重复测量方差分析。

(4)采用 Spearman 相关分析 D1、D3、D7 营养学指标与 m NUTRIC 得分的关系。

(5)①单因素分析：采用卡方检验分析患者一般资料对营养状况影响，采用 Kruskal Wallis 检验分析 D1、D3、D7 疾病严重程度、合并感染性休克、行机械通气、肾上腺皮质功能不全、营养支持方式、手术对营养状况的影响；②多重线性回归探索脓毒症患者营养状况的影响因素。

(C)1994-2023 China Academic Journal Electronic Publishing House. All rights reserved. http://www.cnki.net

结果

3.1 研究对象的资料

3.1.1 研究对象的一般资料

本研究共纳入 102 例脓毒患者为研究对象，其中男性患者 60 例(58.82%)，女性 42 例(41.18%)；年龄在 31 岁～91 岁，平均年龄(66.3±11.32)岁；BMI 大于等于 24(kg/m^2)最多，为 59 例(57.84%)；合并糖尿病为 38 例(37.25%)，不合并糖尿病为 64 例(62.75%)；合并高血压为 45 例(44.12%)，不合并高血压为 57 例(55.88%)；合并冠心病为 22 例(21.57%)，不合并冠心病为 80 例(78.43%)；合并其他慢性病有 30 例(29.41%)，不合并其他慢性病为 72 例(70.59%)；纳入脓毒症患者一般资料。见表 1：

表 1 一般情况资料表(n=102)

参数	例数(n)	构成比(%)
年龄(岁)		
≥65	55	54.92
<65	47	46.08
性别		
男	60	58.82
女	42	41.18
BMI(kg/m^2)		
≤18.5	13	12.75
18.5-23.9	40	39.22
≥24	59	57.84
糖尿病		
有	38	37.25
无	64	62.75
高血压		
有	45	44.12
无	57	55.88
冠心病		
有	22	21.57
无	80	78.43
其他慢性病		
有	31	30.39
无	71	69.61
饮酒史		

(C)1994-2023 China Academic Journal Electronic Publishing House. All rights reserved. http://www.cnki.net

有	42	41.18
无	60	58.82
吸烟史		
有	38	37.25
无	64	62.75
运动情况		
≥8 小时/周	42	41.18
<8 小时每周	60	58.82

3.1.2 研究对象临床资料

本研究纳入脓毒症患者为 53 例(51.96%)，感染性休克为 49 例(48.04%)；主要感染部位为肺部感染有 47 例(46.08%)，其次为腹腔感染有 23 例(22.55%)，余为泌尿系感染、皮肤软组织感染、血性感染、颅内感染、肝脓肿以及其他部位感染。入 ICU 前 1 周内行手术的有 34 例(33.33%)，未行手术为 68 例(66.67%)。其中普通饮食有 29 例(28.43%)；肠内营养为 43 例(42.16%)；全肠外营养 15 例(14.71%)；肠内联合肠外营养为 15 例(14.71%)；研究对象临床资料如表 2 所示：

表 2　研究对象临床资料(n=102)

变量	例数(n)	构成比(%)
脓毒症类型		
脓毒症	53	51.96
感染性休克	49	48.04
主要感染部位		
肺部感染	47	46.08
腹腔感染	23	22.55
泌尿系感染	9	8.82
皮肤软组织感染	9	8.82
血性感染	4	3.92
颅内感染	3	2.94
肝脓肿	2	1.96
其他组部位感染	5	4.90
手术		
有	34	33.33
无	68	66.67
营养支持方式		
普通饮食	29	28.43
肠内营养	42	42.16

(C)1994-2023 China Academic Journal Electronic Publishing House. All rights reserved. http://www.cnki.net

全肠外营养	15	14.71
肠内联合肠外营养	15	14.71
机械通气人数		
无机械通气	64	62.75
机械通气	38	37.25
皮质功能不全		
合并皮质功能不全	42	41.18
未合并皮质功能不全	60	58.82

3.2 研究对象 D1、D3、D7 脓毒症患者营养状况变化

3.2.1 研究对象 D1、D3、D7 血清蛋白变化情况

对 D1、D3、D7 血清蛋白进行重复测量方差分析，并绘制趋势变化图(见图 3-6)。与 D1 相比，D3、D7 天前白蛋白升高,D7 转铁蛋白升高经，差异有统计学意义(P<0.001)；与 D3 相比，前白蛋白在 D7 无明显变化,差异无统计学意义(P>0.05)，转铁蛋白在 D7 有所升高，差异有统计学意义(P<0.001)；白蛋白和总蛋白在 D1、D3、D7 无明显变化，差异无统计学意义(P>0.05)，见表 3。

表 3 脓毒症患者 D1、D3、D7 血清蛋白变化情况(x±s)(n=102)

时间	白蛋白(g/L)	前白蛋白(g/L)	转铁蛋白(g/L)	总蛋白(g/L)
D1	31.23±6.14	0.089±0.047	1.16±0.078	55.57±9.61
D3	31.87±3.53	0.14±0.101[a]	0.99±0.082	54.15±6.55
D7	31.72±3.28	0.18±0.075[a]	1.38±0.071[a,b]	54.93±7.03
F	0.715	12.780	10.099	1.236
P	0.452	<0.001	<0.001	0.291

注： a 表示与 D1 比较差异有统计学意义(P<0.05);b 表示与 D3 比较差异有统计学意义(P<0.05)。

(C)1994-2023 China Academic Journal Electronic Publishing House. All rights reserved. http://www.cnki.net

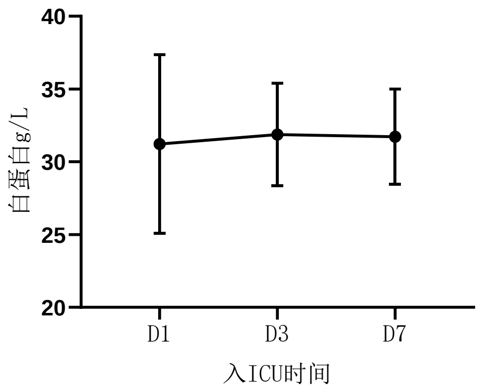

图 3　白蛋白变化趋势

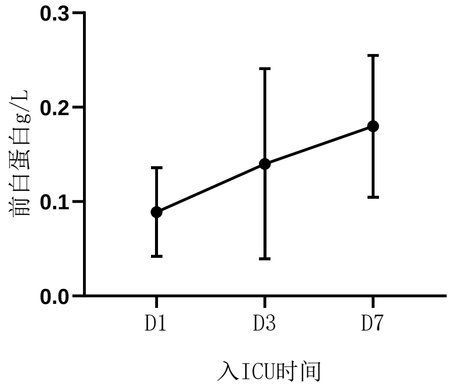

图 4　前白蛋白变化趋势

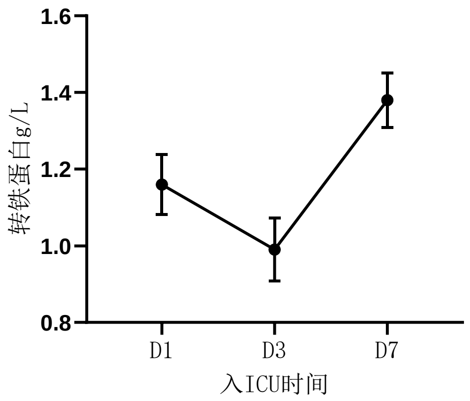

图 5　转铁蛋白变化趋势

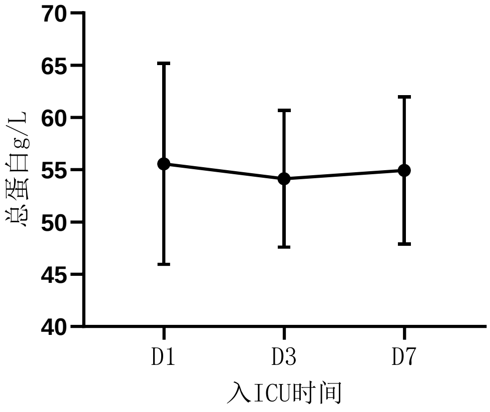

图 6　总蛋白变化趋势

3.2.2 研究对象 D1、D3、D7 脓毒症患者 HB、TC、LYM、UCR 变化情况

对 D1、D3、D7 的 HB、TC、LYM、UCR 进行重复测量方差分析，并绘制趋势变化图(图 7-10)。与 D1 相比，血红蛋白在 D3、D7 时均下降，差异有统计学意义(P<0.001)，尿素/肌酐在 D7 时升高，差异有统计学意义(P<0.05)；与 D3 相比，尿素/肌酐和血红蛋在 D7 时无明显变化，差异无统计意义(P>0.05)，；在 D1、D3、D7 时淋巴细胞绝对值无明显变化，差异无统计学意义（(P>0.05)。具体如表 4 所示：

(C)1994-2023 China Academic Journal Electronic Publishing House. All rights reserved. http://www.cnki.net

表 4 脓毒症患者 D1、D3、D7 的 HB、TC、LYM、UCR 变化情况(x±s)(n=102)

时间	血红蛋白(g/L）	胆固醇(mmol/L)	淋巴细胞绝对值	UCR
D1	117.99±31.13	3.16±1.11	0.95±0.91	0.11±0.050
D3	107.05±22.92[a]	2.76±0.94[a]	0.98±0.70	0.109±0.051
D7	105.28±21.56[a]	2.78±0.94[a]	1.14±0.70	0.12±0.063[a]
F	22.41	9.726	2.245	6.039
P	<0.001	<0.001	0.120	0.003

注：a 表示与 D1 比较差异有统计学意(P<0.05);b 表示与 D3 比较差异有统计学意(P<0.05)。

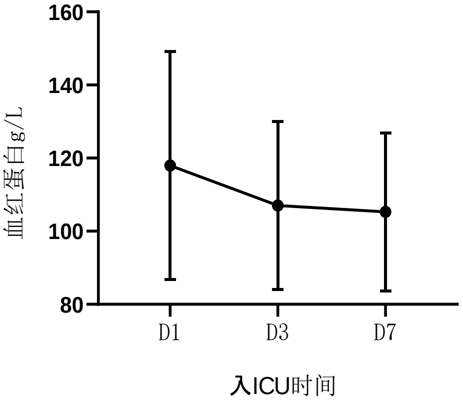

图 7 血红蛋白变化趋势

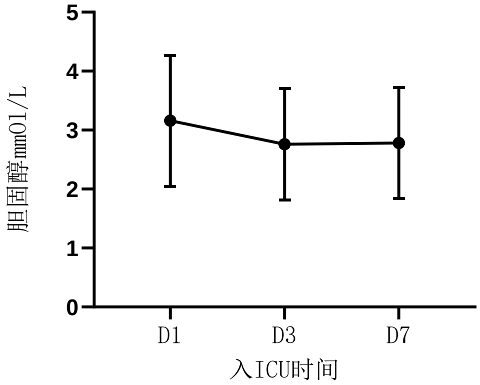

图 8 胆固醇变化趋势

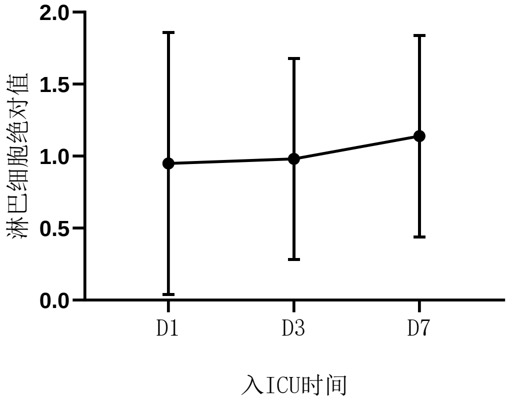

图 9 淋巴细胞绝对值变化趋势

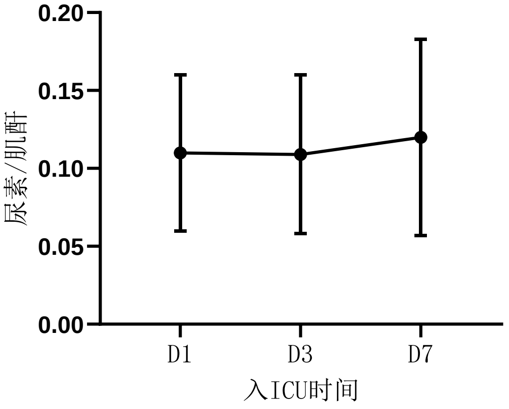

图 10 尿素/肌酐变化趋势

3.2.3 研究对象 D1、D3、D7 静息能量消耗(REE)与呼吸商(RQ)变化情况

对 30 名脓毒症患者于 D1、D3、D7 使用代谢车测定患者 REE 和 RQ 并绘制变化趋势图(图 11,12)。患者 REE 随着住时间逐渐降低,进行两两比较， REE 比较差异无统计

(C)1994-2023 China Academic Journal Electronic Publishing House. All rights reserved. http://www.cnki.net

学意义(P>0.05)。呼吸商(RQ)随着住院时间逐渐增加，进行两两比较时，D1 与 D3 呼吸商，D1 与 D7 天呼吸商、D3 与 D7 比较有差别，差异有统计学意义(P<0.001)。如表 5 所示：

表 5 脓毒症患者 D1、D3、D7 的 REE、RQ 变化情况(x±s)(n=30)

时间	REE(kcal/d)	呼吸商
D1	1884.64±347.491	0.80±0.048
D3	1792.18±259.103	0.86±0.052[a]
D7	1627.36±306.521	0.93±0.043[a,b]
F	2.472	17.525
P	0.11	<0.001

注： a 表示与 D1 比较差异有统计学意义，(P<0.05);b 表示与 D3 比较差异有统计学意义(P<0.05)。

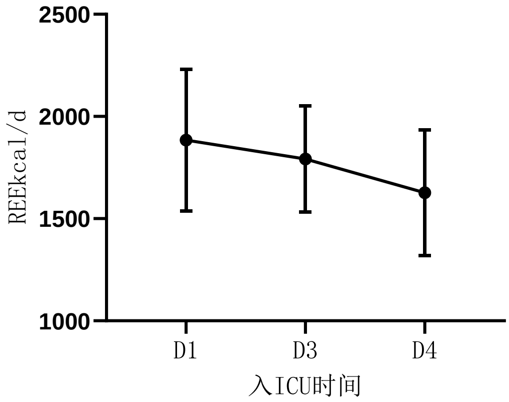

图 11 REE 变化趋势

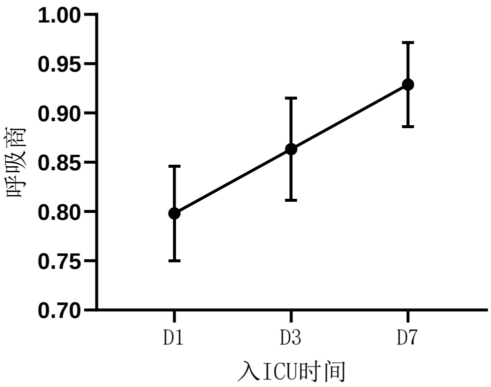

图 12 呼吸商变化趋势

3.2.4 研究对象 D1、D3、D7 营养风险变化情况

使用 mNUTRIC 评分对纳入患者进行营养风险评估。D1 时，患者存在营养风险例数为 43 例(42.15%)；D3 时，存在营养风险例数为 39 例(38.24%)；D7 时，存在营养风险例数为 29 例(28.43%)。在 D1 存在营养风险的 43 例当中，经过治疗，在 D3 有 31 例仍然在营养风险，经过综合治疗后 D7 评估营养风险时仍然有 21 存在营养风险(见表 7，图 15)。对 D1、D3、D7mNUTRIC 得分进行统计，从表中可见，D1 时，mNUTRIC 评分为 4 分人数最多，有 28 例，其次为 6 分，有 20 例；D3 时，mNUTRIC 评分为 4 分人数

(C)1994-2023 China Academic Journal Electronic Publishing House. All rights reserved. http://www.cnki.net

最多，有 27 例，其次为 3 分，有 26 例；D7 时， mNUTRIC 评分为 3 分人数最多，有 35 例，其次为 4 分，有 23 例(见表 6，图 13)。

表 6　脓毒症患者在 D1、D3、D7 高营养风险人数

例数	D1	D3	D7
总高营养风险人数(n/%)	43(42.16%)	39(38.24%)	29(28.43%)
持续高营养风险人数(n/%)	43(42.16%)	31(30.39%)	21(20.59%)

注：持续高营养风险人数：在 D1 高营养风险的患者，经过治疗在 D3、D7 评估时仍然存在营养风险。总高营养风险人数：持续营养风险人数加上新发高营养风险人数。

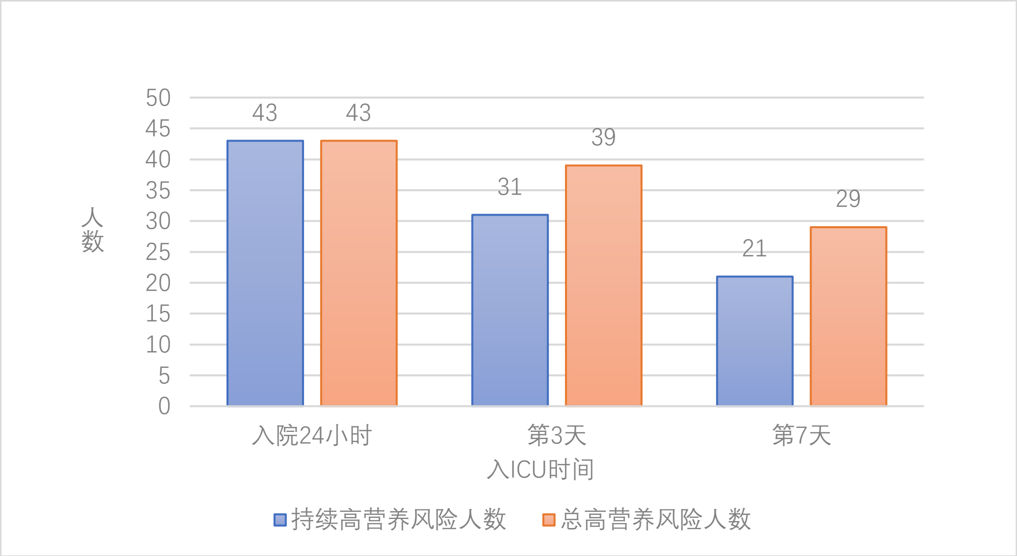

图 13　D1、D3、D7 高营养风险人数变化情况

表 7 脓毒症患者 D1、D3、D7mNUTRIC 得分布情况(n=102)

mNUTRIC 得分	D1	D3	D7
1	2	0	1
2	11	10	14
3	18	26	35
4	28	27	23
5	13	16	11
6	20	13	10
7	5	9	7
8	4	1	1
9	1	0	0

(C)1994-2023 China Academic Journal Electronic Publishing House. All rights reserved. http://www.cnki.net

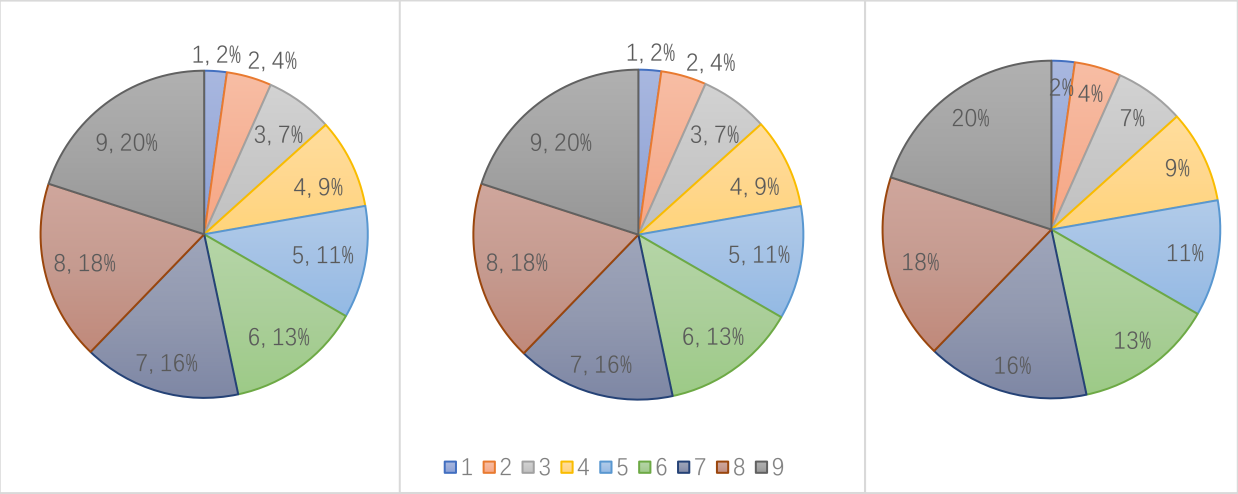

D1 D3 D7

图 14 脓毒症患者在 D1、D3、D7 的 mNUTRIC 得分占比

3.2.4 研究对象营养学指标与 mNUTRIC 得分的相关性分析

Spearman 相关分析结果提示，脓毒症患者在 D1 营养学指标和 mNUTRIC 得分无相关关系(*P*>0.05)，结果表 8 所示：

表 8 研究对象 D1 营养学指标与 mNUTRIC 得分的相关性分析

分析因素	相关系数	*P*
白蛋白	-0.118	0.245
淋巴细胞绝对值	0.012	0.903
胆固醇	-0.142	0.17
血红蛋白	0.091	0.368
总蛋白	-0.09	0.378
UCR	0.108	0.29
前白蛋白	-0.062	0.671
转铁蛋白	-0.208	0.142

Spearman 相关分析结果提示，脓毒症患者在 D3 淋巴细胞绝对值与 mNUTRIC 得分

(C)1994-2023 China Academic Journal Electronic Publishing House. All rights reserved. http://www.cnki.net

成负相关，既淋巴细胞绝对值越低，mNUTRIC 得分越高，营养状况越差(P<0.05)。D3 尿素/肌酐与 mNUTRIC 得分呈正相关，既尿素/肌酐越高，mNUTRIC 得分越高，营养情况越差；但相关关系并不密切(相关关系均小于 0.5)。结果如表 9 所示：

表 9 研究对象 D3 营养学指标与 mNUTRIC 得分的相关性分析

分析因素	相关系数	*P*
白蛋白	-0.066	0.519
淋巴细胞绝对值	-0.316	0.002
胆固醇	-0.183	0.104
血红蛋白	-0.059	0.565
总蛋白	-0.167	0.099
UCR	0.211	0.039
前白蛋白	-0.062	0.656
转铁蛋白	-0.195	0.145

Spearman 相关分析结果提示，脓毒症患者在 D7 天提示白蛋白、胆固醇、淋巴细胞绝对值、胆固醇、血红蛋白、总蛋白、转铁蛋白与 mNUTRIC 得分成负相关，即上述指标越低，mNUTRIC 得分越高，提示营养状况越差；但相关关系并不密切(相关关系均小于 0.5)。结果如表 10 所示：

表 10 研究对象 D7 营养学指标与 mNUTRIC 得分的相关性分析

分析因素	相关系数	*P*
白蛋白	-0.449	<0.001
淋巴细胞绝对值	-0.345	<0.001
胆固醇	-0.355	<0.001
血红蛋白	-0.27	0.007
总蛋白	-0.444	<0.001

(C)1994-2023 China Academic Journal Electronic Publishing House. All rights reserved. http://www.cnki.net

UCR	0.143	0.157
前白蛋白	-0.012	0.933
转铁蛋白	-0.308	0.025

3.4 研究对象 D1、D3、D7 营养状况影响因素单因素分析

3.4.1 研究对象 D1 营养状况影响因素单因素分析

3.4.1.1 一般资料对 D1 营养状况单因素分析

D1 按照有无营养风险分为有营养风险和无营养风险行单因素分析，单因素分析结果显示：两组患者是否饮酒比较差异有统计学意义($P<0.05$)，而性别、年龄、运动情况、BMI、是否吸烟、是否有慢性病(高血压、糖尿病、冠心病等)等因素的比较差异均无统计学意义($P>0.05$)，详见表 11。

表 11 一般资料对脓毒症患者 D1 营养状况分析 (n=102)

因素	有营养风险(n)	无营养风险(n)	X^2	*P*
性别				
男	27	33	0.483	0.487
女	16	26		
年龄(岁)				
≥65	25	30	45.134	0.198
<65	18	29		
BMI(kg/m^2)				
≤18.5	6	7	79.44	0.59
18.5-23.9	16	24		
≥24	21	28		
饮酒史				
有	23	19	4.652	0.031
无	20	40		
吸烟史				
有	19	18	2.725	0.099
无	24	41		
运动情况				
≥8 小时/周	19	23	2.83	0.092
<8 小时每周	24	36		
糖尿病				
有	16	22	3.64	0.994
无	27	37		

(C)1994-2023 China Academic Journal Electronic Publishing House. All rights reserved. http://www.cnki.net

高血压				
有	18	27	0.154	0.695
无	25	32		
冠心病				
有	12	10	1.765	0.184
无	31	49		
其他慢性病				
有	13	18	0.081	0.776
无	30	41		

3.4.1.2 临床资料对研究对象 D1 营养状况单因素分析

分析疾病严重程度、感染严重程度、机械通气、手术、肾上腺皮质功能不全对营养状况的影响，结果显示，APACHEII 评分≥15、合并感染性休克、行机械通气、合并肾上腺皮质对 D1 营养状况存在影响，差异有统计学意义(P<0.05)，具体如表 12 所示：

表 12 临床资料对脓毒症患者 D1 营养状况分析 (n=102)

因素	n(%)	mNUTRIC 评分	H	*P*
APACHEII 评分				
≥15	48 (47.06)	6(5,6)	57.828	<0.001
<15	54 (52.94)	3(2.75,4)		
脓毒症类型				
脓毒症	53(48.1)	3(3,4)	20.419	<0.001
感染性休克	49(51.9)	5(4,6)		
机械通气				
机械通气	64(62.75)	5(4,6)	17.966	<0.001
无机械通气	38(37.25)	3(2,5)		
手术				
手术	34(33.33)	5(3,6)	2.622	0.105
未行手术	68(66.67)	4(3,6)		
皮质功能不全				
合并皮质功能不全	42(41.18)	5(4,6)	33.14	<0.001
未合并皮质功能不全	60(58.82)	3(3,4)		

3.4.2 研究对象 D3 营养状况影响因素单因素分析

在 D3 按照有无营养风险分为有营养风险和无营养风险，进行单因素分析，结果显示：性别、年龄、运动情况、BMI、是否吸烟、是否饮酒、是否有慢性病(高血压、糖尿

(C)1994-2023 China Academic Journal Electronic Publishing House. All rights reserved. http://www.cnki.net

病、冠心病等)等因素的比较差异均无统计学意义(P>0.05)，详见表 13：

表 13 一般资料对脓毒症患者 D3 营养状况影响(n=102)

因素	有营养风险(n)	无营养风险(n)	X^2	P
性别				
男	22	38	0.152	0.697
女	17	25		
年龄(岁)				
≥65	25	30	48.99	0.109
<65	14	33		
BMI(kg/m^2)				
≤18.5	6	9		
18.5-23.9	10	27	79.416	0.591
≥24	23	27		
饮酒史				
有	18	24	1.136	0.286
无	21	39		
吸烟史				
有	16	22	0.384	0.535
无	23	41		
运动情况				
≥8 小时/周	16	26	1.876	0.357
<8 小时/每周	23	34		
糖尿病				
有	12	26	1.136	0.286
无	27	37		
高血压				
有	15	30	0.819	0.435
无	24	33		
冠心病				
有	8	14	0.042	0.838
无	31	49		
其他慢性病				
有	10	21	0.432	0.511
无	29	42		

3.4.2.2 临床资料对研究对象 D3 营养状况单因素分析

分析疾病严重程度、感染严重程度、机械通气、手术、营养支持方式、肾上腺皮质功能不全对营养状况的影响，结果显示，APACHEII 评分≥15、合并感染性休克、行机械通气、合并肾上腺皮质对 D3 营养状况存在影响，差异有统计学意义(P<0.05)，具体如表 14

(C)1994-2023 China Academic Journal Electronic Publishing House. All rights reserved. http://www.cnki.net

所示：

表 14 临床资料对脓毒症患者 D3 营养状况分析 (n=102)

因素	n(%)	mNUTRIC 评分	H	*P*
APACHEII 评分				
≥15	36(35.29)	5(4,6)	28.622	<0.001
<15	66(64.71)	3(3,4)		
脓毒症类型				
脓毒症	53(48.1)	3(3,4)	4.943	0.026
感染性休克	49(51.9)	4(3,5)		
机械通气				
机械通气	56(54.90)	4(3,5)	11.622	<0.001
无机械通气	46(45.10)	3(2,4)		
手术				
手术	34(33.33)	4(3,5)	2.913	0.088
未行手术	68(66.67)	4(3,4)		
营养支持方式				
普通饮食	29(28.43)	3(2,4)[a,b]	18.577	<0.001
肠内营养	43(42.16)	4(3,5)[a]		
全肠外营养	15(14.71)	4(3,4)		
肠内联合肠外	15(14.71)	5(4,5)[b]		
皮质功能不全				
皮质功能不全	34(33.33)	5(4,6)	25.14	<0.001
未合并皮质功能不全	68(66.67)	3(3,4)		

注：营养支持方式进行两两比较时，有相同字母表示比较差异有统计学意义，普通饮食与肠内营养、肠内联合肠外比较差异有统计学意义(*P*<0.05)。

3.4.3 研究对象 D7 营养状况影响因素单因素分析

D7 天单因素分析以疾病严重程度、感染严重程度、机械通气、营养支持方式、合并皮质功能不全对营养状况有影响，差异有统计学意义(*P*<0.05)。而分别以社会人口学资料(年龄、性别、BMI 吸烟史、饮酒史、运动情况、是否有高血压、糖尿病、冠心病、其他慢性病)、手术单因素分析，差异无统计学意义(*P*>0.05)。

3.5 D1、D3、D7 营养状况多因素分析

分别以 D1、D3、D7 的 mNUTRIC 评分为因变量，分别 D1、D3、D7 单因素分析具有统计学意义的因素作为自变量，用多重线性回归分别 D1、D3、D7 对营养状况的影响

(C)1994-2023 China Academic Journal Electronic Publishing House. All rights reserved. http://www.cnki.net

因素进行分析，从表中得出D1、D3营养状况的影响因素均为合并感染性休克、APACHEII 评分≥15 分、合并皮质功能不全；D 7 天营养状况的影响因素为合并感染性休克、APACHEII 评分≥15 分。具体如表 15 所示：

表 15 脓毒症患者营养状况影响因素多重线性回归

时间	自变量	非标准化系数		标准系数	t	*P*	共线性统计量		R^2
		B	标准误差				容差	VIF	
D1	感染性休克	0.543	0.219	0.161	2.481	0.015	0.773	1.293	68.80%
	APACHEII 评分	0.199	0.019	0.708	10.744	<0.001	0.747	1.339	
	CIRCI	0.765	0.233	0.221	3.285	0.001	0.651	1.536	
D3	感染性休克	0.583	0.175	0.196	3.323	0.001	0.779	1.283	73.80%
	APACHEII 评分	0.161	0.017	0.601	9.662	<0.001	0.704	1.421	
	CIRCI	0.927	0.195	0.304	4.741	<0.001	0.663	1.509	
D7	感染性休克	0.575	0.189	0.181	3.05	0.003	0.877	1.140	70.20%
	APACHEII 评分	0.205	0.019	0.719	10.825	<0.001	0.703	1.422	

(C)1994-2023 China Academic Journal Electronic Publishing House. All rights reserved. http://www.cnki.net

讨论

脓毒症患者在经历了创伤、感染、重大手术、烧伤等打击后，使得机体处于高应激、高分解、高消耗代谢状态，而这些变化是由于机体内环境紊乱，神经系统、内分泌系统、交感系统及垂体肾上腺轴等被激活导致的[22]。严重应激后营养状况迅速下降是脓毒症患者普遍存在的现象。尽管营养需求增加，但脓毒症患者往往不愿(因为厌食症)或不能(因为脑病、机械通气、血流动力学不稳定)进食，这会导致严重的营养不良[23]。因此，对于脓毒症，临床营养是综合治疗的基本要素之一，精心策划并实施营养干预可改善患者预后[24]。正确的营养治疗始于对患者营养状况的充分评估和通过间接量热法结合其他监测工具进行的代谢评估。本研究评估患者在 D1、D3、D7 营养状况及分析影响脓毒症患者营养状况的因素，为合理的脓毒症营养支持提供理论依据。

在患者入 ICU D1、D3、D7 分别测量其清蛋白，经过治疗后前白蛋白和转铁蛋白较入 ICUD1 增加。前白蛋白(PA)是急性时相蛋白, 又称转甲状腺素蛋白，其半衰期约 1.9 天，在肝细胞合成。PA 在机体内水平受到多种因素共同影响，当机体肝脏功能受到损害时，体内的前白蛋白合成减少，同时 PA 能够迅速地反映人体内蛋白-能量的改变情况，也是机体内蛋白自主转换更新的良好指标之一，因此 PA 是目前国际上常常用以监测患者营养支持效果以及评价患者营养状况的重要指标之一[25]。转铁蛋白蛋白半衰期为 8 天，在肝脏内合成，人体储存量较少 也可较敏感的反应患者营养状况变化[26]。白蛋白和总蛋白在脓毒症患者 D1、D3、D7 无明显变化，可能原因如下：其一，白蛋白更新较低，半衰期 15 天，每天更替 3%，重症患者低白蛋白主要是血管内向细胞外液再分布或者大出血丢失的结果[27]。其二，当患者血清蛋白降低时，补充外源性人血白蛋白，这一定程度上影响了患者血清蛋白的水平；其三，当患者补充不同剂量的蛋白质、补充方式的不同也可影响患者血清蛋白水平。

与 D1 相比，血红蛋白和胆固醇在 D3、D7 水平有所下降。血红蛋白半衰期为 120 天左右，对于 ICU 患者血红蛋白变化，其受失血、贫血、溶血等情况的影响，慢性营养不良也可使其合成减少[28]。ICU 脓毒症患者营养状况是短期内发生改变的，在脓毒症 D1-

(C)1994-2023 China Academic Journal Electronic Publishing House. All rights reserved. http://www.cnki.net

D7 天时，其变化是否可代表营养状况还需进一步研究。本研究显示，在 D3 时胆固醇水平较入院有所降低，考虑胆固醇降低为大量消耗所致，感染、氧化应激也可使其合成减少[29]。与 D3 相比，在 D7 胆固醇无明显变化，其消耗降低，变化不明显。Haines 等人确定尿素/肌酐比(UCR)是危重病相关分解代谢的潜在生物标志物[30]。本研究中，尿素/肌酐在 D7 大于 D1，可间接反应在 D7 分解代谢入 ICU 明显增高，但尿素/肌酐可否直接反应脓毒患者的分解代谢增强，是否受其他因素影响，还需进一步研究。

将 D1、D3、D7 营养学指标与各阶段对应 mNUTRIC 得分进行相关分析。D1 内营养学指标与 mNUTRIC 评分无相关关系。D1 上述营养学指标的值不能反映脓毒症患者营养状况，上述营养学指标变化可能是由于炎症、应激、失血等因素引起。D3 mNUTRIC 得分与淋巴细胞绝对值、尿素/肌酐与存在相关关系，但相关关系并不密切。D7mNUTRIC 得分与白蛋白、总蛋白、血红蛋白、转铁蛋白存在相关关系，相关关系并不密切(相关关系小于 0.5)。脓毒症患者营养学指标受多种因素影响，如炎症、应激、高凝状态等因素，当上述指标变化时，因结合患者病情评估是否为营养不足引起，上述指标变化并不能完全代表营养状况变化。

利用代谢车监测患者 D1、D3、D7 静息能量消耗，结果显示随着住院时间的增加，患者 REE 逐渐下降，与国内外研究相似[31-33]，但各阶段比较无差异。入 ICU 后，经过临床干预，患者感染得到控制、血流动力学逐渐稳定、呼吸功能的得到改善，可降低患者静息能量消耗。研究 D1、D3、D7 呼吸商存在差异，RQ 的测量可能意味着哪些能量底物被按比例被利用[34]。在脓毒症急性期早期、急性期晚期、慢性期主要代谢物质不同。呼吸商(RQ)根据被代谢的主要底物而变化。脂肪、蛋白质和碳水化合物氧化的 RQ 分别为 0.7、0.82 和 1.0[14, 15]，

本研究显示，脓毒症在各阶段均存在营养不良风险，发生率最高为D1 内为42.15%，与国内外报道的 38%到 78%的重症监护室(ICU)患者在进入 ICU 时营养不良相似[35, 36]。给予脓毒症患者综合治疗后，脓毒症患者营养风险逐渐降低，第 3 天时为 38.24% ，D7 天时 28.43%。第 3 天至第 7 天营养不良风险下降最快，下降了 9.81%。经过 7 天综合治疗后仍然有 28.3%的患者存在营养不良风险，脓毒症患者营养治疗仍然是一个挑战。

(C)1994-2023 China Academic Journal Electronic Publishing House. All rights reserved. http://www.cnki.net

单因素分析显示疾病严重程度、合并脓毒症休克、合并皮质功能不全、机械通气对脓毒症患者 D1、D3、D7 天营养状况有影响。入 ICU 后给予不同的营养支持方式可影响 D1 和 D3 营养状况。进一步多因素分析分析显示疾病严重程度、合并感染性休克、合并皮质功能不全是脓毒症患 D1、D3 影响营养状况的因素。疾病严重程度、合并感染性休克脓毒症患 D7 影响营养状况的因素

证据表明，估计 50 %的患者在进入重症监护病房(ICU)时有肠细胞损伤，并且在 ICU 中大约 62%的患者出现胃肠道症状[37]。急性胃肠道损伤(Acute Gastrointestinal Injury，AGI)定义为患者的胃肠道功能障碍是由于重症急性疾病导致的，并由轻到重将 AGI 分成 4 级。对于重症监护室急性胃肠损伤(AGI)的患者，重度 AGI(III、IV)患者的病情较轻度 AGI(I、II)的患者重，两者 APACHE II 评分有差异[38]。患者发生急性胃肠道损伤(AGI)可出现喂养不耐受(Feeding Intolerance，FI)，导致营养相关并发症，最终导致营养吸收障碍。当给予肠内营养时，还可并发肠道并发症[39]，如呕吐、腹泻、胃肠道出血、吸入性肺炎、再喂养综合征或肠道缺血等[40]。有研究显示，当重症患者的疾病严重程度与胃排空直接相关[41]，者给予营养支持后，还可能存在高营养风险，这种影响持续存在脓毒症患者整个病程中。因此病情严重的患者并发重度 AGI，出现相关营养并发症，最终导致营养状况较差。

当患者发生脓毒症休克时，交感神经兴奋，全身血流重新分布，优先保证供应重要生命器官，胃肠道因其生理学特点成为最先受累的器官之一；胃肠道黏膜缺血缺氧、肠壁细胞代谢障碍、黏膜屏障功能破坏及通透性增加。胃肠黏膜屏障受损、肠道微生态紊乱等引起肠道细菌和内毒素移位，加重患者病情[42]。研究表明[43]，感染性休克患者喂养不耐受可达 34%-38%，提示感染性休克病人是肠内喂养不耐受的高危人群。感染性休克病人或者休克纠正后，行肠内营养治疗时因喂养不耐受导致患者无法达到喂养目标，导致喂养不足。

危重病相关肾上腺皮质功能不全 (CIRCI)表现为机体全身炎症反应失调，代谢障碍和循环衰竭。用于描述重症疾病的一种状态，在这种状态下，血浆皮质醇的浓度尽管高于正常，但仍不足以应对疾病引起的的应激。CIRCI 的发生率估计为 10% ～20%，

(C)1994-2023 China Academic Journal Electronic Publishing House. All rights reserved. http://www.cnki.net

而感染性休克患者 CIRCI 的发病率高达 60%。出现肾上腺皮质功能不全可表现为全身多系统的症状，包括：神经系统、心血管系统、呼吸系统、内环境紊乱等，其中消化系统表现为恶心、呕吐、不耐受肠内营养[44]，因此肾上腺皮质功能不全可通过影响消化系统进而影响患者营养状况。在 D7 天时，合并皮质功能不全患者为 29.41%，部分皮质功能不全得到纠正，对营养状况影响减少，在第 7 天患者皮质功能不全不是影响营养状况的因素。

本研究单因素分析显示，机械通气患者对 D1 内、D3 和 D7 天营造状况均有影响。机械通气患者通常使用镇痛药物，2018 中国成人镇静和镇痛治疗指南指出[45]，ICU 患者非神经性疼痛，阿片类为首选药物。外源性阿片类药物主要作用于肠壁平滑肌 μ 受体，μ 受体是 G 蛋白偶联受体超家族的一员。这种相互作用的结果是环磷酸腺苷和钙水平降低，兴奋性神经递质释放减少，最终导致胃肠蠕动减少[46]。机械通气治疗，患者胃肠道血液供应量减少，导致胃肠道黏膜缺氧缺血，在 EN 期间可诱发恶心呕吐、腹泻等胃肠不耐受的表现[47]。但是使用镇静、镇痛药物会降低患者的 REE，减少患者消耗。这种相互作用下，对于机械通气患者对营养状况存在两面的影响，多因素分析时机械通气不是影响营养因素之一。机械通气对患者营养状况影响好需要进一步研究。

单因素分析，D3 营养支持方式可影响患者营养状况，进行两两比较时，普通饮食的患者的营养状况好于肠内营养、肠内联合肠外营养。可能原因如下，普通饮食的患者可进口进食，无需进行机械通气，没有出现急性胃肠损伤，经口进食的营养物质可充分吸收。实施肠内营养的患者多为机械通气患者，不能进口进食，通过管饲实施肠内营养，机械通气患者由于镇静镇痛药物使用，可使用胃肠蠕动减慢，营养吸减慢。本研究普通饮食患者营养状况优于肠内联合肠外营养支持患者，研究对象实施肠内联合肠外患者胃肠功能尚可，但能量消耗较大或出现营养相关并发症，导致吸收障碍，肠内营养不能满足其营养需求，需通过肠外营养补充营养。在第 D7 天，普通饮食的患者的营养状况好于全肠外营养、肠内联合肠外营养。D7 天时，实行全肠外营养患者分为两大类，一是血流动力学不稳定，还需要大剂量血管活性药物维持循环；二则是存在消化道出血、肠瘘等肠内营养禁忌等情况，此类病人病情相对较重，因此行全肠外营养患者营养状况较差

(C)1994-2023 China Academic Journal Electronic Publishing House. All rights reserved. http://www.cnki.net

可能是由于病情严重以及胃肠功能衰竭相关。但多因素分析时营养支持方式不是影响营养因素之一，营养支持方式对脓毒症患者营养状况影响还需要进一步研究。

本研究存在一些不足：其他影响脓毒症患者营养状况的因素尚未纳入，比如能量摄入、蛋白质摄入、肝功能不全、急性肾功能衰竭等。

本研究创新之处：其一，依据脓毒症患者代谢特点分期，动态观察脓毒症患者的营养状况和持续评估患者营养风险，可对发现存在高营养风险的患者及时干预；其二，本研究采用了代谢车监测患者静息能量消耗，可精确测量患者实际需要的能量，可应用于临床指导能量摄入。

(C)1994-2023 China Academic Journal Electronic Publishing House. All rights reserved. http://www.cnki.net

结论

1.脓毒症患者在急性期早期、急性后期、恢复期均存在高营养风险；

2.脓毒症患者营养状况的影响因素为合并感染性休克、合并肾上腺皮质功能不全、APACHEII评分≥15。

(C)1994-2023 China Academic Journal Electronic Publishing House. All rights reserved. http://www.cnki.net

参考文献

[1]Singer M, Deutschman CS, Seymour CW, et al. The Third International Consensus Definitions for Sepsis and Septic Shock (Sepsis-3). *JAMA*. 2016;315(8):801-810.

[2]Fleischmann-Struzek C, Mellhammar L, Rose N, et al. Incidence and mortality of hospital- and ICU-treated sepsis: results from an updated and expanded systematic review and meta-analysis. *Intensive Care Med*. 2020;46(8):1552-1562

[3]McLaughlin J, Chowdhury N, Djurkovic S, et al. Clinical outcomes and financial impacts of malnutrition in sepsis. *Nutr Health*. 2020;26(3):175-178.

[4]Maicá AO, Schweigert ID. Nutritional assessment of the severely ill patient. Avaliação nutricional em pacientes graves. *Rev Bras Ter Intensiva*. 2008;20(3):286-295.

[5]Lindkvist B, Phillips ME, Domínguez-Muñoz JE. Clinical, anthropometric and laboratory nutritional markers of pancreatic exocrine insufficiency: Prevalence and diagnostic use. *Pancreatology*. 2015;15(6):589-597.

[6]Lambell KJ, Tatucu-Babet OA, Chapple LA, et al.. Nutrition therapy in critical illness: a review of the literature for clinicians. Crit Care. 2020;24(1):35.

[7]Singer P, Blaser AR, Berger MM, et al. ESPEN guideline on clinical nutrition in the intensive care unit. *Clin Nutr*. 2019;38(1):48-79.

[8]苏和毅,莫泽珣,陈珍等.ICU 严重免疫失衡疾病——持续炎症-免疫抑制-分解代谢综合征[J].中华危重病急救医学,2017,29(08):760-764.

[9]Cederholm T, Barazzoni R, Austin P, et al. ESPEN guidelines on definitions and terminology of clinical nutrition. *Clin Nutr*. 2017;36(1):49-64.

[10]刘朝晖, 苏磊, 廖银光,等. 脓毒症患者营养目标摄入对临床预后影响的前瞻性随机对照研究 [J]. 中华危重病急救医学, 2014, 26(3): 4.

[11]McClave SA, Martindale RG, Vanek VW, et al. Guidelines for the Provision and Assessment of Nutrition Support Therapy in the Adult Critically Ill Patient: Society of Critical

(C)1994-2023 China Academic Journal Electronic Publishing House. All rights reserved. http://www.cnki.net

Care Medicine (SCCM) and American Society for Parenteral and Enteral Nutrition (A.S.P.E.N.). *JPEN J Parenter Enteral Nutr*. 2009;33(3):277-316.

[12]Campbell K L , Lane K , Martin A D , et al Resting energy ex*p*enditure and body mass changes in women during adjuvant chemothera*p*y for breast cancer [J]. Cancer Nursing, 2007, 30(2): 95-100.

[13]Walker RN, Heuberger RA. Predictive equations for energy needs for the critically ill. *Respir Care*. 2009;54(4):509-521.

[14]Livesey G, Elia M. Estimation of energy expenditure, net carbohydrate utilization, and net fat oxidation and synthesis by indirect calorimetry: evaluation of errors with special reference to the detailed composition of fuels [published correction appears in Am J Clin Nutr 1989 Dec;50(6):1475]. *Am J Clin Nutr*. 1988;47(4):608-628.

[15]Nakaya Y, Harada N, Kakui S, et al. Severe catabolic state after prolonged fasting in cirrhotic patients: effect of oral branched-chain amino-acid-enriched nutrient mixture. *J Gastroenterol*. 2002;37(7):531-536.

[16]Kosałka K, Wachowska E, Słotwiński R. Disorders of nutritional status in sepsis - facts and myths. *Prz Gastroenterol*. 2017;12(2):73-82.

[17]Identifying critically ill *p*atients who benefit the most from nutrition thera*p*y: the develo*p*ment and initial validation of a novel risk assessment tool [J]. Critical Care,15,6(2011-11-15), 2011, 15(6): R268.

[18]Rahman A , Hasan R M , Agarwala R , et al. Identifying critically-ill *p*atients who will benefit most from nutritional thera*p*y: Further validation of the "modified NUTRIC" nutritional risk assessment tool [J]. Clinical Nutrition, 2016: 158-62.

[19]Surviving Se*p*sis Cam*p*aign: International Guidelines for Management of Se*p*sis and Se*p*tic Shock: 2016 [J]. Critical Care Medicine, 2017, 45.

[20]Taku, Oshima, Mette, et al. Indirect calorimetry in nutritional thera*p*y. A *p*osition *p*a*p*er by the ICALIC study grou*p* [J]. Clin Nutr, 2017, 36(3): 651-62.

(C)1994-2023 China Academic Journal Electronic Publishing House. All rights reserved. http://www.cnki.net

[21]Djillali, Annane, Stephen M , et al. Guidelines for the diagnosis and management of critical illness-related corticosteroid insufficiency (CIRCI) in critically ill patients (Part I): Society of Critical Care Medicine (SCCM) and European Society of Intensive Care Medicine (ESICM) 2017.[J] Intensive care medicine,2018,44(4).

[22]Latifi, Rifat. Nutritional Therapy in Critically Ill and Injured Patients [J]. Surgical Clinics of North America, 2011, 91(3): 579-93.

[23]Alberda C , Gramlich L , Jones N , et al. The relationship between nutritional intake and clinical outcomes in critically ill patients: results of an international multicenter observational study [J]. Intensive Care Medicine, 2009, 35(10): 1728.

[24]Elke G , Van Zanten A R H , Lemieux M , et al. Enteral versus parenteral nutrition in critically ill patients: an updated systematic review and meta-analysis of randomized controlled trials [J]. Critical Care, 2016, 20(1): 1-14.

[25]佚名. 观察前白蛋白在危重症患者营养支持和临床结局中的作用[J]. 临床医药文献电子杂志 [J]. 临床医药文献电子杂志, 2015, 2(21): 2.

[26]郑雪莲, 李玉珍, 朱刚. 血清前白蛋白,转铁蛋白及视黄醇结合蛋白在危重病人应用肠外营养支持中的意义 [J]. 海南医学, 2009, (S3): 2.

[27]Berger M M , Annika R B , Calder P C , et al. Monitoring nutrition in the ICU [J]. Clinical Nutrition, 2018: S0261561418312111-.

[28]Petrosyan I , Blaison G , Emmanuel Andrès, et al. Anaemia in the elderly: an aetiologic profile of a prospective cohort of 95 hospitalised patients [J]. European Journal of Internal Medicine, 2012, 23(6).

[29]刘忠青, 卢昌政, 王先林. 危重患者低胆固醇血症与疾病严重程度关系及对预后的影响 [J]. 中国实用医药, 2015, (27): 3.

[30]Gunst J , Kashani K B , Hermans G The urea-creatinine ratio as a novel biomarker of critical illness-associated catabolism [J]. Intensive Care Medicine, 2019, 45(12): 1813-5.

[31]石俊, 席力罡, 迟天航, 等.静息能量监测在机械通气患者营养支持治疗中的应用价

(C)1994-2023 China Academic Journal Electronic Publishing House. All rights reserved. http://www.cnki.net

值 [J]. 中华危重病急救医学, 2019, 31(1): 4.

[32]赵士兵, 段立彬, 余刚,等. 应用代谢车监测 ICU 患者 REE 变化规律并指导营养支持的前瞻性研究 [J]. 中华危重病急救医学, 2019, 31(12): 5.

[33]Tatucu-Babet O A , Ridley E J , Tierney A C . *P*revalence of Under*p*rescri*p*tion or Over*p*rescri*p*tion of Energy Needs in Critically Ill Mechanically Ventilated Adults as Determined by Indirect Calorimetry: A Systematic Literature Review [J]. J*p*en J *P*arenter Enteral Nutr, 2015, 40(2): 212-25.

[34]Sornwichate Rattanachaiwong,Pierre Singer. Indirect calorimetry as point of care testing[J]. Clinical Nutrition,2019,38(6).

[35]Ceniccola G D , Holanda T P , Pequeno R , et al. Relevance of AND-ASPEN criteria of malnutrition to predict hospital mortality in critically ill patients: A prospective study[J]. Journal of Critical Care, 2017:398.

[36]Lew C , Yandell R , Fraser R , et al. Association Between Malnutrition and Clinical Outcomes in the Intensive Care Unit: A Systematic Review [J]. J*p*en Journal of *P*arenteral & Enteral Nutrition, 2016, 41(5).

[37]Piton G , Belon F , Cypriani B , et al. Enterocyte damage in critically ill patients is associated with shock condition and 28-day mortality[J]. Critical Care Medicine, 2013, 41(9):2169-2176.

[38]孙丽娟, 薛森海, 闫凤,等. 预消化的肠内营养对 ICU 危重症患者的营养状况及肠内营养耐受性的影响 [J]. 现代生物医学进展, 2019, 19(10): 6.

[39]Hsu C W , Sun S F , Lin S L , et al. Duodenal versus gastric feeding in medical intensive care unit patients: a prospective, randomized, clinical study.[J]. Critical Care Medicine, 2009, 37(6):1866.

[40]Joseph, I, Boullata, et al. ASPEN Safe Practices for Enteral Nutrition Therapy[J]. Journal of Parenteral & Enteral Nutrition, [J]. J*p*en J *P*arenter Enteral Nutr, 2017, 41(1): 15.

[41]Aderinto-Adike A O , Quigley E . Gastrointestinal motility problems in critical care: a

(C)1994-2023 China Academic Journal Electronic Publishing House. All rights reserved. http://www.cnki.net

clinical perspective.[J]. Journal of Digestive Diseases, 2014, 15(7):335-344.

[42]吴云, 李学兵, 胡军,等. 脓毒症急性胃肠损伤研究进展 [J]. 中医临床研究, 2020, 12(10): 4.

[43]李龙, 王雨霏. 感染性休克病人喂养不耐受的研究进展 [J]. 护理研究, 2020, 34(22): 3.

[44]王妍, 朱丹丹, 于健. 危重病相关肾上腺皮质功能不全的研究进展 [J]. 实用休克杂志:中英文, 2018, 002(005): 296-300.

[45]佚名. 中国成人 ICU 镇痛和镇静治疗指南[J]. 中华重症医学电子杂志(网络版), 2018, 004(002):90-113.

[46]Kurz A,SesslerD.Opioid-Induced Bowel Dysfunction[J]. Drugs, 2003, 63(7):649-671.

[47]龚年金.中医辨证治疗急性肺损伤/急性呼吸窘迫综合征机械通气患者胃肠功能障碍疗效观察 [J]. 现代中西医结合杂志, 2017, 26(15): 3.

(C)1994-2023 China Academic Journal Electronic Publishing House. All rights reserved. http://www.cnki.net

文献综述

脓毒症患者营养支持研究进展

【摘要】脓毒症(sepsis)是严重感染引起的宿主反应失控导致严重器官功能障碍。脓毒症除了外科干预、液体复苏、抗菌药物治疗、血糖控制、激素治疗、凝血功能改善、机械通气等手段外，营养支持治疗是脓毒症治疗的重要手段。营养支持是要维持细胞的代谢,保持组织器官的结构与功能,进而调整免疫内分泌功能与修复组织,促使病人康复。营养支持不再停留于维持机体氮平衡,保持病人的体重。规范的营养治疗对于临床医师有重要意义，但目前脓毒症患者营养支持尚有争议。本文就脓毒症营养支持研究进展进行综述。

【关键词】脓毒症　营养支持　研究进展

1 脓毒症代谢特点

人体新陈代谢的特点是同步的分解代谢和合成代谢的过程，这不仅使细胞保持体内平衡，而且对其微环境做出反应。这种代谢平衡在脓毒症中被破坏。脓毒症被定义为“宿主对感染的反应失调引起的危及生命的器官功能障碍”[1]。目前的定义不仅关注炎症，还指出体内平衡的系统性障碍以及新陈代谢。脓毒症导致机体功能障碍和线粒体损伤，这被认为是这些患者细胞代谢紊乱的主要原因。

2 营养支持方式以及时机

2.1 肠内营养

《中国严重脓毒症/脓毒性休克治疗指南 (2014)》[2]和 ASPEN 的《2016 年成人危重症病人营养支持治疗实施与评价指南》都指出对于感染性休克患者，当患者液体复苏成功且血流动力学稳定后考虑启动肠内营养，小剂量血管活性药物不是使用早期肠内营养的禁忌证，应在入 ICU 后的前 24-48 小时内尽早开始。在接下来的 48-72 小时内，喂养量应该逐步向目标推进[3]。2017 年《重症患者早期肠内营养:ESICM 临床实践指南》和 2019《ESPEN 重症监护病房临床营养指南》均指出对于能进食的危重患者，口服优于肠内或者肠外营养。对于不能进食的患者应早期启动肠内营养(48 小时内)，而不是延迟

(C)1994-2023 China Academic Journal Electronic Publishing House. All rights reserved. http://www.cnki.net

肠内营养[4, 5]。综上所述，四项国内外临床指南均建议无禁忌症患者早期(48h 内)启动肠内营养。肠内营养维持肠上皮细胞的生长、稳态、功能及调节肠上皮屏障有重要作用。维持肠道免疫的正常功能也离不开肠内营养，肠内营养补充有可能改善胃肠疾病患者的粘膜异常[6]。王熙等人研究，纳入 1733 脓毒症患者比较肠内营养(EN)及肠外营养(PN)对脓毒症患者相关临床指标的改善及临床结局的异同。结果显示，与肠外营养相比，应用肠内营养可改善脓毒症患者感染及脏器损伤程度，降低呼吸机使用时间、ICU 住院率及改善临床结局[7]。然而当患者出现肠梗阻、明显肠缺血、严重的休克、高流量的瘘、严重的肠道出血及腹腔间室综合征等需要考虑延迟肠内营养[8, 9]。

2.2 肠外营养适应症以及时机

当脓毒症患者出现肠内营养禁忌症或肠内营养不能满足能量需求时可启动肠外营养(PN)。目前启动肠外营养的时机尚有争议。在确定肠内营养耐受不良的前提下，如果存在营养风险或者低喂养，则需启动肠外营养治疗。当肠内喂养不足时何时添加肠外营养有一定争议，早年几项大型研究显示早期添加肠外营养可能会增加感染并发症和住院时间，但如果延迟添加肠外营养也可能导致营养不足和不良临床结局[10]。Michael P. Casaer 等人进行随机、多中心试验中，比较了重症监护室成人早期开始肠外营养(48 小时内开始)和晚期开始肠外营养(入 ICU 之后第 8 天)以补充不足的肠内营养，结果显示晚期启动组患者更早从重症监护室存活出院的可能性相对增加 6.3%，晚期开始组患者的重症监护病房感染较少、胆汁淤积发生率较低、需要 2 天以上机械通气的患者比例相对降低了 9.7%、肾脏替代治疗持续时间中值降低了 3 天、医疗保健费用平均降低了 (约 1，600 美元)。结论，与早期开始相比，晚期开始肠外营养与更快的恢复和更少的并发症相关[11]。瑞典的一项研究表明，如果肠内营养的能量充分利用率低于 60%，早期补充 PN 是有益的[12]。

3 脓毒症患者能量和蛋白质需求

3.1 能量评估

对能量摄入量的计算是营养治疗的第一步，目前脓毒症能量摄入主要依据公式计算，由于机械通气、镇静镇痛药物使用、合并基础疾病以及疾病的可影响脓毒症患者静息能

(C)1994-2023 China Academic Journal Electronic Publishing House. All rights reserved. http://www.cnki.net

量消耗,公式法因系数固定不能准确预测患者的静息能量消耗。并且重症患者病情变化较快，需要持续评估其能量变需求 [13]。2016 年 ASPEN 重症营养指南及 2018 ESPEN 指南均推荐，可使用间接测热法测定能量消耗(REE)以此指导营养支持治疗。无法使用间接测热法时，建议基于体重简化公式 25-30kcal/kg/确定能量需求[5, 14]。

3.2 蛋白质需求

目前并没有临床实用方法测量重症病人的蛋白质需求，主要是根据理想、实际或调整后的体质量测量进行理论估算。重症病人常缺乏蛋白质，但是蛋白质在重症不同时期的需求量并不清楚。 全球营养学会基于薄全弱的证据提供了不同的建议(1.2-2.5 g/kg/天)[5, 14-16]。目前对于危重患者最佳蛋白质摄入计量仍然有争议。蛋白质摄入过低以及过高均影响患者预后。有研究报道，对入住 ICU 的老年危重患者，依据 1 周内蛋白质供给量是否达到 1.2 g/(kg·d)进行分组，分为高蛋白组(蛋白质供给量≥1.2 g/(kg·d)和非高蛋白组(蛋白质供给量＜1.2 g/)，研究显示通过增加蛋白质摄入量可以明显改善老年重症患者临床营养状况，缩短机械通气时间及住院时间并降低 28 d 病死率。此研究高蛋白组蛋白质剂量范围在 1.2~2.0 g/(kg·d)，并发现增加蛋白质摄入量可改善预后，但是对于老年重症患者蛋白质摄入量超过这一范围后是否越多越好仍不明确，最佳摄入剂量还需要进一步研究[17]。Shinya Matsushima 等人研究显示，依据后入 ICU 后 7 天内是否达到基于入 ICU 体重的 1.0 g/kg/d 蛋白质分为<1.0 g/kg/d (高蛋白组)或< 1.0 g/kg/d(低蛋白组)，得出结论:足量的蛋白质摄入可能会导致独立行走的较高恢复率[18]。基于脓毒症患者分解代谢大于合成代谢的特点，有人提出了高达 2.5g/kg/d 的蛋白质摄入[19]。然而过多的蛋白质抑制自噬并有可能导致危重症肌病[20]。

4 喂养方式

早期营养支持时的营养液输注途径和方法可影响肠内营养的效果，恰当的营养输注方式可避免喂养不耐受和营养相关并发症。肠内营养支持主要通过连续喂养或间断喂养的方式，但采用哪种方法喂养对患者更有利仍有争议。持续喂养对于肠道吸收能力差或胃肠道功能障碍的患者有较好的耐受性，临床实践中应用较多，但有喂养时间较长、不能有效刺激胃肠激素分泌等缺点[21]。间断喂养可以建立胃肠激素间歇分泌的模式，这更

(C)1994-2023 China Academic Journal Electronic Publishing House. All rights reserved. http://www.cnki.net

有利于建立消化道吸收的基本生理环境，但是可能使导管脱位、腹胀等风险增加[22]。研究显示，间断喂养可以及减少危重患者肠内营养并发症、可减少患者胃残余量异常增多的肠内营养不耐受症状[23-25]。综上所述，建议脓毒症采取间断喂养的方式实施肠内营养。

5 微量营养素以及特定营养物质的补充

5.1 微量营养素

微量营养素在代谢过程中可抗氧化和免疫调节，通常为酶的必须成分以及或辅助因子，它包括微量元素和维生素。

微量元素指生物体所必需的一些元素，占生物体总质量 0.01%以下，如铁、硅、锌、铜、碘、溴、硒、锰等。微量元素参与许多重要的生理过程，如蛋白质结合调节代谢、作为金属酶的辅助因子，微量元素缺乏会导致细胞功能和微循环失调 [26]。血清微量元素影响免疫系统细胞的功能，如淋巴细胞和粒细胞，一些免疫和炎症反应可以改变这些化学物质在体内的分布[27]。ICU 的患者微量营养素水平与疾病严重程度、CRP 或微量营养素摄入量之间没有关联[28]。有文献显示，外科脓毒症患者血清锌水平降低与脓毒症复发易感性相关。此外，外科脓毒症患者在第 28 天和第 90 天出现器官功能障碍并增加院内死亡率的情况显示入 ICU 时血清锌水平较低[29]。有研究显示， 脓毒症患者入 ICU 后补充锌可减少感染的发生，降低死亡率，有利于新生儿神经发育[30]。有文献报道，给予危重患者补充不同剂量的硒、以及给药时机产生不同的临床结局。高剂量硒可增加重症监护室住院天数，但低剂量硒可减少重症监护室患者急性肾功能衰竭的发生[31]。

5.2 维生素

5.2.1 硫胺素

硫胺素是焦磷酸硫胺素(TPP)的前体，焦磷酸硫胺素是柠檬酸循环、ATP 生成、戊糖磷酸途径、葡萄糖代谢和 NADPH 生成所需的几种脱羧酶的必需辅酶[32, 33]。有文献报道，20-70%的感染性休克患者存在硫胺素缺乏[33]。有荟萃分析显示，在 RCT 中，危重患者补充硫胺素可降低 ICU 谵妄的发生率。然而，就总死亡率和脓毒症症患者的死亡率而言，没有显着益处[34]。Jordan A. Woolum 等[35]研究显示对于住进 ICU 的脓毒性休克患者，与未接受硫胺治疗的配对队列患者相比，在 D1 内接受硫胺治疗的患者与乳酸清除

(C)1994-2023 China Academic Journal Electronic Publishing House. All rights reserved. http://www.cnki.net

率提高和 28 天死亡率降低相关。

5.2.2 维生素 C

维生素 C 为水溶性抗氧化剂。可直接清除自由基和回收其他抗氧化剂，并通过促进胶原蛋白合成和维持内皮血管舒张和屏障功能来保护血管内皮。 它是神经递质(去甲肾上腺素、血清素)、皮质醇、肽类激素(血管加压素)和胶原蛋白生物合成的辅因子和共底物[36]。有研究显示脓毒症患者静脉注射每 6 小时静脉注射 25mg/kg 维生素 C，对比安慰剂组，实验组去甲肾上腺素剂量、给药时间以及 ICU 死亡率有统计学意义。认为大剂量抗坏血酸可作为一种安全有效的辅助用药[37]。有回顾性队列研究显示，入住 ICU 且需要机械通气的脓毒症或者感染性休克患者，静脉注射维生素 C(每 8 小时 2 克)的情况，研究患者被分为维生素 C 组或对照组，两组的住院死亡率、重症监护室入 ICU 后 90 天的死亡率没有显著差异。两组休克逆转的中位时间均为 3 天[(IQR)，2 至 5 天]。两组在重症监护室前 4 天脓毒症相关器官衰竭评估评分的变化分别为 1.4±3.3 和 1.4±3.0。结论:在机械通气的脓毒症或脓毒性休克患者中，单独辅助静脉维生素 C 治疗不能降低医院死亡率[38]。2021 脓毒症休克指南尚不推荐静脉注射维生素 C 治疗用于脓毒症辅助治疗。

5.2.3 维生素 D

维生素 D 是机体抵抗感染的核心调控因子。在人类固有免疫系统和天然黏膜屏障功能中，活性维生素 D 触发抗微生物信号通路，提高机体细胞免疫功能，对抗和抑制细菌、真菌、病毒等病原微生物引发的感染。同时，活性维生素 D 也调控超敏反应，减轻器官、组织免疫损伤等病理生理过程[39]。Al-TarrahK 等人[40]研究指出，维生素 D 缺乏与疾病的严重程度及病死率相关。有研究显示，补充维生素 D 可改善维生素 D 严重缺乏者 ARDS 的严重程度[41]。也有报道分析和系统综述研究表明，补充维生素 D 对危重患者的临床结局没有影响[42]。补充维生素 D 对危重患者炎症影响方面，少数临床研究的证据表明，高剂量的维生素 D 干预可能会减少促炎细胞因子，而对抗炎细胞因子和 C 反应蛋白水平似乎没有显著影响。需要进一步的研究来阐明补充维生素 D 对危重患者免疫反应的影响[43]。维生素 D 缺乏被证实是危重患者发生死亡的独立危险因素，补充维生素 D 可降低死亡率。但是补充剂量以及时机目前未有准确界定，存在较大争议，需更多研

(C)1994-2023 China Academic Journal Electronic Publishing House. All rights reserved. http://www.cnki.net

究明确。

5.2.4 定营养物质: 谷氨酰胺和 n-3 脂肪酸

谷氨酰胺是一种条件性必需氨基酸，在脓毒症、创伤和术后等情况下，它的消耗会增加。大量研究表明，氨基酸对细胞免疫具有稳态调节作用。在体外实验中发现，谷氨酰胺可以减少促炎性白细胞介素-6 和白细胞介素-8 的产生，并提高 T 淋巴细胞、B 淋巴细胞和上皮细胞的抗炎性白细胞介素-10 水平[44]。此外，谷氨酰胺可以潜在地调节先天和适应性免疫反应，因为白细胞介素-10 在维持肠粘膜稳态中起着重要作用[45]。换句话说，T 细胞激活需要谷氨酰胺，淋巴细胞活化导致代谢重编程，其中谷氨酰胺代谢及其在炎症期间的需求在特定细胞群中增加。随着脓毒症化患者肌肉质量的损失，谷氨酰胺的产生可能与免疫细胞、肠细胞和肝细胞增加的谷氨酰胺需求不匹配。[46]一项涉及总共485名患者的早期随机对照试验的荟萃分析表明，补充谷氨酰胺可能会降低感染风险、住院时间和死亡风险[47]。入 ICU 时血浆谷氨酰胺水平降低被反复证明是 ICU 患者死亡的独立危险因素。另一个极端是，谷氨酰胺水平很高同样与预后不佳有关[48]。目前尚不清楚谷氨酰胺的最佳摄入计量，因此不应当盲目给予谷氨酰胺。

n- 3 系多不饱和脂肪酸(n～3 PuFAs)是一类包含多个双键且第一个双键出现在碳链甲基端第 3 位的脂肪酸，是人体生长和健康所必需的物质。能抑制血小板聚集，延缓血栓形成，改善微循环的功能，对防治心脑血管的栓塞有显著的作用[49]。在危重患者中，n-3 多不饱和脂肪酸可以改变细胞因子的产生，调节炎症和免疫应答[50]。一项 mate 分析表明富含 n-3 多不饱和脂肪酸的肠外营养方案在降低重症监护病房患者的感染率 ICU 住院时间方面是安全有效的[51]。有文献报道，脓毒症患者肠外营养额外添加 n-3 脂肪酸组死亡率降低，急性生理学和慢性健康评估评分也快速下降[52]。

微量营养素对脓毒症发生发展起重要作用，过高或者过低均会引起相关并发症。补充微量营养素能否改变脓毒症的最终死亡率存在争议，还需要更多的研究。

展望

脓毒症患者的营养支持仍然是一个挑战，部分营养物质的用法存在争议。整合蛋白质组学、代谢组学、转录组学、和微生物组学相互作用的纵向评估的多组学方法为将为脓

(C)1994-2023 China Academic Journal Electronic Publishing House. All rights reserved. http://www.cnki.net

毒症患者营养支持提供精确的指导。在大量人群中同时检查多种代谢途径的选择现在更可行，并且可能允许对脓毒症中发生的代谢变化有更细致入微的了解。随着这些数据在未来几年的出现，开发一种更个体化的方法来治疗脓毒症患者的代谢和营养可能成为可能。

(C)1994-2023 China Academic Journal Electronic Publishing House. All rights reserved. http://www.cnki.net

综述参考文献

[1] Singer M, Deutschman CS, Seymour CW, et al. The Third International Consensus Definitions for Sepsis and Septic Shock (Sepsis-3). JAMA. 2016;315(8):801-810.

[2]中国严重脓毒症/脓毒性休克治疗指南(2014)[J].中华内科杂志,2015,54(06):557-581.

[3]Warren M, McCarthy MS, Roberts PR. Practical Application of the Revised Guidelines for the Provision and Assessment of Nutrition Support Therapy in the Adult Critically Ill Patient: A Case Study Approach. *Nutr Clin Pract*. 2016;31(3):334-341.

[4]Reintam Blaser A, Starkopf J, Alhazzani W, et al. Early enteral nutrition in critically ill patients: ESICM clinical practice guidelines. *Intensive Care Med*. 2017;43(3):380-398.

[5]SINGER P, BLASER A R, BERGER M M, et al. ESPEN guideline on clinical nutrition in the intensive care unit [J]. Clin Nutr, 2019, 38(1): 48-79.

[6]Farré R, Fiorani M, Abdu Rahiman S, Matteoli G. Intestinal Permeability, Inflammation and the Role of Nutrients. *Nutrients*. 2020;12(4):1185. [J]. Nutrients, 2020, 12(4).

[7]王熙,刘振密,谢尹晶,等.肠内外营养与脓毒症预后的关系：一项回顾性现实世界研究[J].罕少疾病杂志,2021,28(04): 63-6.

[8]Weimann A, Braga M, Carli F, et al. ESPEN practical guideline: Clinical nutrition in surgery. *Clin Nutr*. 2021;40(7):4745-4761.

[9]Reintam Blaser A, Starkopf J, Alhazzani W, et al. Early enteral nutrition in critically ill patients: ESICM clinical practice guidelines. *Intensive Care Med*. 2017;43(3):380-398.

[10]Doig G S , Simpson F . Early parenteral nutrition in critically ill patients with short-term relative contraindications to early enteral nutrition: a randomized controlled trial.[J]. Jama, 2013, 2013(default):2130-2138.

[11]Heyland DK, Dhaliwal R, Drover JW, Gramlich L, Dodek P; Canadian Critical Care Clinical Practice Guidelines Committee. Canadian clinical practice guidelines for nutrition support in mechanically ventilated, critically ill adult patients. *JPEN J Parenter Enteral Nutr*.

(C)1994-2023 China Academic Journal Electronic Publishing House. All rights reserved. http://www.cnki.net

2003;27(5):355-373.

[12]Heidegger CP, Berger MM, Graf S, et al. Optimisation of energy provision with supplemental parenteral nutrition in critically ill patients: a randomised controlled clinical trial. *Lancet*. 2013;381(9864):385-393.

[13]Oshima T, Berger MM, De Waele E, et al. Indirect calorimetry in nutritional therapy. A position paper by the ICALIC study group. *Clin Nutr*. 2017;36(3):651-662.

[14]McClave SA, Martindale RG, Vanek VW, et al. Guidelines for the Provision and Assessment of Nutrition Support Therapy in the Adult Critically Ill Patient: Society of Critical Care Medicine (SCCM) and American Society for Parenteral and Enteral Nutrition (A.S.P.E.N.). *JPEN J Parenter Enteral Nutr*. 2009;33(3):277-316.

[15]Sioson MS, Martindale R, Abayadeera A, et al. Nutrition therapy for critically ill patients across the Asia-Pacific and Middle East regions: A consensus statement. *Clin Nutr ESPEN*. 2018;24:156-164.

[16]Elke G, Hartl WH, Kreymann KG, et al. Clinical Nutrition in Critical Care Medicine - Guideline of the German Society for Nutritional Medicine (DGEM). *Clin Nutr ESPEN*. 2019;33:220-275.

[17]姚哲放,王美霞,赵兰,等.老年重症患者蛋白质供给量与预后的相关性 [J].中华临床医师杂志(电子版),2021,15(05):347-52.

[18]Matsushima S, Yoshida M, Yokoyama H, et al. Effects on physical performance of high protein intake for critically ill adult patients admitted to the intensive care unit: A retrospective propensity-matched analysis. *Nutrition*. 2021;91-92:111407.

[19]Singer P, Hiesmayr M, Biolo G, et al. Pragmatic approach to nutrition in the ICU: expert opinion regarding which calorie protein target. *Clin Nutr*. 2014;33(2):246-251.

[20]Hermans G, Casaer MP, Clerckx B, et al. Effect of tolerating macronutrient deficit on the development of intensive-care unit acquired weakness: a subanalysis of the EPaNIC trial. *Lancet Respir Med*. 2013;1(8):621-629.

(C)1994-2023 China Academic Journal Electronic Publishing House. All rights reserved. http://www.cnki.net

[21]卢孔渺,黄曼.重症患者的肠内营养喂养方式：间断喂养,还是持续喂养[J]. 中华急诊医学杂志,2020,(01):137-8-9-40.

[22]Palakshappa JA, Reilly JP, Schweickert WD, et al. Quantitative peripheral muscle ultrasound in sepsis: Muscle area superior to thickness. *J Crit Care*. 2018;47:324-330.

[23]吴国庆,曹群,茅益东,等.不同鼻饲喂养方式在重症脑卒中患者中的应用比较[J].现代医学与健康研究电子杂志,2019,3(17):2.

[24]董敬之,刘睿,李璐,等.间断喂养与持续喂养对危重症患者肌肉萎缩和营养状态的影响[J].中华危重病急救医学,2021,33(7):5.

[25]于安山,邹志胜,王春丽,等.成人重症患者经鼻胃管持续喂养与间断喂养效果比较的Meta 分析[J].江西医药, 2020,55(11):5.

[26]李锐,何先弟.脓毒症的发生发展与微量营养素的相关性研究进展 [J]. 中国医师杂志,2020,22(5):5.

[27]Gammoh NZ, Rink L. Zinc in Infection and Inflammation. *Nutrients*. 2017;9(6):624.

[28]Koekkoek WAC, Hettinga K, de Vries JHM, van Zanten ARH. Micronutrient deficiencies in critical illness. *Clin Nutr*. 2021;40(6):3780-3786.

[29]Janine H , Tim-Philipp S , Thorben B , et al. Persistent low serum zinc is associated with recurrent sepsis in critically ill patients - A pilot study[J]. Plos One, 2017, 12(5):e0176069.

[30]Newton B , Ballambattu V B , Bosco Dhas B , et al. Efficacy of zinc supplementation on serum calprotectin, inflammatory cytokines and outcome in neonatal sepsis - A randomized controlled trial[J]. Journal of Maternal-Fetal and Neonatal Medicine, 2017;30(13):1627-1631.

[31]Mousavi MA, Saghaleini SH, Mahmoodpoor A, Ghojazadeh M, Mousavi SN. Daily parenteral selenium therapy in critically ill patients: An updated systematic review and meta-analysis of randomized controlled trials. *Clin Nutr ESPEN*. 2021;41:49-58.

[32]Leite HP, de Lima LF. Metabolic resuscitation in sepsis: a necessary step beyond the hemodynamic?. *J Thorac Dis*. 2016;8(7):E552-E557.

[33]Mallat J , Lemyze M , Thevenin D . Do not forget to give thiamine to your septic shock

(C)1994-2023 China Academic Journal Electronic Publishing House. All rights reserved. http://www.cnki.net

patient![J]. Journal of Thoracic Disease, 2016, 8(6):1062.

[34]Sedhai YR, Shrestha DB, Budhathoki P, et al. Effect of thiamine supplementation in critically ill patients: A systematic review and meta-analysis. *J Crit Care*. 2021;65:104-115.

[35]Woolum JA, Abner EL, Kelly A, Thompson Bastin ML, Morris PE, Flannery AH. Effect of Thiamine Administration on Lactate Clearance and Mortality in Patients With Septic Shock. *Crit Care Med*. 2018;46(11):1747-1752.

[36]Oudemans-van Straaten HM, Spoelstra-de Man AM, de Waard MC. Vitamin C revisited. *Crit Care*. 2014;18(4):460.

[37]Zabet MH, Mohammadi M, Ramezani M, Khalili H. Effect of high-dose Ascorbic acid on vasopressor's requirement in septic shock. *J Res Pharm Pract*. 2016;5(2):94-100.

[38]Ahn J H , Dong K O , Jin W H , et al. Vitamin C alone does not improve treatment outcomes in mechanically ventilated patients with severe sepsis or septic shock: A retrospective cohort study[J]. Journal of Thoracic Disease, 2019, 11(4):1562-1570.

[39]张磊,董砚虎.维生素 D 抗感染机制研究进展[J].中华内分泌代谢杂志, 2021,37(2):4.

[40]Khaled A T , Martin H , Naiem M , et al. Vitamin D status and its influence on outcomes following major burn injury and critical illness[J]. Burns & Trauma, 2018:11.

[41]李娜,王美霞,周晋萌,等.维生素 D 对脓毒性休克导致急性呼吸窘迫综合征患者的干预价值研究[J].中华危重症医学杂志：电子版,2020,13(5):7.

[42]Zhang M , Jativa D F . Vitamin C supplementation in the critically ill: A systematic review and meta-analysis[J]. SAGE Open Medicine, 2018, 6:205031211880761.

[43]Sma A , Lsb A , Gr B , et al. The effect of vitamin D supplementation on inflammation in critically ill patients: A systematic review - ScienceDirect[J]. PharmaNutrition, 2020, 13.

[44]Coffier M , Marion R , P Ducrotté, et al. Modulating effect of glutamine on IL-1β-induced cytokine production by human gut[J]. Clinical Nutrition, 2003, 22(4):407-413.

[45]Mantovani A, Marchesi F. IL-10 and macrophages orchestrate gut homeostasis. *Immunity*. 2014;40(5):637-639.

(C)1994-2023 China Academic Journal Electronic Publishing House. All rights reserved. http://www.cnki.net

[46]Carr EL, Kelman A, Wu GS, et al. Glutamine uptake and metabolism are coordinately regulated by ERK/MAPK during T lymphocyte activation. *J Immunol*. 2010;185(2):1037-1044.

[47]Novak F , Heyland D K , Avenell A , et al. Glutamine supplementation in serious illness: a systematic review of the evidence.[J]. Critical Care Medicine, 2002, 30(9):2022.

[48]Rodas P , Rooyackers O , Hebert C , et al. Glutamine and glutathione at ICU admission in relation to outcome[J]. Clinical Science, 2012, 122(12):591-597.

[49]陈蝶玲,黄巍峰,郑晓辉,等.N3 系多不饱和脂肪酸膳食参考摄入量的研究进展[J].食品工业科技,2015,36(11):378-88.

[50]Mayer K , Meyer S , Reinholz-Muhly M , et al. Short-time infusion of fish oil-based lipid emulsions, approved for parenteral nutrition, reduces monocyte proinflammatory cytokine generation and adhesive interaction with endothelium in humans.[J]. Journal of Immunology, 2003, 171(9):4837-43.

[51]Pradelli L, Mayer K, Muscaritoli M, Heller AR. n-3 fatty acid-enriched parenteral nutrition regimens in elective surgical and ICU patients: a meta-analysis [published correction appears in Crit Care. 2012;17(1):405]. *Crit Care*. 2012;16(5):R184.

[52]Mayer K, Schaefer MB, Hecker M. Intravenous n-3 fatty acids in the critically ill. *Curr Opin Clin Nutr Metab Care*. 2019;22(2):124

(C)1994-2023 China Academic Journal Electronic Publishing House. All rights reserved. http://www.cnki.net

附录

附表 1 APACHEⅡ评分标准

			0 分	1 分	2 分	3 分	4 分
A、生理学评分	1.T(℃)		36.0-38.4	34-35.9 或 38.5-38.9	32-33.9	30-31.9 或 39-40.9	≤29.9 或≥41
	2.MAP(mmHg)		70-109		50-69 或 110-129	130-159	≤49 或≥160
	3.HR(次/min)		70-109		55-69 或 110-139	40-54 或 140-179	≤39 或≥180
	4.RR(次/min)		12-24	10-11 或 25-34	6-9	35-49	≤5 或≥50
	5. 二选一	FiO2<0.5 时测 PaO2	>70	61-70		55-60	<55
		FiO2>0.5 时测 AaDO2	<200		200-349	350-499	≥500
	6	动脉血 pH	7.33-7.49	7.5-7.59	7.25-7.32	7.15-7.24 或 7.60-7.69	≥7.7 或<7.15
	7.Na+(mmol/L)		130-149	150-154	120-129 或 155-159	111-119 或 160-179	≤110 或≥180
	8.K+(mmol/L)		3.5-5.4	3.0-3.4 或 5.5-5.9	2.5-2.9	6.0-6.9	<2.5 或≥7
	9.Cr(umol/L)		53-127		<53 或 128-171	172-304	≥305
	10.HCT(%)		30-45.9	46-49.9	20-29.9 或 50-59.9		<20 或≥60
	11.WBC(*10^9/L)		3-14.9	15-19.9	1-2.9 或 20-39.9		<1 或≥40
	12.15-GCS 评分						
B.年龄评分			0 分	2 分	3 分	5 分	6 分
			≤44 岁	45-54 岁	55-64 岁	65-74 岁	≥75 岁
C.慢性健康状况评分(有严重器官系统功能不全或免疫损害)			2 分		5 分		0 分
			非手术或择期手术后		不能手术或急诊手术后		无上述情况

备注：APACHE II评分=A+B+C

(C)1994-2023 China Academic Journal Electronic Publishing House. All rights reserved. http://www.cnki.net

附表 2 序贯器官衰竭（SOFA）评分

变量	0	1	2	3	4
PaO_2/FiO_2,mmHg	>400	≤150	≤150	≤150	≤150
呼吸机支持				是	是
血小板,10^9/L	>150	≤150	≤100	≤50	≤20
胆红素,umol/L	<20.1	≤34.1	≤102.5	≤205.1	>205.2
平均动脉压,mmHg	≥70	<70			
多巴胺，ug/(kg.min)			≤5	>5	>15
多巴喷丁胺，ug/(kg.min)			任何剂量		
肾上腺素，ug/(kg.min)				≤0.1	>0.1
去甲肾上腺素，ug/(kg.min)				≤0.1	>0.1
GCS 评分	15	13-14	10-12	6-9	<6
肌酐,umol/L	<106	≤176	≤308	≤442	>442
尿量，ml/d				≤500	≤200

附表 3 mNUTRIC 评分

	0 分	1 分	2 分	3 分
年龄	<50	50-<75	≥75	
APACHEII评分	<15	15-<20	20-28	≥28
SOFA 评分	<6	6-<10	≥10	
合并器官功能障碍数量	0-1	≥2		
入住 ICU 前住院天数	0-<1	≥1		

备注：改良 NUTRIC 评分总分为 5-9 分时，提示存在高营养风险；总分 0-4 分时，提示低营养风险。

(C)1994-2023 China Academic Journal Electronic Publishing House. All rights reserved. http://www.cnki.net

致谢

转眼间 2022 年的夏天悄然而至，2019 年的金秋仿佛还在眼前。虽叹时光易流逝，三年的时间就这样从指缝间溜走，也留下了一生最宝贵的财富。

有师如斯，庆幸至极。首先感谢我的导师—马晓薇教授，她是不仅是一位救死扶伤的医者，也是一位辛勤的学者，更是一位循循善诱的老师。在临床上，从诊疗思维到临床操作，她都悉心教授。科研上，从选题到文章撰写，她都耐心指导。在生活上，从工作到学习，她都无微不至的关心。学生深知遇良师不易，无论是她学术上的严谨态度还是为人处世都将是我一生学习的榜样。祝愿我的导师在科研、工作的道路上勇攀高峰，事事顺利，身体健康。

学贵得师，亦师亦友。其次感谢营养科常静老师给予课题的支持，感谢重症医学科的罗彩琴老师、沈佳老师、姚再先老师、王东磊老师以及王肖华护士长在重症医学科轮转期间给予的支持和鼓励，同时感谢在其他科室轮转期间帮助过我的老师。祝各位老师事业生活更上一层楼。

焉得萱草，言树之背。感谢父母的养育之恩，给了我温馨和睦的家庭，让我无后顾之忧追求自己的理想。唯有不断努力，让你们引以为傲。感谢爷爷奶奶，外公外婆给予的鼓励，每每遇到困难时，您们的鼓励总是支撑着我向前、向上。祝愿我的家人身体健康，平安顺遂。

山河不足重，重在遇知己。感谢我的舍友马丽莉，同门薛小兰、吕玉珍，师妹胥海欢。工作学习之余，幸得你们陪伴，让我在千里之外他乡感受到了家的温暖，更感谢你们对我的包容和关怀。愿我们保持热爱，奔赴未来，高处相见。

此刻，是终点，是我与校园告别的终点；更是起点，也是我由医学生变成医生的起点。

(C)1994-2023 China Academic Journal Electronic Publishing House. All rights reserved. http://www.cnki.net

攻读学位期间发表的学术论文目录

无。

(C)1994-2023 China Academic Journal Electronic Publishing House. All rights reserved. http://www.cnki.net

个人简介

一般情况：

姓　名：	杨宇璐
性　别：	女
年　龄：	26 岁
民　族：	汉族
籍　贯：	云南昆明

学习、进修与工作经历：

2014 年~2019 年	西北民族大学	临床医学专业	攻读学士学位
2019 年~2022 年	宁夏医科大学	急诊医学专业	攻读硕士学位

(C)1994-2023 China Academic Journal Electronic Publishing House. All rights reserved. http://www.cnki.net

开题、中期及学位论文答辩委员组成

1、开题报告专家小组成员

序号	姓名	职称/导师类型	工作单位	担任职务
1	杨立山	教授/硕导	宁夏医科大学总医院	组长
2	马希刚	教授/博导	宁夏医科大学总医院	委员
3	王晓红	教授/博导	宁夏医科大学总医院	委员
4	马磊	主任医师/硕导	宁夏医科大学总医院	委员

2、中期考核组成员

序号	姓名	职称/导师类型	工作单位	担任职务
1	杨立山	教授/硕导	宁夏医科大学总医院	组长
2	杨晓军	教授/硕导	宁夏医科大学总医院	委员
3	马希刚	教授/博导	宁夏医科大学总医院	委员
4	王晓红	教授/博导	宁夏医科大学总医院	委员
5	马磊	主任医师/硕导	宁夏医科大学总医院	委员

3、学位论文答辩委员会成员

序号	姓名	职称/导师类型	工作单位	担任职务
1	杨立山	教授/硕导	宁夏医科大学总医院	主席
2	杨晓军	教授/硕导	宁夏医科大学总医院	委员
3	马希刚	教授/博导	宁夏医科大学总医院	委员
4	王晓红	教授/博导	宁夏医科大学总医院	委员
5	卢燕	教授/硕导	银川市第一人民医院	委员

(C)1994-2023 China Academic Journal Electronic Publishing House. All rights reserved. http://www.cnki.net
