# Supplementary material for: Development and external validation of machine learning models for the early prediction of malnutrition in critically ill patients: a prospective observational study
Source: BMC Med Inform Decis Mak. 2025 Jul 3;25:248. doi: 10.1186/s12911-025-03082-9 (PMC12225150; doi:10.1186/s12911-025-03082-9)
Supplement: Supplementary file 15 — Supplementary Material 15 [file 12911_2025_3082_MOESM15_ESM.pdf]

DOI：10. 16506/j. 1009-6639. 2019. 08. 013

·论　著·

ICU 患者肠内营养喂养不足危险因素分析及预防措施

姚洋，江航帅，许宣宣，周佳烨
浙江医院 重症监护室，杭州 310053

摘要：目的　分析 ICU 患者肠内营养喂养不足危险因素分析及预防措施。**方法**　回顾性分析 2016 年 11 月至 2018 年 11 月在浙江医院 ICU 接受治疗且需肠内营养患者 152 例，其中 125 例喂养计划完成，作为完成组，剩余 27 例喂养计划未完成，作为未完成组；采集患者临床资料，包含 APACHE Ⅱ 评分、年龄及性别等，记录患者使用镇痛药物量（舒芬太尼）、行机械通气、使用镇静药物量（丙泊酚、咪达唑仑针）、使用去甲肾上腺激素量、意识水平情况（镇静者行 RASS 评分，其他行 GCS 评分）。同时观察未完成肠内营养者的因素，包含新发血流动力学不稳定、鼻饲管障碍、消化道出血、呕吐、需停止喂养的医疗检查或者操作、腹痛、腹泻、胃潴留及腹胀等；对影响患者肠内喂养不足因素行单因素和多因素回归分析。**结果**　未完成肠内喂养患者因素主要及占比为腹胀 33.33％、胃潴留 18.52％、医疗检查或者操作 14.81％、呕吐 11.11％；未完成组患者舒芬太尼剂量、丙泊酚剂量、去甲肾上腺素剂量分别为（0.05±0.02）g/（kg·min）、（185.50±25.17）mg/d 和（0.13±0.02）g/（kg·min），均高于完成组，差异有统计学意义（$P<0.05$）；两组患者机械通气比例、意识水平及咪达唑仑剂量差异无统计学意义（$P>0.05$）；使用高剂量的舒芬太尼、丙泊酚和去甲肾上腺素为造成 ICU 患者肠内喂养不足独立危险因素（$P<0.05$）。**结论**　ICU 患者肠内营养喂养不足危险因素为使用高剂量舒芬太尼、丙泊酚和去甲肾上腺素，针对上述患者应密切观察其胃肠道耐受情况，避免出现误吸。

关键词：肠内营养；喂养不足；重症监护室；危险因素
中图分类号：R605.97　**文献标识码：**A　**文章编号：**1009-6639（2019）08-0699-04

Analysis of risk factors for enteral nutrition feeding in patients with ICU and preventive measures

YAO Yang，JIANG Hang-shuai，XU Xuan-xuan，ZHOU Jia-ye
Zhejiang Hospital ICU Hangzhou 310053
Corresponding author：*JIANG Hang-shuai*，*E-mail*：*joy1981@163. com*

Abstract：**Objective**　To analyze the risk factors and preventive measures of enteral nutrition feeding in patients with ICU.　**Methods**　A retrospective analysis of 152 patients who received treatment for IUD in Zhejiang hospital from November 2016 to November 2018，of which 125 patients completed the feeding plan. As the completion group，the remaining 27 feeding plans were not completed as an unfinished group. Collect clinical data of patients，including APACHE II score，age and gender，record the amount of analgesic drugs（sufentanil），the proportion of mechanical ventilation，the amount of sedative drugs（propofol，midazolam），The amount of norepinephrine and the level of consciousness were used（respiratory RASS score，other GCS scores）. At the same time observe the factors of unfinished enteral nutrition，including new hemodynamic instability，nasal feeding tube disorders，gastrointestinal bleeding，vomiting，need to stop feeding medical examination or operation，abdominal pain，diarrhea，gastric retention and bloating；Univariate and multivariate regression analysis of factors affecting intestinal feeding in patients.　**Results**　The main factors of incomplete enteral feeding were abdominal distension 33.33％，gastric retention 18.52％，medical examination or operation 14.81％，vomiting 11.11％. The dosage of sufentanil，propofol and norepinephrine in the incomplete group were（0.05±0.02）g/kg·min，（185.50±25.17）mg/d and（0.13±0.02）g/（kg·min），respectively，

基金项目：浙江省医药卫生科技计划基金资助项目（编号 2017KY173）
作者简介：姚洋，护师，主要从事 重症监护工作
通信作者：江航帅，E-mail：joy1981@163. com

(C)1994-2023 China Academic Journal Electronic Publishing House. All rights reserved.　http://www.cnki.net

higher than those in the complete group，and the difference was statistically significant（$P<0.05$）；there was no significant difference in mechanical ventilation ratio，consciousness level and midazolam dose between the two groups（$P>0.05$）；high doses of sufentanil，propofol and norepinephrine were used. Insufficient risk factors for intestinal feeding in patients with ICU（$P<0.05$）． **Conclusions**　The risk factors for inadequate enteral nutrition feeding in ICU patients are high-dose sufentanil，propofol and norepinephrine. The above patients should be closely observed for gastrointestinal tolerance and avoid aspiration.

Key words：Enteral nutrition；Inadequate feeding；Intensive care unit；Risk factor

肠内喂养不足为医院 ICU 内广泛存在问题之一，会造成患者处于高分解状况与营养支持不足。临床造成患者肠内喂养不足不仅是低估了患者的需求量，还有一个因素为医嘱计划喂养量没有完成[1-2]。相关研究显示，医院 ICU 内患者肠内喂养量通常只能够达计划量的 51%～99%，且不同单位间其差异比较大，导致患者肠内喂养中断主要因素为气道护理、反流、喂养装置障碍、呕吐、医疗操作障碍及腹泻等[3]。当前对 ICU 肠内喂养不足研究主要为在整个 ICU 期间患者有无达到喂养的目标，但不是每天都进行评估，如使用镇静药与血管活性药物等对患者肠内喂养可能产生影响的因素则每天都进行调整[4-5]。因此，本研究经过分析 ICU 患者肠内营养喂养不足危险因素分析及预防措施，为临床患者诊疗提供一些借鉴。

1　资料与方法

1.1　病例资料　回顾性分析 2016 年 11 月至 2018 年 11 月在浙江医院 ICU 接受治疗且需肠内营养患者 152 例，排除存在血流动力学不稳定需连续液体复苏、显著酸中毒（pH＜7.25）及消化道穿孔等肠内喂养禁忌症，或者由于其他因素停止肠内营养者。其中 125 例喂养计划完成，作为完成组，剩余 27 例喂养计划未完成，作为未完成组。完成组患者年龄在 52～78 岁间，平均（73.14±6.39）岁，男性患者 73 例，女性患者 52 例，APACHE Ⅱ 平均评分（21.16±6.28）分；未完成组年龄在 50～79 岁间，平均（72.20±6.57）岁，男性患者 17 例，女性患者 10 例，APACHE Ⅱ 平均评分（21.57±6.10）分，两组患者临床资料差异无统计学意义（$P>0.05$），有可比性。患者或家属知情并签署同意书。

2.2　研究方法　（1）患者喂养方式：ICU 肠内喂养经过鼻饲管均匀、连续地泵入肠内营养素。患者完成喂养界定，实际完成喂养数量/计算喂养数量×100%≥65%判定为喂养完成，低于 65%则判定为喂养不足。（2）观察指标：采集患者临床资料，包含 APACHE Ⅱ 评分、年龄及性别等，记录患者使用镇痛药物量（舒芬太尼）、行机械通气、使用镇静药物量（丙泊酚、咪达唑仑）、使用去甲肾上腺激素量、意识水平情况（镇静者行 RASS 评分，其他行 GCS 评分）。同时观察未完成肠内营养者的因素，包含新发血流动力学不稳定、鼻饲管障碍、消化道出血、呕吐、需停止喂养进行医疗检查或者操作、腹痛、腹泻、胃潴留及腹胀等。

1.3　统计学处理　采用 SPSS 19.0 分析，计量资料（$\bar{x}\pm s$）表示，t 检验，计数资料 χ^2 检验，有两组间有差异的指标行 logistic 回归分析。患者意识水平当做单因素分析，但包含了 GCS 评分与 RASS 评分，所以对比意识水平时将 GCS 评分与 RASS 评分依据第 90 百分位加权后分析，$P<0.05$ 差异有统计学意义。

2　结　果

2.1　未完成组患者中断肠内喂养因素　未完成肠内喂养患者因素主要为腹胀、胃潴留、医疗检查或者操作、呕吐等，见表 1。

表 1　未完成组患者中断肠内喂养因素（例，%）

中断肠内喂养因素	例数（$n=27$）	发生率（%）
医疗检查或者操作	4	14.82
胃潴留	5	18.52
腹胀	9	33.33
腹痛	1	3.70
呕吐	3	11.11
新出现循环动力学不稳定	1	3.70
鼻饲管障碍	2	7.41
消化道出血	2	7.41

2.2　单因素分析 ICU 患者肠内喂养不足情况　未完成组患者舒芬太尼剂量、丙泊酚剂量、去甲肾上腺素剂量高于完成组，差异有统计学意义（$P<0.05$）；两组患者机械通气比例、意识水平及咪达唑仑剂量，差异无统计学意义（$P>0.05$），见表

(C)1994-2023 China Academic Journal Electronic Publishing House. All rights reserved.　http://www.cnki.net

2。

2.3 肠内喂养不足患者多因素回归分析 将组间对比有差异的单因素分析纳入行 logistic 回归分析，结果显示使用高剂量的舒芬太尼、丙泊酚和去甲肾上腺素为造成 ICU 患者肠内喂养不足独立危险因素（$P<0.05$），见表3。

表2 ICU 患者肠内喂养不足单因素分析

相关因素	完成组（n=125）	未完成组（n=27）	t/χ^2 值	P 值
舒芬太尼剂［g/（kg·min）］	0.01±0.01	0.05±0.02	12.064	<0.05
丙泊酚剂量（mg/d）	158.37±24.61	185.50±25.17	25.743	<0.05
咪达唑仑剂量（mg/d）	80.24±8.67	83.41±8.70	0.672	>0.05
去甲肾上腺素剂量（g/（kg·min）	0.04±0.02	0.13±0.02	9.762	<0.05
意识水平				
GCS 评分	13.04±3.87	12.25±3.91	0.089	>0.05
RASS 评分	4.09±1.64	4.81±1.53	0.315	>0.05
加权以后	0.92±0.34	1.08±0.32	0.409	>0.05
机械通气				
否	22（17.60）	6（22.22）	0.267	>0.05
是	103（82.40）	21（77.78）		

表3 肠内喂养不足患者 logistic 回归分析

相关因素	Wald	标准误	回归系数	P 值	OR 值	95%CI
舒芬太尼	4.291	1.130	2.158	0.008	8.691	1.833～16.354
丙泊酚	4.247	0.739	1.129	0.029	2.357	1.101～4.882
去甲肾上腺素	4.730	1.291	3.099	0.014	4.269	2.397～23.108

3 讨论

本研究显示即便 ICU 内护理工作者人员充足，但依然有部分患者的肠内喂养计划没有完成，这些患者使用舒芬太尼、丙泊酚与去甲肾上腺素数量过多有关。相关研究显示，在不同医院 ICU 内，其肠内喂养完成情况差异比较大，且上述研究中患者肠内完成喂养标准也都是实际喂养/计划喂养×100%≥65%，中断肠内喂养因素包含起气道护理、腹胀、医疗检查或者操作及胃潴留等，本研究使用的标准相同，结果也和相关研究近似。

医院 ICU 危重者多用药物为去甲肾上腺素，Annika 等[6]和 Nicolaus 等[7]研究显示，使用血管升压药物会造成临床患者对肠内营养无法正常摄入，这主要是由于使用血管升压药物时患者自身病情比较严重，同时会使患者胃肠道缺血情况加重，对胃肠道功能产生影响。但 Peter 等研究显示[8]，患者使用去甲肾上腺素行肠内喂养也比较安全，但患者肠内喂养耐受程度则会受去甲肾上腺素数量影响。本文研究显示，未完成组患者去甲肾上腺素剂量高于完成组，且高剂量去甲肾上腺素是造成 ICU 患者喂养不足危险因素。另外，本研究中排除了需连续液体复苏、显著酸中毒及血流动力学不稳定者，纳入患者去甲肾腺素的最大剂量也只有 0.3g/（kg·min），但其对患者完成肠内喂养也有影响。有研究已经验证，肠内喂养会受到使用镇静药物的影响，镇静药物会造成患者体内胃肠的蠕动下降[9-11]。但患者需求镇静药物的剂量受自身疾病程度影响，若临床患者存在严重感染，则胃肠道处于低灌注，对胃肠道蠕动能力也有干扰，所以在本研究中患者肠内喂养不足无法判定是患者本身疾病还是镇静药物所导致的。医院 ICU 内多见短效阿片类镇静药物为丙泊酚，患者不良反应主要为呕吐、腹胀和恶心等[12-13]。尽管当前对于连续泵注短效的阿片类药物对患者胃肠道的耐受性无针对性探究，但本研究显示，未完成组患者丙泊酚剂量高于完成组，且高剂量丙泊酚为造成 ICU 患者喂养不足危险因素。针对患者肠内喂养不足的相关危险因素，临床可采取适当预防措施，首先为预防禁食的时间，有专家认为患者禁食时间太长也为造成患者

(C)1994-2023 China Academic Journal Electronic Publishing House. All rights reserved. http://www.cnki.net

肠内喂养不足因素，患者生命体征稳定后，在入住ICU 24～72 h内可行肠内营养。然后为提升患者喂养耐受性，喂养不耐受为胃延迟排空和胃动力紊乱间接的指标，ICU内有近半患者会出现延迟性胃排空，造成患者喂养不耐受，临床症状是返流、胃残余量增大、呕吐、腹泻和腹胀等，那么针对此种症状可依据美国肠外肠内营养学会所发布的推荐意见，采取预防不耐受的一些措施，包含在喂养时量床头适当抬高30°～45°；患者间隔4h检测一次体内胃残留量；肠内喂养时采用专门营养泵，不能替代使用静脉输液泵；肠内营养物采用加热器加热；肠内喂养封闭管道装置每48h换1次[14-16]。同时要加强临床护理人员学习并掌握肠内营养管理和安全性评估，对患者胃腔残留量要密切观察，避免出现误吸情况。对患者需4 h检测一次残留量，若残留量≥200 ml，则使输注速度下降或者停止；若潴留量≤100 ml，则使输注速度提高至20 ml/h；若残留量≤200 ml，则保持原速度输注。

综上所述，ICU患者肠内营养喂养不足危险因素为使用高剂量舒芬太尼、丙泊酚和去甲肾上腺素，针对上述患者应密切观察其胃肠道耐受情况，避免出现误吸。

参考文献

[1] 张伟，朱宁宁，雷婷婷，等．胃癌患者术后肠内营养喂养不足循证预防护理方案构建[J]. 护理学杂志，2018，33（11）：5-8.

[2] Md R A，Mat Nor M B. Refeeding hypophosphataemia after enteral nutrition in a Malaysian intensive care unit：risk factors and outcome [J]. Asia Pac J Clin Nutr，2018，27（2）：329-332.

[3] Pw V D B，Rasmussen-Conrad ELNaber A H，Wanten G J. What you think is not what they get：significant discrepancies between prescribed and administered doses of tube feeding [J]. Br J Nutr，2008，101（1）：68-71.

[4] 曹金凤，倪娟娟，刘娟，等．危重患者肠内营养期间发生喂养不耐受危险因素及相关护理干预[J]. 山西医药杂志，2017，46（18）：2251-2253.

[5] 周田田，王保荣，朱小芳，等．ICU脓毒症患者肠内营养喂养不耐受的相关因素分析[J]. 护理学报，2017，24（16）：6-9.

[6] Annika R B，Joel S，Waleed A，*et al*. Early enteral nutrition in critically ill patients：ESICM clinical practice guidelines [J]. Intensive Care Med. 2017；43（3）：380-398.

[7] Nicolaus N，Kristina S，Lori B，*et al*. Implementing Dietary Modifications and Assessing Nutritional Adequacy of Diets for Inflammatory Bowel Disease. [J]. Gastr Hep（N Y），2019；15（3）：133-144.

[8] Peter J W，Luc C，Mark D，*et al*. Proteins and amino acids are fundamental to optimal nutrition support in critically ill patients [J]. Crit Care. 2014；18（6）：591.

[9] 刘芳，高岚，王宇娇，等．重症脑损伤患者肠内营养支持的护理实践与依据[J]. 中国护理管理，2017，17（9）：1166-1171.

[10] Lee Z Y，Ibrahim N A，Mohdyusof B N. Prevalence and duration of reasons for enteral nutrition feeding interruption in a tertiary intensive care unit. [J]. Nutrition，2018，53（25）：666-669.

[11] 郑祥德，周文来，兰清．空肠营养管床旁徒手安置技术在基层医院重症监护病房的应用[J]. 中华临床营养杂志，2016，24（1）：45-46.

[12] Orinovsky I，Raizman E. Improvement of Nutritional Intake in Intensive Care Unit Patients via a Nurse-Led Enteral Nutrition Feeding Protocol. [J]. Cr Care Nu，2018，38（3）：38-41.

[13] 方伯梁，钱素云．PICU脓毒症及严重脓毒症患儿肠内营养摄入能量及蛋白质充分性及其与预后的关系[J]. 首都医科大学学报，2016，37（2）：125-129.

[14] Lv B，Hu LH，Chen LF，*et al*. Blind bedside postpyloric placement of spiral tube as rescue therapy in critically ill patients：a prospective，tricentric，observational study [J]. Crit Care. 2017；21：248.

[15] 宿英英，曾小雁，姜梦迪，等．重症神经疾病病人肠内营养能量预测目标值与实际供给值比较[J]. 肠外与肠内营养，2016，23（4）：261-264.

[16] Padar M，Uusvel G，Starkopf L，*et al*. Implementation of enteral feeding protocol in an intensive care unit：Before-and-after study [J]. World J Cr Care Med，2017，6（1）：56-64.

收稿日期：2019-01-05 修回日期：2019-05-25

(C)1994-2023 China Academic Journal Electronic Publishing House. All rights reserved. http://www.cnki.net
