# Supplementary material for: Development and external validation of machine learning models for the early prediction of malnutrition in critically ill patients: a prospective observational study
Source: BMC Med Inform Decis Mak. 2025 Jul 3;25:248. doi: 10.1186/s12911-025-03082-9 (PMC12225150; doi:10.1186/s12911-025-03082-9)
Supplement: Supplementary file 16 — Supplementary Material 16 [file 12911_2025_3082_MOESM16_ESM.pdf]

[30] 付向阳,郭春兰.纳米银抗菌敷料在糖尿病并发浅表脓肿切开引流伤口换药中的应用[J].中华医院感染学杂志,2011,21(9):1818-1819.

[31] Muangman P,Pundee C,Opasanon S,*et al*. A prospective,randomized trial of silver containing hydrofiber dressing versus 1% silver sulfadiazine for the treatment of partial thickness burns [J]. Int Wound J,2010,7(4):271-276.

[32] Asz J,Asz D,Moushey R,*et al*. Treatment of toxic epidermal necrolysis in a pediatric patient with a nano crystalline silver dressing[J]. J Pediatr Surg,2006,41(12):9-12.

[33] 葛小静,章宏伟,史京萍,等.藻酸盐银联合水凝胶敷料对慢性创面愈合的作用[J].中国组织工程研究,2012,16(3):539-542.

[34] Tien DC,Tseng KH,Liao CY,*et al*. Colloidal silver fabrication using the spark discharge system and its antimicrobial effect on Staphylococcus aureus[J]. Medical Engineering Physics,2008,30(8):948-952.

[35] Cencetti C,Bellini D,Pavesio A,*et al*. Preparation and characterization of antimicrobial wound dressings based on silver,gellan,PVA and borax[J]. Carbohydr Polym,2012,90(3):1362-1370.

[36] Vigneshwaran N,Nachance RP,Balasubramany RH,*et al*. A novel one-pot'green' synthesis of stable silver nanoparticles using soluble starch[J]. Carbohydtate Research,2006,341(12):2012-2018.

[37] Agren MS,Mirastschijski U,Karlsmark T,*et al*. Topical synthetic inhibitor of matrix metalloproteinases delays epidermal regeneration in human wounds[J]. Exp Dermatol,2001(10):337-348.

[38] Agren MS. Matrix metalloproteinases(MMPs) are required for reepithelialization of cutaneous wounds[J]. Arch Dermatol Res,1999,291:583-590.

(收稿日期:2014-07-22;修回日期:2015-03-11)
(本文编辑 孙玉梅)

重症卒中病人早期肠内营养喂养不达标状况及其影响因素分析

张　力,张晓梅,梁玉婷,彭　娟,周宏珍

摘要:[目的]调查重症卒中病人早期肠内营养达标率的影响因素。[方法]选取 2013 年 1 月—2014 年 6 月的重症脑卒中病人 182 例,分为达标组与不达标组,通过 Logistic 分析,探讨早期肠内营养不达标的主要影响因素。[结果]182 例病人中有 123 例(67.6%)早期喂养不达标,两组病人格拉斯哥昏迷评分(GCS)、腹泻、呕吐或反流、胃残余量>100 mL、机械通气的差异有统计学意义($P<0.05$)。Logistic 回归分析结果显示,病人出现腹泻、呕吐或反流、胃残余量>100 mL、行机械通气对早期肠内营养不达标有影响。[结论]腹泻、呕吐或反流、胃残余量>100 mL、机械通气可能为卒中病人早期肠内营养不达标的影响因素。

关键词:重症卒中;早期肠内营养;不达标状况;影响因素

Analysis of non-compliance condition of early enteral nutrition feeding of severe stroke patients and its influencing factors

Zhang Li,Zhang Xiaomei,Liang Yuting,*et al*(Nanfang Hospital of Southern Medical University,Guangdong 510515 China)

Abstract **Objective**:To investigate the influencing factors of compliance rate of early enteral nutrition feeding of severe stroke patients. **Methods**:A total of 182 cases of severe stroke patients were selected from 2013 January to 2014 June,and they were divided into compliance group and non non-compliance group,through the Logistic analysis,to probe into the main influencing factors of non-compliance of early enteral nutrition. **Results**:There were 123 cases with non-compliance of early feeding in 182 patients(67.6%). There were statistically significant difference in GCS score,diarrhea,vomiting or regurgitation,gastric residual volume >100 mL and mechanical ventilation between both groups($P<0.05$). Logistic regression analysis results showed that patients with diarrhea,vomiting,stomach or regurgitation,gastric residual volume >100 mL and mechanical ventilation had influence on non-compliance condition of early enteral nutrition feeding. **Conclusion**:Diarrhea,vomiting or regurgitation,gastric residual volume >100 mL and mechanical ventilation may be the influencing factors of non-compliance of early enteral nutrition of severe stroke patients.

Key words severe stroke;early enteral nutrition;non-compliance condition;influencing factors

中图分类号:R473.74 **文献标识码:**A **doi:**10.3969/j.issn.1009-6493.2015.10.007 **文章编号:**1009-6493(2015)04A-1175-04

基金项目:广东省科技计划项目,编号:2011B031800283。

作者简介:张力,硕士研究生在读,单位:510515,南方医科大学南方医院;张晓梅、梁玉婷、彭娟、周宏珍(通讯作者)单位:510515,南方医科大学南方医院。

(C)1994-2023 China Academic Journal Electronic Publishing House. All rights reserved. http://www.cnki.net

目前，对重症脑卒中病人行营养支持的首选途径为肠内营养[1]。神经内科重症监护室行肠内营养的方式普遍为肠内营养泵持续喂养。研究表明，在没有肠内营养禁忌证的情况下，对重症卒中病人尽早地进行肠内营养比肠外营养更加适合病人的生理状况，有利于提高病人的自身免疫力，维持一个正常的胃肠道功能[2]。但肠内营养期间会出现一系列相关的问题，如腹胀、腹泻、高血糖、呕吐、误吸等，影响病人肠内营养的进行[3]。而且对于重症脑卒中病人来说，一些临床操作，如腰椎穿刺，也经常使得肠内营养不得不暂停[4]。研究表明，重症病人早期肠内营养的喂养不达标情况，会延长病人的 ICU 停留时间，增加病人的医疗费用[5]。因此，为了初步了解重症卒中病人早期肠内营养不达标的影响因素，为进一步改善重症卒中病人的肠内营养提供依据，本研究对某三级甲等医院神经内科重症监护室的重症脑卒中病人的早期肠内营养情况进行了观察和调查。现报告如下。

1　对象与方法

1.1　研究对象　采取横断面研究，调查 2013 年 1 月—2014 年 6 月某三级甲等医院神经内科重症脑卒中病人 182 例。纳入标准：年龄≥18 岁；入住神经内科重症监护室后 48 h 内行肠内营养，并预计肠内营养时间＞3 d 的病人。排除标准：①有腹部、食管、十二指肠、胰腺、胃手术史；②从食管、胃或肠道给药有禁忌证者；③造瘘术肠内营养者；④孕妇；⑤治疗限制者；有肺炎或疑似肺炎者；⑥胃部以下方式置管者；⑦入 ICU 48 h 内未进行肠内营养者。

1.2　方法

1.2.1　营养支持方法　本研究采用的营养素为雅培公司提供的瑞代[肠内营养乳剂（TPF-D）]、能全力[肠内营养混悬液（TPF）]以及佳维体[肠内营养混悬液（TPF-FOS）]3 种。对于入住神经重症监护室的所有病人均留置鼻胃管。病人实行早期肠内营养，时间在 48 h 以内实行，并根据喂养量调节速度以保证肠内营养喂养的连续性。采用肠内营养泵连续输注，控制温度在 39 ℃～41 ℃。

1.2.2　调查方法　研究者在查阅大量文献综述的基础上采用自行设计的调查表进行调查。调查表由两部分构成：①病人的一般资料及一般检查情况，包括性别、年龄、格拉斯哥昏迷评分（GCS）等；②肠内营养支持相关资料，包括机械通气、腹泻、高血糖、呕吐或反流、误吸、胃残余量＞100 mL 等。根据《欧洲肠外肠内营养学会（ESPEN）肠内营养指南》，病人每日所需的肠内营养量为 25 kcal/（kg · d）（1 kcal＝4.18 kJ）[6]。当病人处于应激状态时，可以给予病人目标喂养量的 60%[7]。因此，本研究以肠内营养的第 3 天目标喂养量的 60%为分界线，将进行早期肠内营养的病人分为两类：≥ 60%目标喂养量为达标组，＜60%为喂养不达标组。

1.2.3　资料分析方法　数据采用 SPSS 13.0 软件进行统计分析，计量资料采用 t 检验，计数资料采用 χ^2 检验，影响因素采用二分类 Logistic 回归分析。

2　结果

2.1　病人一般资料　符合纳入标准的病人共 182 例，其中目标喂养量达标者 59 例（32.4%），不达标者 123 例（67.6%），两组一般资料比较见表 1。

表 1　两组一般资料比较（n=182）

组别	例数	性别（例）		年龄（$\bar{x}\pm s$）	GCS 评分	腹泻	呕吐或反流	高血糖	胃残余量＞100 mL	机械通气
		男	女	岁	分	例	例	例	例	例
未达标组	123	73	50	61.36±15.08	7.12±3.40	49	22	26	20	33
达标组	59	39	20	61.17±15.49	10.08±3.68	13	2	14	3	7
统计值		χ^2=0.764		t=0.907	t=−2.180	χ^2=5.627	χ^2=7.319	χ^2=0.156	χ^2=4.511	χ^2=8.132
P		0.382		0.336	0.022	0.018	0.007	0.693	0.034	0.004

2.2　Logistic 回归分析　以肠内营养达标状况为应变量，以病人的一般资料、入神经内科重症监护室后肠内营养相关资料为自变量进行 Logistic 回归分析。最终有 4 个变量进入了 Logistic 回归分析模型，结果见表 2。

表 2　早期肠内营养不达标多因素的 Logistic 回归分析

项目	回归系数	P	OR 值	OR 值 95%CI
腹泻	0.792	0.039	2.208	1.042～4.682
呕吐或反流	2.093	0.006	8.112	1.797～36.610
胃残余量＞100 mL	1.408	0.033	4.087	1.124～14.868
机械通气	1.183	0.011	3.265	1.309～8.143

3　讨论

3.1　重症卒中病人早期肠内营养喂养达标状况　研究表明，对重症病人尽早实施肠内营养不仅能为病人提供足够的能量和营养物质，而且能维持肠道的正常生理功能，改善组织灌注与营养吸收，并且能明显降低感染并发症与死亡率[2]。因此，肠内营养是其首要的营养供给方式。对于重症卒中病人来说，尽早使用肠内营养已经得到了广泛的应用与重视，但在实际应用方面，肠内营养的使用量经常处于较低的水平。本研

(C)1994-2023 China Academic Journal Electronic Publishing House. All rights reserved.　http://www.cnki.net

究中 182 例目标病人中,早期肠内营养达标者只有 59 例(32.4%),远远低于未达标例数 123 例(67.6%)。

值得关注的是,在肠内营养的不良问题方面,都有存在早期肠内营养不达标的现象发生,详见表 1。这与以往的只是以早期肠内营养不耐受这一因素来判定早期肠内营养好坏有所不同[8]。以往研究表明,重症病人早期肠内营养的喂养不达标,会延长病人的 ICU 停留时间(达标组为7.50 d±3.54 d,不达标组为 13.11 d±9.8 d,$P<0.05$),增加病人的医疗费用(达标组为 3.64 万元±2.83 万元,不达标组 6.57 万元±4.65 万元,$P<0.05$)[6]。提示,临床医护人员对重症卒中病人在施行早期肠内营养时,仅仅只是考虑耐受与否是不够的,还应虑病人早期肠内营养能否达标。当然这也有待于进一步的临床研究。

3.2 重症卒中病人早期肠内营养达标的影响因素及改善措施 本研究的两组病人在年龄、性别、是否有高血糖方面差异无统计学意义($P>0.05$),详见表 1。两组 GCS 评分显示差异有统计学意义($P=0.022$)。但 Logistic 回归分析显示 GCS 评分和高血糖并不是影响早期肠内营养达标的因素(未纳入)。

本研究还发现,两组病人在早期肠内营养期间是否发生腹泻的差异有统计学意义($P=0.018$),Logistic 回归分析显示腹泻为影响早期肠内营养不达标的因素($OR=2.208$,95%CI为1.042~4.682,$P=0.039$)。这可能是由于病人发生腹泻,医护人员暂停肠内营养而导致。当病人发生腹泻时,临床护士可能会担心这些症状会引起其他护理问题出现,如增加臀部压疮的风险,往往对病人的症状予以过度严重的描述,使得肠内营养经常处于暂停状态[9],这就很大程度上导致了喂养不达标。导管污染也可能导致病人发生腹泻[10]。所以,当病人有腹泻出现时,临床护士应该尽早报告医生,协同医生找出病人腹泻的原因,以更好地保证肠内营养的进行。

本研究显示,两组病人是否发生呕吐或反流比较,差异有统计学意义($P=0.007$),Logistic 回归分析显示,呕吐或反流为影响病人早期肠内营养不达标的因素($OR=8.112$,95%CI为1.797~36.610,$P=0.006$)。这可能与重症卒中病人 24 h 都处于卧床状态而导致肠胃动力不足,再加上使用肠内营养泵持续喂养,使病人的胃残余量增大有关。病人发生呕吐不仅增加护士的工作量,而且浪费了营养物质,继而影响肠内营养的达标。对于重症卒中病人,一定要加强对胃残余的监测,同时对于胃动力不足的病人,报告医生,合理运用胃动力药物,及时调整肠内营养泵的速度以适应病人。将病人的床头抬高 30°以上也可以降低呕吐或反流的发生[11]。

两组研究比较,胃残余量>100 mL 差异有统计学意义,Logistic 回归分析显示胃残余量>100 mL 为影响早期肠内营养达标的因素($OR=4.087$,95%CI 为 1.124~14.868,$P=0.033$)。监测病人的胃残余量对于肠内营养有重要的指导意义,减少病人发生肠内营养并发症,如腹胀、呕吐、反流、误吸的发生[12]。胃残余量一般都和胃潴留有关。以往研究显示,虽然对于以多少胃残余量定义胃潴留阈值没有统一标准,如根据学者王小松[13]研究表明,当病人胃残余量大于或等于 100 mL 时,则认为病人出现胃潴留。黄尹明[14]认为,当病人的胃残余量大于 200 mL 时则认为是病人出现胃潴留。国外学者通过综述文献的基础上,认为当胃残余量大于 500 mL 才认为是胃潴留[15]。但病人出现胃残余量增大,一般预示着病人的胃肠道功能比较差,无论是吸收功能还是运动功能都低于正常,这就可能影响肠内营养的正常进行,进一步影响肠内营养达标率。对于胃残余量比较大的病人,临床护士应及时告知医生,使用胃动力药物促进消化,或使用物理方法,如按摩腹部,促进消化。

两组病人比较,是否进行机械通气差异有统计学意义($P=0.004$),Logistic 回归分析显示机械通气为影响早期肠内营养不达标的因素($OR=3.265$,95%CI 为 1.309~8.143,$P=0.011$)。行机械通气的病人本身就是属于病情相对比较重,加之人工气道吸痰对气道的刺激病人咳嗽而增加腹压,从而增加呕吐或反流,影响病人对营养液的吸收[16],进而影响早期肠内营养达标。因此,对于行机械通气的病人,吸痰时严格无菌操作的同时,动作一定要轻,吸痰过程中要暂停营养泵。

4 小结

通过本研究发现,重症卒中病人早期肠内营养达标状况有待于进一步提高,影响肠内营养早期实施的因素是多元的、广泛的。护理人员作为肠内营养的重要实施者、管理者、参与者,在肠内营养治疗中起着重要的作用。建议在肠内营养实施过程中,尤其肠内营养实施的早期,依据病人的实际情况,合理应用肠内营养剂,采取个体化肠内营养支持。应该指出,本研究仅对影响重症卒中早期肠内营养达标状况进行初步调查及分析,有待扩大样本量及采取有效干预措施进行下一步研究,而且对于不同肠内营养剂之间是否有影响也有待于进一步的临床研究。

参考文献:

[1] Khalid I,Dashy P,Dig ovine B. Early enterable nutrition and out-

(C)1994-2023 China Academic Journal Electronic Publishing House. All rights reserved. http://www.cnki.net

comes of critically ill patients treated with vasopressors and mechanical ventilation[J]. Am J Crit Care,2010,19(3):261-268.
[2] Woo SH,Finch CK,Broyles JE,*et al*. Early vs delayed enteral nutrition in critically ill medical patients[J]. Nutr Clin Pract,2010,25(2):205-211.
[3] 陈梅芳.神经内科重症患者肠内营养时机与并发症的分析及护理[J].中国医药指南,2013(10):28.
[4] 方燕云.重症脑卒中患者早期肠内营养中断的原因及对策[J].中国中医急症,2012,21(5):849-850.
[5] 方理超,徐文秀,刘励军.早期肠内营养达标对不同程度重症患者预后的影响[J].中华急诊医学杂志,2010,19(11):1201-1204.
[6]《欧洲肠外肠内营养学会(ESPEN)肠内营养指南》介绍系列[J].中国全科医学,2007,10(16):1354.
[7] 高宝祥.不同应激程度危重症患者早期肠内营养达标率差异的研究[D].苏州:苏州大学,2013:1.
[8] 魏娜,王春梅.肠内营养不耐受的危险因素研究[J].护理研究,2008,22(9B):2358-2359.
[9] Valls Miró C,Carreño Granero A,Domingo Felici CM,*et al*. Measurement of residual gastric volume:In search of better evidence[J]. Enferm Intensiva,2006,17(4):154-162.
[10] 李秀川.肠内营养腹泻相关因素的调查分析及护理干预[J].临床护理杂志,2008,7(4):5-7.
[11] 程艳爽,王建荣,马燕兰.鼻饲体位与方式对创伤昏迷病人胃内容物反流及误吸的影响[J].护理研究,2006,20(7B):1992-1995.
[12] 鹿振辉.肠内营养过程中测定胃残余量的意义[J].中华现代护理杂志,2009,15(1):39-40.
[13] 王小松.监测胃残余量在COPD患者机械通气治疗中的临床应用[J].中国冶金工业医学杂志,2011,28(6):707-708.
[14] 黄伊明.胃残余量监测在内科机械通气病人肠内营养中的应用护理[J].护理实践与研究,2013,10(20):30-31.
[15] Pullen RJ. Measuring gastric residual volume[J]. Nursing,2004,34(4):18.
[16] 周飞燕,许勤,陈丽,等.胃癌术后患者早期肠内营养达标状况及其影响因素研究[J].护理学杂志,2012,27(6):76-78.

(收稿日期:2014-09-29;修回日期:2015-03-11)
(本文编辑 孙玉梅)

家庭-社区-医院系统护理干预对颅脑外伤综合征病人生活质量的影响

陈戈婷,麦顺和,兰　一

摘要:[目的]探讨家庭-社区-医院系统护理干预模式对颅脑外伤综合征病人生活质量的影响。[方法]将200例颅脑外伤综合征病人随机分为观察组和对照组各100例,对照组在住院期间遵医嘱执行常规治疗和护理,出院后除定期给予调查了解情况外不采取其他特殊治疗护理措施,观察组实施家庭-社区-医院系统护理干预模式,经过护理干预3个月~12个月,平均9.02个月,并进行追踪随访,比较两组颅脑外伤综合征病人的临床观察指标、临床疗效、病人满意度。[结果]两组病人在临床观察指标、临床疗效、病人满意度方面差异均有统计学意义($P<0.05$或$P<0.01$)。[结论]实施家庭-社区-医院系统护理干预能有效减轻病人颅脑综合征症状,提高病人的生活质量和病人的满意度。

关键词:颅脑外伤综合征;家庭-社区-医院系统护理干预;生活质量

Influence of family-community-hospital system nursing intervention on quality of life of patients with craniocerebral injury syndrome

Chen Geting,Mai Shunhe,Lan Yi(Chancheng Hospital of First People's Hospital of Foshan City Guangdong Province,Guangdong 528061 China)

Abstract **Objective**:To probe into the influence of family-community-hospital system nursing intervention on the quality of life of patients with craniocerebral injury syndrome. **Methods**:A total of 200 patients with craniocerebral injury syndrome were randomly divided into observation group and control group,100 cases in each group. The patients in control group received the routine therapy and nursing care following the doctor's advice when they were in hospital. When leaving the hospital,they didn't take other special treatment or nursing except some regular investigation;the patients in observation group received family-community-hospital system nursing intervention,after nursing intervention for 3-12 months,average 9.02 months to follow-up. The clinical observation index,clinical efficacy and patients' satisfaction were compared between both groups. **Results**:There was statistically significant difference in clinical observation index,clinical efficacy and patients' satisfaction between both groups($P<0.05$ or $P<0.01$). **Conclusion**:The implementation of family-community-hospital system nursing intervention can effectively relieve the symptoms in patients with craniocerebral injury syndrome and improve the quality of life and the satisfaction of patients.

Key words craniocerebral injury syndrome;family-community-hospital system nursing intervention;quality of life

中图分类号:R473.74　**文献标识码**:A　**doi**:10.3969/j.issn.1009-6493.2015.10.008　**文章编号**:1009-6493(2015)04A-1178-04

基金项目:广东省佛山市卫生局医学科研课题,编号:2014167。

作者简介:陈戈婷,本科,单位:528061,广东省佛山市第一人民医院禅城医院;麦顺和、兰一单位:528061,广东省佛山市第一人民医院禅城医院。

(C)1994-2023 China Academic Journal Electronic Publishing House. All rights reserved. http://www.cnki.net
