# Supplementary material for: Development and external validation of machine learning models for the early prediction of malnutrition in critically ill patients: a prospective observational study
Source: BMC Med Inform Decis Mak. 2025 Jul 3;25:248. doi: 10.1186/s12911-025-03082-9 (PMC12225150; doi:10.1186/s12911-025-03082-9)
Supplement: Supplementary file 17 — Supplementary Material 17 [file 12911_2025_3082_MOESM17_ESM.pdf]

· 论著 ·

重症患者肠内喂养不足的危险因素分析

郑蕾[1]　桑岭[2]　黎毅敏[2]　闫凤[1]　刘晓青[2]

1 广州医科大学附属第一医院营养科(广东广州　510120); 2 广州医科大学附属第一医院重症医学科(广东广州　510120)

【摘要】 目的　了解重症医学科(ICU)内重症患者肠内喂养不足的发生率、主要原因和导致喂养不足的危险因素。**方法**　采用前瞻性单中心研究,选择2013年10月至2013年12月广州医科大学附属第一医院重症医学科收治的需要肠内营养的患者。患者入组后记录性别、年龄以及入组时急性生理学和慢性健康状况评分系统Ⅱ(APACHEⅡ)评分。其后以每天为观察单位,记录患者当日的计划肠内喂养量和实际完成肠内喂养量,以实际完成肠内喂养量/计划肠内喂养量≥65%为界,将患者当日分为肠内喂养完成组(≥65%)和肠内喂养不足组(<65%)。分别记录患者是否需要机械通气、意识水平、去甲肾上腺素使用量、镇静药物使用量、镇痛药物使用量,并记录患者肠内营养中断的原因。**结果**　总共入组符合合标准行肠内喂养天数302 d,来源于31例患者。分为肠内喂养完成组(≥65%)249 d和肠内喂养不足组(<65%)53 d。肠内喂养不足组营养喂养中断的主要原因为腹胀(38.1%),胃潴留(26.4%),医疗操作或检查(17.3%),呕吐(11.4%),消化道出血(9.2%),鼻饲管障碍(5.8%),新出现循环动力学不稳定(4.3%),腹痛(1.4%)。两组间进行单因素比较发现喂养成功组去甲肾上腺素、丙泊酚、瑞芬太尼使用量小于喂养不足组,两组间比较有显著差异($P<0.05$),将以上指标进行Logistic多因素分析发现以上三者均为肠内喂养不足的独立危险因素($P<0.05$)。**结论**　ICU内重症患者仍存在肠内喂养不足的现象,去甲肾上腺素、丙泊酚、瑞芬太尼的使用是导致喂养不足的独立危险因素。

【关键词】 肠内营养;　去甲肾上腺素;　丙泊酚;　瑞芬太尼

Risk Factors Analysis for Inadequate Enteral Nutrition in Critically Ill Patients　*Zheng Lei*[*], *Sang Ling, Li Yimin, Yan Feng, Liu Xiaoqing.* [*] *Department of Nutriology, The First Affiliated Hospital of Guangzhou Medical University, Guangzhou, Guangdong, 510120, China*

Corresponding Author: Liu Xiaoqing, E-mail: lxq1118@126. com

【Abstract】 Objective　To determine the incidence rate, main causes and risk factors of inadequate enteral nutrition in critically ill patients in intensive care unit(ICU). **Methods**　A single-center prospective cohort study was conducted. Critically ill patients who need enteral nutrition in ICU of the First Affiliated Hospital of Guangzhou Medical University from October 2013 to December 2013 were enrolled. Each patient´s demographic and clinical characteristics(gender, age, APACHE Ⅱ score), the need for ventilatory support, the level of consciousness, the use and dosage of medications, and the causes for cessation of enteral nutrition were recorded. The daily evaluation of nutrition intake was executed. Enteral nutrition was considered adequate if the patient received at least 65% of the recommended calories (complete group ≥65% and insufficient group <65%). **Results**　A total of 302 daily evaluations were done in 31 patients. They were divided into an enteral nutrition complete group (249 cases) and an enteral nutrition insufficient group (53 cases). The main reasons for insufficient enteral nutrition were ventosity (38.1%), reflux (26.4%), medical procedure and examination (17.3%), vomiting (11.4%), gastrointestinal bleeding (9.2%), problems with the enteral nutrition tube(5.8%), hemodynamic instability(4.3%), and abdominal pain(1.4%). Univariate analysis revealed that the dosage of norepinephrine, propofol and remifentanil were significantly lower in the enteral nutrition complete group compared with the enteral nutrition insufficient group($P<0.05$). Logistic analysis revealed that the dosage of norepinephrine, propofol and remifentanil

DOI: 10.7507/1671-6205.2014096

通信作者: 刘晓青, E-mail: lxq1118@126. com

(C)1994-2024 China Academic Journal Electronic Publishing House. All rights reserved.　http://www.cnki.net

were independent risk factors for inadequate enteral nutrition ($P < 0.05$). **Conclusion** Achievement of daily enteral nutrition goals is inadequate in critically ill patients, and the main risk factors associated with this failure are the use and dosage of norepinephrine, propofol and remifentanil.

【Key words】 Enteral nutrition; Norepinephrine; Propofol; Remifentanil

肠内喂养不足在重症医学科(ICU) 内是普遍存在的问题[1-2]，它将导致营养支持不足和患者的高分解代谢状态[3-4]。而肠内喂养不足除了由于对患者需求量的低估外，另一个重要原因是未能完成医嘱计划内的喂养量。研究表明在 ICU 内肠内喂养量一般仅达到计划内的 51% ~99%，各单位间差别较大[5-8]，造成肠内喂养中断的原因主要有反流、呕吐、腹泻、医疗操作及检查、喂养装置障碍以及气道护理。目前的研究主要集中在患者在整个 ICU 停留时间内是否达到喂养目标而不是每日评估，而一些可能影响肠内喂养的因素如血管活性药物和镇静药物的使用是需要每日调整的。另外，护士工作量已经被认为会影响重症患者的预后和住院费用[9-10]，Honda 等[11]指出护患比 > 0.51 可以更好地完成肠内喂养计划。但对于护理人员充足的 ICU，还没有专门的研究证实患者肠内喂养不足的危险因素。因此，我们设计本研究，希望通过对 ICU 内行肠内喂养的重症患者的每日观察，发现肠内喂养每日的实际完成量以及造成肠内喂养不足的危险因素。将来可以更好地指导临床医护人员完成肠内喂养计划。

对象与方法

一、对象

采用前瞻性单中心观察性研究方法，选择 2013 年 10 月至 2013 年 12 月广州医科大学附属第一医院 ICU 收治的需要肠内营养的患者，所有患者入住 ICU > 48 h。排除标准: 存在肠内喂养的禁忌证如消化道穿孔，明显酸中毒(pH < 7.25)，血流动力学不稳定需要持续液体复苏，或其他原因由临床医生主动停止肠内营养的患者。

二、方法

1. 肠内喂养方式: ICU 肠内喂养方式为通过鼻饲管持续匀速泵入肠内营养素。本研究单位护患比例常规≥0.6。

2. 观察指标: 在患者入组后记录性别、年龄以及入组时急性生理学和慢性健康状况评分系统Ⅱ(APACHEⅡ) 评分。其后以每天为观察单位，记录患者当日的计划肠内喂养量和实际完成肠内喂养量，以实际完成肠内喂养量/计划肠内喂养量≥65% 为界，将患者当日分为肠内喂养完成组(≥65%) 和肠内喂养不足组(< 65%)。分别记录患者是否需要机械通气、意识水平(镇静患者使用 Ramsay 评分，其余患者使用 GCS 评分) 、去甲肾上腺素使用量、镇静药物使用量(咪唑安定、丙泊酚) 、镇痛药物使用量(瑞芬太尼)。并记录患者肠内营养中断的原因: 鼻饲管障碍、呕吐、腹痛、腹胀、胃潴留(每 4 h 回抽鼻饲管，回抽量≥4 h 鼻饲量的 50%) 、腹泻(水样便≥3 次/d) 、需要停止喂养的医疗操作或检查、消化道出血、新发的血流动力学不稳定以及其他原因。

三、统计学处理

利用 EXCEL 为患者建立数据库，使用 SPSS 17.0 统计软件包进行统计分析。计量资料用 $\bar{x} \pm s$ 或中位数(四分位间距) [M(Q)] 表示。两组间计量资料比较，正态分布时采用 t 检验，非正态分布时采用非参数检验，率的比较使用 χ^2 检验。所有指标先进行单因素分析，有显著性差异的指标进行 Logistic 多因素分析。由于意识水平作为一个单因素比较指标，但它包括 Ramsay 评分和 GCS 评分，因此在进行意识水平比较时先将 Ramsay 评分和 GCS 评分根据第 90 百分位数进行加权(weighted analysis) 后再进行分析。$P < 0.05$ 为差异有统计学意义。

结 果

一、一般情况

共入组符合标准行肠内喂养天数 302 d。来源于 31 例患者，男 19 例，女 12 例; 年龄(72.4 ±7.82) 岁; APACHEⅡ评分为(21.4 ±9.12) 分。

二、肠内喂养不足危险因素的单因素分析

将观察单位以实际完成肠内喂养量/计划肠内喂养量≥65% 为界，将患者当日分为肠内喂养完成组(≥65%) 249 d 和肠内喂养不足组(< 65%) 53 d。肠内喂养不足组营养喂养中断的主要原因为腹胀(38.1%) 、胃潴留(26.4%) 、医疗操作或检查(17.3%) 、呕吐(11.4%) 、消化道出血(9.2%) 、鼻饲管障碍(5.8%) 、新出现循环动力学不稳定(4.3%) 和腹痛(1.4%)。两组间单因素比较结果见表 1。

(C)1994-2024 China Academic Journal Electronic Publishing House. All rights reserved. http://www.cnki.net

表 1　肠内喂养不足危险因素单因素分析 [M(Q)]

变量	喂养完成组 (n = 249)	喂养不足组 (n = 53)	P 值
机械通气(例):			
是	205	41	0.44
否	44	12	
意识水平:			
Ramsay 评分	4(3 ~ 6)	5(5 ~ 6)	0.21
GCS 评分	13(10 ~ 14)	12(10 ~ 13)	0.41
加权后	0.9(0.7 ~ 1.0)	1.0(0.8 ~ 1.0)	0.08
去甲肾上腺素剂量($\mu g \cdot kg^{-1} \cdot min^{-1}$)	0.04(0.02 ~ 0.18)	0.12(0.02 ~ 0.28)	0.03
咪达唑仑剂量(mg/d)	78(42 ~ 120)	89(68 ~ 185)	0.12
丙泊酚剂量(mg/d)	150(80 ~ 210)	190(130 ~ 250)	0.08
瑞芬太尼剂量($\mu g \cdot kg^{-1} \cdot min^{-1}$)	0.01(0 ~ 0.05)	0.05(0 ~ 0.08)	0.00

三、肠内喂养不足危险因素的多因素分析

由表 2 可见喂养成功组去甲肾上腺素、丙泊酚、瑞芬太尼使用量小于喂养不足组，两组间比较具有显著性差异。将以上指标进行 Logistic 多因素分析，结果发现高剂量的去甲肾上腺素、丙泊酚、瑞芬太尼使用均是重症患者肠内喂养不足的独立危险因素。结果见表 2。

表 2　肠内喂养不足危险因素多因素分析

变量	回归系数	标准误	Wald	P	OR	95% CI
去甲肾上腺素	3.102	1.286	4.727	0.012	4.273	2.405 ~ 23.112
丙泊酚	1.133	0.742	4.251	0.031	2.366	1.098 ~ 4.877
瑞芬太尼	2.164	1.126	5.302	0.005	8.700	1.627 ~ 17.627

讨　　论

本研究结果提示即使在护理人员充足的 ICU 内，仍然有相当一部分患者未能完成肠内喂养计划，而这一部分患者往往使用更多的去甲肾上腺素、丙泊酚和瑞芬太尼治疗。既往研究证实在不同 ICU 内，肠内喂养计划的完成存在较大差别[5-8]。这些研究均以实际喂养量/计划喂养量≥65%作为患者是否完成肠内喂养计划的标准，而肠内喂养中断的主要原因有腹胀、胃潴留、医疗操作或检查以及气道护理。而本研究采用了相同的标准，也得到了相似的结果。

去甲肾上腺素是 ICU 内危重患者的常用药物，Oliveira 等[12]和 Mentec 等[13]分别在各自的研究中提出血管升压药物的使用将导致患者不能正常地摄入肠内营养，考虑原因为血管升压药物高剂量使用本身就代表患者病情较危重，并且有可能会加重患者的胃肠道缺血，影响胃肠道功能。而 Mancl 等[14]的研究虽然证实使用去甲肾上腺素患者接受肠内喂养的安全性，但同时也指出去甲肾上腺素的剂量会影响患者对肠内喂养的耐受性。本研究中喂养完成组去甲肾上腺素的剂量小于喂养不足组，且两组间有显著差异，Logistic 分析也证明去甲肾上腺素的使用是导致肠内喂养不足的独立危险因素。需要特别指出的是，本研究并未纳入血流动力学非常不稳定、存在明显酸中毒或需要持续液体复苏的患者，去甲肾上腺素最大用量为 0.3 $\mu g \cdot kg^{-1} \cdot min^{-1}$。即使如此，去甲肾上腺素的用量在本研究中也明显影响了肠内喂养的完成。

既往研究已经证明镇静药物的使用将影响肠内喂养的完成，认为镇静药物的使用会导致胃肠蠕动的减少[15]。本研究发现丙泊酚的使用是导致肠内喂养不足的独立危险因素。当然，疾病本身的严重程度也决定着对镇静药物需求的多少，严重感染的患者存在胃肠道的低灌注，也会影响胃肠道的蠕动功能。因此，我们无法肯定到底是镇静药物本身导致的肠内喂养不足还是患者自身的疾病严重程度造成的影响。

瑞芬太尼是 ICU 内常用的短效阿片类镇痛药物，其主要不良反应有恶心、腹胀、呕吐。虽然目前尚无专门针对持续泵注短效阿片类药物对重症患者胃肠道耐受性的研究，但本研究发现喂养完成组患者瑞芬太尼用量少于不足组，两组间比较有显著差异，而且 Logistic 分析也证实瑞芬太尼用量大是导致重症患者肠内喂养不足的独立危险因素。

综上所述，ICU 内重症患者仍存在肠内喂养不足的现象，去甲肾上腺素、丙泊酚、瑞芬太尼的使用是导致喂养不足的独立危险因素，对于这一类患者，应重视对胃肠道耐受性的观察，避免发生吸入性肺炎等并发症，并结合患者具体情况，决定是否加用肠外营养治疗。

(C)1994-2024 China Academic Journal Electronic Publishing House. All rights reserved.　http://www.cnki.net

参 考 文 献

1 Binnekade JM, Tepaske R, Bruynzeel P, et al. Daily enteral feeding practice on the ICU: attainment of goals and interfering factors. Crit Care, 2005, 9: R218-R225.

2 McClave SA, Sexton LK, Spain DA, et al. Enteral tube feeding in the intensive care unit: factors impeding adequate delivery. Crit Care Med, 1999, 27: 1252-1256.

3 Stapleton RD, Jones N, Heyland DK. Feeding critically ill patients: what is the optimal amount of energy? Crit Care Med, 2007, 35: S535-S540.

4 Woo SH, Finch CK, Broyles JE, et al. Early vs delayed enteral nutrition in critically ill medical patients. Nutr Clin Pract, 2010, 25: 205-211.

5 Kyle UG, Genton L, Heidegger CP, et al. Hospitalized mechanically ventilated patients are at higher risk of enteral underfeeding than non-ventilated patients. Clin Nutr, 2006, 25: 727-735.

6 De Jonghe B, Appere-De-Vechi C, Fournier M, et al. A prospective survey of nutritional support practices in intensive care unit patients: what is prescribed? What is delivered? Crit Care Med, 2001, 29: 8-12.

7 O´Meara D, Mireles-Cabodevila E, Frame F, et al. Evaluation of delivery of enteral nutrition in critically ill patients receiving mechanical ventilation. Am J Crit Care, 2008, 17: 53-61.

8 van den Broek P, Rasmussen-Conrad EL, Naber A, et al. What you think is not what they get: significant discrepancies between prescribed and administered doses of tube feeding. Br J Nutr, 2009, 101: 68-71.

9 Bou R, Gomar S, Hervás F, et al. Eradication of a nosocomial outbreak of multidrug-resistant Acinetobacter baumannii infections after adjusting nursing workloads and reinforcing specific precautions. Enferm Infecc Microbiol Clin, 2013, 31: 854-589.

10 Schwab F, Meyer E, Geffers C, et al. Understaffing, overcrowding, inappropriate nurse: ventilated patient ratio and nosocomial infections: which parameter is the best reflection of deficits? J Hosp Infect, 2012, 80: 133-139.

11 Honda C, Freitas F, Stanich P, et al. Nurse to Bed Ratio and Nutrition Support in Critically Ill Patients. Am J Crit Care, 2013, 22: e71-e78.

12 Oliveira SM, Burgos M, Santos E, et al. Gastrointestinal complications and protein-calorie adequacy in intensive care unit enteral nutrition patients. Rev Bras Ter Intensiva, 2010, 22: 270-273.

13 Mentec H, Dupont H, Bocchetti M, et al. Upper digestive intolerance during enteral nutrition in critically ill patients: frequency, risk factors, and complications. Crit Care Med, 2001, 29: 1955-1961.

14 Mancl EE, Muzevich KM. Tolerability and Safety of Enteral nutrition in critically ill patients receiving intravenous vasopressor therapy. J Parent Enteral Nutr, 2013, 37: 641-651.

15 Heyland D, Cook DJ, Winder B, et al. Enteral nutrition in the critically ill patient: a prospective survey. Criti Care Med, 1995, 23: 1055-1060.

(收稿日期: 2014-01-14)
(本文编辑: 张元芬)

· 读者 · 作者 · 编者 ·

文稿中须写成斜体的外文字符

1. 生物学中拉丁学名的属名和种名包括亚属、亚种、变种应斜体，例如大肠杆菌 *Escherichia coli*、幽门螺杆菌 *Helibacter pylori*、肺炎克雷伯菌 *Klebsilla pneumoniae*。

2. 各种基因的缩写符号应斜体(基因表达产物缩写符号应写成正体)，例如人脆性智力低下基因 1 *FMRI*1、原癌基因 *RAF*1(人)、病癌基因 *V-RAF-*1(鼠)、抑癌基因 *p*53(鼠)等。

3. 限制性内切核酸酶缩写符号中前 3 个字母应斜体，例如 *Hind* Ⅲ、*BamH* Ⅰ、*Sal* 等。

4. 各种统计学符号应斜体，例如样本数 *n*、*t* 检验、*F* 检验、概率 *P*、相关系数 *r* 等。

5. 各种物理量的量符号应斜体(pH 用正体外)，例如长度 *L*、面积 *A*(或 *S*)、体积 *V*、质量 *m*、时间 *t*、压力 *p*、相对分子质量 *M*、物质的量浓度 *c* 等。

6. 化学中表示旋光性、分构型、构象、取代基位等符号应为斜体，例如左旋 *L*-、右旋 *D*-、临位 *o*-、对位 *p*-、反位 *trans*-、顺式 *cis*- 等。

7. 数学中用字母表示的数和一般函数应斜体。

8. 英文中使用的某些拉丁词应斜体，例如 *vs*、*in situ*、*in vivo*、*in vitro* 等。

本刊编辑部

(C)1994-2024 China Academic Journal Electronic Publishing House. All rights reserved. http://www.cnki.net
