# Supplementary material for: Development and external validation of machine learning models for the early prediction of malnutrition in critically ill patients: a prospective observational study
Source: BMC Med Inform Decis Mak. 2025 Jul 3;25:248. doi: 10.1186/s12911-025-03082-9 (PMC12225150; doi:10.1186/s12911-025-03082-9)
Supplement: Supplementary file 18 — Supplementary Material 18 [file 12911_2025_3082_MOESM18_ESM.pdf]

· 论著 ·

重症病人肠内营养中断现状及其对热卡达标的影响研究

左俊焘[1,2]，李嘉琪[1,2]，徐　瑶[3]，蔡丽娜[1,2]，吴翠丽[2]，郑恒宇[1,2]，孙　蜓[3]，叶向红[2]
（1. 南京大学医学院，江苏南京210093；2. 南京大学医学院附属金陵医院/东部战区总医院全军普通外科研究所，江苏南京210002；3. 蚌埠医学院研究生院，安徽蚌埠233000）

［摘要］ **目的**：对重症病人肠内营养中断现状进行调查并探讨中断时间与次数对热卡达标的影响。 **方法**：回顾性收集2022年1月至2022年11月入住南京大学医学院附属金陵医院普通外科ICU实施肠内营养的重症病人，记录病人1周内行肠内营养的相关资料，包括每日实际摄入热卡、肠内营养中断的原因、次数与时间。采用多元线性回归分析肠内营养7 d达标天数的相关因素。 **结果**：共纳入115例重症病人，肠内营养7 d内总中断次数为136次，总中断时间为512.94 h。肠内营养中断发生频率最高的原因是外出检查（33%），其次分别是喂养不耐受（15%）与医生操作（13%）；肠内营养中断的持续时间因原因而异，包括手术或术前准备10.20（8.81, 18.11）h、喂养不耐受7.33（1.34, 21.71）h、气道操作4.30（0.93, 10.84）h等。多元线性回归分析显示肠内营养7 d达标天数与中断时间和次数呈负相关（$P < 0.05$）。 **结论**：肠内营养中断常见的原因是外出检查、喂养不耐受、医生操作等，肠内营养中断时间越长、中断次数越多，热卡摄入不足的风险越高。医护人员尽量避免肠内营养中断事件的发生从而改善病人的临床营养结局。

［关键词］ 重症监护病房；肠内营养；中断；热卡达标
中图分类号：R459.3　　文献标识码：A　　DOI：10.16151/j.1007-810x.2023.03.008

The interruption of enteral nutrition in critically ill patients and its effect on caloric intake

ZUO Jun-tao[1,2], LI Jia-qi[1,2], XU Yao[3], CAI Li-na[1,2], WU Cui-li[2], ZHENG Heng-yu[1,2], SUN Ting[3], YE Xiang-hong[2]
（1. *Medical School, Nanjing University, Nanjing* 210093*, Jiangsu, China*；2. *Research Institute of General Surgery, Jinling Hospital, Affiliated Hospital of Medical School, Nanjing University, Nanjing* 210002*, Jiangsu, China*；3.*Graduate School, Bengbu Medical College, Bengbu* 233000*, Anhui, China*）

［**Abstract**］ ***Objective***: To investigate the reasons, frequency and duration of enteral nutrition (EN) interruptions in critically ill patients and the effects of the interruptions on caloric intake. ***Methods***: A retrospective study was conducted at the Department of General Surgery, Jinling Hospital, between January 2022 to November 2022. A total of 115 critically ill patients who hospitalized in the intensive care unit (ICU) and received EN were enrolled in this study. The data related to EN feedings within 1 week, including the reasons, times and duration of EN interruptions, and the daily caloric intake of the patients, were collected. Multiple linear regression analyses were done to identify the factors related to the days of reaching caloric targets within 7-day EN feedings. ***Results***: The total times of EN interruptions in the patients were 136, and the total EN interruption duration was 512.94 h. The most common reason for EN interruption was diagnostic examination (33%), followed by feeding intolerance (15%) and other medical treatments (13%). The duration of EN interruption was varied owing to different reasons, in which the surgical operations and preoperative preparation accounted for the longest interruption time (10.20 h each patient, range: 8.81 ~ 18.11 h), feeding intolerance

基金项目：军事医学创新工程项目（18CXZ040）
作者简介：左俊焘，护理硕士研究生，从事重症营养研究。E-mail：zuojuntao199838@163.com
通讯作者：叶向红，E-mail：icuyz@126.com

(C)1994-2023 China Academic Journal Electronic Publishing House. All rights reserved.　http://www.cnki.net

for 7.33 h (1.34 ~ 21.71 h), and airway manipulation for 4.30 h (0.93 ~ 10.84 h). Daily EN interruption time had significant effects on the patients' caloric intake and balance per day. Multiple linear regression analysis showed that the duration and frequency of EN interruption were negatively correlated with the days of reaching caloric goal value during 7-day EN feedings ($P < 0.05$). ***Conclusions*:** In the present study, we demonstrate that the common causes of EN interruption in critically ill patients are diagnostic testing, feeding intolerance, and medical manipulations. The duration and frequency of EN interruption have a negative impact on patients' caloric intake. The data indicate that medical and nursing staff should try to reduce the occurrence of EN interruption, which could contribute to improve the clinical outcome of EN feeding in critically ill patients.

［**Key words**］ Intensive care unit; Enteral nutrition; Interruption; Caloric goal achievement

肠内营养（enteral nutrition, EN）是胃肠功能正常但不能实现足够口服营养摄入病人的首选，能维持胃肠黏膜结构完整性，防止细菌易位，并降低全身免疫反应、应激调节和疾病严重程度[1-3]。2019年欧洲临床营养与代谢协会（European society for clinical nutrition and metabolism, ESPEN）指出危重症病人在接受EN治疗的过程中建议持续输注，不随意中断[4]。然而，重症病人会因为各种原因导致肠内营养中断（enteral nutrition interruption, ENI），如诊断检查、医护操作、喂养不耐受、置管困难等，这通常会导致营养不良和不良后果[5]。喂养不足与住院时间延长、感染和器官衰竭等并发症的高患病率以及高死亡率有关[6-8]。国际上对于ENI的定义尚未统一，Stechmiller[9]、Kim等[10]将其定义为EN中断时间持续1h以上；在EN间断输注的前提下，每天输注3次，每次持续时间30 min，30 min内病人没有获得预计的营养量。本研究旨在调查成人重症病人肠内营养中断的发生现状并进行原因分析，探讨中断时间与次数对热卡达标的影响，为今后制定肠内营养中断处理流程提供依据。

1 资料和方法

1.1 研究对象　选取2022年1月至2022年11月南京大学医学院附属金陵医院普通外科ICU收治的115例行肠内营养的重症病人。纳入标准：①年龄≥18岁；②入住ICU且急性生理与慢性健康（acute physiology and chronic health evaluation, APACHE II）评分≥8分。排除标准：①过去3 d内曾接受EN治疗；②长期使用激素或免疫抑制剂；③恶性肿瘤正在行放、化疗或特殊治疗；④病情无法逆转、终末期；⑤妊娠期妇女。剔除标准：①可自主进食，无需营养治疗；②EN治疗天数< 7 d；③资料记录不全者。本研究已通过医院临床试验伦理委员会批准（2022DZKY-111-01）。

1.2 数据收集

1.2.1 一般资料调查　调查病人的一般资料包括性别、年龄、体质量、体质指数（body mass index, BMI）、APACHE II评分、序贯器官衰竭估计评分（sequential organ failure assessment, SOFA）、改良危重症营养风险评分（modified nutrition risk in the critically ill, mNUTRIC）。

1.2.2 营养治疗相关资料　由护理研究生登录ICU电子系统查看病例，记录营养液种类、EN开始的时间与途径、ENI情况（原因、次数与时间）、每日实际摄入热卡。目标热卡参照美国肠外和肠内营养学会（American society for parenteral and enteral nutrition, ASPEN）和美国重症医学会（The society of critical care medicine, SCCM）联合指南[11]所推荐的依据体质量的公式进行计算。根据ESPEN指南[4]推荐，以（肠内营养热卡+补充性肠外营养热卡）/热卡目标量>70%为界，将病人当日分为热卡达标（≥70%）与热卡不达标（<70%）。ENI的判定标准：在EN连续输注的过程中，成人重症病人中断持续10 min以上[12]。

1.3 质量控制　由科室护士长（护理学科带头人）与护理研究生组成调研小组共5人。根据研究目的、意义与方法、ENI的相关定义、调研工具与记录方式等对调查人员进行培训，在收集资料的过程中定时抽查调查人员对相关观察数据的理解和正确记录方式。

1.4 统计学方法　使用SPSS 25.0进行统计分析。对所有缺失值和离群值再次与原始记录核对。正态分布的计量资料采用均数±标准差（$\bar{x} \pm s$）表示；偏态分布计量资料则采用M（P25, P75）表示。计数资料采用例数、百分比（%）表示。采用多元线性回归分析EN 7 d达标天数与喂养中断次数和时间的关系，以$P < 0.05$

(C)1994-2023 China Academic Journal Electronic Publishing House. All rights reserved. http://www.cnki.net

为差异有统计学意义。

2　结　　果

2.1　调查对象一般资料　本研究经筛选最终共纳入115病人，流程图见图1。其中胰腺炎102例(88.7%)，肠瘘5例(4.3%)，其它8例(7.0%)；行早期EN 97例(84.3%)；EN途径为鼻肠管76例(66.1%)，鼻胃管34例(29.6%)，空肠造瘘管5例(4.3%)，见表1。

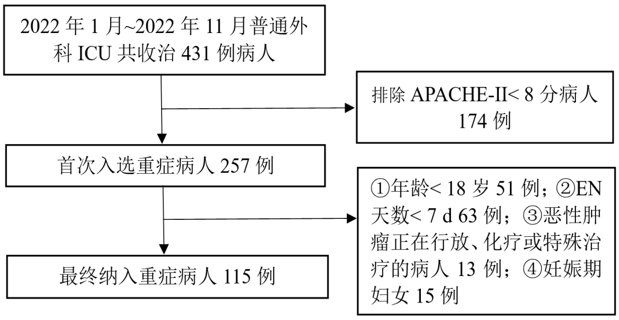

图1　病人筛选流程图

Figure 1　Flowchart of patient selection

表1　病人基本信息

Table1　Basic information of critically ill patients

基本情况	结果
性别[n(%)]	
男	77(67.0)
女	38(33.0)
年龄(岁, $\bar{x}\pm s$)	45.90 ± 14.37
体质量(kg, $\bar{x}\pm s$)	73.35 ± 16.48
体重指数(kg/m^2, $\bar{x}\pm s$)	25.13 ± 4.68
病种[n(%)]	
胰腺炎	102(88.7)
肠瘘	5(4.3)
其它	8(7.0)
APACHE II评分[分, M(P25, P75)]	12(9, 16)
SOFA评分[分, M(P25, P75)]	4(3, 6)
mNUTRIC评分[分, M(P25, P75)]	2(1, 3)
EN开始的时间[n(%)]	
≤48 h	97(84.3)
> 48 h	18(15.7)
EN途径[n(%)]	
鼻肠管	76 (66.1)
鼻胃管	34(29.6)
空肠造瘘管	5(4.3)

2.2　重症病人肠内营养中断现状　115例重症病人EN 7 d内总中断次数为136次，总中断时间为512.94 h。ENI发生频率最高的原因是外出检查45次(33%)、其次是喂养不耐受21次(15%)、医生操作18次(13%)、不明原因15次(11%)、基础护理14次(10%)、管道因素8次(6%)、气道操作6次(4%)、CRRT相关治疗5次(4%)、手术或术前准备4次(3%)，见图2；ENI持续时间最长的原因是手术或术前准备10.20(8.81, 18.11)h、其次是喂养不耐受7.33(1.34, 21.71)h、气道操作4.30(0.93, 10.84)h，不明原因2.60(1.84, 6.44)h、CRRT相关治疗2.43(0.88, 3.71)h、管道因素1.95(1.38, 6.41)h、医生操作1.71(1.40, 1.91)h、外出检查0.90(0.58, 1.22)h、基础护理0.86(0.67, 1.35)h，见表2。

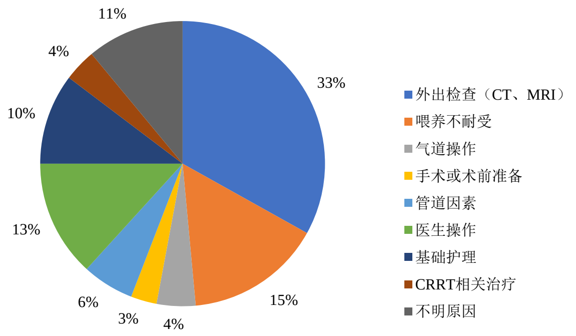

图2　肠内营养中断原因

Figure 2　Reasons for EN interruption

表2　肠内营养中断持续时间

Table 2　Duration of enteral nutrition interruption per episode

肠内营养中断原因	时间[h, M(P25, P75)]
总计	1.32(0.42, 3.72)
外出检查(CT、MRI)	0.90(0.58, 1.22)
喂养不耐受	7.33(1.34, 21.71)
气道操作	4.30(0.93, 10.84)
手术或术前准备	10.20(8.81, 18.11)
管道因素	1.95(1.38, 6.41)
医生操作	1.71(1.40, 1.91)
基础护理	0.86(0.67, 1.35)
CRRT相关治疗	2.43(0.88, 3.71)
不明原因	2.60(1.84, 6.44)

2.3　重症病人EN 7d达标天数多元线性回归分析　以EN 7 d达标天数作为因变量，EN 7 d总中断次数与时间为自变量进行多元线性回归分析，见表3。

3　讨　　论

重症病人入ICU后，滋养性肠内营养的医嘱量都很难完成，主要原因是ENI较频繁[13]。研究显示，54% ~ 84%ICU病人遭受ENI[5]。本研究结果表明

(C)1994-2023 China Academic Journal Electronic Publishing House. All rights reserved.　http://www.cnki.net

表3 重症病人EN 7 d达标天数多元线性回归分析

Table 3 Multiple linear regression results of EN 7 d compliance days

变量	标准误	P值	95%CI
常量	0.297	< 0.001	4.158 ~ 5.336
中断次数	0.221	0.005	-1.076 ~ -0.202
中断时间	0.027	0.004	-0.134 ~ -0.206

ENI发生频率最高的原因是外出检查（CT、MRI），其余依次是喂养不耐受、医生操作等，与Erika等[5]研究结果相似。ENI持续时间相对较长的事件包括手术或术前准备、喂养不耐受和气道操作，持续时间相对较短的事件包括基础护理、外出检查、医生操作，与Uozumi等[14]研究结果不同，可能与相关研究对ENI的定义不同有关。本研究纳入人群包含重症急性胰腺炎病人占总人群的88.7%，SAP病人由于腹腔积液、积气造成腹内压增高，从而导致胃肠功能受损易出现呕吐或反流、腹泻、胃潴留等喂养不耐受症状[15-16]，建议临床医护人员主动定时使用肠内营养耐受性评估与管理工具，对不耐受症状进行分级再针对处理，从而减少ENI的发生[17-18]。

本研究结果显示，重症病人中断次数与EN 7 d达标天数负相关，表明ENI发生频率越高，EN 7 d达标天数越少。尽管有学者认为ICU期间ENI难以避免[12]，但McClave等[11]研究显示65%的ENI是可以避免的。如为重症卧床病人翻身、鼻胃管堵管、气道管理、物理康复、设备故障或延迟、未及时添加营养液等导致的中断[19-20]。一项前瞻性研究结果显示，因重症病人进行气道相关操作所造成的ENI高达40%，每人平均中断时间达7.4 h/d[21]。今后应基于循证提取证据构建方案，并通过小组讨论和专家函询逐步形成ENI预防管理方案，为ICU医护人员提供有效的ENI干预策略进而减少中断事件的发生。

大部分重症病人病情严重且病情变化迅速，容易发生感染、休克等情况，导致对病人需进行较多的临床操作程序，这就会造成中断时间的延长，从而给病人带来更大的经济负担[22]。本研究结果表明ENI持续时间越长，EN 7 d达标天数越少。临床对ICU病人进行EN时，会遭遇许多导致中断的操作，操作结束后护士因临床工作繁重未及时恢复EN，导致其中断发生且时间延长[20, 23-24]。目前较多ICU在进行EN时保持恒定的输注速度且24 h固定不变，即基于速率的肠内营养输注方案，但会导致因频繁输注中断造成营养供应缺失[25]。国内外有关研究提出基于容量的肠内营养输注方案（volume based feeding, VBF），即在发生中断后需重新启动EN时，根据剩余时间和剩余摄入量调整输注速度，从而弥补因中断所造成的喂养不足[26-28]。2项Meta分析提供了强有力的证据来验证VBF方案优化营养供给的有效性和安全性，研究结果显示VBF方案显著改善了危重症病人热量与蛋白质的输送，且胃肠道并发症的发生率不会随之增加[24, 29]。因此，当病人进行EN的过程中遭受各自人为因素导致的中断后，医护人员应根据每日输注目标重新计算喂养速率。

调查研究显示ICU护士对肠内营养有一定程度的认知，但对其对病人预后的影响并不清楚，导致其ENI时有发生[23, 30-31]。本研究结果表明不明原因导致的ENI占总中断例数11.03%，高于国外研究[5, 32]，可能与部分护理人员对ENI的意识不够有关。国内一项调查研究[33]发现ICU护士对ENI的态度处于较高水平，但对ENI基本知识较缺乏，部分护士无主动学习的意识且执行能力较差。米元元等[34]根据知识、信念、行为三个维度制定了ICU医护人员肠内营养中断管理知信行量表，该量表经检验具有良好的信效度，可客观反映ICU医护人员对肠内营养中断知信行水平，为制定针对性强的培训方案提供依据。因此，建议针对不同层次的护理人员制定合理的培训目标以提升护理人员对肠内营养中断的认知水平。

本研究发现重症病人ENI常见的原因为外出检查（CT、MRI）、喂养不耐受、医生操作等。ENI导致重症病人热卡摄入不足和热卡难以达标。因此，多学科应加强合作并进行营养知识培训，提高重症病人热卡达标率。本研究尚存在一定局限性，病种覆盖欠完整，可能导致结果偏倚；未考虑中断喂养后提高输注速度是否能更好地达标；未进一步探讨ENI对病人预后的影响，今后可开展多中心、大样本研究进行完善。

【参考文献】

[1] 黎介寿. 肠内营养与肠屏障功能. 肠外与肠内营养, 2016,23(5): 257-259.

[2] Reintam BA, Starkopf J, Alhazzani W, et al. Early enteral nutrition in critically ill patients: ESICM clinical practice guidelines. Intensive Care Med, 2017,43(3):380-398.

[3] Boullata JI, Carrera AL, Harvey L, et al. ASPEN Safe Practices for Enteral Nutrition Therapy [Formula: see text]. JPEN J Parenter Enteral Nutr, 2017,41(1):15-103.

[4] Singer P, Blaser AR, Berger MM, et al. ESPEN guideline on

(C)1994-2023 China Academic Journal Electronic Publishing House. All rights reserved. http://www.cnki.net

clinical nutrition in the intensive care unit. Clin Nutr, 2019,38(1):48-79.

[5] Salciute-Simene E, Stasiunaitis R, Ambrasas E, et al. Impact of enteral nutrition interruptions on underfeeding in intensive care unit. Clin Nutr, 2021,40(3):1310-1317.

[6] Ndahimana D, Kim EK. Energy Requirements in Critically Ill Patients. Clin Nutr Res, 2018,7(2):81-90.

[7] Powers J, Samaan K. Malnutrition in the ICU patient population. Crit Care Nurs Clin North Am, 2014,26(2):227-242.

[8] Javid Z, Shadnoush M, Khadem-Rezaiyan M, et al. Nutritional adequacy in critically ill patients: Result of PNSI study. Clin Nutr, 2021,40(2):511-517.

[9] Stechmiller J, Treloar DM, Derrico D, et al. Interruption of enteral feedings in head injured patients. J Neurosci Nurs, 1994,26(4):224-229.

[10] Kim H, Stotts NA, Froelicher ES, et al. Adequacy of early enteral nutrition in adult patients in the intensive care unit. J Clin Nurs, 2012,21(19-20):2860-2869.

[11] McClave SA, Taylor BE, Martindale RG, et al. Guidelines for the Provision and Assessment of Nutrition Support Therapy in the Adult Critically Ill Patient: Society of Critical Care Medicine (SCCM) and American Society for Parenteral and Enteral Nutrition (A.S.P.E.N.). JPEN J Parenter Enteral Nutr, 2016,40(2):159-211.

[12] Peev MP, Yeh DD, Quraishi SA, et al. Causes and consequences of interrupted enteral nutrition: a prospective observational study in critically ill surgical patients. JPEN J Parenter Enteral Nutr, 2015,39(1):21-27.

[13] Kuslapuu M, Jõgela K, Starkopf J, et al. The reasons for insufficient enteral feeding in an intensive care unit: A prospective observational study. Intensive Crit Care Nurs, 2015,31(5):309-314.

[14] Uozumi M, Sanui M, Komuro T, et al. Interruption of enteral nutrition in the intensive care unit: a single-center survey. J Intensive Care, 2017,5:52.

[15] Bejarano N, Navarro S, Rebasa P, et al. Intra-abdominal pressure as a prognostic factor for tolerance of enteral nutrition in critical patients. JPEN J Parenter Enteral Nutr, 2013,37(3):352-360.

[16] Kirkpatrick AW, Roberts DJ, De Waele J, et al. Intra-abdominal hypertension and the abdominal compartment syndrome: updated consensus definitions and clinical practice guidelines from the World Society of the Abdominal Compartment Syndrome. Intensive Care Med, 2013,39(7):1190-1206.

[17] 叶向红, 彭南海, 江方正, 等. 重症急性胰腺炎合并腹腔高压患者早期肠内营养耐受性的管理. 中华护理杂志, 2016,51(12):1439-1442.

[18] 朱金凤, 罗 月, 姚惠萍, 等. 基于信息化的肠内营养耐受性动态管理对重症病人喂养效果的影. 肠外与肠内营养, 2020,27(2):104-108.

[19] O'Meara D, Mireles-Cabodevila E, Frame F, et al. Evaluation of delivery of enteral nutrition in critically ill patients receiving mechanical ventilation. Am J Crit Care, 2008,17(1):53-61.

[20] 米元元, 卢 洁. 重症患者肠内营养中断影响因素及干预策略的研究进展. 护士进修杂志, 2022,37(10):886-891.

[21] Ramakrishnan N, Daphnee DK, Ranganathan L, et al. Critical care 24 × 7: But, why is critical nutrition interrupted?. Indian J Crit Care Med, 2014,18(3):144-148.

[22] 宫雪梅, 叶向红, 武 燕, 等. 重症患者早期肠内营养喂养中断现状的调查研究. 中华现代护理杂志, 2019(13):1646-1650.

[23] 宫雪梅, 叶向红, 邢 娟, 等. 重症病人早期肠内营养喂养中断原因的系统评价. 肠外与肠内营养, 2018,25(5):285-290.

[24] Wang L, Wang Y, Li HX, et al. Optimizing enteral nutrition delivery by implementing volume-based feeding protocol for critically ill patients: an updated meta-analysis and systematic review. Crit Care, 2023,27(1):173.

[25] McClave SA, Martindale RG, Rice TW, et al. Feeding the critically ill patient. Crit Care Med, 2014,42(12):2600-2610.

[26] Haskins IN, Baginsky M, Gamsky N, et al. Volume-Based Enteral Nutrition Support Regimen Improves Caloric Delivery but May Not Affect Clinical Outcomes in Critically Ill Patients. JPEN J Parenter Enteral Nutr, 2017,41(4):607-611.

[27] Taylor B, Brody R, Denmark R, et al. Improving enteral delivery through the adoption of the "Feed Early Enteral Diet adequately for Maximum Effect (FEED ME)" protocol in a surgical trauma ICU: a quality improvement review. Nutr Clin Pract, 2014,29(5):639-648.

[28] 路艳霞, 马 靓. 基于容量的肠内营养输注方案在ICU患者中的应用. 中华急危重症护理杂志, 2020,1(1):53-57.

[29] 喻海涛, 蔡 丽, 陈 璐, 等. 基于容量的肠内营养输注方案对ICU患者营养摄入影响的Meta分析. 中华急危重症护理杂志, 2023,4(2):106-111.

[30] Marshall AP, Cahill NE, Gramlich L, et al. Optimizing nutrition in intensive care units: empowering critical care nurses to be effective agents of change. Am J Crit Care, 2012,21(3):186-194.

[31] Cahill NE, Murch L, Cook D, et al. Barriers to feeding critically ill patients: a multicenter survey of critical care nurses. J Crit Care, 2012,27(6):727-734.

[32] Engel JM, Muhling J, Junger A, et al. Enteral nutrition practice in a surgical intensive care unit: what proportion of energy expenditure is delivered enterally?. Clin Nutr, 2003, 22(2):187-192.

[33] 王 娟, 徐 静, 苏靖雯, 等. ICU护士肠内营养喂养中断知信行现状及其与自我效能感的中介研究. 护理学, 2021, 10(3):178-184.

[34] 米元元, 田 飞, 包 磊, 等. ICU医护人员肠内营养中断管理知信行量表的编制及信效度检验. 护理学杂志, 2022,37(19):82-86.

（2023-02-28收稿；2023-04-07修回）
（责任编辑：颜文娟；英文编辑：王晨阳）

(C)1994-2023 China Academic Journal Electronic Publishing House. All rights reserved. http://www.cnki.net
