# Supplementary material for: Development and external validation of machine learning models for the early prediction of malnutrition in critically ill patients: a prospective observational study
Source: BMC Med Inform Decis Mak. 2025 Jul 3;25:248. doi: 10.1186/s12911-025-03082-9 (PMC12225150; doi:10.1186/s12911-025-03082-9)
Supplement: Supplementary file 19 — Supplementary Material 19 [file 12911_2025_3082_MOESM19_ESM.docx]

**Table S1. Literature Screening Tracker**

| **Number** | **DOI/PMID** | **Article Title** | **Database** | **Initial Screening**  **(Include?)** | **Exclusion Reason Category** | **Full-Text Evaluation**  **(Include?)** | **Final Inclusion Status** |
| --- | --- | --- | --- | --- | --- | --- | --- |
| 1 | 10.5935/0103-507X.20190067 | Risk factors for protein-caloric inadequacy in patients in an intensive care unit | PubMed | Yes | Not Applicable | Yes | Included |
| 2 | 10.1093/ajcn/nqab023 | Factors associated with delayed enteral nutrition in the intensive care unit: A propensity score-matched retrospective cohort study | PubMed | Yes | Not Applicable | Yes | Included |
| 3 | 10.1186/s13054-017-1805-z | Current clinical nutrition practices in critically ill patients in Latin America: a multinational observational study | PubMed | Yes | Not Applicable | Yes | Included |
| 4 | 10.1111/jan.12051 | Malnutrition in Austrian hospital patients. Prevalence, risk factors, nursing interventions, and quality indicators: A descriptive multicentre study | PubMed | Yes | Not Applicable | Yes | Included |
| 5 | 23370829 | Gastrointestinal symptoms during the first week of intensive care are associated with poor outcome: A prospective multicentre study | PubMed | Yes | Not Applicable | Yes | Included |
| 6 | 31061655 | Implementing dietary modifications and assessing nutritional adequacy of diets for inflammatory bowel disease | PubMed | Yes | Not Applicable | Yes | Included |
| 7 | 22085763 | Identifying critically ill patients who benefit the most from nutrition therapy: the development and initial validation of a novel risk assessment tool | PubMed | Yes | Not Applicable | Yes | Included |
| 8 | 10.1002/ncp.10214 | Determination of nutrition risk and status in critically ill patients: what are our considerations? | PubMed | Yes | Not Applicable | Yes | Included |
| 9 | 10.16151/j.1007-810x.2022.03.008 | Analysis of influencing factors of early enteral nutrition not up to goal in critically ill patients | CNKI | Yes | Not Applicable | Yes | Included |
| 10 | 10.14033/j.cnki.cfmr.2020.36.018 | Current status and influencing factors of malnutrition in ICU critically ill patients | CNKI | Yes | Not Applicable | Yes | Included |
| 11 | 10.3969/j.issn.1009-6493.2015.10.007 | Analysis of noncompliance condition of early enteral nutrition feeding of severe stroke patients and its influencing factor | CNKI | Yes | Not Applicable | Yes | Included |
| 12 | 10.7507/1671-6205.2014096 | Risk factors analysis for inadequate enteral nutrition in critically ill patients | CNKI | Yes | Not Applicable | Yes | Included |
| 13 | 10.16151/j.1007-810x.2023.03.008 | The interruption of enteral nutrition in critically ill patients and its effect on caloric intake | CNKI | Yes | Not Applicable | Yes | Included |
| 14 | 10.27258/d.cnki.gnxyc.2022.000421 | Analysis of nutritional status and influencing factors of patients with sepsis | CNKI | Yes | Not Applicable | Yes | Included |
| 15 | 10.3760/cma.j.issn.1673-4203.2010.03.006 | Influencing factors of the early enteral nutrition in patients of severe acute pancreatitis | SinoMed | Yes | Not Applicable | Yes | Included |
| 16 | 10.3760/cma.j.issn.1672-7088.2017.03.010 | Adequate feeding of early enteral nutrition in critical patients with cancer | SinoMed | Yes | Not Applicable | Yes | Included |
| 17 | 10.3969/j.issn.1002-1949.2010.06.007 | Logistic regression analysis of the factors affecting the early enteral nutrition up to goal in critically ill patients | Wanfang | Yes | Not Applicable | Yes | Included |
| 18 | 10.3760/cma.j.issn.2095-4352.2016.06.017 | Analysis of nutritional risk assessment and prognosis in critically ill patients | SinoMed | Yes | Not Applicable | Yes | Included |
| 19 | 10.16506/j.1009-6639.2019.08.013 | Analysis of risk factors for enteral nutrition feeding in patients with ICU and preventive measures | Wanfang | Yes | Not Applicable | Yes | Included |
| 20 | 10.3346/jkms.2018.33.e10 | Prevalence of Malnutrition in Hospitalized Patients: a Multicenter Cross-sectional Study | PubMed | Yes | Not suitable research subjects | No | Excluded |
| 21 | 8216978 | Clinical risk factors for malnutrition in HIV-1-infected patients | PubMed | Yes | Not suitable research subjects | No | Excluded |
| 22 | 10823981 | Epidemiological study of malnutrition in elderly patients in acute, sub-acute and long-term care using the MNA | PubMed | Yes | Not suitable research subjects | No | Excluded |
| 23 | 29935370 | Food insecurity, weight and nutritional status among older adults attending senior centres in Lisbon | PubMed | Yes | Not suitable research subjects | No | Excluded |
| 24 | 16582029 | Gender differences in factors associated with nutritional status of older medical patients | PubMed | Yes | Not suitable research subjects | No | Excluded |
| 25 | 28327565 | Impaired nutritional status in geriatric trauma patients | PubMed | Yes | Not suitable research subjects | No | Excluded |
| 26 | 31443557 | Inflammation, Appetite and Food Intake in Older Hospitalized Patients | PubMed | Yes | Not suitable research subjects | No | Excluded |
| 27 | 30482563 | Lipid paradox in patients with acute myocardial infarction: Potential impact of malnutrition | PubMed | Yes | Not suitable research subjects | No | Excluded |
| 28 | 28717814 | Malnutrition in Hospitalised Older Adults: A Multicentre Observational Study of Prevalence, Associations and Outcomes | PubMed | Yes | Not suitable research subjects | No | Excluded |
| 29 | 22953614 | MEasuring Nutrition risk in hospitalized patients: MENU, a hospital-based prevalence survey | PubMed | Yes | Not suitable research subjects | No | Excluded |
| 30 | 30272846 | Prevalence and associated risk factors of malnutrition among hospitalized adults in a multisite study in Ho Chi Minh city Viet Nam | PubMed | Yes | Not suitable research subjects | No | Excluded |
| 31 | 36173228 | Risk factors for inpatient malnutrition and length of stay assessed by 'NutritionDay' in China | PubMed | Yes | Not suitable research type | No | Excluded |
| 32 | 22964126 | Risk factors of malnutrition at hospital admission | PubMed | Yes | Not suitable research type | No | Excluded |
| 33 | 11518553 | Simple nutrition screening tools for healthcare facilities: development and validity assessment | PubMed | Yes | Not suitable research type | No | Excluded |
| 34 | 10.1080/07315724.2020.1774821 | MUST-Plus: A Machine Learning Classifier That Improves Malnutrition Screening in Acute Care Facilities | Cochrane Library | Yes | Not suitable research subjects | No | Excluded |
| 35 | 10.3969/j.issn.1673-5765.2010.08.006 | Risk Factors of Malnutrition in Hospitalized Patients with Acute Stroke | CNKI | Yes | Not suitable research subjects | No | Excluded |
| 36 | 10.3969/j.issn.1009-6493.2017.34.030 | Status quo of nutritional risk and its influencing factors analysis in patients with diabetes mellitus complicated with acute myocardial infarction | CNKI | Yes | Not suitable research subjects | No | Excluded |
| 37 | 10.14126/j.cnki.1008-7044.2022.01.026 | Factors Influencing Malnutrition in Severe COVID-19 Patients | CNKI | Yes | Not suitable research subjects | No | Excluded |
| 38 | 10.3760/cma.j.issn.1673-4904.2018.08.009 | The nutritional risk screening and nutritional assessment and analysis of influencing factors of nutritional risk and malnutrition in patients with acute cerebral infarction | SinoMed | Yes | Not suitable research subjects | No | Excluded |
| 39 | 10.3760/cma.j.issn.1673-4904.2018.08.009 | The nutritional risk screening and nutritional assessment and analysis of influencing factors of nutritional risk and malnutrition in patients with acute cerebral infarction | SinoMed | Yes | Not suitable research subjects | No | Excluded |
| 40 | 10.12122/j.issn.1673-4254.2022.09.17 | Association of nutritional status with clinical outcomes of stroke patients with acute  anterior circulation large vessel occlusion after emergency endovascular treatment | Wanfang | Yes | Not suitable research subjects | No | Excluded |
| 41 | 10.1097/MCO.0b013e328350767e | Nutrition risk assessment in the ICU | Web of Science | Yes | Not suitable research type | No | Excluded |
| 42 | 10.1186/cc10572 | Do we need an assessment of the nutrition risk in the critically ill patient | Web of Science | Yes | Not suitable research type | No | Excluded |
| 43 | 32305181 | ESPEN Body composition in older acute stroke patients after treatment with individualized, nutritional supplementation while in hospital CoV-2 infection | PubMed | Yes | Not suitable research type | No | Excluded |
| 44 | 12087704 | Establishing nutritional guidelines for critically ill patients: Part 1 | PubMed | Yes | Not suitable research type | No | Excluded |
| 45 | 35059185 | Indicators of nutritional risk in hospital inpatients: a narrative review | PubMed | Yes | Not suitable research type | No | Excluded |
| 46 | 23186132 | Malnutrition in Austrian hospital patients. Prevalence, risk factors, nursing interventions, and quality indicators: a descriptive multicentre stud | PubMed | Yes | Not suitable research type | No | Excluded |
| 47 | 33748170 | Negative Association Between Mediterranean Diet Adherence and COVID-19 Cases and Related Deaths in Spain and 23 OECD Countries: An Ecological Study | PubMed | Yes | Not suitable research type | No | Excluded |
| 48 | 24484922 | Nutrition and hydration in older adults in critical care | PubMed | Yes | Not suitable research type | No | Excluded |
| 49 | 34140163 | Nutritional management of individuals with obesity and COVID-19: ESPEN expert statements and practical guidance | PubMed | Yes | Not suitable research type | No | Excluded |
| 50 | 15055033 | The rationale of early enteral nutrition | PubMed | Yes | Not suitable research type | No | Excluded |
| 51 | 8197254 | Risk factors for poor nutritional status | PubMed | Yes | Not suitable research type | No | Excluded |
| 52 | 32303438 | What is nutritional assessment? A quick guide for critical care clinicians | PubMed | Yes | Not suitable research type | No | Excluded |
| 53 | 36793999 | Importance of nutritional assessment tools in the critically ill patient: A systematic review | PubMed | Yes | Not suitable research type | No | Excluded |
| 54 | 29576355 | Nutrition therapy for critically ill patients across the Asia-Pacific and Middle East regions: A consensus statement | PubMed | Yes | Not suitable research type | No | Excluded |
| 55 | 10.1002/jpen.2228 | Nutrition support for critically ill patients | PubMed | Yes | Not suitable research type | No | Excluded |
| 56 | 10.1002/jpen.2267 | Guidelines for the provision of nutrition support therapy in the adult critically ill patient: The American Society for Parenteral and Enteral Nutrition | PubMed | Yes | Not suitable research type | No | Excluded |
| 57 | 12138581 | Establishing nutritional guidelines for critically ill patients: Part 2 | PubMed | Yes | Not suitable research type | No | Excluded |
| 58 | 10.1186/1471-2318-10-75 | Body composition in older acute stroke patients after treatment with individualized, nutritional supplementation while in hospital | Cochrane Library | Yes | Not suitable research type | No | Excluded |
| 59 | 10.1002/14651858.CD012340.pub2 | Early enteral nutrition (within 48 hours) versus delayed enteral nutrition (after 48 hours) with or without supplemental parenteral nutrition in critically ill adults | Cochrane Library | Yes | Not suitable research type | No | Excluded |
| 60 | 10.1016/j.clnu.2019.09.007 | Early high protein intake and mortality in critically ill ICU patients with low skeletal muscle area and -density | Cochrane Library | Yes | Not suitable research type | No | Excluded |
| 61 | 30260486 | The Effect of Higher Protein Dosing in Critically Ill Patients: A Multicenter Registry-Based Randomized Trial: The EFFORT Trial | Cochrane Library | Yes | Not suitable research type | No | Excluded |
| 62 | 10.1002/14651858.CD012276.pub2 | Enteral versus parenteral nutrition and enteral versus a combination of enteral and parenteral nutrition for adults in the intensive care unit | Cochrane Library | Yes | Not suitable research type | No | Excluded |
| 63 | 10.1371/journal.pone.0231777 | The implementation of a nutrition protocol in a surgical intensive care unit; a randomized controlled trial at a tertiary care hospital | Cochrane Library | Yes | Not suitable research type | No | Excluded |
| 64 | 10.1111/ggi.13854 | Individualized nutritional treatment for acute stroke patients with malnutrition risk improves functional independence measurement: A randomized controlled trial | Cochrane Library | Yes | Not suitable research type | No | Excluded |
| 65 | 10.1186/s40635-020-00354-8 | Malnutrition in patients with ischemic stroke | Cochrane Library | Yes | Not suitable research type | No | Excluded |
| 66 | 10.1016/j.clnesp.2018.03.123 | Use of standard enteral formula versus enteric formula with prebiotic content in nutrition therapy: A randomized controlled study among neuro-critical care patients | Cochrane Library | Yes | Not suitable research type | No | Excluded |
| 67 | 10.1007/s00134-006-0238-y | Early enteral immunonutrition vs. parenteral nutrition in critically ill patients without severe sepsis: a randomized clinical trial | Cochrane Library | Yes | Not suitable research type | No | Excluded |
| 68 | 27585532 | Early goal-directed nutrition in ICU patients (EAT-ICU): protocol for a randomised trial | Cochrane Library | Yes | Not suitable research type | No | Excluded |
| 69 | 10.1016/s0749-0704(05)70269-4 | Early nutritional support in critical illness is important | Cochrane Library | Yes | Not suitable research type | No | Excluded |
| 70 | 10.1001/jama.2013.5124 | Early parenteral nutrition in critically ill patients with short-term relative contraindications to early enteral nutrition: a randomized controlled trial | Cochrane Library | Yes | Not suitable research type | No | Excluded |
| 71 | 10.1186/s13063-022-06898-2 | Effectiveness of intensive perioperative nutrition therapy among adults undergoing gastrointestinal and oncological surgery in a public hospital: study protocol for a pragmatic randomized control trial | Cochrane Library | Yes | Not suitable research type | No | Excluded |
| 72 | 26176044 | Effects of Delayed Enteral Nutrition on Inflammatory Responses and Immune Function Competence in Critically Ill Patients with Prolonged Fasting | Cochrane Library | Yes | Not suitable research type | No | Excluded |
| 73 | 10.12669/pjms.38.1.4451 | Effects of enteral nutrition on heart function, inflammatory markers and immune function in elderly patients with chronic heart failure | Cochrane Library | Yes | Not suitable research type | No | Excluded |
| 74 | 10.1016/S0140-6736(17)32146-3 | Enteral versus parenteral early nutrition in ventilated adults with shock: a randomised, controlled, multicentre, open-label, parallel-group study (NUTRIREA-2) | Cochrane Library | Yes | Not suitable research type | No | Excluded |
| 75 | 10.1177/0148607107031002119 | Synbiotics, prebiotics, glutamine, or peptide in early enteral nutrition: a randomized study in trauma patients." JPEN. Journal of parenteral and enteral nutrition | Cochrane Library | Yes | Not suitable research type | No | Excluded |
| 76 | Conference | A useful risk assessment tool of malnutrition and its prognostic impact in patients with acute heart failure | Web of Science | Yes | Not full-text available | No | excluded |
| 77 | Conference | Assessment of malnutrition and its prognosis by risk scores in patients with acute heart failure | Web of Science | Yes | Not full-text available | No | excluded |
| 78 | Conference | Modifiable Risk Factors for Malnutrition among Older Adults Receiving Care in the Emergency Department | Web of Science | Yes | Not full-text available | No | excluded |
| 79 | Conference | Assessment of Malnutrition in emergency laparotomy patients. A QIP highlighting simple measures can improve early recognition and optimisation of high-risk patients | Web of Science | Yes | Not full-text available | No | excluded |
| 80 | 11033531 | Influence of nutritional status on development of nosocomial pneumonia in case of acute decompensation of chronic obstructive pulmonary diseases | PubMed | Yes | Not Chinese and English article | No | excluded |
| 81 | 19160902 | Nutritional Score Risk for mortality in critically ill patients (NSRR: Nutritional Score Risk Research) | Web of Science | Yes | Not Chinese and English article | No | excluded |
| 82 | 17130995 | Recommendations for the nutritional assessment of critically ill patients | Web of Science | Yes | Not Chinese and English article | No | excluded |
