# Supplementary material for: Development and external validation of machine learning models for the early prediction of malnutrition in critically ill patients: a prospective observational study
Source: BMC Med Inform Decis Mak. 2025 Jul 3;25:248. doi: 10.1186/s12911-025-03082-9 (PMC12225150; doi:10.1186/s12911-025-03082-9)
Supplement: Supplementary file 20 — Supplementary Material 20 [file 12911_2025_3082_MOESM20_ESM.docx]

**Table S2. List of Experts**

| Experts | Name | Profession | Nationality |
| --- | --- | --- | --- |
| 1 | Zhongbin Chen | Critical Care Physician | China |
| 2 | Yehua Xu | Critical Care Physician | China |
| 3 | Yuxiang Zhao | Critical Care Physician | China |
| 4 | Lu Wang | Critical Care Physician | China |
| 5 | Caixia Xie | Critical Care Physician | China |
| 6 | Hua Jiang | Critical Care Physician | China |
| 7 | Liangyu Yin | Clinical Dietitian | China |
| 8 | Yu Wang | Clinical Dietitian | China |
| 9 | Xingwei Wu | Researcher | China |
| 10 | Dongliang Yang | Researcher | China |
