# Supplementary material for: Development and external validation of machine learning models for the early prediction of malnutrition in critically ill patients: a prospective observational study
Source: BMC Med Inform Decis Mak. 2025 Jul 3;25:248. doi: 10.1186/s12911-025-03082-9 (PMC12225150; doi:10.1186/s12911-025-03082-9)
Supplement: Supplementary file 21 — Supplementary Material 21 [file 12911_2025_3082_MOESM21_ESM.docx]

**Table S3. Voting Results**

| 43 variables | Votes in favor | Votes against |
| --- | --- | --- |
| Reduced Energy Intake | 10 | 0 |
| BMI | 9 | 1 |
| Albumin | 9 | 1 |
| Hemoglobin | 9 | 1 |
| Total Protein | 8 | 2 |
| Red Blood Cell Count | 8 | 2 |
| Hematocrit | 8 | 2 |
| Whole Blood hs-CRP | 7 | 3 |
| CD4+ T Lymphocyte Count | 7 | 3 |
| Age | 7 | 3 |
| Sodium Ions | 6 | 4 |
| Potassium Ions | 6 | 4 |
| Phosphorus Ions | 6 | 4 |
| Magnesium Ions | 6 | 4 |
| Platelet Count | 6 | 4 |
| Total Bilirubin | 6 | 4 |
| Fasting Blood Glucose Value | 6 | 4 |
| Serum Urea | 6 | 4 |
| Serum Creatinine | 6 | 4 |
| Serum Uric Acid | 6 | 4 |
| Oxygen Saturation | 5 | 4 |
| PO2 | 5 | 4 |
| PH Value | 5 | 4 |
| Neutrophil Count | 5 | 5 |
| White Blood Cell Count | 5 | 5 |
| Lymphocyte Count | 5 | 5 |
| Body Temperature | 5 | 5 |
| Heart Rate | 5 | 5 |
| Respiratory Rate | 5 | 5 |
| Systolic Blood Pressure | 5 | 5 |
| Diastolic Blood Pressure | 5 | 5 |
| IL-6 | 5 | 5 |
| Procalcitonin | 5 | 5 |
| Gender | 5 | 5 |
| Chronic Gastrointestinal Symptoms | 5 | 5 |
| Acute Gastrointestinal Symptoms | 5 | 5 |
| Treatment with Sedatives | 5 | 5 |
| Treatment with Vasopressor Drugs | 5 | 5 |
| Mechanical Ventilation | 5 | 5 |
| Marital Status | 4 | 6 |
| Smoking History | 3 | 7 |
| Alcohol Consumption History | 3 | 7 |
| Surgical History | 3 | 7 |

a) Tiebreaker rationale for variables with equal votes:

IL-6 (5 vs. 5): Included per ESPEN 2023 criteria linking systemic inflammation to malnutrition.

Neutrophil Count (5 vs. 5): Prioritized due to its association with infection-related malnutrition in prior studies.

Marital Status (4 vs. 6): Excluded as social factors were deemed secondary to biochemical/biometric parameters in the final model.

Smoking/Alcohol History (3 vs. 7): Removed due to insufficient empirical support in malnutrition prediction literature.
