# Supplementary material for: Development and external validation of machine learning models for the early prediction of malnutrition in critically ill patients: a prospective observational study
Source: BMC Med Inform Decis Mak. 2025 Jul 3;25:248. doi: 10.1186/s12911-025-03082-9 (PMC12225150; doi:10.1186/s12911-025-03082-9)
Supplement: Supplementary file 22 — Supplementary Material 22 [file 12911_2025_3082_MOESM22_ESM.docx]

| Variable | Missingness (%) | Imputation Method |
| --- | --- | --- |
| BMI | 16.9% | Random Forest |
| lymphocyte count | 0.2% | Random Forest |
| IL-6 | 17.3% | Random Forest |
| procalcitonin | 10.8% | Random Forest |
| CD4+ T lymphocyte | 50.1% | Random Forest |
| hs-CRP | 2.5% | Random Forest |
| pH Value | 1.4% | Random Forest |
| PaO2 | 1.3% | Random Forest |

**Table S4. List of all candidate predictors and their missing data proportions**
