# Supplementary material for: Development and external validation of machine learning models for the early prediction of malnutrition in critically ill patients: a prospective observational study
Source: BMC Med Inform Decis Mak. 2025 Jul 3;25:248. doi: 10.1186/s12911-025-03082-9 (PMC12225150; doi:10.1186/s12911-025-03082-9)
Supplement: Supplementary file 23 — Supplementary Material 23 [file 12911_2025_3082_MOESM23_ESM.docx]

## Table S5. Variables transformation method and assignment instructions

| Variables | Variable type | Transformation method and assignment instructions |
| --- | --- | --- |
| Gender | Binary categorical variable | Label encoding, 1: male, 0: female |
| Mechanical ventilation | Binary categorical variable | Label encoding, 1: yes, 0: No |
| Treatment with vasopressor drugs | Binary categorical variable | Label encoding, 1: yes, 0: No |
| Treatment with sedatives | Binary categorical variable | Label encoding, 1: yes, 0: No |
| Reduced energy intake | Binary categorical variable | Label encoding, 1: yes, 0: No |
| Chronic gastrointestinal symptoms | Binary categorical variable | Label encoding, 1: yes, 0: No |
| Acute gastrointestinal symptoms | Binary categorical variable | Label encoding, 1: yes, 0: No |
| Age | Continuous variable | Standardizing, unit: year |
| BMI | Continuous variable | Standardizing, unit: kg/m2 |
| Total protein | Continuous variable | Standardizing, unit: g/L |
| Albumin | Continuous variable | Standardizing, unit: g/L |
| Hemoglobin | Continuous variable | Standardizing, unit: g/L |
| Red blood cell count | Continuous variable | Standardizing, unit: 1012 /L |
| White blood cell count | Continuous variable | Standardizing, unit: 109 /L |
| Neutrophil count | Continuous variable | Standardizing, unit: 109 /L |
| Lymphocyte count | Continuous variable | Standardizing, unit: 109 /L |
| Hematocrit | Continuous variable | Standardizing, unit: % |
| Fasting blood glucose value | Continuous variable | Standardizing, unit: mmol/L |
| Interleukin-6 | Continuous variable | Standardizing, unit: pg/mL |
| Procalcitonin | Continuous variable | Standardizing, unit: ng/mL |
| CD4^+^ T lymphocyte count | Continuous variable | Standardizing, unit: /uL |
| Whole blood hypersensitive C-reactive protein | Continuous variable | Standardizing, unit: mg/L |
| PH value | Continuous variable | Standardizing, unit: none |
| Arterial oxygen partial pressure | Continuous variable | Standardizing, unit: mmHg |
| Oxygen saturation | Continuous variable | Standardizing, unit: % |
| Sodium ions | Continuous variable | Standardizing, unit: mmol/L |
| Potassium ions | Continuous variable | Standardizing, unit: mmol/L |
| Magnesium ions | Continuous variable | Standardizing, unit: mmol/L |
| Phosphorus ions | Continuous variable | Standardizing, unit: mmol/L |
| Platelet count | Continuous variable | Standardizing, unit: 109 /L |
| Serum urea | Continuous variable | Standardizing, unit: umol/L |
| Serum creatinine | Continuous variable | Standardizing, unit: mmol/L |
| Serum uric acid | Continuous variable | Standardizing, unit: umol/L |
| Total bilirubin | Continuous variable | Standardizing, unit: umol/L |
| Body temperature | Continuous variable | Standardizing, unit: ℃ |
| Heart rate | Continuous variable | Standardizing, unit: beats / minute |
| Respiratory rate | Continuous variable | Standardizing, unit: breaths / minute |
| Systolic blood pressure | Continuous variable | Standardizing, unit: mmHg |
| Diastolic blood pressure | Continuous variable | Standardizing, unit: mmHg |

Note: Abbreviations, BMI = Body Mass Index.
