# Supplementary material for: Development and external validation of machine learning models for the early prediction of malnutrition in critically ill patients: a prospective observational study
Source: BMC Med Inform Decis Mak. 2025 Jul 3;25:248. doi: 10.1186/s12911-025-03082-9 (PMC12225150; doi:10.1186/s12911-025-03082-9)
Supplement: Supplementary file 25 — Supplementary Material 25 [file 12911_2025_3082_MOESM25_ESM.pdf]

RStudio

File Edit Code View Plots Session Build Debug Profile Tools Help

Go to file/function Addins

Source

Console Terminal Background Jobs

R 4.3.1 · ~/

```
R version 4.3.1 (2023-06-16 ucrt) -- "Beagle Scouts"
Copyright (C) 2023 The R Foundation for Statistical Computing
Platform: x86_64-w64-mingw32/x64 (64-bit)

R is free software and comes with ABSOLUTELY NO WARRANTY.
You are welcome to redistribute it under certain conditions.
Type 'license()' or 'licence()' for distribution details.

R is a collaborative project with many contributors.
Type 'contributors()' for more information and
'citation()' on how to cite R or R packages in publications.

Type 'demo()' for some demos, 'help()' for on-line help, or
'help.start()' for an HTML browser interface to help.
Type 'q()' to quit R.

[Workspace loaded from ~/.RData]

> # Binary outcomes(Logistic prediction models)
> # Using pmsampsize to calculate the minimum sample size required for developing a prediction model
> library(pmsampsize)
> pmsampsize(type="b", cstatistic = 0.858, parameters=39, prevalence=0.39)
Given input C-statistic = 0.858  & prevalence = 0.39
Cox-Snell R-sq = 0.3493

NB: Assuming 0.05 acceptable difference in apparent & adjusted R-squared
NB: Assuming 0.05 margin of error in estimation of intercept
NB: Events per Predictor Parameter (EPP) assumes prevalence = 0.39

           Samp_size Shrinkage Parameter CS_Rsq Max_Rsq Nag_Rsq  EPP
Criteria 1       794     0.900        39 0.3493   0.737   0.474 7.94
Criteria 2       837     0.905        39 0.3493   0.737   0.474 8.37
Criteria 3       366     0.905        39 0.3493   0.737   0.474 3.66
Final            837     0.905        39 0.3493   0.737   0.474 8.37

 Minimum sample size required for new model development based on user inputs = 837,
 with 327 events (assuming an outcome prevalence = 0.39) and an EPP = 8.37


> 
```
